# Reagent-controlled enantioselectivity switch for the asymmetric fluorination of β-ketocarbonyls by chiral primary amine catalysis†
†Electronic supplementary information (ESI) available: Experimental details including characterization date, copies of ^1^H, ^13^C, and ^19^F NMR and HPLC traces. See DOI: 10.1039/c6sc03109a



**DOI:** 10.1039/c6sc03109a

**Published:** 2016-08-26

**Authors:** Yang'en You, Long Zhang, Sanzhong Luo

**Affiliations:** a Beijing National Laboratory for Molecule Sciences (BNLMS) , Key Laboratory for Molecular Recognition and Function , Institute of Chemistry , The Chinese Academy of Sciences , Beijing 100190 , China . Email: luosz@iccas.ac.cn; b University of Chinese Academy of Sciences , Beijing , 100049 , China; c Collaborative Innovation Center of Chemical Science and Engineering , Tianjin , 300071 , China

## Abstract

A reagent-controlled enantioselectivity switch was uncovered in the asymmetric α-fluorination of β-ketocarbonyls by a chiral primary amine catalyst. By a simple swap of fluorination reagents, both enantiomers of the quaternary fluorination adducts could be obtained with good yields and high enantioselectivity. Mechanistic studies disclosed dual H-bonding and electrostatic stereocontrolling modes for the catalysis.

## Abstract 2

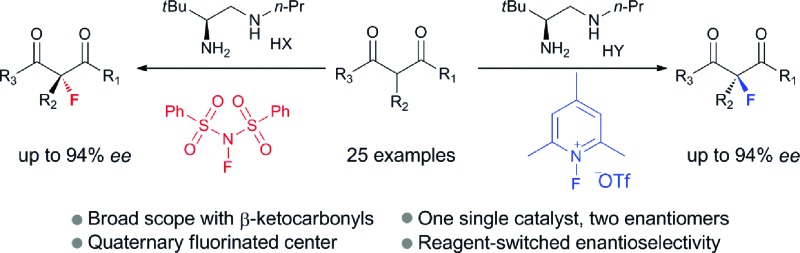
A swap of fluorination reagents led to a switch of enantioselectivity in a chiral primary amine catalyzed asymmetric α-fluorination of β-ketocarbonyls.

Asymmetric catalysis is arguably the most effective and atom-economic approach for access to optically pure compounds. Using a single chiral catalyst to get two enantiomeric products is attractive as it eliminates the need for the synthesis of catalyst enantiomers, which is not a trivial effort in most cases.^1^ Recently, a switch of enantioselectivity has been noted in a number of metal catalyzed reactions by simply varying the solvent, temperature and additives without structural modifications of the chiral ligands.^2^ However, such an external switch with good enantioselectivity for both enantiomers is still very rare for organocatalytic reactions.^3^ Nagasawa successfully achieved a solvent-switch of enantioselection in an asymmetric Mannich reaction with middle to high ee. Maruoka developed asymmetric aldol and Mannich reactions by an achiral-acid-additive induced switch. Matsubara reported a procedure-controlled enantioselectivity switch with moderate ee. Herein, we reported an enantioselective switch for synthetically important asymmetric fluorination reactions by two different stereocontrolling modes.

Catalytic enantioselective construction of carbon–fluorine bonds is of significant synthetic interest due to the prevalence of fluorinated drugs and agricultural agents.^4d,h^ In this regard, enamine catalysis has appeared as a prominent strategy for the fluorination of aldehydes and ketones. Pioneering works by the groups^7^ of Jørgensen, Barbas and MacMillan have achieved asymmetric α-fluorination of linear aldehydes with high enantioselectivity. The reaction has recently been extended to α-branched aldehydes by primary amine catalysis.^8^ In contrast, the asymmetric fluorination of ketones by amine catalysis has remained far less developed, particularly for branched ketones. Two elegant contributions along this line have been recently reported for the reactions of cyclic ketones by the research groups of MacMillan^9^ and Toste.^10^ However, the asymmetric α-fluorination of acyclic ketones remains largely undeveloped not only in aminocatalysis, but in general in asymmetric catalysis. Previously, Lewis acid catalysis (Scheme 1, I) has been frequently attempted in the asymmetric fluorination of acyclic ketones,^4–6^ but successes have only been seen with β-ketoesters bearing bulky (*e.g. t*-butyl) ester groups.^5^ We have pursued the fluorination reaction of acyclic β-ketocarbonyls using our primary amine catalysts^12^ and during this process we observed an unprecedented reagent controlled switch of enantioselectivity. Thus, by simply choosing two readily available fluorination reagents, one single chiral primary amine catalyst would be able to provide two enantiomers, respectively, bearing fluorinated quaternary centers with good enantioselectivity in both cases (Scheme 1, II).

**Scheme 1:**
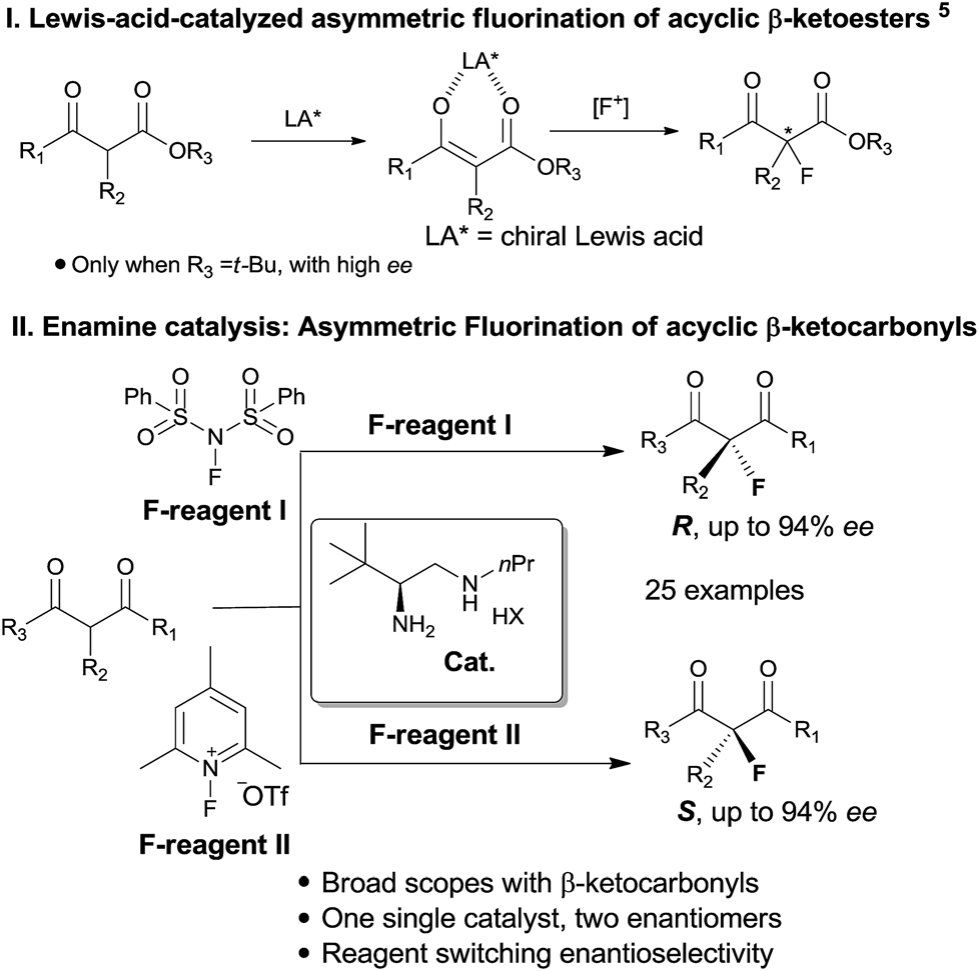
Asymmetric α-fluorination of cyclic and acyclic ketones.

Our initial investigation was performed with acetoacetate **1a** in the presence of our typical primary amine catalyst **I**/TfOH. A quick survey of different fluorination reagents was conducted. The reaction proceeded smoothly in all the tests and a switch of chiral induction was clearly noted among different fluorination reagents. While the reaction with NFSI (**2a**) gave 51% ee (Table 1, entry 1), the use of **2b** and **2c** led to –71% ee and –38% ee, respectively, with opposite chiral induction (Table 1, entries 2 and 3). A switch of chiral induction was generally observed with other primary–tertiary diamine catalysts such as **IV** and **V** (Table 1, entries 7–10). Though enantioselectivity varied in different solvents (see the ESI for details†), the switching phenomenon was uniformly observed (Table 1, entries 4–6 *vs.* 1–3). In all the cases examined, fluorination reagent **2b** showed better enantioselectivity than **2c**. The reagents **2a** and **2b** were then selected for subsequent optimization with the aim to find a generally applied enantio-switching catalytic system (see the ESI for details†). In this regard, we were delighted to identify a simple primary–secondary diamine **II** that gave good enantioselectivity in both of the switching reactions (Table 1, entries 11 and 12). Thus, the best reaction conditions are a combination of **II**/(DNBA I) (1 : 1) with **2a** as the fluorination reagent in chloroform at room temperature, providing the product with 86% yield and 92% ee, and the configuration of the product is *R*-configuration. On the other hand, when **2b** was used as the fluorination reagent, by the same primary amine **II**/(DNBA II) in methanol, the desired product was obtained with 95% yield and –90% ee, and the product was *S*-configuration. At this stage, alteration of the acidic additive from TfOH to dinitrobenzoic acid was found to give a small but noticeable improvement in enantioselectivity (Table 1, entries 13 and 14 *vs.* 11 and 12). An additional benefit of the use of dinitrobenzoic acid is the ease in handling and manipulation as the resulting salts are crystal solids and bench stable. Adamantyl primary amine **III**, a close analogue of **II**, was also found to deliver an excellent –93% ee for the *S*-selective process, however, this catalyst performed rather poorly in the *R*-selective process with only 72% ee (Table 1, entries 15 and 16). This observation indicated a critical balance of steric hindrance for both the two enantio-switching processes.

**Table 1:** Screening and optimization^*a*^ 
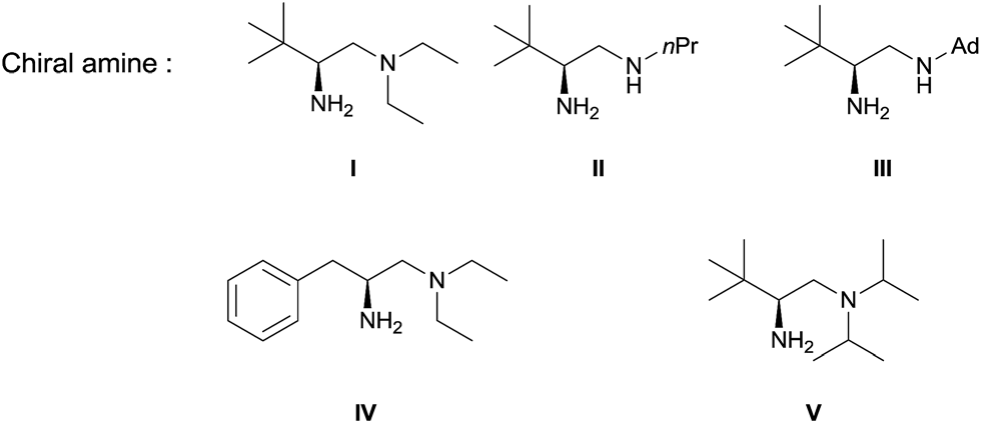

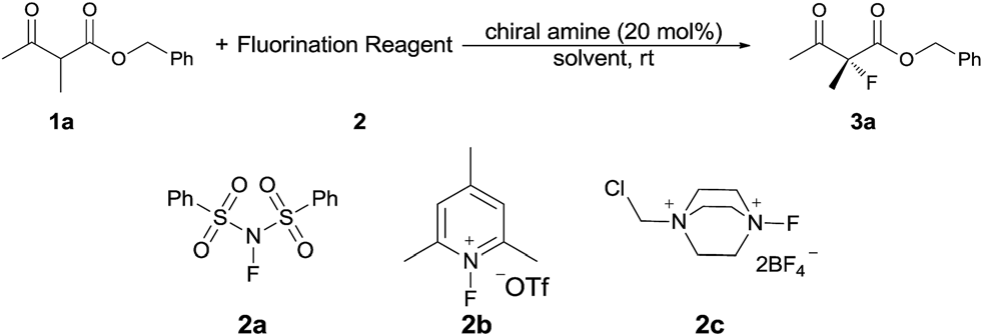					
Entry	Amine catalyst	Fluorination reagent	Solvent	Yield^*b*^ (%)	ee^*c*^ (%)
1	**I**/TfOH	**2a**	CHCl_3_	41	51
2	**I**/TfOH	**2b**	CHCl_3_	75	–71
3	**I**/TfOH	**2c**	CHCl_3_	71	–38
4	**I**/TfOH	**2a**	CH_3_OH	54	21
5	**I**/TfOH	**2b**	CH_3_OH	75	–83
6	**I**/HOTf	**2c**	CH_3_OH	94	–69
7	**IV**/TfOH	**2a**	CHCl_3_	50	42
8	**IV**/TfOH	**2b**	CH_3_OH	88	–60
9	**V**/TfOH	**2a**	CHCl_3_	45	33
10	**V**/TfOH	**2b**	CH_3_OH	75	–93
11	**II**/TfOH	**2a**	CHCl_3_	72	81
12	**II**/TfOH	**2b**	CH_3_OH	90	–89
**13** ^*d*^	**II**/**DNBA-I**	**2a**	**CHCl**_**3**_	**85**	**92**
**14** ^*d*^	**II**/**DNBA-II**	**2b**	**CH**_**3**_ **OH**	**95**	**–90**
15	**III**/TfOH	**2a**	CHCl_3_	42	72
16	**III**/TfOH	**2b**	CH_3_OH	82	–93

^*a*^General conditions: **1a** (0.075 mmol), **2** (0.05 mmol), amine catalyst (20 mol%) in solvent at r.t. for 24 h.

^*b*^Isolated yield.

^*c*^Determined by HPLC on a chiral stationary phase.

^*d*^DNBA-I: 2,4-(NO_2_)_2_PhCO_2_H; DNBA-II: 3,4-(NO_2_)_2_PhCO_2_H.

Under the optimized conditions, the substrate scope for the *R*-selective process was firstly investigated as above by using 20 mol% **II**/DNBA I as the catalyst, and **2a** as the fluorination reagent, in CHCl_3_. As shown in Table 2, a variety of β-keto esters, including benzyl, ethyl, isopropyl, allyl, *n*-butyl, cinnamyl, and naphthalen-1-ylmethyl ester all gave the desired products with excellent ee (89–93% ee) and good yields (up to 99%) (Table 2, entries 1–7). Alteration of the group R_2_, α-substituents on acetoacetates (**3h–3n**), was well tolerated. Increasing the bulkiness of the α-substituent as in **3g–3k** from methyl to iso-butyl led to a reduction in the reactivity, but excellent enantioselectivity was obtained in all these cases (Table 1, entries 8–11). It is noted that the reaction also tolerated α-allyl (**3l**), α-propargyl (**3m**) or α-benzyl groups (**3n**), affording the desired fluorination adducts in good yields and high enantioselectivity (Table 2, entries 12–14). 1,3-Diketone has been examined in the reaction, showing good reactivity but moderate ee (Table 2, entry 15), which is likely a result of uncontrolled enol-fluorination due to the predominant enol-form of 1,3-diketones. An ethyl ketone **3p** also worked in the reaction, showing low activity and moderate to good enantioselectivity (Table 2, entry 16). The observed low reactivity could be ascribed to its difficulty in forming enamine.^12^

**Table 2:** Substrate scope^*a*^ 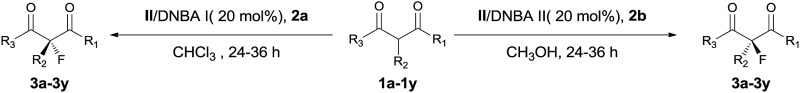

Entry^*b*^ ^,^ ^*c*^	*R* product	*S* product	Entry^*b*^ ^,^ ^*c*^	*R* product	*S* product
1–5	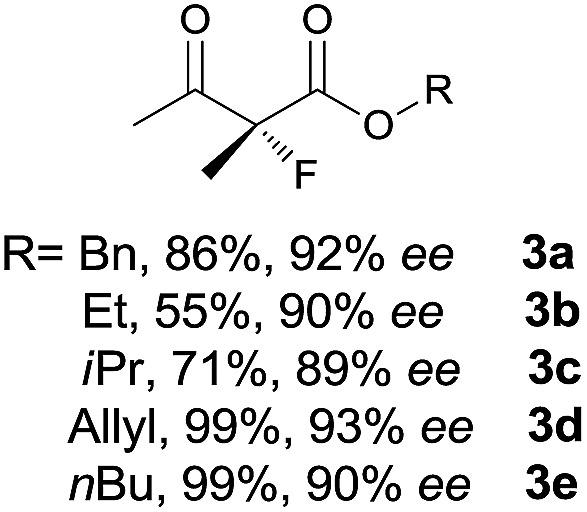	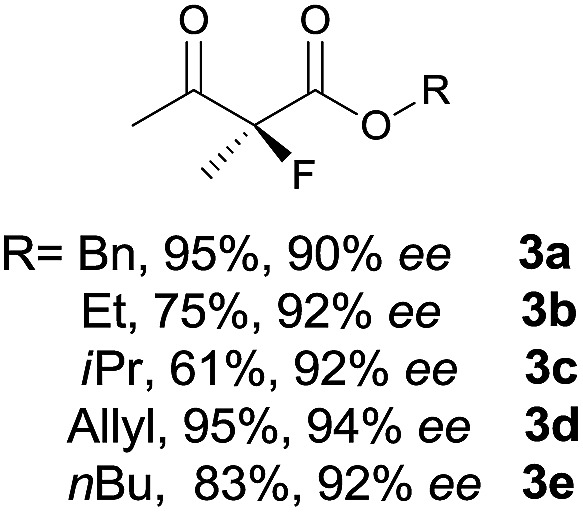	16	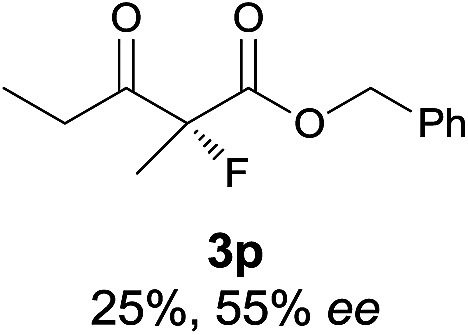	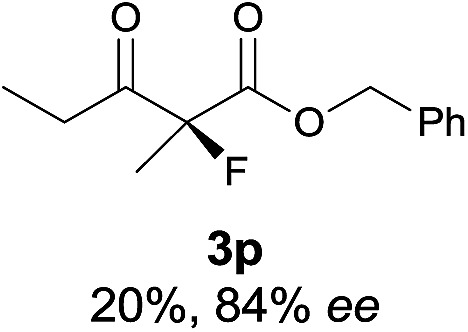
6	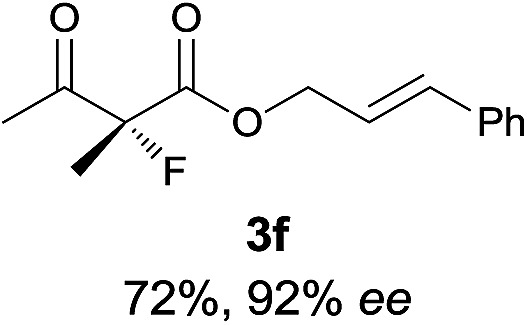	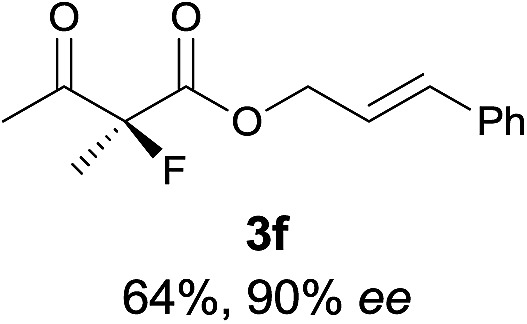	17	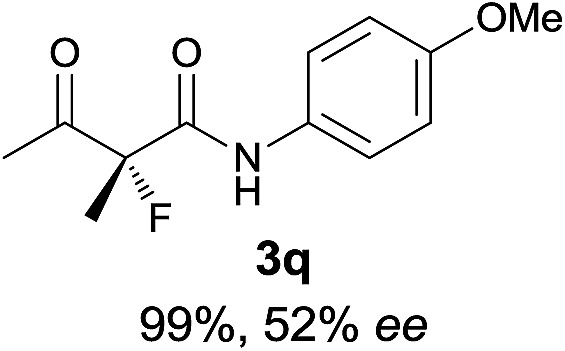	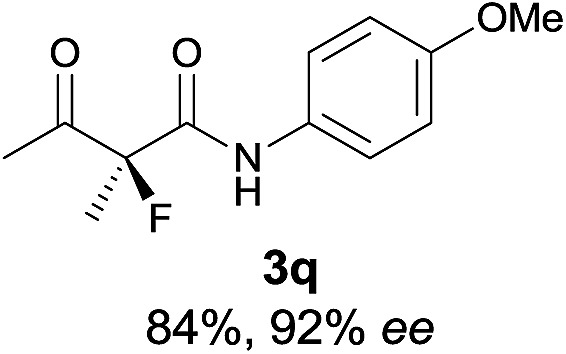
7	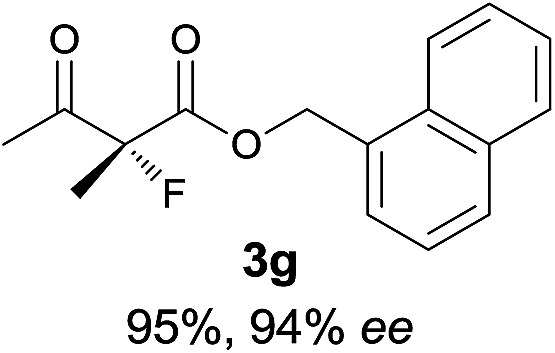	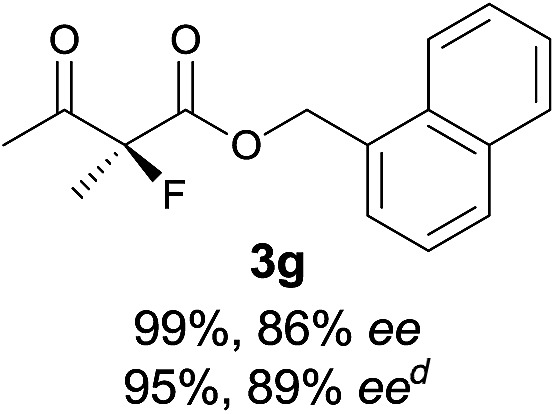	18	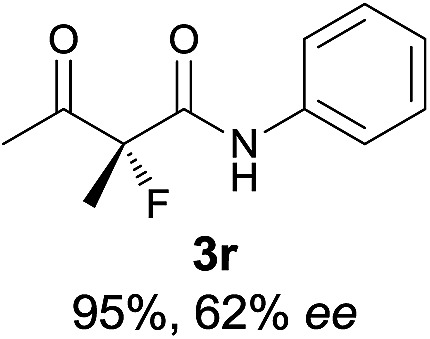	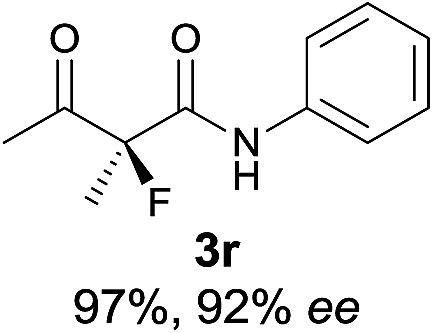
8	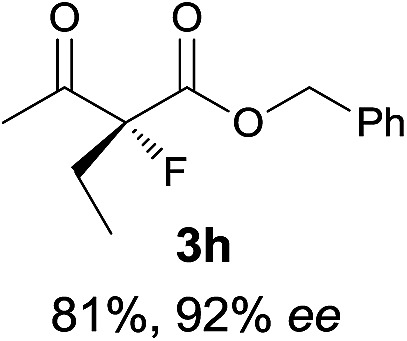	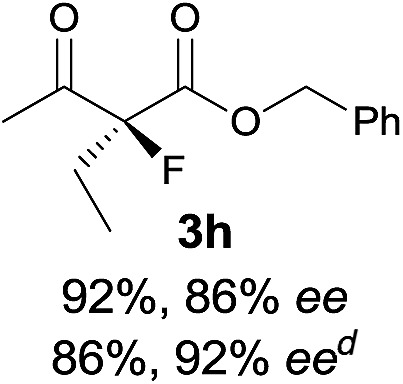	19	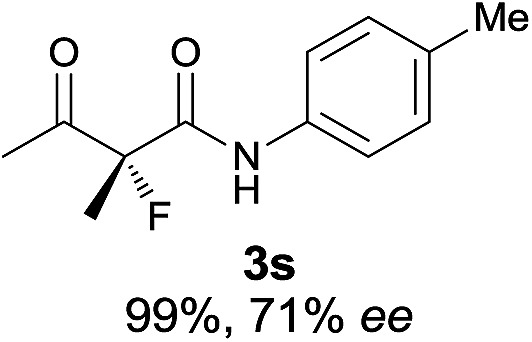	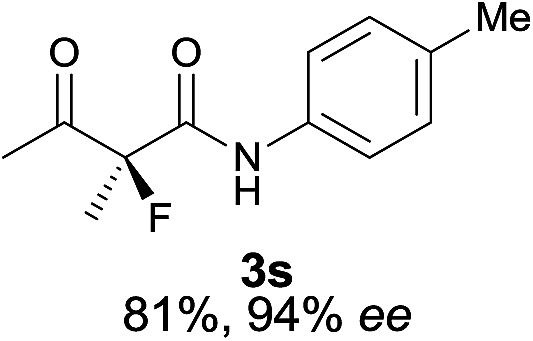
9	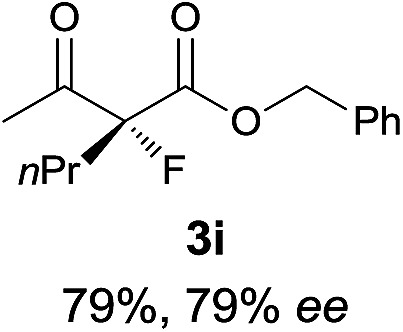	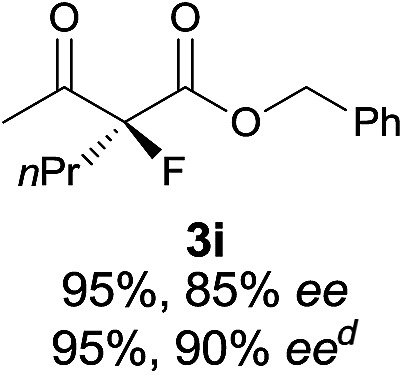	20	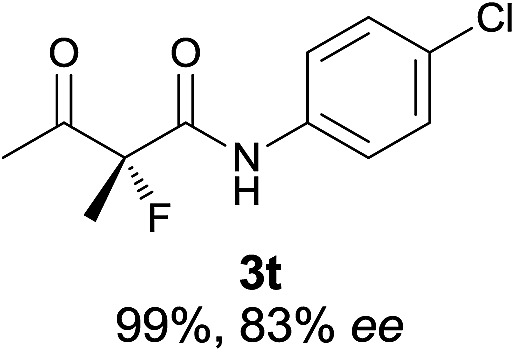	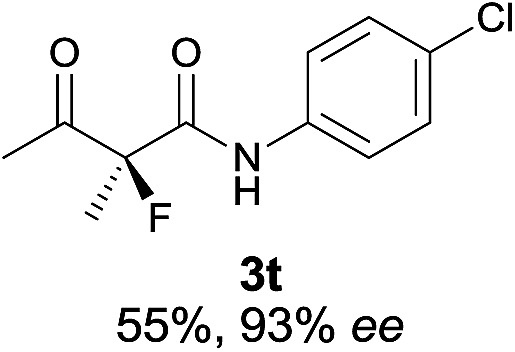
10	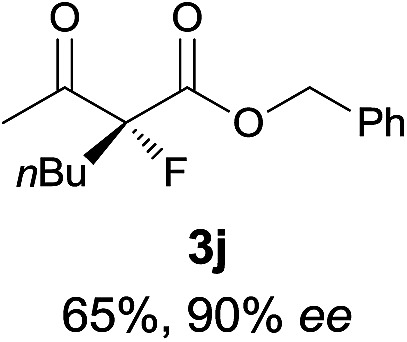	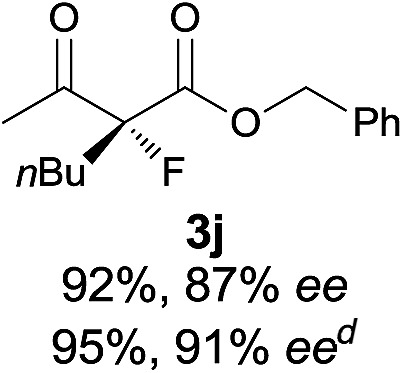	21	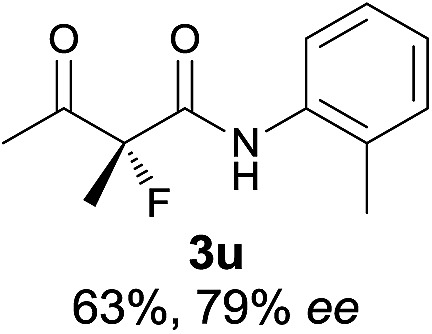	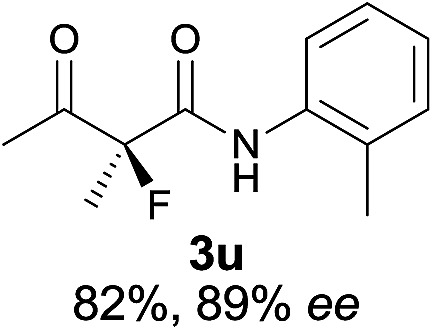
11	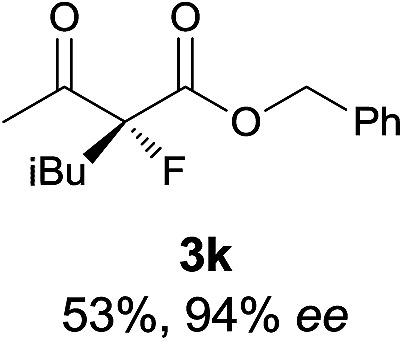	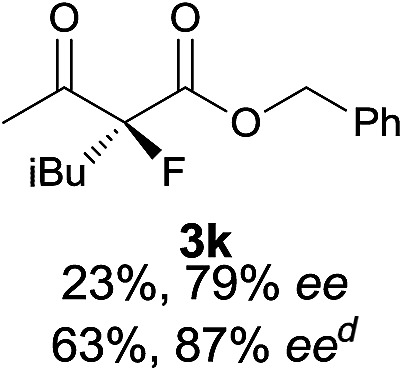	22	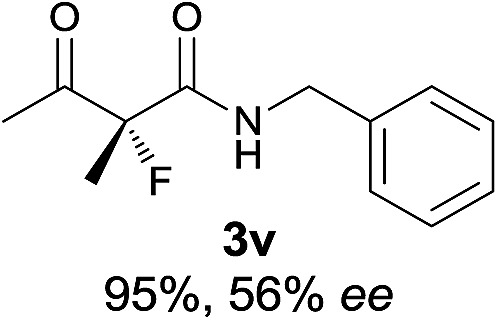	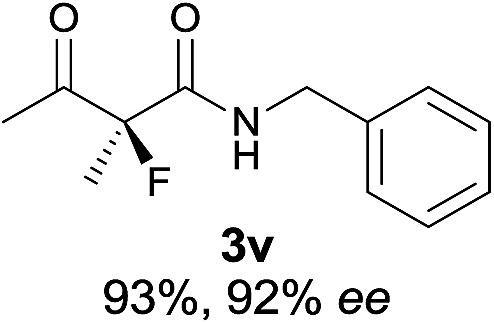
12	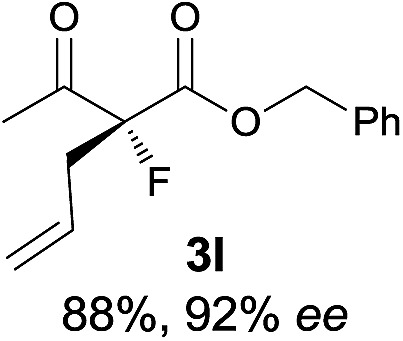	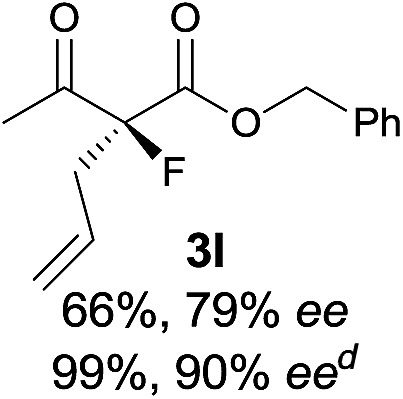	23	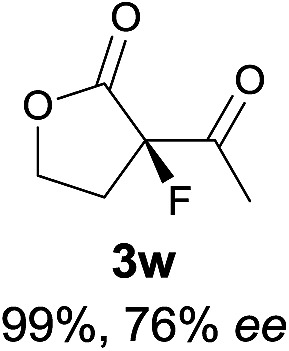	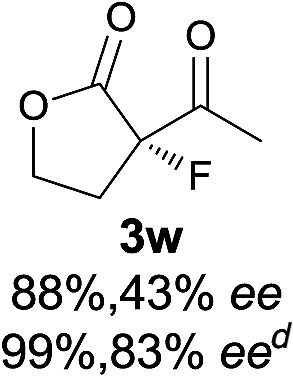
13	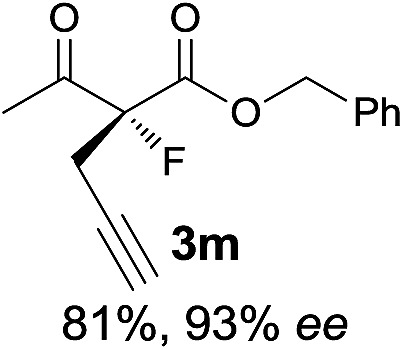	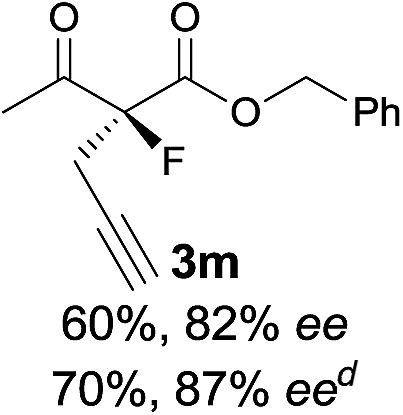	24	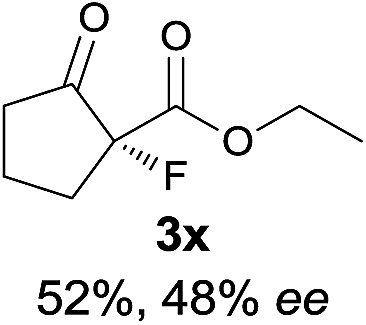	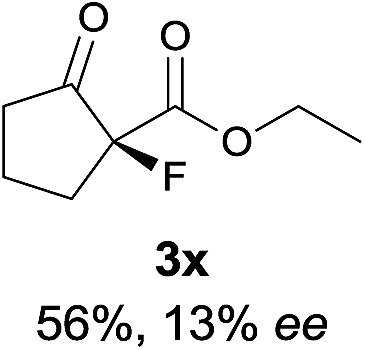
14	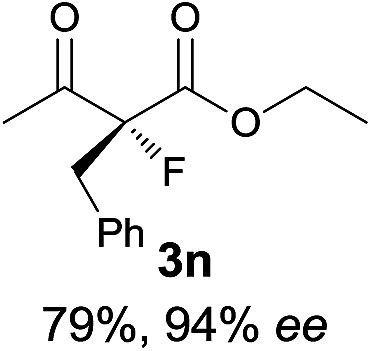	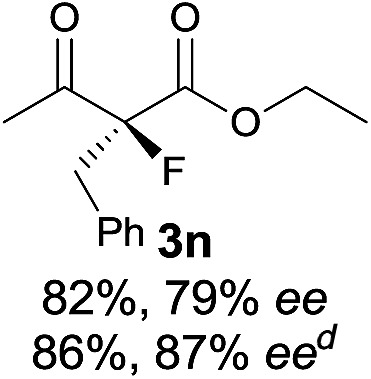	25	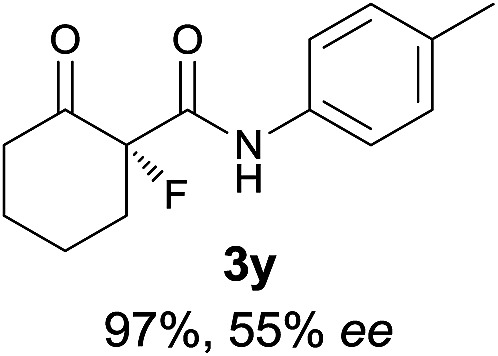	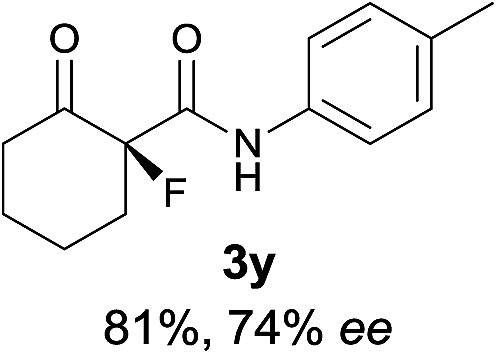
15	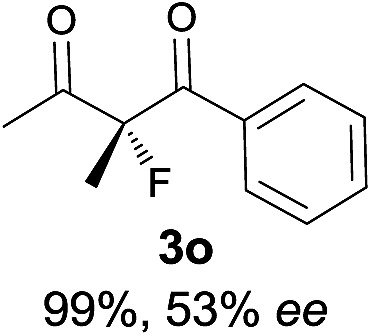	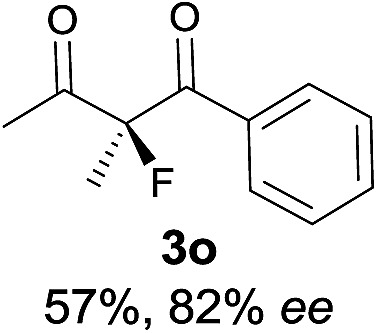			

^*a*^General conditions: **1** (0.075 mmol), **2a** (0.05 mmol), and **II**/DNBA-I (0.01 mmol, 20 mol%) in CHCl_3_ (0.25 mL) at r.t. for 24–36 h; **1** (0.075 mmol), **2b** (0.05 mmol), and **II**/DNBA-II (0.01 mmol, 20 mol%) in CH_3_OH (0.4 mL) at r.t. for 24–36 h.

^*b*^Yields shown are of isolated products.

^*c*^The ee was determined by GC or HPLC on a chiral stationary phase.

^*d*^Catalyst **III**/TfOH was used instead of **II**/DNBA-II at r.t. for 36 h.

The substrate scope could be further extended to β-ketoamides.^11^ Both *N*-aryl and *N*-aliphatic amides worked well in the reactions with good yields and moderate to good enantioselectivities (Table 2, entries 17–22 and 25). It seems that the free N–H moiety has a marginal effect on the reactions. In particular, *N*-aryl amides bearing either electron-donating or electron-withdrawing groups were equally applied, with the latter giving slightly better enantioselectivity (Table 2, entry 20 *vs.* 19). A lactone-type substrate **3w** could also be incorporated in the current catalysis with 76% ee and 99% yield.^6*c*^ Cyclic ketocarbonyls have also been examined, showing good reactivity but moderate enantioselectivity (Table 2, entries 24 and 25).

The same substrate scope was also tested for the *S*-selective reactions. In these cases, the reaction was examined with **2b** as the fluorination reagent, and 20 mol% **II**/DNBA-II or **III**/HOTf as the catalyst in CH_3_OH. In most cases, the reactivity and enantioselectivity were comparable to those obtained in the *R*-selective processes. One particular exception was with the reactions of β-ketoamides, wherein the *S*-selective process with primary amine catalyst **II** worked extremely well to afford the desired adducts in good yields and high enantioselectivities (Table 2, entries 17–22 and 25). In comparison, the enantioselectivity with the *R*-process was moderate. The use of primary amine catalyst **III**/TfOH has been found to deliver improved enantioselectivity in the *S*-selective reactions (Table 2, entries 7–14 and 23).

To probe the utility of our fluorination reaction in preparative synthesis, a gram-scale reaction of β-ketoester **1a** (7.5 mmol, 1.545 g) was performed with NFSI (5 mmol, 1.576 g) as the fluorination reagent in CHCl_3_ for 48 h delivering the desired product **3a** with a good yield (0.952 g, 85% yield) and excellent enantioselectivity (94% ee). The chiral primary amine catalyst could be quantitatively recovered by concentrating the aqueous solution after work up.

Though an enol-type could not be completely ruled out, the current experimental observations as well as our previous studies strongly favour an enamine mechanism (see the ESI for details†). To account for the switch of enantioselectivity, we have proposed two plausible enamine-based intermolecular F-attack transition states.^13^ For the NFSI (**2a**) based *R*-selective process, an H-bonding mode I between the sulfonyl moiety and the protonated ammonium N–H was invoked in the stereocontrol. In this model, the *Si*-facial fluorination was largely disfavoured due to geometrically unfavourable H-bonding with the ammonium N–H. On the other hand, an electrostatic mode II was proposed for the *N*-fluoro-pyridinium (**2b**) based *S*-selective process, wherein the electrostatic repulsion between the cationic charged ammonium and pyridinium species plays a dominant role. Steric effects would also contribute in this model, however, the impact should be minor, as we note that primary amine **II** performed equally as well as its more bulky counterparts such as catalysts **III** and **V**. The observed solvent effect (*e.g.* CH_3_OH *vs.* CHCl_3_, Table 1, entries 1–6) is also in line with the electrostatic model since ionic species would become highly dissociated in polar protic CH_3_OH, a feature favourable for the electrostatic repulsion interaction.

The stereocontrolling modes (I and II) could be further verified by DFT calculations at the B3LYP/6-31G* level of approximation (see the ESI for details†). For mode II, the *S*-selective TS-*S* was favoured over the *R*-selective TS-*R* by 3.0 kcal mol^–1^, which is consistent with the experimental observations. We further calculated the electrostatic surface potential (ESP) of the two located TSs. As revealed in Fig. 1b, the ammonium and pyridinium moieties of TS-*R*, both bearing positively charged surfaces, seem not to have any noticeable steric interaction from being close together. Thus, TS-*R* would be mainly disfavoured by electrostatic repulsion not by steric effects. By AIM analysis, we could also identify an attractive C–H···F interaction between the *tert*-butyl group of the amine catalyst and the fluorination reagent **2b**, as shown in Fig. 1b, which may also contribute in facilitating the *Si*-facial attack in TS-*S*.^14^

**Fig. 1:**
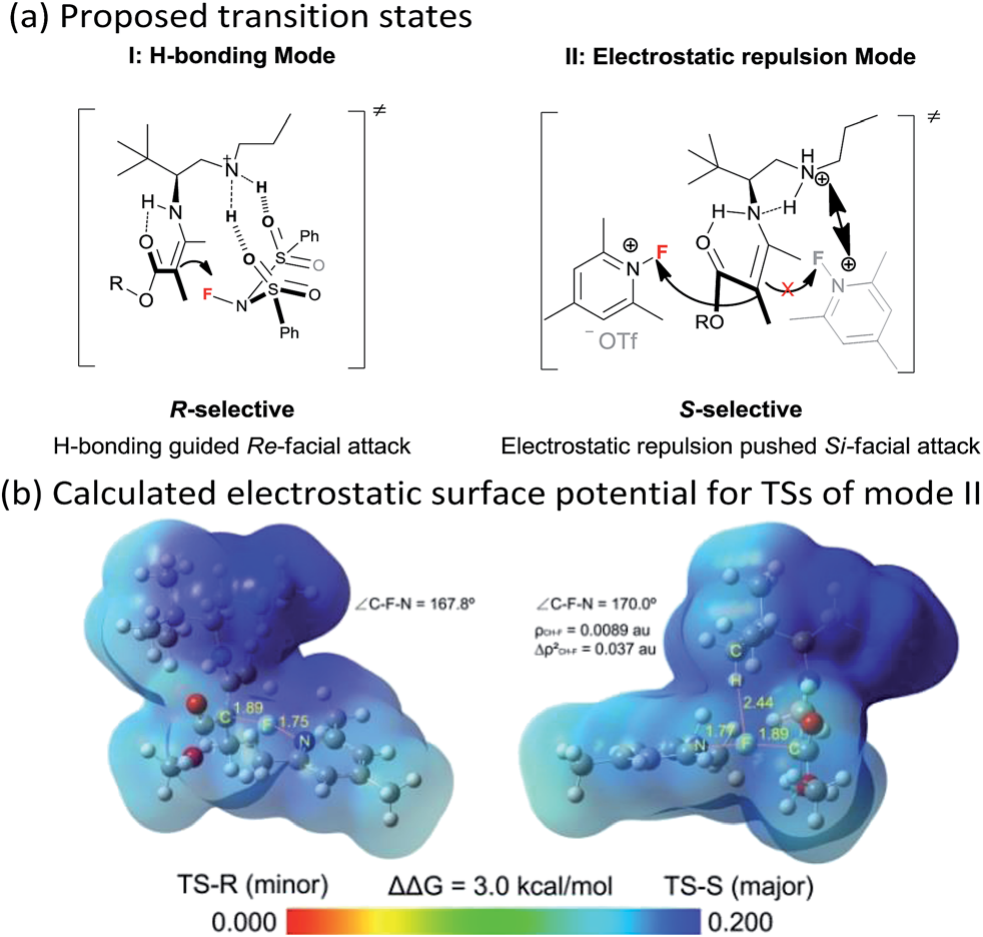
(a) Proposed transition states (I and II) for the two enantioselectivity switch fluorination reactions; (b) calculated electrostatic surface potential for TSs of the electrostatic mode II.

## Conclusions

In summary, we have presented herein a reagent-controlled enantioselectivity switch for the organocatalytic asymmetric fluorination of β-ketocarbonyls. A simple swap of the fluorination reagent switched the enantioselectivity with good reactivity and enantiomeric excess in both cases. Mechanistic studies revealed dual H-bonding and electrostatic stereocontrolling modes for a single chiral primary amine catalyst. Further explorations of switchable enantioselectivity in other reactions are currently underway in our laboratory.

## References

[cit1] (b) Comprehensive Asymmetric Catalysis, ed. E. N. Jacobsen, A. Pfaltz and H. Yamamoto, Springer, Berlin, 1999.

[cit2] Wang J., Feringa B. L. (2011). Science.

[cit3] Sohtome Y., Tanaka S., Takada K., Yamaguchi T., Nagasawa K. (2010). Angew. Chem., Int. Ed..

[cit4] Champagne P. A., Desroches J., Hamel J.-D., Vandamme M., Paquin J.-F. (2015). Chem. Rev..

[cit5] Hintermann L., Togni A. (2000). Angew. Chem., Int. Ed..

[cit6] Kim D. Y., Park E. J. (2002). Org. Lett..

[cit7] Marigo M., Fielenbach D., Braunton A., Kjærsgaard A., Jørgensen K. A. (2005). Angew. Chem., Int. Ed..

[cit8] Brandes S., Niess B., Bella M., Prieto A., Overgaard J., Jørgensen K. A. (2006). Chem.–Eur. J..

[cit9] Kwiatkowski P., Beeson T. D., Conrad J. C., MacMillan D. W. C. (2011). J. Am. Chem. Soc..

[cit10] Yang X., Phipps R. J., Toste F. D. (2014). J. Am. Chem. Soc..

[cit11] Zheng L.-S., Wei Y.-L., Jiang K.-Z., Deng Y., Zheng Z.-J., Xu L.-W. (2014). Adv. Synth. Catal..

[cit12] Zhang L., Fu N., Luo S. (2015). Acc. Chem. Res..

[cit13] The intramolecular F-attack with the secondary NH–F species (see ref. 9*b*), *in situ* transferred from the fluorination reagent to the aminocatalyst, though not completely excluded, may not be applicable in our case. First, there is no such precedence on fluorination with NH–F species. In addition, even if an F-transfer did occur, facile proton release instead of F-attackmight be the dominant pathway, leading to an unreactive neutral-N–F species or even poisoning of the catalyst. Experimentally, we did not detect such species by *in situ* ESI-MS in both cases

[cit14] In TS-*S*, a short C–H···F distance was found of 2.44 Å. Atoms in Molecules (AIM) analysis at the bond critical point suggested a weak interaction between H and F where the electron density (*ρ*) is 0.0089 au and the Laplacian value (Δ*ρ* ^2^) is 0.037 au. These values indicate that a weak C–H···F interaction is present. For a review on C–H···F–C interaction, see: Liu C.-C. Chan M. C. W., Acc. Chem. Res., 2015, 48 , 1580 .25993345

