# Supplementary material for: Reagent-controlled enantioselectivity switch for the asymmetric fluorination of β-ketocarbonyls by chiral primary amine catalysis
Source: Chem Sci. 2016 Aug 26;8(1):621–6. doi: 10.1039/c6sc03109a (PMC5358536; doi:10.1039/c6sc03109a)
Supplement: Supplementary file 1 [file SC-008-C6SC03109A-s001.pdf]

Electronic Supplementary Material (ESI) for Chemical Science.
This journal is © The Royal Society of Chemistry 2016

Reagent-Controlled Enantioselectivity Switch in Asymmetric Fluorination of β-Ketocarbonyls by Chiral Primary Amine Catalysis

*Yang'en You, Long Zhang and Sanzhong Luo**

Supporting Information

Table of Contents

General information and materials..S2

Experimental section…………………………………………………………......S3

Mechanism studies…………………………………………………………….......S15

Calculated Transition States ……………………………………………..…...S17

NMR spectra………………………………………………………………..…..S28

HPLC charts……………………………………………………………………….S78

General information: Commercial reagents were purified prior to use following the guidelines of Perrin and Armarego.[1] All solvents were purified according to the method of Grubbs. Nuclear magnetic resonance (NMR) spectra were recorded using Bruker AV-400, AV-500 and AV-600 spectrometers. ^{1}H, ^{13}C and ^{19}F NMR spectra were measured on a NMR instrument (400 and 500 MHz for ^{1}H NMR, 100 and 125 MHz for ^{13}C NMR, 471 and 565 MHz for ^{19}F NMR). Tetramethylsilane (TMS) served as the internal standard for ^{1}H NMR, and $CDCl_3$ served as the internal standard for ^{13}C NMR. The enantiomeric excesses were determined by HPLC analysis on Chiral Daicel Chiralpak OD-H, AD-H, AS-H and OJ-H columns or chiral stationary phases (CP-Chirasil-Dex CB: 25 m · 0.25 mm). Optical rotation were measured on a commercial polarimeter and reported as follows: $[\alpha]_D^{22}$ (c = g/100 mL, solvent). HRMS was recorded on a commercial instrument (ESI and APCI Source).

Materials: The corresponding β-Ketoesters **1a-1n** and **1o** were prepared by alkylation of the corresponding α-unsubstituted β-ketoesters with alkyl iodide.[1] Cyclic β-ketoesters **1p** were from commercial Alfa-Aesar. β-ketoamides **1q-1v** were prepared according to literature precedent.[4,5] **2a**, **2b** and **2c** were purchased from commercial suppliers and used without further purification.

Experimental section:

A）Optimization of reaction conditions

a) Initial solvent screening

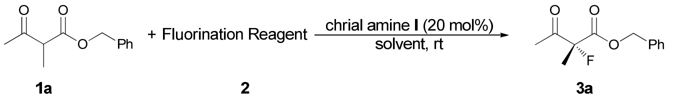

Entry	Flourination reagent	Solvent	Yield[b]	*ee*[c]
1	**2a**	$CHCl_3$	41%	51%
2	**2a**	MeOH	54%	21%
3	**2a**	MeCN	36%	11%
4	**2a**	THF	36%	33%
5	**2a**	Et_2O	90%	12%
6	**2c**	$CHCl_3$	71%	-38%
7	**2c**	MeOH	94%	-69%
8	**2c**	MeCN	10%	-6%
9	**2c**	Et_2O	85%	-58%
10	**2c**	CH_2Cl_2	81%	-15%
11	**2c**	1,4-dioxane	67%	-51%
12	**2b**	$CHCl_3$	75%	-71%
13	**2b**	MeOH	75%	-83%

[a] General conditions: **1a** (0.075mmol), **2** (0.05 mmol), **I**/TfOH(20 mol%) in solvent (0.25 mL) at rt for 24 h. [b] Isolated yield. [c] Determined by HPLC on a chiral stationary phase.

Chiral amine :

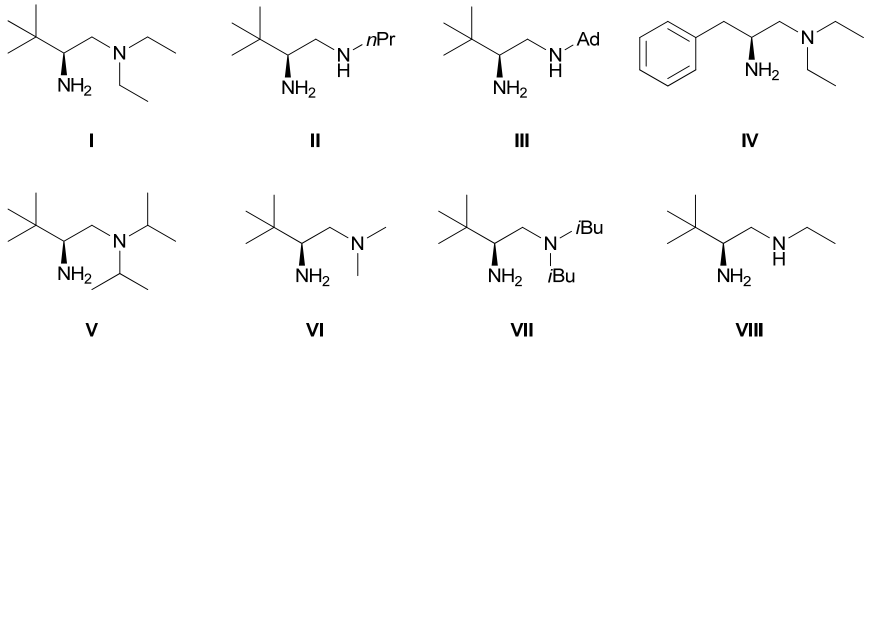

b) Screening of primary amine

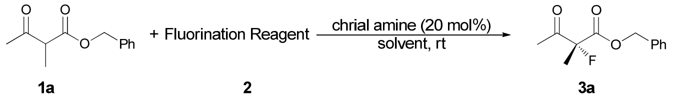

Entry	Flourination reagent	Catalyst	Yield[b]	*ee*[c]
1	**2a**	**I**/TfOH	58%	54%
2	**2a**	**II**/TfOH	72%	81%
**3**	**2a**	**II/DNBA I**	**85%**	**92%**
4	**2a**	**II**/DNBA II	80%	90%
5	**2a**	**III**/TfOH	42%	72%
6	**2a**	**IV**/TfOH	50%	42%
7	**2a**	**V**/TfOH	45%	33%
8	**2a**	**VI**/TfOH	55%	65%
9	**2a**	**VII**/TfOH	45%	38%
10	**2a**	**VIII**/TfOH	71%	83%
11	**2a**	**VIII**/DNBA I	81%	90%
12	**2a**	**VIII**/DNBA II	82%	91%
13	**2b**	**I**/TfOH	75%	-83%
14	**2b**	**II**/TfOH	90%	-89%
15	**2b**	**II**/DNBA I	90%	-88%
**16**	**2b**	**II/DNBA II**	**95%**	**-90%**
17[d]	**2b**	**II**/DNBA II	49%	-89%
18	**2b**	**III**/DNBA II	18%	Rac.
**19**	**2b**	**III/TfOH**	**82%**	**-93%**
20[d]	**2b**	**III**/TfOH	71%	-92%
21	**2b**	**IV**/TfOH	88%	-60%
22	**2b**	**V**/TfOH	75%	-93%
23	**2b**	**VIII**/TfOH	91%	-88%

[a] General conditions: **1a** (0.075mmol), **2** (0.05 mmol), amine catalyst(20 mol%) in solvent (0.25 mL) at rt for 24 h, DNBA I: 2,4-$(NO_2)_2PhCO_2H$; DNBA II: 3,4-$(NO_2)_2PhCO_2H$. [b] Isolated yield. [c] Determined by HPLC on a chiral stationary phase. [d] 10 mol% catalyst was used.

c) Screening of acidic additive

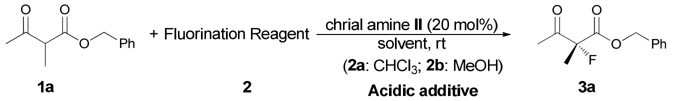

Entry	Acid	Fluorination reagent	Yield[b]	*ee*[c]
1	$PhCO_2H$	**2a**	65%	86%
2	m-NO_2PhCO_2H	**2a**	66%	88%
**3**	**DNBA-I**	**2a**	**90%**	**91%**
4	DNBA-II	**2a**	84%	90%
5	TFA	**2a**	80%	87%
6	TfOH	**2a**	75%	81%
7	Boc- L-*tert*-Leucine	**2a**	76%	89%
8	PhCOOH	**2b**	81%	-89%

9	DNBA-I	**2b**	89%	-87%
10	DNBA-II	**2b**	90%	-90%
**11[d]**	**DNBA-II**	**2b**	**98%**	**-90%**
12	TFA	**2b**	76%	-86%
13	TfOH	**2b**	90%	-89%

[a] General conditions: **1a** (0.075mmol), **2** (0.05 mmol), **II/acidic additive** (20 mol%) in solvent (0.25 mL) at rt for 24 h. [b]NMR yield. [c] Determined by HPLC on a chiral stationary phase. [d]0.4 ml MeOH was been used.

d) Further screening of solvents using primary amine II.

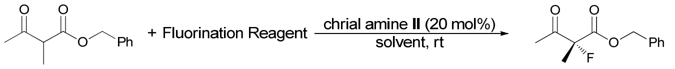

1a **2** **3a**

Entry	Flourination reagent	Solvent	Yield[b]	*ee*[c]
**1**	**2a**	**$CHCl_3$**	**91%**	**92%**
2	**2a**	MeOH	80%	47%
3	**2a**	MeCN	83%	59%
4	**2a**	THF	57%	62%
5	**2a**	Et_2O	70%	59%
6	**2a**	CH_2Cl_2	76%	87%
7	**2b**	$CHCl_3$	40%	-48%
**8**	**2b**	**MeOH**	**90%**	**-90%**
9	**2b**	THF	41%	-41%
10	**2b**	MeCN	76%	-88%
11	**2b**	Et_2O	70%	-77%
12	**2b**	CH_2Cl_2	45%	-63%
13	**2b**	H_2O	51%	-70%

[a] General conditions: **1a** (0.075mmol), **2** (0.05 mmol), **II**/DNBA I or **II**/DNBA II(20 mol%) in solvent (0.25 mL) at rt for 24 h. [b]NMR yield. [c] Determined by HPLC on a chiral stationary phase.

B) General procedure for fluorination reaction

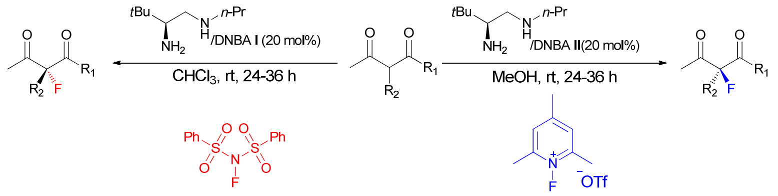

For reaction with 2a: To a flame-dried tube equipped with a magnetic stir bar was added benzyl 2-methyl-3-oxobutanoate (**1**, 0.075 mmol), primary amine (**II**/DNBA I, 20 mol%), the mixture was diluted with 0.1 mL of anhydrous $CHCl_3$. The mixture stirred 10 min, and then NFSI (**2a**, 0.05 mmol) and 0.15 ml $CHCl_3$ was added. The reaction was conducted at rt for 24 h, the solvent was removed and the residue was purified by silica gel chromatography (10% EtOAc in Petroleum ether) to give **3** as a yellow oil. The enantiomeric excess was determined by HPLC (OJ-H).

The reaction was performed in gram scale under the same procedure.

For reaction with 2b: To a flame-dried tube equipped with a magnetic stir bar was added benzyl 2-methyl-3-oxobutanoate (**1**, 0.075 mmol), primary amine (**II**/DNBA II, 20 mol%), **2b** (0.05 mmol), the mixture was diluted with 0.4 mL of anhydrous MeOH. The reaction was conducted at rt for 24 h, the solvent was removed and the residue was purified by silica gel chromatography (10% EtOAc in Petroleum ether) to give **3** as a yellow oil. The enantiomeric excess was determined by HPLC (OJ-H).

As for the issue of SDE[6] about fluorination containing compounds, compound *R*-**3a** was selected as a model substrate to carry out SDE test. The achiral chromatography test gave a negative result, confirming that the isolation by chromatography does not alter the original enantiomeric composition.

Characterization data for new compounds:

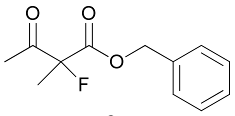

3a

Pale yellow oil; (*R*)-**3a**: 86% yield; (*S*)-**3a**: 95% yield; IR (thin film, cm^{-1}) 3347, 2971, 2931, 2884, 1738, 1669, 1467, 1379, 1311, 1162, 1130, 953, 817; ^{1}H NMR (400 MHz, $CDCl_3$) δ 7.46 – 7.28 (m, 5H), 5.24 (s, 2H), 2.28 (d, *J* = 4.5 Hz, 3H), 1.69 (d, *J* = 22.1 Hz, 3H) ppm; ^{13}C NMR (101 MHz, $CDCl_3$) δ 202.37, 202.09, 166.98, 166.73, 134.78, 128.82, 128.80, 128.30, 98.74, 96.81, 68.10, 25.09, 20.03, 19.81 ppm; ^{19}F NMR (471 MHz, $CDCl_3$) δ -157.00; HRMS (ESI) calcd for $C_{12}H_{12}FO_3^-$: 223.0776, found 223.0774; HPLC analysis: Daicel Chiralpak OJ-H, flow rate =1.0 mL/min, λ= 207 nm, hexane/iso-propanol = 96:4, (*R*)-**3a**: 92% *ee*; $[\alpha]_D^{22}$ = -9.9 (c = 1.0, $CHCl_3$), retention time: 17.2 min (major) and 19.5 min (minor); (*S*)-**3a**: 90% *ee*; $[\alpha]_D^{22}$ = 17.6 (c = 0.50, $CHCl_3$), retention time: 17.1 min (minor) and 19.0 min (major).

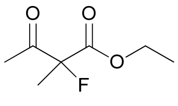

3b

Colorless oil[3]; (*R*)-**3b**: 55% yield; (*S*)-**3b**: 75% yield; IR (thin film, cm^{-1}) 2921, 1731, 1668, 1219; IR (thin film, cm^{-1}) 2921, 1731, 1668, 1219; ^{1}H NMR (400 MHz, $CDCl_3$) δ 4.25 (q, *J* = 7.1 Hz, 2H), 2.30 (d, *J* = 4.5 Hz, 3H), 1.66 (d, *J* = 22.1 Hz, 3H), 1.28 (t, *J* = 7.1 Hz, 3H) ppm; ^{13}C NMR (101 MHz, $CDCl_3$) δ 202.55, 202.27, 167.11, 66.86, 98.70, 96.78, 62.70, 25.05, 19.99, 19.77, 14.05 ppm; ^{19}F NMR (565 MHz, $CDCl_3$) δ -157.04 (s) ppm; GC analysis: CP-Chirasil-Dex CB, isotherm 40 °C, N_2, (*R*)-**3b**: 90% *ee*; $[\alpha]_D^{22}$ = -30.0 (c = 0.27, $CHCl_3$), retention time: 12.0 min (major) and 15.0 min (minor); (*S*)-**3b**: 92% *ee*; $[\alpha]_D^{22}$ = 49.5 (c = 0.20, $CHCl_3$), retention time: 12.6 min (minor) and 14.2 min (major). The spectroscopic data for **3b** matched those described in the literature[2]; CAS NO.: 122795-13-5; For the *S* enantiomer $[\alpha]_D$ = 46.3 (c = 0.80 in $CHCl_3$) is reported in the literature[3].

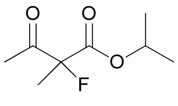

3c

Colorless oil; (*R*)-**3c**: 71% yield; (*S*)-**3c**: 61% yield; IR (thin film, cm^{-1}) 2921, 1731, 1668, 1219, 772; ^{1}H NMR (400 MHz, $CDCl_3$) δ 5.18 – 4.97 (m, 1H), 2.47 – 2.20 (m, 3H), 1.66 (dd, *J* = 22.1, 3.1 Hz,

3H), 1.27 (dd, J = 3.6, 2.4 Hz, 6H) ppm; ^{13}C NMR (101 MHz, $CDCl_3$) δ 202.54, 202.25, 166.46, 98.70, 96.78, 70.75, 25.07, 21.60, 19.89, 19.66 ppm; ^{19}F NMR (565 MHz, $CDCl_3$) δ -157.04 (s) ppm; HRMS (ESI) calcd for $C_8H_{14}FO_3^+$: 177.0921, found 177.0915; GC analysis: CP-Chirasil-Dex CB, isotherm 40 °C, N_2, (*R*)-**3c**: 89% *ee*; $[\alpha]_D^{22}$ = -52.0 (c = 0.15, $CHCl_3$), retention time: 17.9 min (major) and 19.1 min (minor); (*S*)-**3c**: 92% *ee*; $[\alpha]_D^{22}$ = 10.8 (c = 0.40, $CHCl_3$), retention time: 18.2 min (minor) and 19.0 min (major).

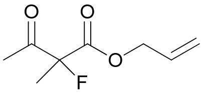

3d

Colorless oil; (*R*)-**3d**: 99% yield; (*S*)-**3d**: 95% yield; IR (thin film, cm^{-1}) 3840, 3735, 3567, 2920, 2850, 1730, 1669; ^{1}H NMR (400 MHz, $CDCl_3$) δ 5.90 (m, 1H), 5.32 (dd, J = 22.8, 13.8 Hz, 2H), 4.70 (d, J = 5.7 Hz, 2H), 2.33 (d, J = 4.6 Hz, 3H), 1.70 (d, J = 22.1 Hz, 3H) ppm; ^{13}C NMR (101 MHz, $CDCl_3$) δ 202.54, 202.26, 166.82, 166.57, 130.89, 119.67, 98.77, 96.84, 66.95, 25.15, 20.11, 19.88 ppm; ^{19}F NMR (565 MHz, $CDCl_3$) δ -156.93 (s) ppm; HRMS (ESI) calcd for $C_8H_{12}FO_3^+$:175.0765, found 175.0759; GC analysis: CP-Chirasil-Dex CB, isotherm 80 °C, N_2, (*R*)-**3d**: 93% *ee*; $[\alpha]_D^{22}$ = -40.5 (c = 0.20, $CHCl_3$), retention time: 15.6 min (major) and 16.3 min (minor); (*S*)-**3d**: 94% *ee*; $[\alpha]_D^{22}$ = 17.7 (c = 0.57, $CHCl_3$), retention time: 15.7 min (minor) and 16.2 min (major).

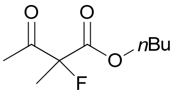

3e

Colorless oil; (*R*)-**3e**: 99% yield; (*S*)-**3e**: 83% yield; IR (thin film, cm^{-1}) 2961, 2922, 2851, 1757, 1737, 1359, 1281, 1141, 1108; ^{1}H NMR (400 MHz, $CDCl_3$) δ 4.22 (d, J = 6.5 Hz, 2H), 2.32 (d, J = 4.5 Hz, 3H), 1.71 (s, 2H), 1.68 – 1.59 (m, 3H), 1.37 (dd, J = 15.0, 7.5 Hz, 2H), 0.93 (t, J = 7.4 Hz, 3H) ppm; ^{13}C NMR (101 MHz, CDCl3) δ 202.65, 202.37, 167.21, 166.96, 98.77, 96.85, 66.53, 30.50, 25.12, 20.05, 19.82, 19.06, 13.73 ppm; ^{19}F NMR (565 MHz, $CDCl_3$) δ -164.45 (s) ppm; HRMS (ESI) calcd for $C_9H_{16}FO_3^+$:191.1078, found 191.1071; GC analysis: CP-Chirasil-Dex CB, isotherm 70 °C, N_2, (*R*)-**3e**: 90% *ee*; $[\alpha]_D^{22}$ = -31.1 (c = 0.55, $CHCl_3$), retention time: 15.9 min (major) and 16.8 min (minor); (*S*)-**3e**: 92% *ee*; $[\alpha]_D^{22}$ = 17.7 (c = 0.57, $CHCl_3$), retention time: 16.1 min (minor) and 16.6min (major).

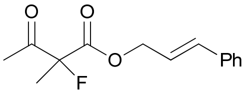

3f

Pale yellow oil; (*R*)-**3f**: 72% yield; (*S*)-**3f**: 64% yield; IR (thin film, cm^{-1}) 3840, 3734,3566, 2920, 1755, 1731, 1220, 772; ^{1}H NMR (400 MHz, $CDCl_3$) δ 7.39 (d, J = 7.8 Hz, 2H), 7.33 (t, J = 7.4 Hz, 2H), 7.29 (d, J = 7.2 Hz, 1H), 6.68 (d, J = 15.8 Hz, 1H), 6.37 – 6.13 (m, 1H), 4.86 (d, J = 6.6 Hz, 2H), 2.34 (d, J = 4.6 Hz, 3H), 1.71 (d, J = 22.1 Hz, 3H) ppm; ^{13}C NMR (101 MHz, $CDCl_3$) δ 202.51, 202.23, 166.96, 166.70, 135.92, 135.78, 128.79, 128.53, 126.88, 121.64, 98.79, 96.86, 77.48, 77.16, 76.84, 67.09, 25.16, 20.13, 19.90 ppm; ^{19}F NMR (565 MHz, $CDCl_3$) δ -156.87 (s) ppm; HRMS (ESI) calcd for $C_{14}H_{16}FO_3^+$: 251.1078, found 251.1074; HPLC analysis: Daicel Chiralpak AS-H, flow rate =1.0 mL/min, λ= 254 nm, hexane/iso-propanol = 95:5, (*R*)-**3f**: 92% *ee*; $[\alpha]_D^{22}$ = -32.7 (c = 0.30, $CHCl_3$), retention time: 7.6 min (minor) and 8.9 min (major). (*S*)-**3f**: 90% *ee*; $[\alpha]_D^{22}$ = 17.2 (c = 0.25, $CHCl_3$),

retention time: 7.7 min (major) and 9.0 min (minor).

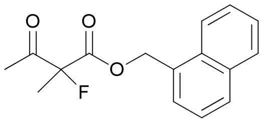

3g

Pale yellow oil; (*R*)-**3g**: 95% yield; (*S*)- **3g**: 99% yield; IR (thin film, cm^{-1}) 3567, 1750, 1732, 1278, 1220, 1136, 772; ^{1}H NMR (400 MHz, $CDCl_3$) δ 7.95 (d, J = 8.2 Hz, 1H), 7.88 (t, J = 7.8 Hz, 2H), 7.62 – 7.49 (m, 3H), 7.49 – 7.39 (m, 1H), 5.79 – 5.56 (m, 2H), 2.23 (d, J = 4.5 Hz, 3H), 1.68 (d, J = 22.1 Hz, 3H) ppm; ^{13}C NMR (101 MHz, $CDCl_3$) δ 202.39, 202.11, 167.03, 166.77, 133.82, 131.54, 130.18, 129.90, 128.91, 127.93, 126.91, 126.21, 125.33, 123.31, 98.74, 96.81, 66.61, 25.03, 20.01, 19.78 ppm; ^{19}F NMR (565 MHz, $CDCl_3$) δ -156.80 (s) ppm; HRMS (ESI) calcd for $C_{16}H_{16}FO_3^+$: 275.1078, found 275.1075; HPLC analysis: Daicel Chiralpak OJ-H, flow rate =1.0 mL/min, λ= 223 nm, hexane/iso-propanol = 96:4, (*R*)-**3g**: 94% *ee*; $[\alpha]_D^{22}$ = -41.0 (c = 0.30, $CHCl_3$), retention time: 32.7 min (major) and 38.0 min (minor). (*S*)- **3g**: 86% *ee*; $[\alpha]_D^{22}$ = 32.7 (c = 0.22, $CHCl_3$), retention time: 31.9 min (minor) and 36.4 min (major), 95% yield; 89% *ee*; retention time: 31.9 min (minor) and 36.4 min (major)

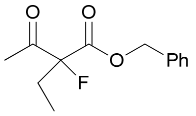

3h

Pale yellow oil; (*R*)-**3h**: 91% yield; (*S*)-**3h**: 92% yield; IR (thin film, cm^{-1}) 3567, 2921, 1756, 1732, 1260, 772; ^{1}H NMR (400 MHz, $CDCl_3$) δ 7.46 – 7.28 (m, 5H), 5.36 – 5.11 (m, 2H), 2.27 (d, J = 4.8 Hz, 3H), 2.24 – 1.98 (m, 2H), 0.92 (t, J = 7.4 Hz, 3H) ppm; ^{13}C NMR (101 MHz, $CDCl_3$) δ 202.32, 202.03, 166.42, 166.16, 134.86, 128.80, 128.78, 128.35, 101.87, 99.91, 68.03, 27.52, 27.30, 26.03, 7.16, 7.12 ppm; ^{19}F NMR (471 MHz, $CDCl_3$) δ -169.15 (s)ppm; HRMS (ESI) calcd for $C_{13}H_{16}FO_3^+$: 239.1078, found 239.1079; HPLC analysis: Daicel Chiralpak OJ-H, flow rate =1.0 mL/min, λ= 207 nm, hexane/iso-propanol = 90:10, (*R*)-**3h**: 92% *ee*; $[\alpha]_D^{22}$ = -21.3 (c = 0.34, $CHCl_3$), retention time: 11.4 min (major) and 15.2 min (minor); (*S*)-**3h**: 86% *ee*; $[\alpha]_D^{22}$ = 30.3 (c = 0.30, $CHCl_3$), retention time: 11.5 min (minor) and 15.0 min (major), 92% *ee*; retention time: 11.6 min (minor) and 15.4 min (major).

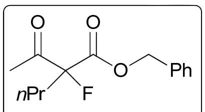

3i

Pale yellow oil; (*R*)-**3i**: 79% yield; (*S*)-**3i**: 95% yield; 95% yield; IR (thin film, cm^{-1}) 3735, 3567, 2966, 1756, 1732, 1219, 772; ^{1}H NMR (400 MHz, $CDCl_3$) δ 7.43 – 7.28 (m, 5H), 5.35 – 5.07 (m, 2H), 2.27 (d, J = 4.8 Hz, 3H), 2.22 – 1.88 (m, 2H), 1.48 – 1.17 (m, 2H), 0.91 (t, J = 7.4 Hz, 3H)ppm; ^{13}C NMR (101 MHz, $CDCl_3$) δ 202.33, 202.04, 166.50, 166.24, 134.87, 128.82, 128.79, 128.36, 101.66, 99.70, 68.05, 36.13, 35.92, 25.96, 16.38, 16.34, 14.03 ppm; ^{19}F NMR (471 MHz, $CDCl_3$) δ -167.12 (s) ppm; HRMS (ESI) calcd for $C_{14}H_{18}FO_3^+$: 253.1235, found 253.1233; HPLC analysis: Daicel Chiralpak OJ-H, flow rate =1.0 mL/min, λ= 207 nm, hexane/iso-propanol = 95:5, (*R*)-**3i**: 79% *ee*; $[\alpha]_D^{22}$ = -71.7 (c = 0.10, $CHCl_3$), retention time: 11.5 min (major) and 13.4 min (minor). (*S*)-**3i**: 85% *ee*; $[\alpha]_D^{22}$ = 24.3 (c = 0.35, $CHCl_3$), retention time: 11.8 min (minor) and 13.7 min (major), 90% *ee*; retention time: 12.0 min (minor) and 13.8 min (major).

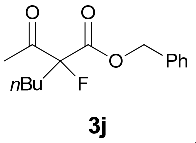

3j

Pale yellow oil; (*R*)-**3j**: 65% yield; (*S*)-**3j**: 92% yield; 95% yield; IR (thin film, cm^{-1}) 3019, 2962, 1756, 1732, 1219, 772; ^{1}H NMR (400 MHz, CDCl3) δ 7.45 – 7.27 (m, 5H), 5.30 – 5.14 (m, 2H), 2.27 (d, J = 4.8 Hz, 3H), 2.22 – 1.93 (m, 2H), 1.36 – 1.19 (m, 4H), 0.86 (t, J = 7.0 Hz, 3H) ppm; ^{13}C NMR (101 MHz, $CDCl_3$) δ 202.3(*S*)-**3j**: 92% yield; 5, 202.07, 166.52, 166.26, 134.88, 128.81, 128.41, 101.67, 99.70, 68.05, 33.89, 33.68, 25.98, 24.90, 24.88, 22.62, 13.84 ppm; ^{19}F NMR (471 MHz, $CDCl_3$) δ -167.15 (s) ppm; HRMS (ESI) calcd for $C_{15}H_{20}FO_3^+$: 267.1391, found 267.1390; HPLC analysis: Daicel Chiralpak OJ-H, flow rate =1.0 mL/min, λ= 207 nm, hexane/iso-propanol = 95:5, (*R*)-**3j**: 89% *ee*; $[\alpha]_D^{22}$ = -35.7 (c = 0.30, $CHCl_3$), retention time: 8.8 min (major) and 10.0 min (minor); (*S*)-**3j**: 87% *ee*; $[\alpha]_D^{22}$ = 28.2 (c = 0.45, $CHCl_3$), retention time: 9.0 min (minor) and 10.2 min (major), 91% *ee*; retention time: 9.2 min (minor) and 10.4 min (major).

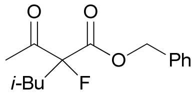

3k

Pale yellow oil; (*R*)-**3k**: 53% yield; (*S*)-**3k**: 23% yield; 63% yield; IR (thin film, cm^{-1}) 2961, 2920, 1756, 1732, 1219, 772; ^{1}H NMR (500 MHz, $CDCl_3$) δ 7.43 – 7.29 (m, 5H), 5.23 (s, 2H), 2.28 (d, *J* = 4.9 Hz, 3H), 2.16 – 2.03 (m, 1H), 1.95 (ddd, *J* = 21.6, 15.0, 6.9 Hz, 1H), 1.73 (dt, *J* = 13.3, 6.7 Hz, 1H), 0.90 (dd, *J* = 14.3, 6.7 Hz, 6H) ppm; ^{13}C NMR (126 MHz, $CDCl_3$) δ 202.30, 202.07, 166.67, 166.47, 134.73, 128.82, 128.81, 128.45, 101.78, 100.19, 68.13, 41.96, 41.80, 25.81, 24.39, 23.55, 23.54, 23.39 ppm;^{19}F NMR (565 MHz, $CDCl_3$) δ -163.92 (s) ppm; HRMS (ESI) calcd for $C_{15}H_{20}FO_3^+$: 267.1391, found 267.1391; HPLC analysis: Daicel Chiralpak OJ-H, flow rate =1.0 mL/min, λ= 207 nm, hexane/iso-propanol = 95:5, (*R*)-**3k**: 94% *ee*; $[\alpha]_D^{22}$ = -41.1 (c = 0.35, $CHCl_3$), retention time: 9.2 min (major) and 9.9 min (minor); (*S*)-**3k**: 79% *ee*; $[\alpha]_D^{22}$ = 28.0 (c = 0.30, $CHCl_3$), retention time: 9.2 min (minor) and 9.8 min (major), 87% *ee*; retention time: 9.2min (minor) and 9.7 min (major).

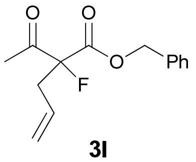

3l

Pale yellow oil; (*R*)-**3l**: 88% yield; (*S*)-**3l**: 66% yield; 99% yield; IR (thin film, cm^{-1}) 3735, 3648, 3567, 1757, 1732, 1220, 772; ^{1}H NMR (400 MHz, $CDCl_3$) δ 7.40 – 7.29 (m, 5H), 5.69 (ddt, J = 17.0, 9.7, 7.2 Hz, 1H), 5.24 (t, J = 7.7 Hz, 2H), 5.17 (dd, J = 13.4, 8.7 Hz, 2H), 3.02 – 2.70 (m, 2H), 2.27 (d, J = 4.8 Hz, 3H) ppm; ^{13}C NMR (101 MHz, $CDCl_3$) δ 201.82, 201.53, 165.97, 165.72, 134.75, 129.18, 129.15, 128.84, 128.81, 128.45, 121.13, 100.67, 98.69, 68.19, 38.36, 38.15, 26.09 ppm; ^{19}F NMR (565 MHz, $CDCl_3$) δ -166.18 (s) ppm; HRMS (ESI) calcd for $C_{14}H_{16}FO_3^+$: 251.1078, found 251.1075; HPLC analysis: Daicel Chiralpak OJ-H, flow rate =1.0 mL/min, λ= 207 nm, hexane/iso-propanol = 95:5, (*R*)-**3l**: 92% *ee*; $[\alpha]_D^{22}$ = -34.3 (c = 0.28, $CHCl_3$), retention time: 12.7 min (major) and 15.8 min (minor); (*S*)-**3l**: 79% *ee*; $[\alpha]_D^{22}$ = 24.0 (c = 0.40, $CHCl_3$), retention time: 12.7 min (major) and 15.8 min (minor); 90% *ee*; retention time: 13.2 min (minor) and 16.7 min (major).

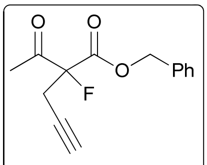

3m

Pale yellow oil; (*R*)-**3m**: 81% yield; (*S*)-**3m**: 60% yield; 70% yield; IR (thin film, cm^{-1})3295, 2920, 2850, 1757, 1733, 1283, 1219, 1078, 772, 698; ^{1}H NMR (400 MHz, $CDCl_3$) δ 7.35 (d, J = 5.0 Hz, 5H), 5.34 – 5.20 (m, 2H), 3.04 (ddd, J = 40.5, 22.7, 17.0 Hz, 2H), 3.00 – 2.84 (m, 1H), 2.34 (dd, J = 4.8, 1.4 Hz, 1H), 2.18 – 1.93 (m, 2H) ppm; ^{13}C NMR (101 MHz, $CDCl_3$) δ 201.16, 200.87, 165.06, 164.81, 134.51, 128.91, 128.84, 128.75, 128.58, 128.43, 98.96, 96.93, 72.31, 72.19, 68.56, 68.05, 26.21, 26.17, 24.76, 24.55, 21.94 ppm; ^{19}F NMR (565 MHz, $CDCl_3$) δ -164.45 (s) ppm; HRMS (ESI) calcd for $C_{14}H_{14}FO_3^+$: 249.0922, found 249.0918; HPLC analysis: Daicel Chiralpak OJ-H, flow rate =1.0 mL/min, λ= 207 nm, hexane/iso-propanol = 95:5, (*R*)-**3m**: 93% *ee*; $[\alpha]_D^{22}$ = -34.4 (c = 0.45, $CHCl_3$), retention time: 26.7 min (major) and 31.5 min (minor); (*S*)-**3m**: 82% *ee*; $[\alpha]_D^{22}$ = 54.0 (c = 0.20, $CHCl_3$), retention time: 28.0 min (minor) and 31.8 min (major), 87% *ee*; retention time: 27.5 min (minor) and 32.0 min (major).

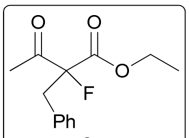

3n

Pale yellow oil[3]; (*R*)-**3n**: 79% yield; (*S*)-**3n**: 82% yield; 86% yield; IR (thin film, cm^{-1}) 3840, 3735, 3567, 1756, 1731, 1219, 772; ^{1}H NMR (400 MHz, $CDCl_3$) δ 7.25 (dt, J = 19.3, 7.4 Hz, 5H), 4.22 (q, J = 7.1 Hz, 2H), 3.54 – 3.25 (m, 2H), 2.13 (d, J = 5.1 Hz, 3H), 1.24 (t, 3H) ppm; ^{13}C NMR (101 MHz, $CDCl_3$) δ 202.70, 202.41, 166.00, 165.74, 133.22, 130.52, 128.55, 127.57, 101.13, 99.14, 62.80, 39.99, 39.78, 26.38, 14.07; ^{19}F NMR (471 MHz, $CDCl_3$) δ -164.56 (s) ppm; HRMS (ESI) calcd for $C_{13}H_{16}FO_3^+$: 239.1078, found 239.1077; HPLC analysis: Daicel Chiralpak OJ-H, flow rate =1.0 mL/min, λ= 207 nm, hexane/iso-propanol = 90:10, (*R*)-**3n**: 94% *ee*; $[\alpha]_D^{22}$ = -18.3 (c = 0.24, $CHCl_3$), retention time: 14.0 min (minor) and 20.5 min (major); (*S*)-**3n**: 79% *ee*; $[\alpha]_D^{22}$ = 4.0 (c = 0.10, $CHCl_3$), retention time: 13.9 min (major) and 20.6 min (minor), 87% *ee*; retention time: 14.6 min (major) and 22.1 min (minor).

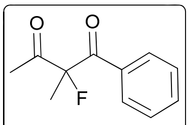

3o

Colorless oil; (*R*)-**3o**: 99% yield; (*S*)-**3o**: 57% yield; IR (thin film, cm^{-1}) 3567, 2919, 1730, 1689, 1219, 772; ^{1}H NMR (400 MHz, $CDCl_3$) δ 8.11 (d, J = 7.2 Hz, 1H), 7.98 (d, J = 8.3 Hz, 1H), 7.60 (t, J = 7.2 Hz, 1H), 7.48 (dt, J = 12.1, 6.0 Hz, 2H), 2.34 (d, J = 3.4 Hz, 3H), 1.82 (d, J = 22.6 Hz, 3H) ppm; ^{13}C NMR (126 MHz, $CDCl_3$) δ 202.70, 202.50, 194.05, 193.85, 134.12, 129.95, 129.90, 128.83, 128.83, 104.28, 102.72, 25.05, 20.96, 20.77 ppm. ^{19}F NMR (565 MHz, $CDCl_3$) δ -151.77 (s) ppm; HRMS (ESI) calcd for $C_{11}H_{12}FO_2^+$: 195.0816, found 195.0812. HPLC analysis: Daicel Chiralpak OJ-H, flow rate =0.5 mL/min, λ= 254 nm, hexane/iso-propanol = 95:5, (*R*)-**3o**: 53% *ee*; $[\alpha]_D^{22}$ = 39.5 (c = 0.25, $CHCl_3$), retention time: 46.2 min (minor) and 48.0 min (major); (*S*)-**3o**: 82% *ee*; $[\alpha]_D^{22}$ = -14.0 (c = 0.20, $CHCl_3$), retention time: 46.1 min (major) and 47.9 min (minor).

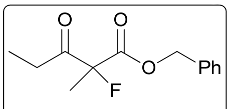

3p

Colorless oil; (*R*)-**3p**: 25% yield; (*S*)-**3p**: 20% yield; IR (thin film, cm^{-1}) 3557, 2920, 2850, 1769, 1752, 1727, 1470, 1314, 1270, 1164, 1126, 1044, 1021, 772; ^{1}H NMR (400 MHz, $CDCl_3$) δ 7.34 (td, *J* = 7.8, 1.4 Hz, 5H), 5.23 (s, 2H), 2.79 – 2.53 (m, 2H), 1.55 (s, 3H), 1.04 (t, *J* = 7.2 Hz, 3H). ^{19}F NMR (565 MHz, $CDCl_3$) δ -151.77 (s) ppm; ^{13}C NMR (101 MHz, $CDCl_3$) δ 205.24, 204.97, 167.17, 166.91, 134.86, 128.83, 128.79, 128.28, 98.92, 96.99, 68.06, 30.77, 20.38, 20.15, 7.14 ppm; ^{19}F NMR (565 MHz, $CDCl_3$) δ -159.14 (s) ppm; HRMS (ESI) calcd for $C_{13}H_{16}FO_3^+$: 239.1078, found 239.1072; HPLC analysis: Daicel Chiralpak OJ-H, flow rate =1.0 mL/min, λ= 207 nm, hexane/iso-propanol = 96:4, (*R*)-**3p**: 55% *ee*; $[\alpha]_D^{22}$ = -13.5 (c = 0.47, $CHCl_3$), retention time: 12.4 min (major) and 14.3 min (minor); (*S*)-**3p**: 84% *ee*; $[\alpha]_D^{22}$ = 4.9 (c = 0.31, $CHCl_3$), retention time: 12.6 min (minor) and 15.1 min (major).

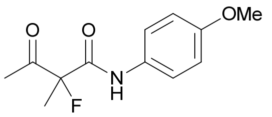

3q

Colorless oil; (*R*)-**3q**: 99% yield; (*S*)-**3q**: 84% yield; IR (thin film, cm^{-1}) 3325, 2922, 1733, 1667, 1532, 1514, 1450, 1247, 1220, 1137, 776; ^{1}H NMR (400 MHz, $CDCl_3$) δ 8.05 (s, 1H), 7.45 (d, J = 8.6 Hz, 2H), 6.88 (d, J = 8.6 Hz, 2H), 3.79 (s, 3H), 2.37 (d, J = 2.5 Hz, 3H), 1.82 (d, J = 22.8 Hz, 3H) ppm; ^{13}C NMR (101 MHz, $CDCl_3$) δ 201.25, 201.01, 164.74, 164.53, 157.20, 129.60, 121.84, 114.43, 100.71, 98.75, 55.63, 25.31, 21.08, 20.85; ^{19}F NMR (565 MHz, $CDCl_3$) δ -157.00 (s) ppm; HRMS (ESI) calcd for $C_{12}H_{15}FNO_3^+$: 240.1031, found 240.1032; HPLC analysis: Daicel Chiralpak AD-H, flow rate =1.0 mL/min, λ= 207 nm, hexane/iso-propanol = 90:10, (*R*)-**3q**: 52% *ee*; $[\alpha]_D^{22}$ = 5.0 (c = 0.61, $CHCl_3$), retention time: 11.6 min (major) and 12.5 min (minor); (*S*)-**3q**: 92% *ee*; $[\alpha]_D^{22}$ = -0.5 (c = 0.45, $CHCl_3$), retention time: 11.6 min (minor) and 12.5 min (major).

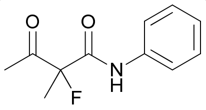

3r

Colorless oil; (*R*)-**3r**: 95% yield; (*S*)-**3r**: 97% yield; IR (thin film, cm^{-1}) 3320, 1740, 1688, 1602, 1545, 1494, 1446, 1135, 762; ^{1}H NMR (400 MHz, $CDCl_3$) δ 8.14 (s, 1H), 7.55 (d, J = 7.9 Hz, 2H), 7.36 (t, J = 7.9 Hz, 2H), 7.17 (t, J = 7.4 Hz, 1H), 2.37 (d, J = 3.1 Hz, 3H), 1.83 (d, J = 22.8 Hz, 3H) ppm; ^{13}C NMR (101 MHz, $CDCl_3$) δ 201.18, 200.94, 164.96, 164.76, 136.55, 129.34, 125.47, 120.12, 100.70, 98.74, 25.32, 21.10, 20.87 ppm; ^{19}F NMR (565 MHz, $CDCl_3$) δ -156.89 (s) ppm; HRMS (ESI) calcd for $C_{11}H_{11}FNO_2^-$: 208.0779, found 208.0774; HPLC analysis: Daicel Chiralpak OJ-H, flow rate =1.0 mL/min, λ= 207 nm, hexane/iso-propanol = 90:10, (*R*)-**3r**: 62% *ee*; $[\alpha]_D^{22}$ = -8.0 (c = 0.20, $CHCl_3$), retention time: 22.7 min (major) and 25.5 min (minor); (*S*)-**3r**: 92% *ee*; $[\alpha]_D^{22}$ = 7.1 (c = 0.35, $CHCl_3$), retention time: 23.0 min (minor) and 25.2 min (major).

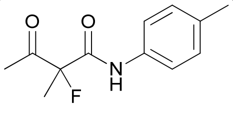

3s

Colorless oil; (*R*)-**3s**: 99% yield; (*S*)-**3s**: 81% yield; IR (thin film, cm^{-1}) 3336, 2923, 1738, 1682, 1599, 1532, 1407, 1358, 1319, 1220, 1139, 1128, 817, 776; ^{1}H NMR (400 MHz, $CDCl_3$) δ 8.11 (s, 1H), 7.43 (d, J = 8.2 Hz, 2H), 7.15 (d, J = 8.1 Hz, 2H), 2.36 (d, J = 2.9 Hz, 3H), 2.32 (s, 3H), 1.82 (d, J = 22.8 Hz, 3H) ppm; ^{13}C NMR (101 MHz, $CDCl_3$) δ 201.19, 200.95, 164.81, 164.61, 135.15, 133.98, 129.76, 120.13, 100.68, 98.72, 25.27, 21.02, 21.00, 20.79 ppm; ^{19}F NMR (565 MHz, $CDCl_3$) δ -158.46 (s) ppm; calcd for $C_{12}H_{13}FNO_2^-$: 222.0936, found 222.0931; HPLC analysis: Daicel Chiralpak AS-H, flow rate =1.0 mL/min, λ= 207 nm, hexane/iso-propanol = 90:10, (*R*)-**3s**: 71% *ee*; $[\alpha]_D^{22}$ = -2.6 (c = 0.61, $CHCl_3$), retention time: 12.7 min (major) and 14.5 min (minor); (*S*)-**3s**: 94% *ee*; $[\alpha]_D^{22}$ = 5.0 (c = 0.40, $CHCl_3$), retention time: 12.8 min (minor) and 14.5 min (major).

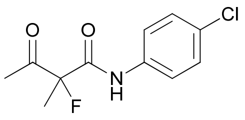

3t

Colorless oil; (*R*)-**3t**: 99% yield; (*S*)-**3t**: 55% yield; IR (thin film, cm^{-1}) 3336, 3019, 1738, 1689, 1597, 1532, 1494, 1402, 1219, 828, 762; ^{1}H NMR (400 MHz, $CDCl_3$) δ 8.17 (s, 1H), 7.51 (d, J = 7.9 Hz, 2H), 7.31 (d, J = 7.9 Hz, 2H), 2.55 – 2.24 (m, 3H), 1.82 (d, J = 22.8 Hz, 3H) ppm; ^{13}C NMR (101 MHz, $CDCl_3$) δ 201.16, 200.92, 164.97, 164.77, 135.12, 130.54, 129.35, 121.38, 100.65, 98.69, 25.34, 21.17, 20.94 ppm; ^{19}F NMR (565 MHz, $CDCl_3$) δ -164.45 (s) ppm; HRMS (ESI) calcd for $C_{11}H_{10}ClFNO_2^-$: 242.0390, found 242.0385; HPLC analysis: Daicel Chiralpak OJ-H, flow rate =1.0 mL/min, λ= 207 nm, hexane/iso-propanol = 90:10, (*R*)-**3t**: 83% *ee*; $[\alpha]_D^{22}$ = 5.1 (c = 0.40, $CHCl_3$), retention time: 24.3 min (major) and 26.3 min (minor); (*S*)-**3t**: 93% *ee*; $[\alpha]_D^{22}$ = -4.6 (c = 0.31, $CHCl_3$), retention time: 24.5 min (minor) and 25.5 min (major).

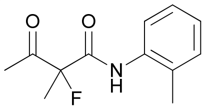

3u

Colorless oil; (*R*)-**3u**: 63% yield; (*S*)-**3u**: 82% yield; IR (thin film, cm^{-1}) 3342, 2920, 1738, 1682, 1526, 1458, 1219, 762; ^{1}H NMR (400 MHz, CDCl3) δ 8.07 (s, 1H), 7.85 (d, J = 7.9 Hz, 1H), 7.33 – 7.17 (m, 2H), 7.12 (t, J = 7.4 Hz, 1H), 2.39 (d, J = 3.2 Hz, 3H), 2.28 (s, 3H), 1.85 (d, J = 22.7 Hz, 3H) ppm; ^{13}C NMR (101 MHz, $CDCl_3$) δ 201.60, 201.35, 165.01, 164.81, 134.39, 130.80, 129.16, 127.10, 126.05, 122.63, 100.82, 98.86, 25.32, 21.29, 21.06, 17.61 ppm; ^{19}F NMR (565 MHz, $CDCl_3$) δ -156.89 (s) ppm; HRMS (ESI) calcd for $C_{12}H_{15}FNO_2^+$: 224.1081, found 224.1083; HPLC analysis: Daicel Chiralpak AS-H, flow rate =1.0 mL/min, λ= 207 nm, hexane/iso-propanol = 95:5, (*R*)-**3u**:79% *ee*; $[\alpha]_D^{22}$ = -7.3 (c = 0.30, $CHCl_3$), retention time: 11.2 min (minor) and 17.2 min (major); (*S*)-**3u**: 89% *ee*; $[\alpha]_D^{22}$ = 7.0 (c = 0.20, $CHCl_3$), retention time: 11.2 min (major) and 17.5 min (minor).

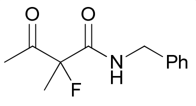

3v

Colorless oil; (*R*)-**3v**: 95% yield; (*S*)-**3v**: 93% yield; IR (thin film, cm^{-1}) 3355, 2920, 2849, 1738, 1668, 1532, 1220, 762; ^{1}H NMR (400 MHz, $CDCl_3$) δ 7.33 (dq, *J* = 14.4, 7.1 Hz, 3H), 7.27 (t, *J* = 3.4 Hz, 2H), 6.73 (s, 1H), 4.48 (d, *J* = 5.8 Hz, 2H), 2.32 (d, *J* = 3.2 Hz, 3H), 1.76 (d, *J* = 22.7 Hz, 3H) ppm; ^{13}C NMR (101 MHz, $CDCl_3$) δ 201.31,201.07, 167.04, 166.83, 137.28, 129.05, 128.06, 127.90, 100.73, 98.78, 43.72, 25.24, 21.00, 20.78 ppm; ^{19}F NMR (565 MHz, $CDCl_3$) δ -158.46 (s) ppm; HRMS (ESI) calcd for $C_{12}H_{13}FNO_2^-$: 222.0936, found 222.0931; HPLC analysis: Daicel Chiralpak OJ-H, flow rate =1.0 mL/min, λ= 207 nm, hexane/iso-propanol = 90:10, (*R*)-**3v**: 56% *ee*; $[\alpha]_D^{22}$ = -20.9 (c = 0.35, $CHCl_3$), retention time: 28.8 min (major) and 34.9 min (minor); (*S*)-**3v**: 92% *ee*; $[\alpha]_D^{22}$ = 3.0 (c = 0.35, $CHCl_3$), retention time: 28.0 min (minor) and 32.9 min (major).

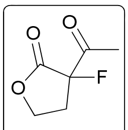

3w

Pale yellow liquid[3]; (*R*)-**3w**: 99% yield; (*S*)-**3w**: 88% yield; 99% yield; ^{1}H NMR (400 MHz, $CDCl_3$) δ 4.55 – 4.37 (m, 2H), 2.96 – 2.76 (m, 1H), 2.65 – 2.48 (m, 1H), 2.47 (d, *J* = 4.9 Hz, 3H) ppm; ^{13}C NMR (101 MHz, $CDCl_3$) δ 203.45, 203.14, 169.31, 169.07, 97.41, 95.38, 65.90, 65.85, 32.27, 32.06, 26.05 ppm; ^{19}F NMR (565 MHz, $CDCl_3$) δ -162.52 (s) ppm; HPLC analysis: Daicel Chiralpak OD-H, flow rate =0.5 mL/min, λ= 227 nm, hexane/iso-propanol = 95:5, (*R*)-**3w**: 76% *ee*; $[\alpha]_D^{22}$ = -29.1 (c = 0.28, $CHCl_3$), retention time: 18.6 min (major) and 20.5 min (minor); (*S*)-**3w**: 43% ; $[\alpha]_D^{22}$ = 14.0 (c = 0.20, $CHCl_3$), retention time: 19.1 min (minor) and 21.0 min (major), 83% *ee*; retention time: 19.2 min (minor) and 21.0 min (major).

The spectroscopic data for **3w** matched those described in the literature[3].

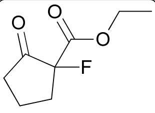

3x

Colorless oil; (*R*)-**3x**: 52% yield; (*S*)-**3x**:56% yield; IR (thin film, cm^{-1}) 3355, 2920, 2849, 1738, 1668, 1532, 1220, 762; ^{1}H NMR (400 MHz, $CDCl_3$) δ 4.29 (q, *J* = 7.1 Hz, 2H), 2.64 – 2.52 (m, 1H), 2.49 (dd, *J* = 10.4, 5.1 Hz, 2H), 2.32 (ddd, *J* = 28.6, 14.3, 7.0 Hz, 1H), 2.22 – 2.03 (m, 2H), 1.31 (t, *J* = 7.1 Hz, 3H)； ^{13}C NMR (101 MHz, $CDCl_3$) δ 207.77, 207.60, 167.76, 167.49, 95.79, 93.80, 62.53, 35.87, 34.17, 33.97, 18.24, 18.21, 14.20； ^{19}F NMR (565 MHz, $CDCl_3$) δ -164.06 (s). HRMS (ESI) calcd for $C_8H_{12}FNO_3^+$:175.0765, found: 175.0765; GC analysis: CP-Chirasil-Dex CB, isotherm 80°C, N_2, (*R*)-**3x**: 48% *ee*; $[\alpha]_D^{22}$ = 75.0 (c = 0.30, $CHCl_3$), retention time: 26.9 min (minor) and 30.5 min (major); (*S*)-**3x**: 13% *ee*; $[\alpha]_D^{22}$ = -1.56 (c = 0.32, $CHCl_3$), retention time: 26.5 min (major) and 30.2 min (minor).

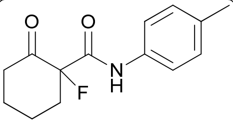

3y

White solid; (*R*)-**3z**: 97% yield; (*S*)-**3z:** 81% yield; IR (thin film, cm^{-1}) 3351, 2922,2865, 1731, 1684, 1676, 1654, 1597, 1529, 1406, 1316, 1111, 1070, 816,773; ^{1}H NMR (400 MHz, $CDCl_3$) δ 7.98 (s, 1H), 7.39 (d, *J* = 8.4 Hz, 2H), 7.20 – 7.04 (m, 2H), 2.96 (ddd, *J* = 14.1, 12.0, 5.9 Hz, 1H), 2.71 – 2.49 (m, 2H), 2.34 (m, 1H), 2.32 (s, 3H), 2.17 – 1.97 (m, 2H), 1.97 – 1.84 (m, 1H), 1.85 – 1.66 (m, 1H); ^{13}C NMR (101 MHz, $CDCl_3$) δ 202.51, 202.34, 165.02, 164.82, 135.12, 133.94, 129.76, 120.35, 120.00, 99.33, 97.33, 40.42, 37.54, 37.34, 26.39, 22.09, 22.01, 21.03; ^{19}F NMR (565 MHz, $CDCl_3$) δ -154.90 (s) ppm; HRMS (ESI) calcd for $C_{14}H_{17}FNO_2^+$:250.1238, found:250.1240; HPLC analysis: Daicel Chiralpak OD-H, flow rate =1.0 mL/min, λ= 248 nm, hexane/iso-propanol = 95:5, (*R*)-**3o**: 55% *ee*; $[\alpha]_D^{22}$ = 67.7 (c = 0.40, $CHCl_3$), retention time: 11.1 min (minor) and 12.4 min (major); (*S*)-**3o**: 74% *ee*; $[\alpha]_D^{22}$ = -126.6 (c = 0.32, $CHCl_3$), retention time: 11.1 min (major) and12.5 min (minor).

Mechanism studies

In-situ ESI-MS studies of the reaction mixture

An oven-dried 10 mL schlenk tube was charged with **1a** (0.15 mmol), primary amine **II** (0.02 mmol), DNBA I (0.02 mmol), followed by $CHCl_3$ (0.5 mL). The mixture was stirred under air at room temperature for 10 min. Then an aliquot was taken for ESI-MS analysis.

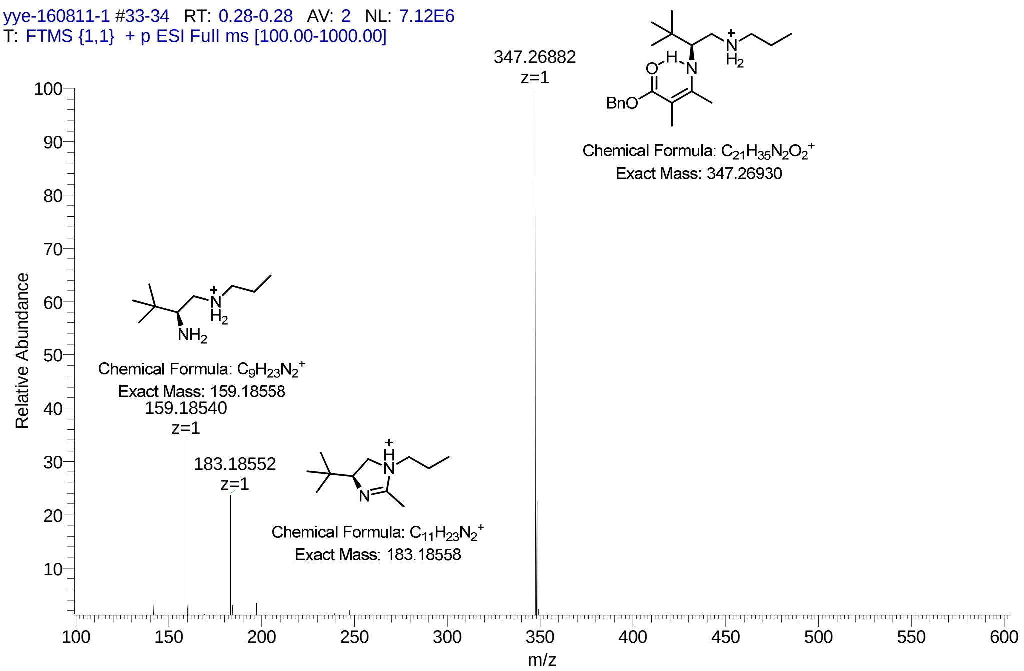

Reaction with NFSI (2a): To the above reaction mixture, NFSI (**2a**, 0.10 mmol) was added. The reaction was stirred under air at room temperature for 1h. Then an aliquot was taken for ESI-MS analysis. It was found that the enamine signal decreased whereas a fluorinated iminium ion was clearly noted, a clear indication of the enamine pathway.

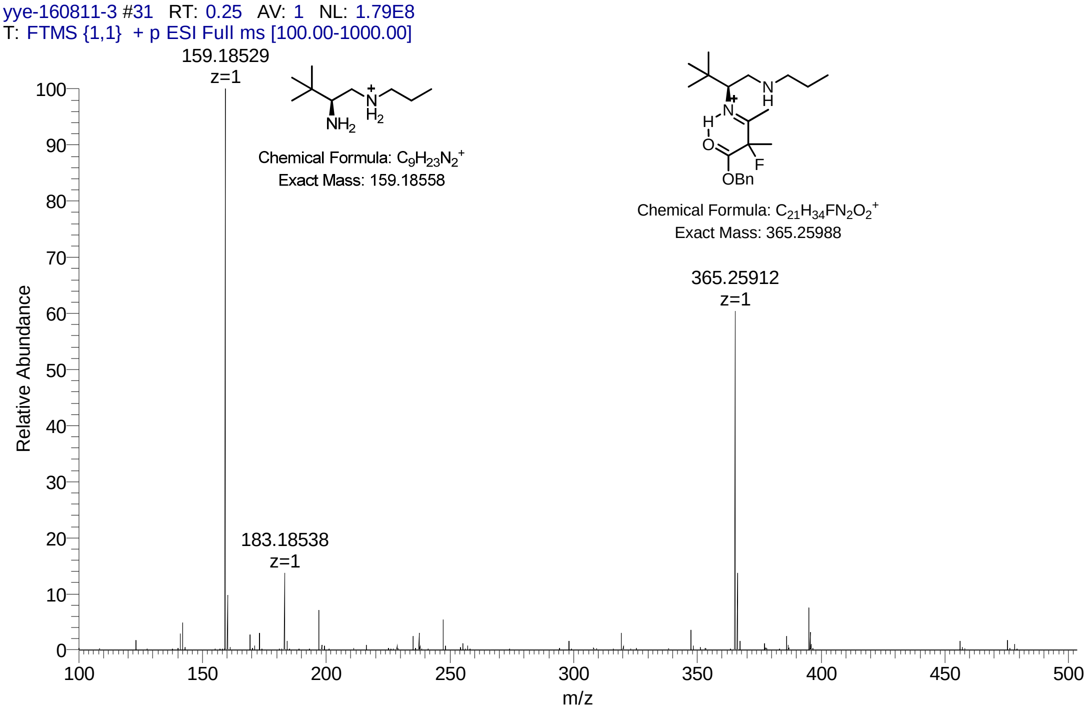

Reaction with 2b: An oven-dried 10 mL schlenk tube was charged with **1a** (0.15 mmol), primary amine **II** (0.02 mmol), DNBA II (0.02 mmol) and then NFCO-OTf (**2b**, 0.10 mmol) and 0.8 ml MeOH was added. The reaction was stirred under air at room temperature for 1h. Then an aliquot was taken for ESI-MS analysis. The pattern is similar to the case with NFSI, both enamine intermediate and the fluorinated iminium ion were clearly observed, verifying again the enamine mechanism.

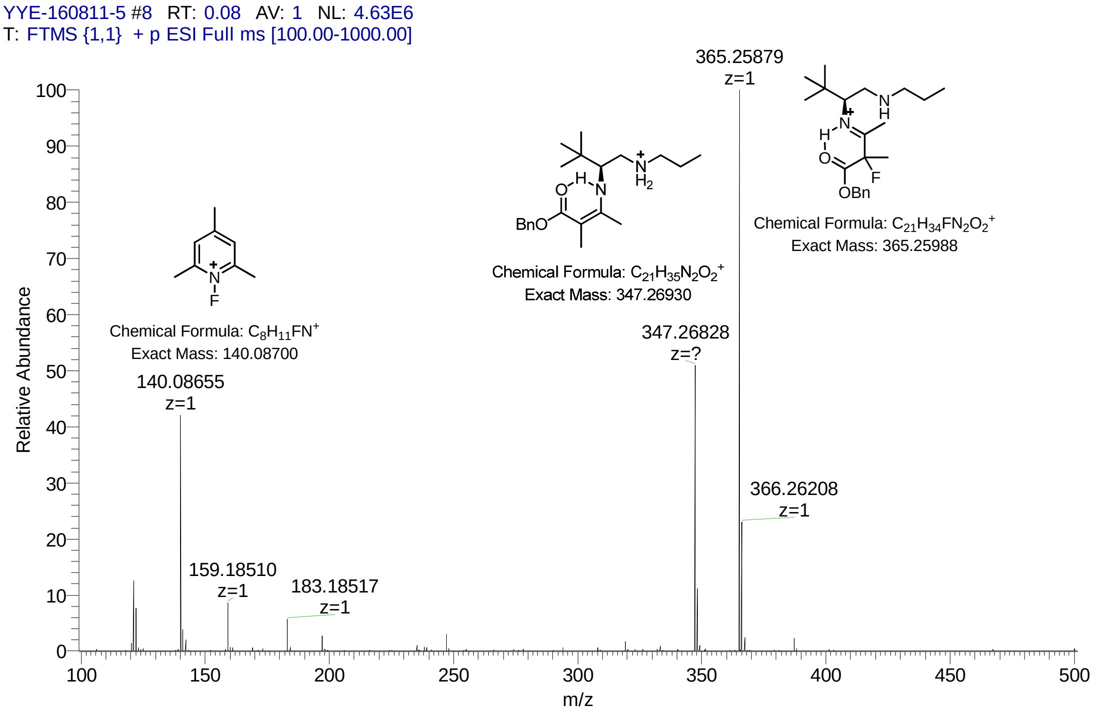

Calculated transition states

DFT calculations were performed with the Gaussian 09 program package.[7] The recently developed M06-2X functional[8] together with the 6-31G(d) basis set were used for the geometry optimizations and vibrational calculations. The transition state nature was confirmed by calculation of harmonic vibrational frequencies as only one imaginary frequency existed. The SMD continuum solvation model[8,9] with chloroform or methanol as the solvent were used in single point energy calculations and these calculations were performed at the M06-2x/6-311+G(d,p) level with gas phase optimized structures.

1. The fluorination transition state with NFSI

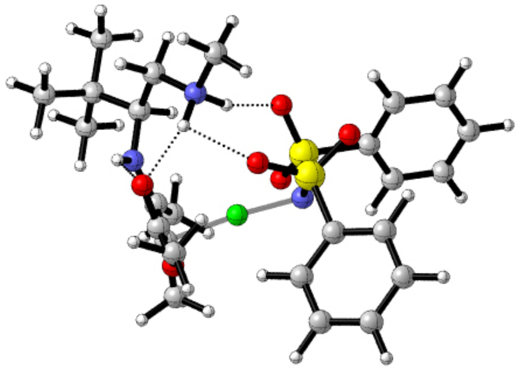

TS-R$_{\mathrm{NFSI}}$

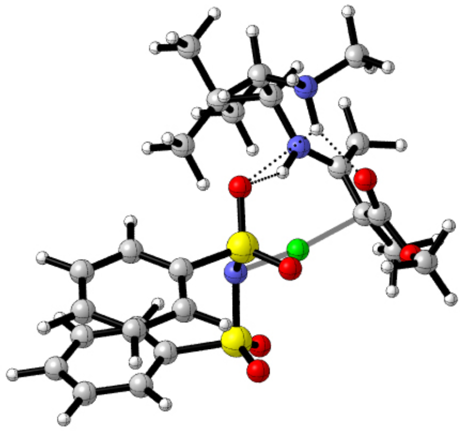

TS-S$_{\mathrm{NFSI}}$

2. The fluorination transition state with NFCO-OTf

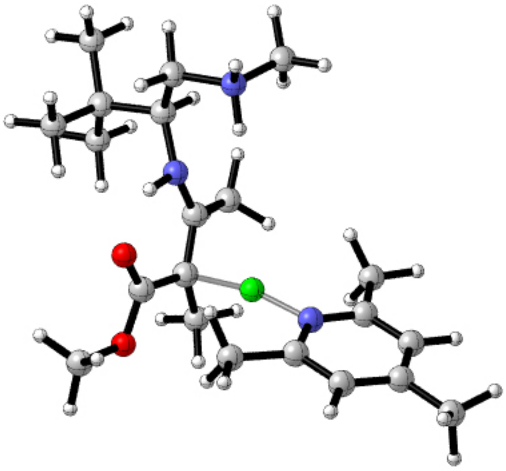

TS-R$_{\mathrm{NFCO\text{-}OTf}}$

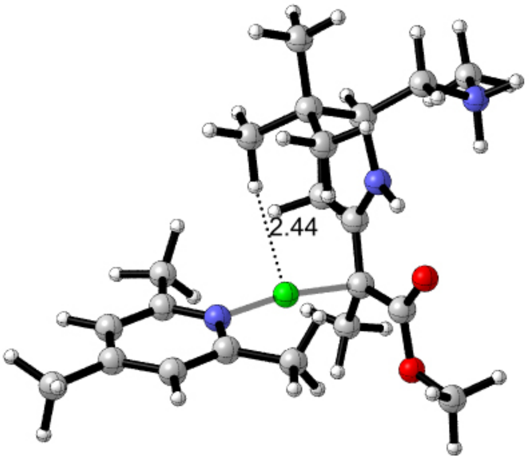

TS-S$_{\mathrm{NFCO\text{-}OTf}}$

3. The energies and coordinates of transition states.

(1) TS-R$_{\mathrm{NFSI}}$

E_{sol} = -2486.16768378

Zero-point correction= 0.618948 (Hartree/Particle)

Thermal correction to Energy= 0.657741

Thermal correction to Enthalpy= 0.658685

Thermal correction to Gibbs Free Energy= 0.547896
Sum of electronic and zero-point Energies= -2484.925381
Sum of electronic and thermal Energies= -2484.886588
Sum of electronic and thermal Enthalpies= -2484.885644
Sum of electronic and thermal Free Energies= -2484.996433

	E (Thermal)	CV	S
	KCal/Mol	Cal/Mol-Kelvin	Cal/Mol-Kelvin
Total	412.739	147.179	233.176
Electronic	0.000	0.000	0.000
Translational	0.889	2.981	44.844
Rotational	0.889	2.981	37.364
Vibrational	410.961	141.217	150.968

Standard orientation:

Center Number	Atomic Number	Atomic Type	Coordinates (Angstroms) X	Y	Z
1	6	0	-5.099065	1.066480	0.083986
2	6	0	-5.450254	2.340547	0.863659
3	1	0	-4.789622	3.172828	0.593062
4	1	0	-6.474991	2.642954	0.629609
5	1	0	-5.398906	2.192640	1.947501
6	6	0	-5.396433	1.293563	-1.401890
7	1	0	-4.842015	2.152744	-1.796324
8	1	0	-5.147566	0.409892	-1.998796
9	1	0	-6.462727	1.495895	-1.539183
10	6	0	-5.951293	-0.106672	0.582911
11	1	0	-5.734064	-1.025122	0.027490
12	1	0	-5.820518	-0.304031	1.652177
13	1	0	-7.009639	0.124212	0.429769
14	6	0	-3.582714	0.768409	0.241984
15	1	0	-3.009625	1.570439	-0.238336
16	6	0	-3.153205	0.701487	1.718725
17	1	0	-3.795753	0.027673	2.293963
18	1	0	-3.211721	1.696462	2.164312
19	7	0	-3.183966	-0.501479	-0.370806
20	1	0	-1.105489	0.791106	1.307394
21	7	0	-1.752250	0.227065	1.901617
22	6	0	-1.274963	0.362239	3.306938
23	1	0	-1.287394	1.419249	3.573686
24	1	0	-1.936976	-0.205117	3.962287
25	1	0	-3.245182	-1.307197	0.252125

26	6	0	-2.399844	-0.710912	-1.428518
27	6	0	-1.571715	-1.867449	-1.479208
28	6	0	-2.360150	0.280383	-2.548596
29	1	0	-2.707522	1.265352	-2.240633
30	1	0	-1.344994	0.385473	-2.928212
31	1	0	-3.018055	-0.073107	-3.352324
32	6	0	-1.035662	-2.410256	-2.773952
33	1	0	-1.145545	-1.693059	-3.586817
34	1	0	0.023676	-2.656107	-2.675923
35	1	0	-1.563489	-3.327800	-3.054726
36	6	0	-1.421155	-2.703827	-0.267426
37	8	0	-1.972801	-2.516953	0.818310
38	8	0	-0.550545	-3.685021	-0.445280
39	6	0	-0.200771	-4.432358	0.732745
40	1	0	0.238280	-3.756766	1.469760
41	1	0	-1.088383	-4.913837	1.145286
42	1	0	0.523820	-5.171798	0.397446
43	1	0	-1.632764	-0.755055	1.594079
44	1	0	-0.260230	-0.029100	3.352273
45	6	0	2.134580	2.861263	-0.426306
46	6	0	2.754669	3.161272	-1.638379
47	6	0	3.863168	3.998788	-1.619799
48	6	0	4.324030	4.517956	-0.410280
49	6	0	3.684146	4.207970	0.788781
50	6	0	2.575958	3.368289	0.793299
51	1	0	2.364287	2.757463	-2.566769
52	1	0	4.363230	4.251156	-2.548585
53	1	0	5.189594	5.172606	-0.403022
54	1	0	4.048468	4.620584	1.723397
55	1	0	2.065714	3.101557	1.711425
56	16	0	0.717513	1.815411	-0.479372
57	8	0	-0.061557	2.016951	0.759746
58	8	0	0.028394	1.981392	-1.745375
59	7	0	1.354046	0.245128	-0.522238
60	16	0	1.799059	-0.462343	0.965066
61	6	0	2.994216	-1.639426	0.418667
62	6	0	2.578086	-2.675390	-0.414143
63	6	0	4.304280	-1.507724	0.861825
64	6	0	3.519514	-3.615478	-0.812493
65	1	0	1.543813	-2.731967	-0.740909
66	6	0	5.232869	-2.463531	0.456040
67	1	0	4.579238	-0.679147	1.505857
68	6	0	4.841172	-3.509406	-0.375299
69	1	0	3.225633	-4.431124	-1.465188

70	1	0	6.262108	-2.388681	0.790255
71	1	0	5.571118	-4.248443	-0.689957
72	8	0	2.437369	0.551959	1.791157
73	8	0	0.643875	-1.180382	1.512154
74	9	0	-0.090049	-0.673087	-0.952653

（2） **TS-S$_{NFSI}$**

E$_{sol}$ = -2486.15818096

Zero-point correction= 0.618283 (Hartree/Particle)
Thermal correction to Energy= 0.657099
Thermal correction to Enthalpy= 0.658043
Thermal correction to Gibbs Free Energy= 0.547905
Sum of electronic and zero-point Energies= -2484.901263
Sum of electronic and thermal Energies= -2484.862447
Sum of electronic and thermal Enthalpies= -2484.861503
Sum of electronic and thermal Free Energies= -2484.971641

	E (Thermal)	CV	S
	KCal/Mol	Cal/Mol-Kelvin	Cal/Mol-Kelvin
Total	412.336	147.385	231.805
Electronic	0.000	0.000	0.000
Translational	0.889	2.981	44.844
Rotational	0.889	2.981	37.166
Vibrational	410.558	141.423	149.795

Standard orientation:

Center Number	Atomic Number	Atomic Type	Coordinates (Angstroms) X	Y	Z
1	6	0	2.475652	3.138906	-0.351528
2	6	0	2.903208	4.404629	0.403302
3	1	0	3.995040	4.501457	0.444000
4	1	0	2.513941	5.288113	-0.110785
5	1	0	2.512460	4.430920	1.426086
6	6	0	2.904079	3.263799	-1.816828
7	1	0	3.990114	3.377269	-1.914999
8	1	0	2.582331	2.397039	-2.402399
9	1	0	2.439133	4.148799	-2.261027
10	6	0	0.949625	2.978995	-0.299733
11	1	0	0.614141	2.070521	-0.813434
12	1	0	0.562379	2.947732	0.723714
13	1	0	0.488158	3.837948	-0.797056

14	6	0	3.195240	1.907908	0.278546
15	1	0	4.269689	2.000219	0.081581
16	6	0	2.956826	1.851176	1.802747
17	1	0	1.910925	2.059653	2.029837
18	1	0	3.596273	2.567190	2.323367
19	7	0	2.724010	0.642452	-0.265381
20	1	0	2.705628	-0.252599	1.855703
21	7	0	3.232183	0.495326	2.376817
22	6	0	4.661073	0.083318	2.359448
23	1	0	4.987329	0.031114	1.320403
24	1	0	5.257399	0.808963	2.913587
25	1	0	1.826379	0.335951	0.123919
26	6	0	3.221309	-0.171557	-1.189549
27	6	0	2.661992	-1.476821	-1.290066
28	6	0	4.301262	0.272175	-2.128395
29	1	0	4.843946	1.144192	-1.761347
30	1	0	5.002847	-0.544318	-2.312251
31	1	0	3.856626	0.545984	-3.092762
32	6	0	2.766499	-2.251898	-2.573540
33	1	0	2.787444	-1.584057	-3.436847
34	1	0	3.654159	-2.894984	-2.600633
35	1	0	1.889574	-2.894933	-2.669910
36	6	0	2.494053	-2.237355	-0.031321
37	8	0	2.639151	-1.791378	1.106544
38	8	0	2.198321	-3.501868	-0.257740
39	6	0	1.931045	-4.307596	0.901349
40	1	0	2.820629	-4.356448	1.532026
41	1	0	1.097238	-3.874524	1.454456
42	1	0	1.679228	-5.291322	0.511477
43	1	0	2.870984	0.471101	3.335090
44	1	0	4.738667	-0.907684	2.804961
45	6	0	-3.232690	-0.001965	-1.482313
46	6	0	-3.047617	1.244903	-2.077697
47	6	0	-4.089532	2.162199	-2.031329
48	6	0	-5.289540	1.825342	-1.402244
49	6	0	-5.456854	0.571105	-0.821209
50	6	0	-4.421000	-0.359003	-0.856265
51	1	0	-2.108006	1.478178	-2.569284
52	1	0	-3.972194	3.137140	-2.493045
53	1	0	-6.101484	2.545350	-1.372890
54	1	0	-6.394964	0.312600	-0.340473
55	1	0	-4.522939	-1.343911	-0.412552
56	16	0	-1.897964	-1.171964	-1.505854
57	8	0	-2.327981	-2.378849	-0.824008

58	8	0	-1.291686	-1.206942	-2.819008
59	7	0	-0.762787	-0.289549	-0.552452
60	16	0	-0.595132	-0.630547	1.086842
61	6	0	-2.159731	-0.109966	1.716730
62	6	0	-2.518866	1.234071	1.612099
63	6	0	-3.018079	-1.080769	2.222237
64	6	0	-3.781499	1.614067	2.045574
65	1	0	-1.826575	1.953474	1.184692
66	6	0	-4.276881	-0.678364	2.663281
67	1	0	-2.700698	-2.118126	2.251038
68	6	0	-4.653276	0.659343	2.573793
69	1	0	-4.090635	2.651163	1.968753
70	1	0	-4.964210	-1.412401	3.070441
71	1	0	-5.638605	0.964204	2.912503
72	8	0	-0.385146	-2.032410	1.393399
73	8	0	0.454895	0.323512	1.511667
74	9	0	0.800570	-0.904973	-1.040447

（3） **TS-R$_{NFCO-OTf}$**

E$_{sol}$ = -1237.09569733

Zero-point correction= 0.582632 (Hartree/Particle)

Thermal correction to Energy= 0.614993

Thermal correction to Enthalpy= 0.615937

Thermal correction to Gibbs Free Energy= 0.518819

Sum of electronic and zero-point Energies= -1235.906525

Sum of electronic and thermal Energies= -1235.874164

Sum of electronic and thermal Enthalpies= -1235.873220

Sum of electronic and thermal Free Energies= -1235.970338

	E (Thermal) KCal/Mol	CV Cal/Mol-Kelvin	S Cal/Mol-Kelvin
Total	385.914	119.652	204.401
Electronic	0.000	0.000	0.000
Translational	0.889	2.981	43.723
Rotational	0.889	2.981	35.973
Vibrational	384.136	113.690	124.705

Standard orientation:

Center Number	Atomic Number	Atomic Type	Coordinates (Angstroms) X	Y	Z
1	6	0	-4.426285	-0.064436	-0.039319

2	6	0	-5.701041	-0.908881	-0.186758
3	1	0	-5.693851	-1.779527	0.479994
4	1	0	-6.571173	-0.303087	0.079389
5	1	0	-5.860859	-1.252369	-1.214195
6	6	0	-4.398661	0.550361	1.364826
7	1	0	-4.396264	-0.220313	2.144862
8	1	0	-3.533403	1.207794	1.509848
9	1	0	-5.292268	1.162910	1.512417
10	6	0	-4.417270	1.057720	-1.085037
11	1	0	-3.514006	1.675827	-1.030540
12	1	0	-4.524382	0.683638	-2.109012
13	1	0	-5.270089	1.718570	-0.905702
14	6	0	-3.184400	-1.002456	-0.183362
15	1	0	-3.248242	-1.740351	0.621113
16	6	0	-3.171260	-1.730786	-1.529121
17	1	0	-3.151963	-1.021151	-2.360725
18	1	0	-4.032650	-2.390592	-1.641762
19	7	0	-1.917012	-0.271630	-0.076708
20	1	0	-1.126259	-1.990697	-1.442745
21	7	0	-1.935494	-2.586041	-1.671567
22	6	0	-1.911382	-3.813142	-0.810329
23	1	0	-1.889425	-3.507322	0.234667
24	1	0	-2.808130	-4.397340	-1.017242
25	1	0	-1.750247	0.424292	-0.824306
26	6	0	-1.252857	0.056141	1.046125
27	6	0	-0.339953	1.148279	1.060195
28	6	0	-1.433102	-0.747211	2.298900
29	1	0	-2.057283	-1.629291	2.165933
30	1	0	-0.454670	-1.068114	2.666214
31	1	0	-1.882124	-0.126867	3.082191
32	6	0	0.177613	1.670991	2.376349
33	1	0	0.470728	0.859404	3.043900
34	1	0	1.052186	2.296736	2.201209
35	1	0	-0.573359	2.284333	2.886676
36	6	0	-0.371564	2.099138	-0.088405
37	8	0	-0.861915	1.847543	-1.185338
38	8	0	0.208465	3.251034	0.185441
39	6	0	0.208815	4.230847	-0.871194
40	1	0	0.740951	3.839404	-1.740743
41	1	0	-0.817469	4.472815	-1.149606
42	1	0	0.716103	5.098853	-0.457434
43	1	0	-1.832722	-2.865066	-2.654292
44	1	0	-1.020455	-4.392820	-1.050180
45	6	0	3.482070	0.482377	-0.802970

46	6	0	3.379776	-1.268557	0.801510
47	6	0	4.712200	-1.585825	0.560035
48	6	0	5.453431	-0.861796	-0.380643
49	6	0	4.815095	0.178598	-1.063526
50	1	0	5.169155	-2.397823	1.117814
51	1	0	5.353705	0.770222	-1.797711
52	6	0	6.902860	-1.167440	-0.623536
53	1	0	7.133743	-2.211152	-0.401870
54	1	0	7.185249	-0.953967	-1.656636
55	1	0	7.524605	-0.542179	0.026778
56	6	0	2.515827	-1.960258	1.814089
57	1	0	1.567915	-2.262087	1.360318
58	1	0	3.021010	-2.840859	2.211545
59	1	0	2.296580	-1.280514	2.643670
60	6	0	2.733794	1.616896	-1.437299
61	1	0	1.783527	1.273584	-1.855471
62	1	0	2.514230	2.373816	-0.676854
63	1	0	3.332614	2.072848	-2.226178
64	7	0	2.847310	-0.275209	0.090782
65	9	0	1.228971	0.246290	0.500347

（4） **TS-$S_{NFCO-OTf}$**

E_{sol} = -1237.10108042

Zero-point correction= 0.582982 (Hartree/Particle)

Thermal correction to Energy= 0.615379

Thermal correction to Enthalpy= 0.616324

Thermal correction to Gibbs Free Energy= 0.519359

Sum of electronic and zero-point Energies= -1235.912684

Sum of electronic and thermal Energies= -1235.880287

Sum of electronic and thermal Enthalpies= -1235.879343

Sum of electronic and thermal Free Energies= -1235.976308

	E (Thermal)	CV	S
	KCal/Mol	Cal/Mol-Kelvin	Cal/Mol-Kelvin
Total	386.156	119.672	204.080
Electronic	0.000	0.000	0.000
Translational	0.889	2.981	43.723
Rotational	0.889	2.981	35.788
Vibrational	384.379	113.711	124.568

Standard orientation:

Center Atomic Atomic Coordinates (Angstroms)

Number	Number	Type	X	Y	Z
1	6	0	1.737699	-2.284461	-0.789543
2	6	0	2.462213	-3.599602	-1.104632
3	1	0	2.997969	-3.987524	-0.229975
4	1	0	1.732148	-4.357199	-1.402455
5	1	0	3.169696	-3.499039	-1.934766
6	6	0	0.627069	-2.541336	0.236183
7	1	0	1.026682	-2.949320	1.172696
8	1	0	0.068258	-1.623061	0.449068
9	1	0	-0.080954	-3.272179	-0.166135
10	6	0	1.104988	-1.705267	-2.061248
11	1	0	0.537806	-0.790709	-1.851093
12	1	0	1.834970	-1.492898	-2.849925
13	1	0	0.403088	-2.434849	-2.475036
14	6	0	2.749054	-1.283279	-0.148449
15	1	0	3.059156	-1.703250	0.812187
16	6	0	3.994512	-1.085900	-1.022577
17	1	0	3.725365	-0.810828	-2.045984
18	1	0	4.608158	-1.987678	-1.047079
19	7	0	2.145285	0.030322	0.064591
20	1	0	4.293869	0.879114	-0.441796
21	7	0	4.869454	0.027985	-0.503027
22	6	0	5.503423	-0.230656	0.831440
23	1	0	4.717838	-0.279237	1.584742
24	1	0	6.045181	-1.175401	0.780205
25	1	0	1.892809	0.552691	-0.790444
26	6	0	1.506523	0.504762	1.141832
27	6	0	0.643327	1.633359	1.027444
28	6	0	1.663692	-0.153275	2.479502
29	1	0	2.368769	-0.982740	2.479197
30	1	0	1.988297	0.583966	3.220119
31	1	0	0.694632	-0.538876	2.812131
32	6	0	0.147873	2.339254	2.261721
33	1	0	-0.103118	1.630955	3.052453
34	1	0	0.890270	3.044931	2.650681
35	1	0	-0.749857	2.906617	2.018370
36	6	0	0.649977	2.406935	-0.248268
37	8	0	1.190979	2.036953	-1.287033
38	8	0	-0.011597	3.543155	-0.156349
39	6	0	-0.058575	4.354054	-1.346916
40	1	0	0.953364	4.632176	-1.643519
41	1	0	-0.537560	3.799262	-2.156119
42	1	0	-0.639986	5.230857	-1.073659

43	1	0	5.604817	0.220425	-1.193716
44	1	0	6.187861	0.586422	1.058609
45	6	0	-3.037474	-0.903506	1.152849
46	6	0	-3.053215	0.365604	-0.855733
47	6	0	-4.322422	-0.114946	-1.163364
48	6	0	-4.971123	-1.006795	-0.303182
49	6	0	-4.308344	-1.392481	0.866444
50	1	0	-4.804354	0.224083	-2.075585
51	1	0	-4.778575	-2.071505	1.571478
52	6	0	-6.356266	-1.500458	-0.605158
53	1	0	-6.539736	-2.474895	-0.148257
54	1	0	-7.093729	-0.797645	-0.202021
55	1	0	-6.524519	-1.577173	-1.681418
56	6	0	-2.310810	1.371790	-1.684437
57	1	0	-1.341738	0.980378	-2.007056
58	1	0	-2.895356	1.643105	-2.563970
59	1	0	-2.130837	2.269802	-1.084850
60	6	0	-2.272974	-1.241740	2.398565
61	1	0	-1.392782	-1.845961	2.156786
62	1	0	-1.942782	-0.325588	2.895715
63	1	0	-2.901406	-1.809355	3.085442
64	7	0	-2.485439	-0.076256	0.265834
65	9	0	-0.897638	0.610508	0.627267

References:

(1) D. Kalaitzakis, S. Kambourakis, D. J. Rozzell and I. Smonou, *Tetrahedron: Asymmetry*, 2007, **18**, 2418.

(2) R. D. Chambers, M. A. Fox and G. Sandford, *Lab Chip*, 2005, **5**, 1132.

(3) M. Frings and C. Bolm, *Eur. J. Org. Chem.*, 2009, 4085.

(4) A. L. Searles, and H. G. Lindwall, *J. Am. Chem. Soc.*, 1946, **68**, 988.

(5) J. Hutchinson, G. Sandford and J. F. S. Vaughan, *Tetrahedron*, 1998, **54**, 2867.

(6) (a) V. A. Soloshonok, C. Roussel, O. Kitagawa and A. E. Sorochinsky, *Chem. Soc. Rev.*, 2012, **41**, 4180; (b) M. Maeno, E. Tokunaga, T. Yamamoto, T. Suzuki, Y. Ogino, E. Ito, M. Shiro, T. Asahi and N. Shibata, *Chem. Sci*., 2015, **6**, 1043; (c) S. Ogawa, T. Nishimine, E. Tokunaga, S. Nakamura and N. Shibata, *J. Fluorine Chem.*, 2010, **131**, 521.

(7) M. J. Frisch, G. W. Trucks, H. B. Schlegel, G. E. Scuseria, M. A. Robb, J. R. Cheeseman, G. Scalmani, V. Barone, B. Mennucci, G. A. Petersson, H. Nakatsuji, M. Caricato, X. Li, H. P. Hratchian, A. F. Izmaylov, J. Bloino, G. Zheng, J. L. Sonnenberg, M. Hada, M. Ehara, K. Toyota, R. Fukuda, J. Hasegawa, M. Ishida, T. Nakajima, Y. Honda, O. Kitao, H. Nakai, T. Vreven, J. A. Montgomery, J. E. Peralta, Jr., F. Ogliaro, M. Bearpark, J. J. Heyd, E. Brothers, K. N. Kudin, V. N. Staroverov, R. Kobayashi, J. Normand, K. Raghavachari, A. Rendell, J. C. Burant, S. S. Iyengar, J. Tomasi, M. Cossi, N .Rega, J. M. Millam, M. Klene, J. E. Knox, J. B. Cross, V. Bakken, C.

Adamo, J. Jaramillo, R. Gomperts, R. E. Stratmann, O. Yazyev, A. J. Austin, R. Cammi, C. Pomelli, J. W. Ochterski, R. L. Martin, K. Morokuma, V. G. Zakrzewski, G. A. Voth, P. Salvador, J. J. Dannenberg, S. Dapprich, A. D. Daniels, O. Farkas, J. B. Foresman, J. V. Ortiz, J. Cioslowski and D. J. Fox, *Gaussian 09, Revision A.01*, Gaussian, Inc., Wallingford CT, 2009.

(8) (a) Y. Zhao and D. G. Truhlar, *Acc. Chem. Res.*, 2008, **41**, 157; (b) Y. Zhao and D. G. Truhlar, *Theor. Chem. Acc.*, 2008, **120**, 215.

(9) (a) A. V. Marenich, C. J. Cramer and D. G. Truhlar, *J. Phys. Chem. B*, 2009, **113**, 6378; (b) R. F. Ribeiro, A. V. Marenich, C. J. Cramer and D. G. Truhlar, *J. Phys. Chem. B*, 2011, **115**, 14556.

NMR spectra:

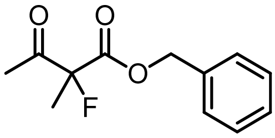

3a

^{1}H NMR (CDCl$_3$, 400 MHz)

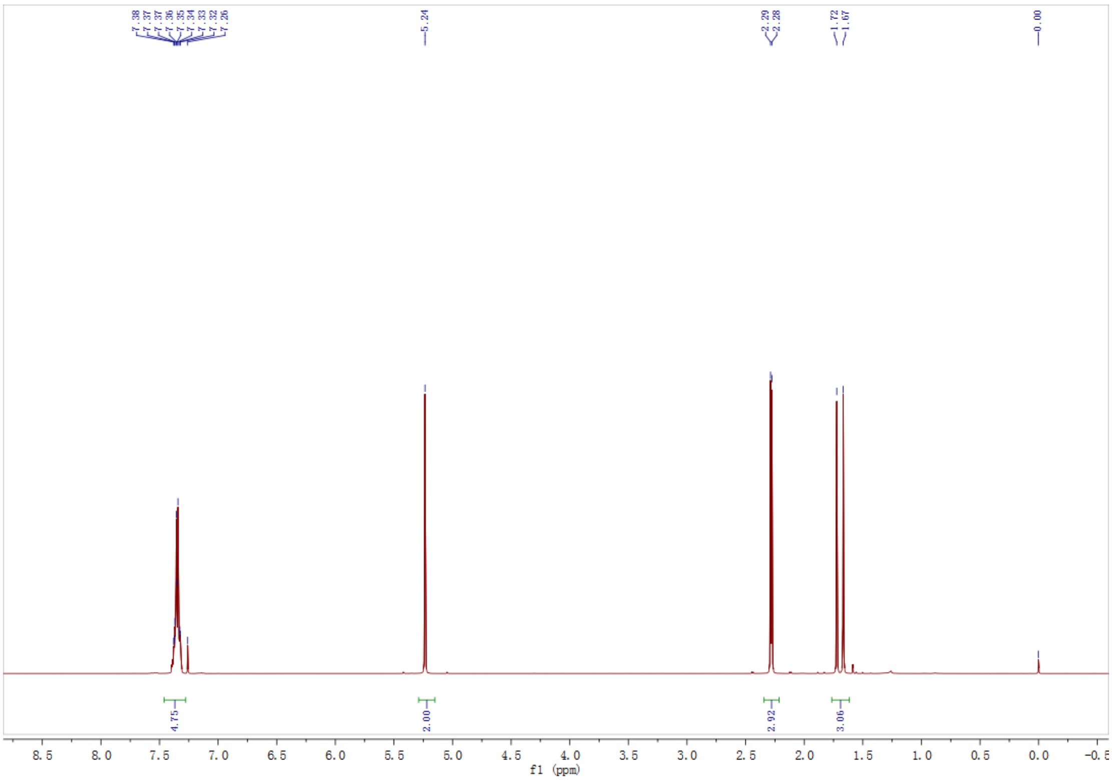

^{13}C NMR ($CDCl_3$, 101 MHz)

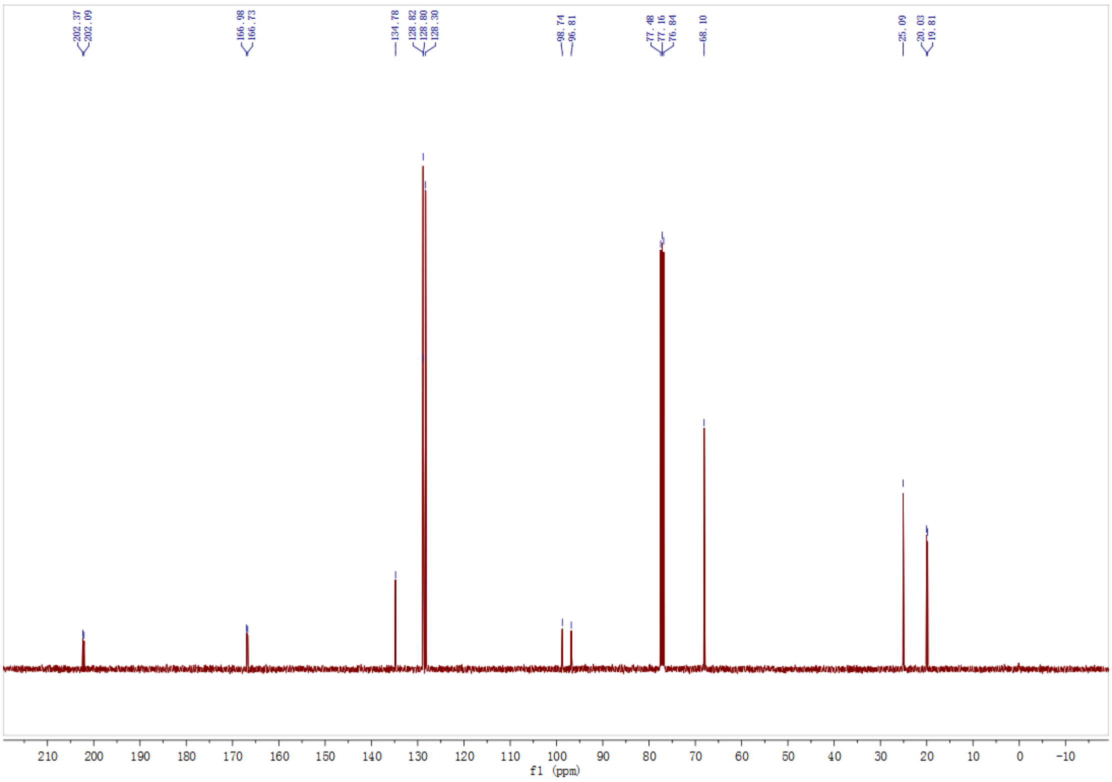

^{19}F NMR ($CDCl_3$, 471 MHz)

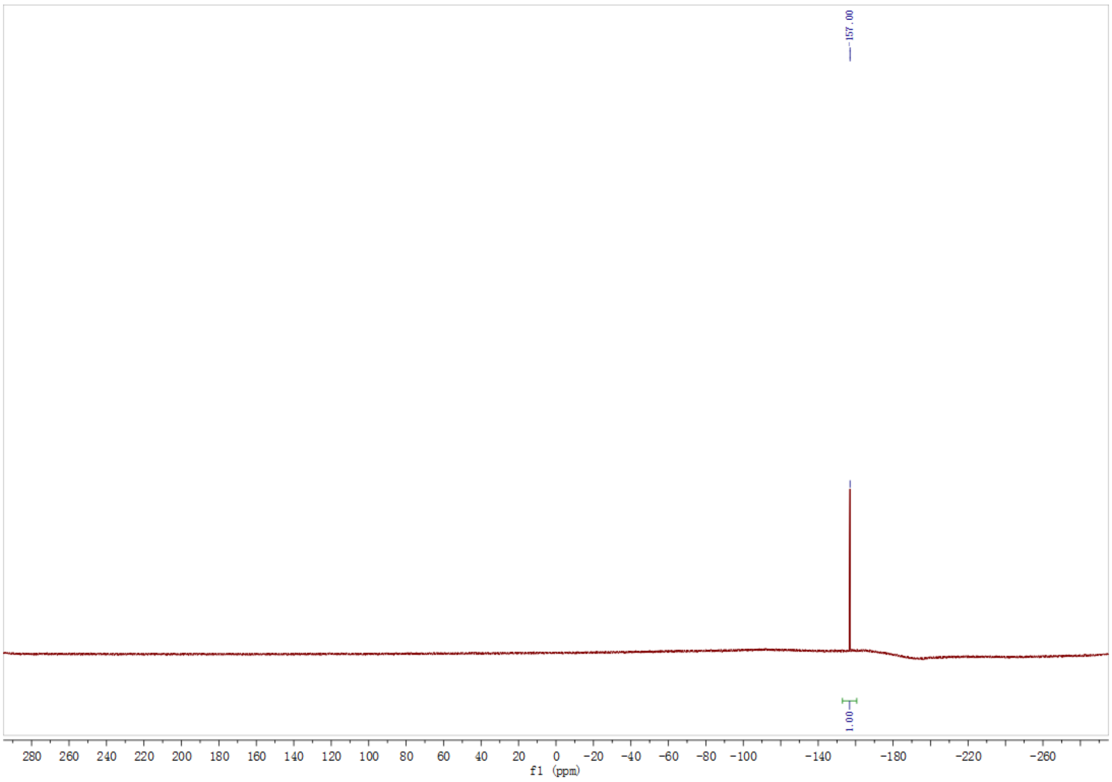

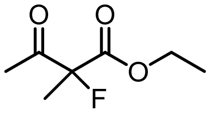

3b

^{1}H NMR ($CDCl_3$, 400 MHz)

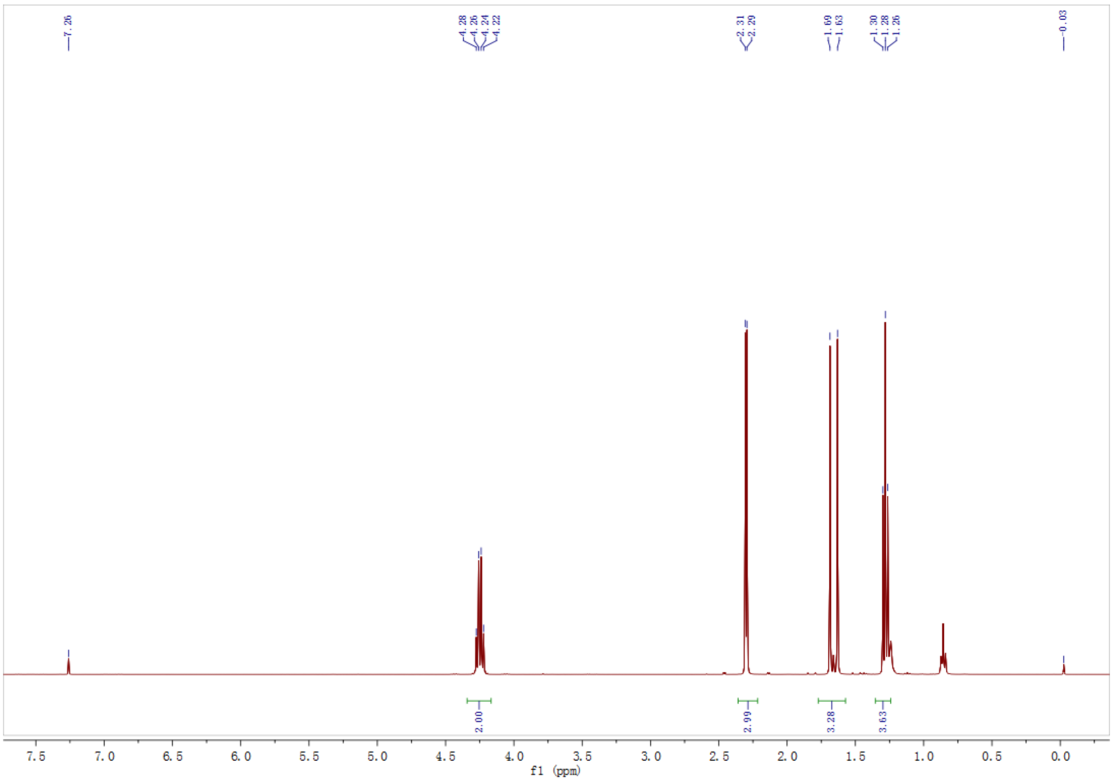

^{13}C NMR ($CDCl_3$, 101 MHz)

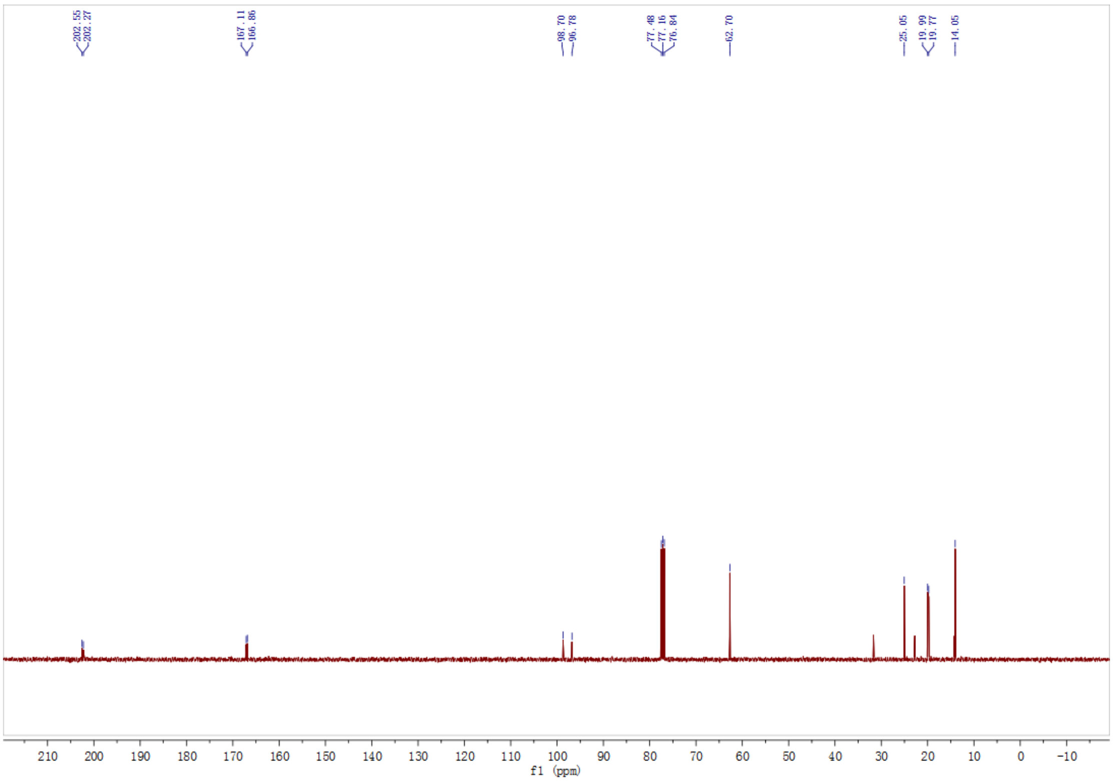

^{19}F NMR ($CDCl_3$, 565 MHz)

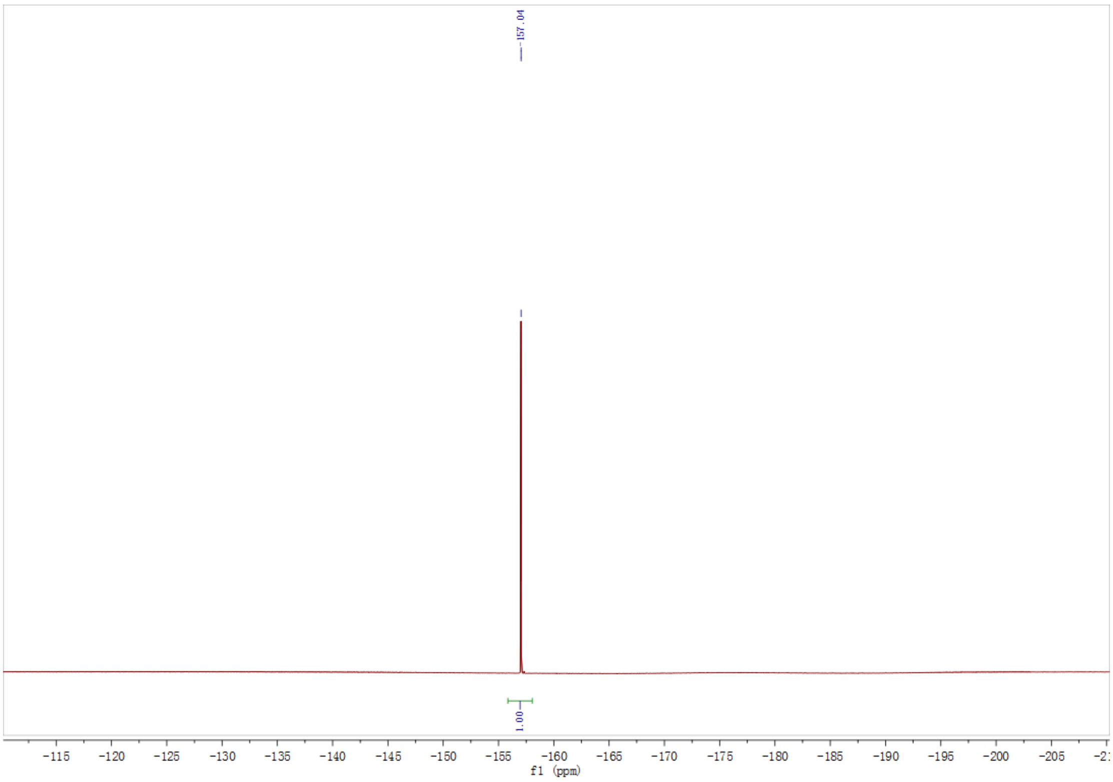

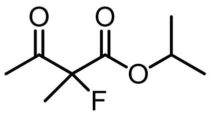

3c

^{1}H NMR (CDCl$_3$, 400 MHz)

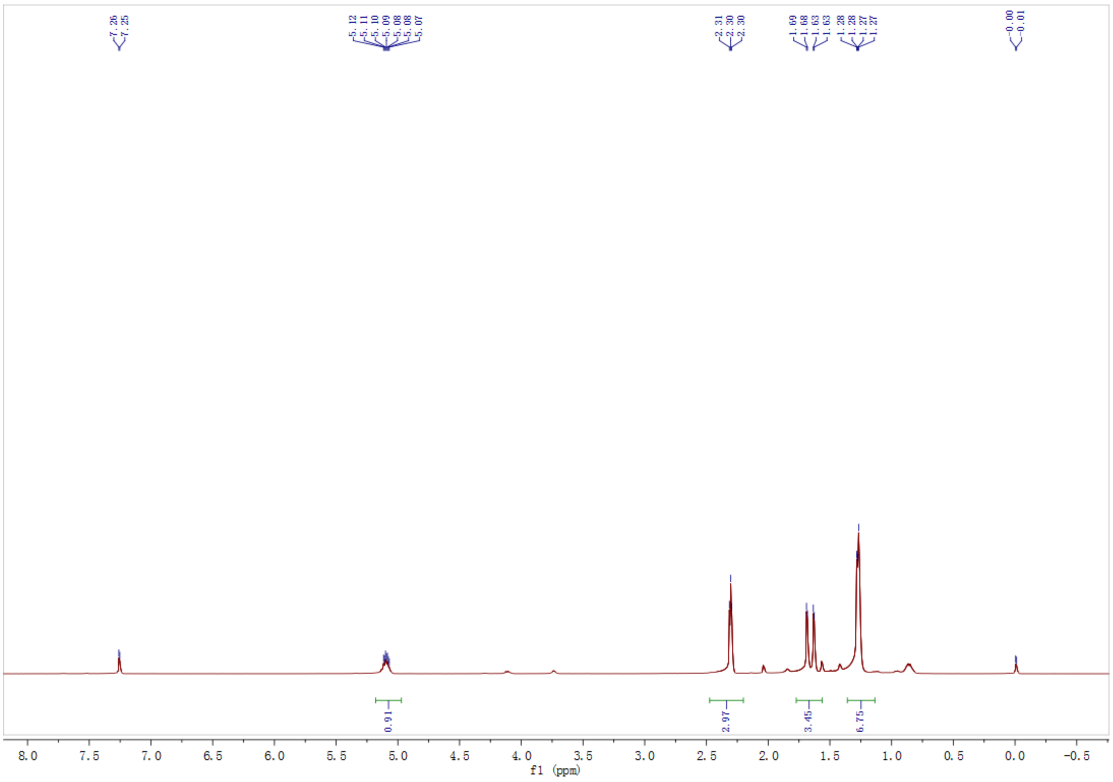

^{13}C NMR ($CDCl_3$, 101 MHz)

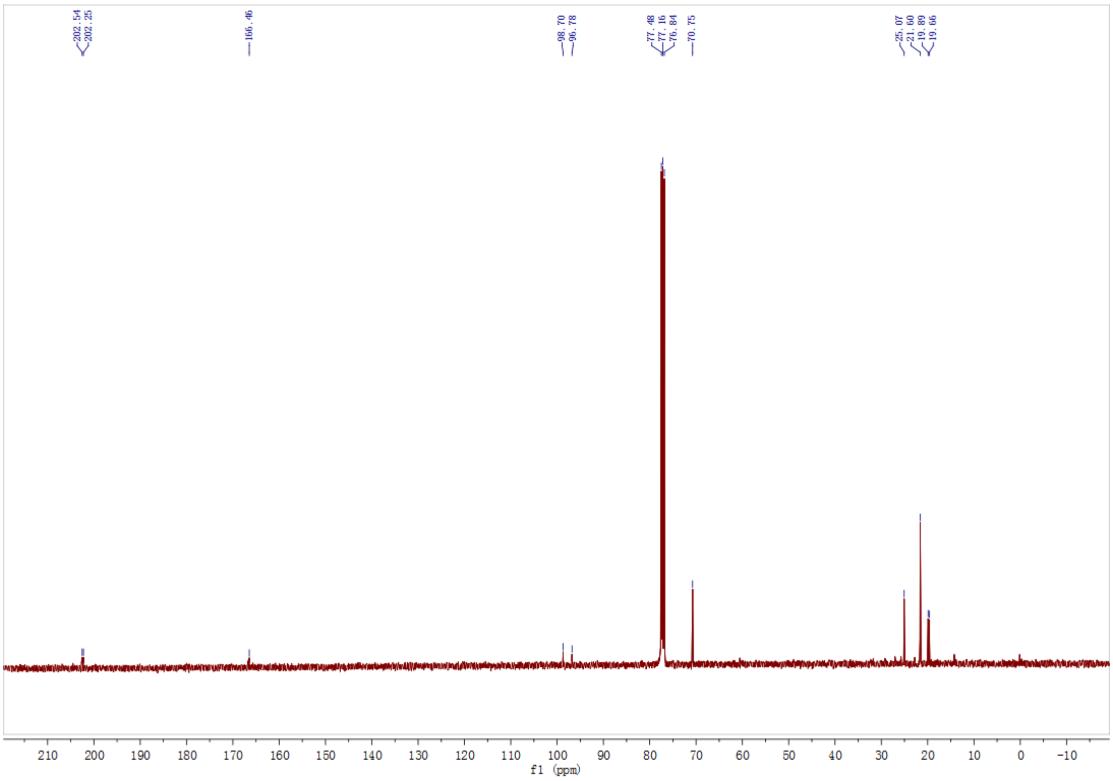

^{19}F NMR ($CDCl_3$, 565 MHz)

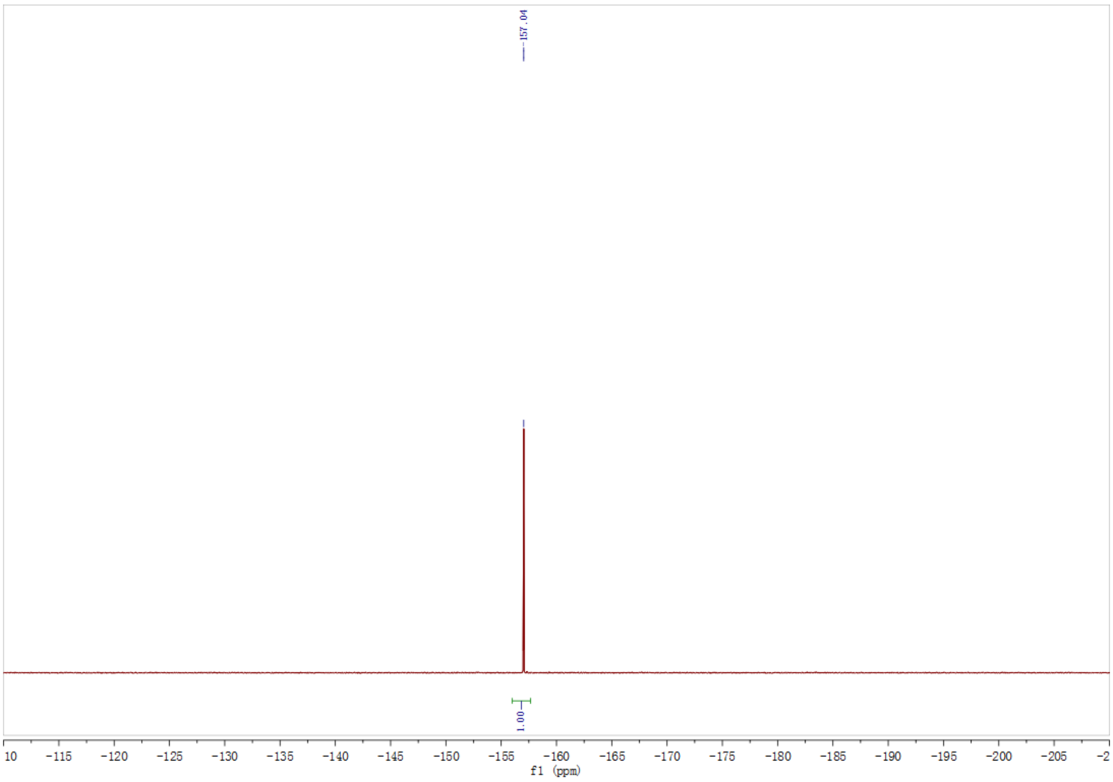

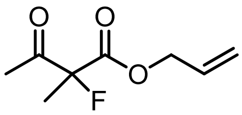

3d

^{1}H NMR (CDCl$_3$, 400 MHz)

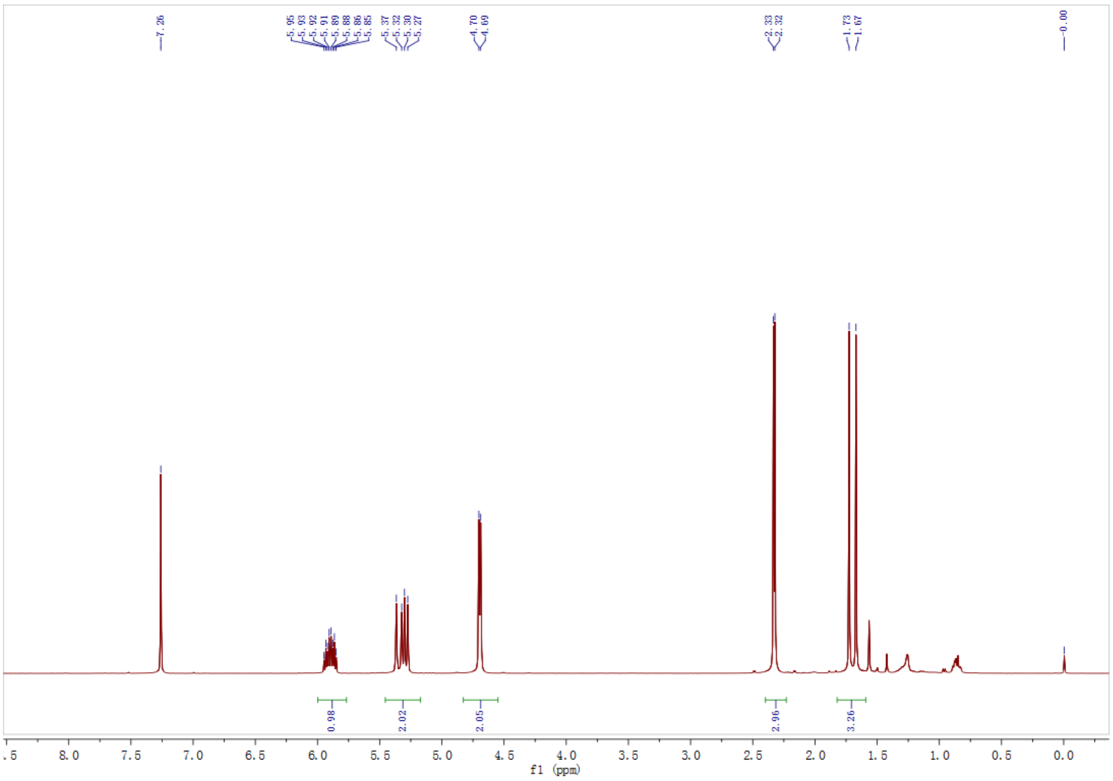

^{13}C NMR ($CDCl_3$, 101 MHz)

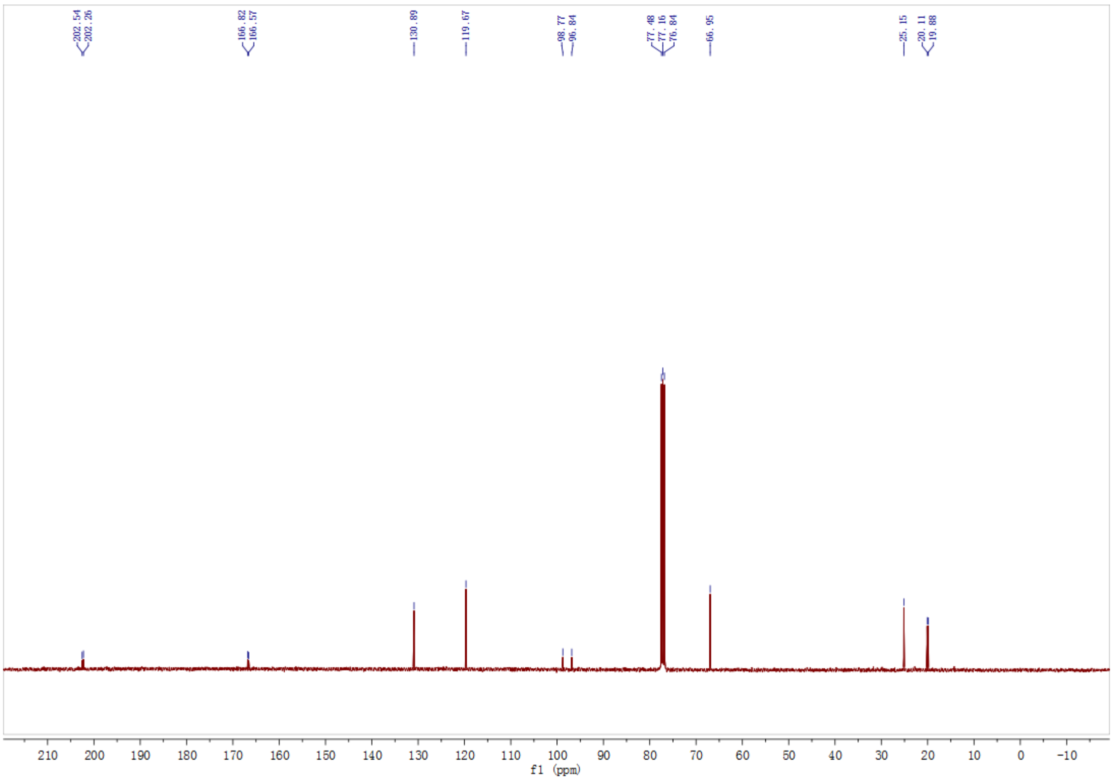

^{19}F NMR ($CDCl_3$, 565 MHz)

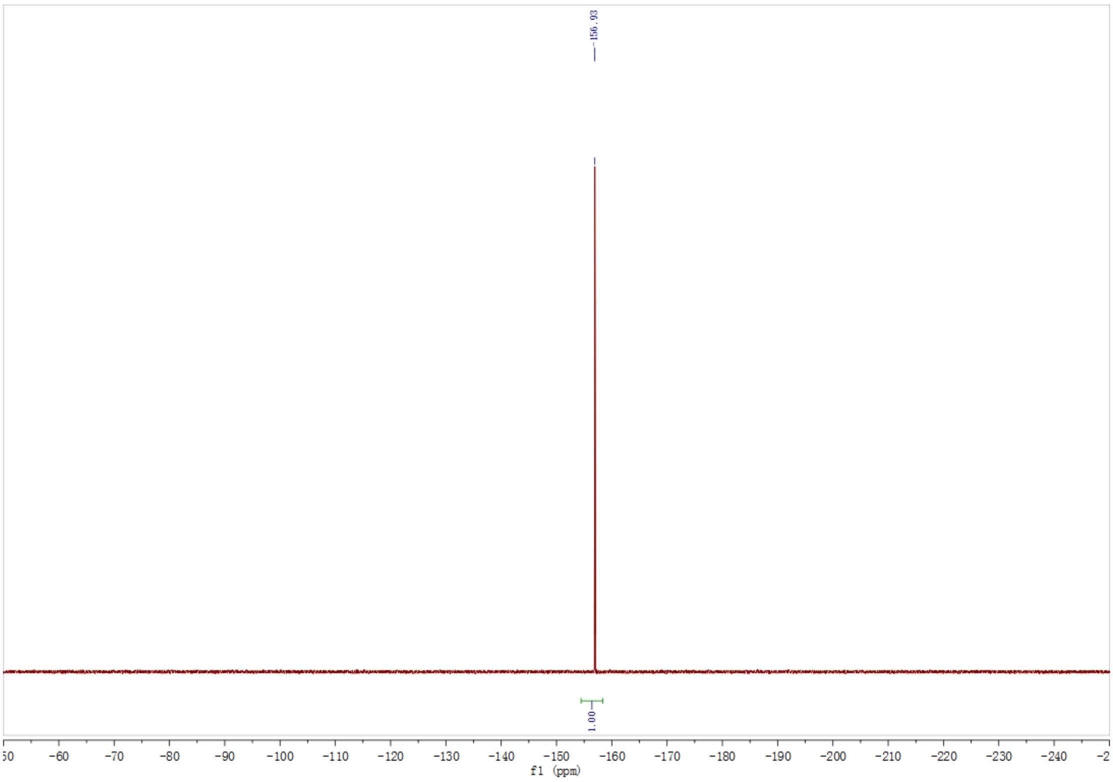

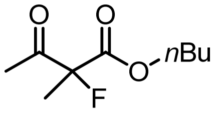

3e

^{1}H NMR (CDCl$_3$, 400 MHz)

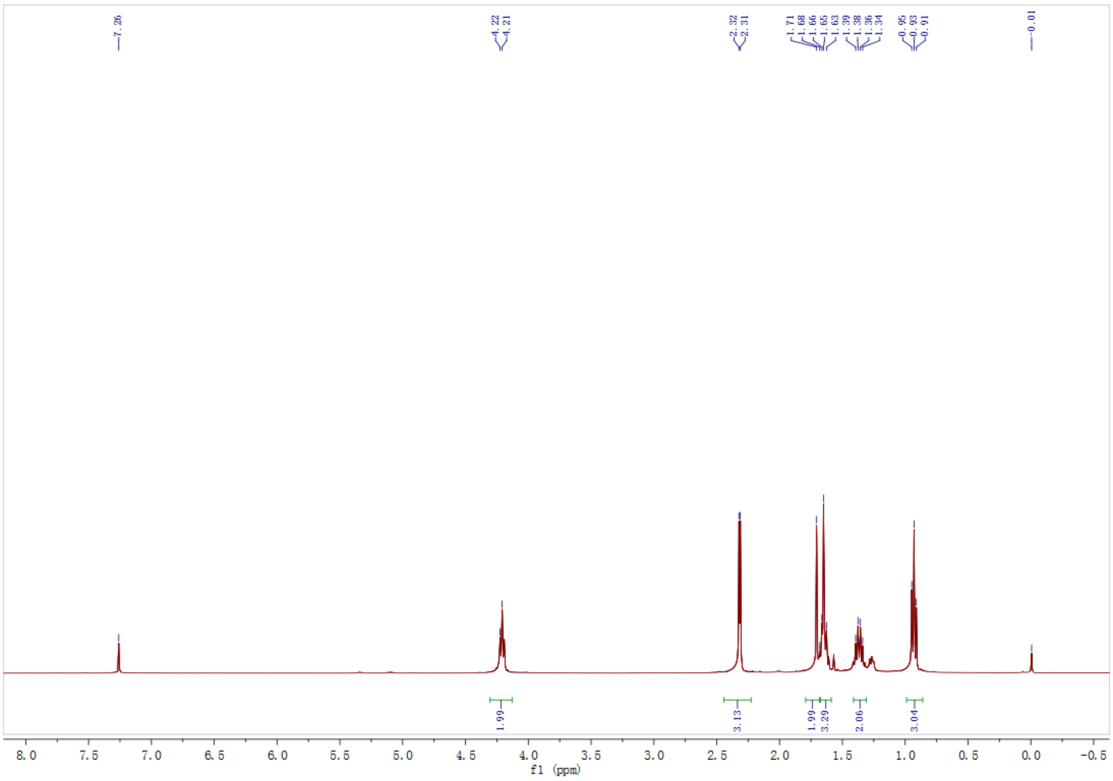

^{13}C NMR ($CDCl_3$, 101 MHz)

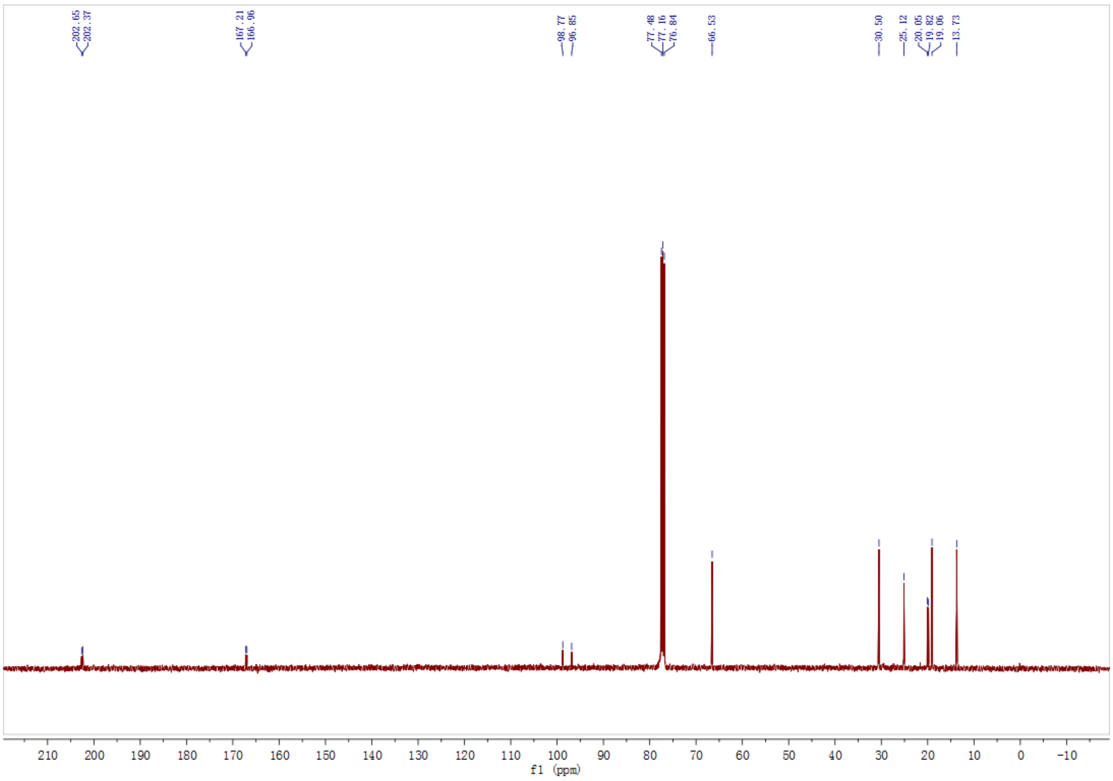

^{19}F NMR ($CDCl_3$, 565MHz)

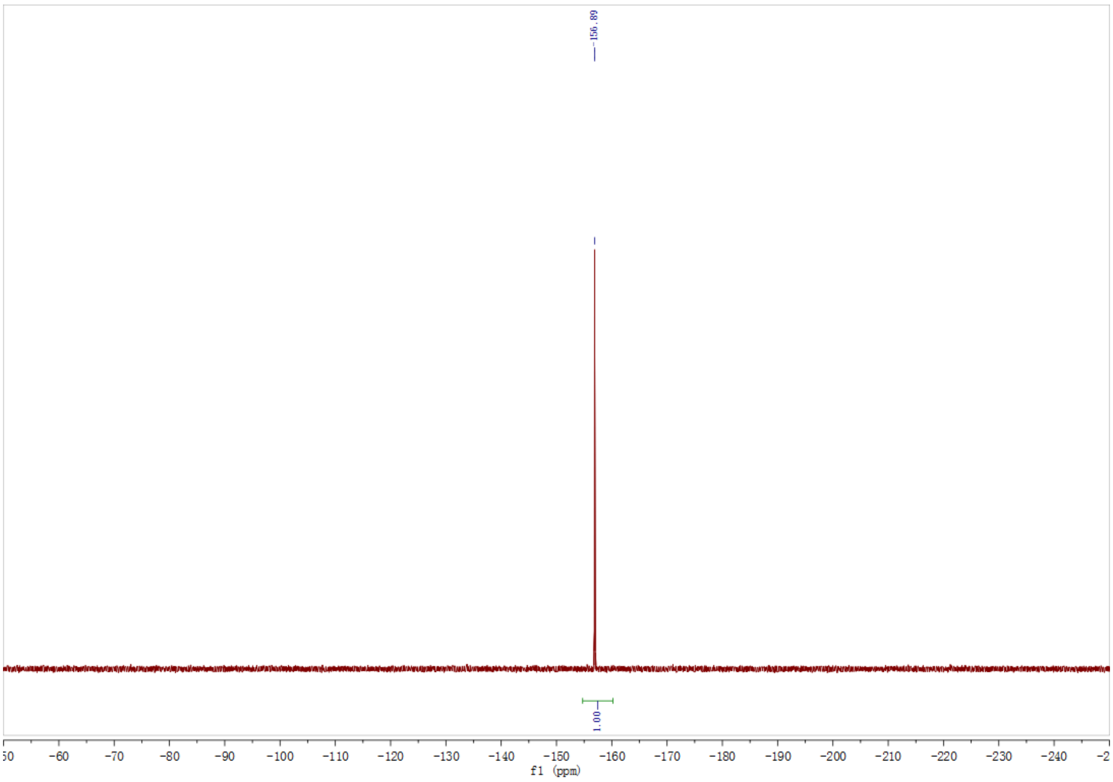

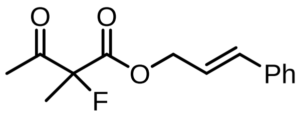

3f

^{1}H NMR ($CDCl_3$, 400 MHz)

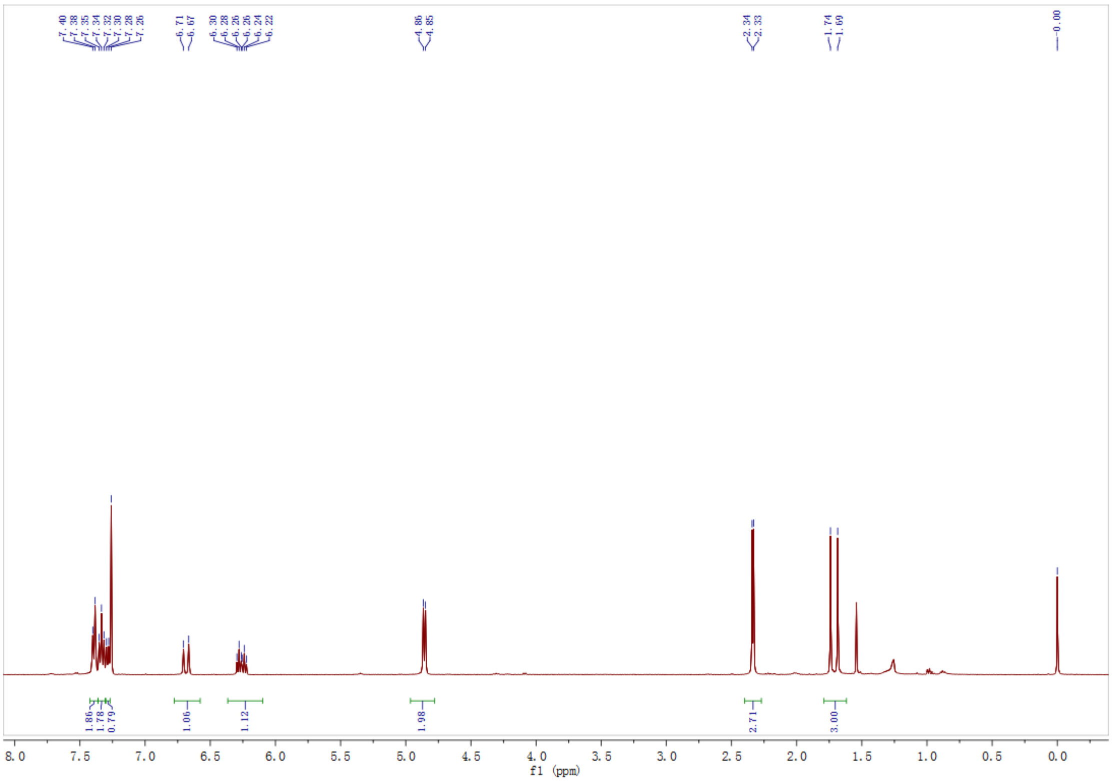

^{13}C NMR ($CDCl_3$, 101 MHz)

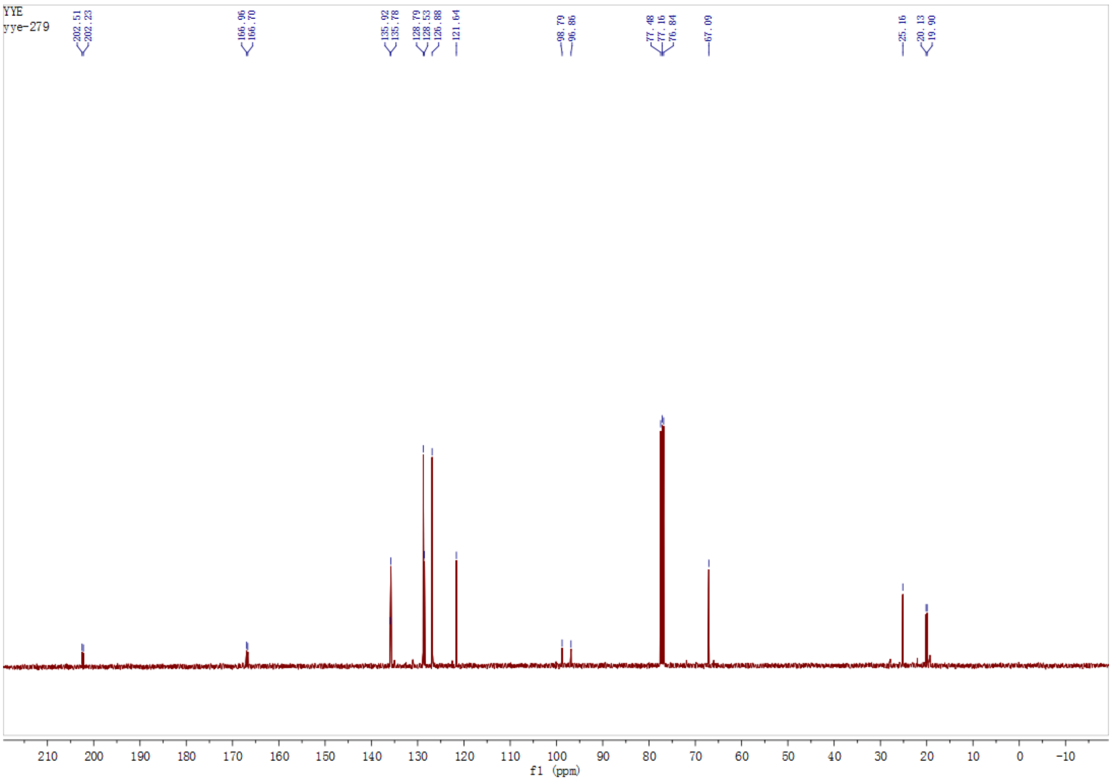

^{19}F NMR ($CDCl_3$, 565 MHz)

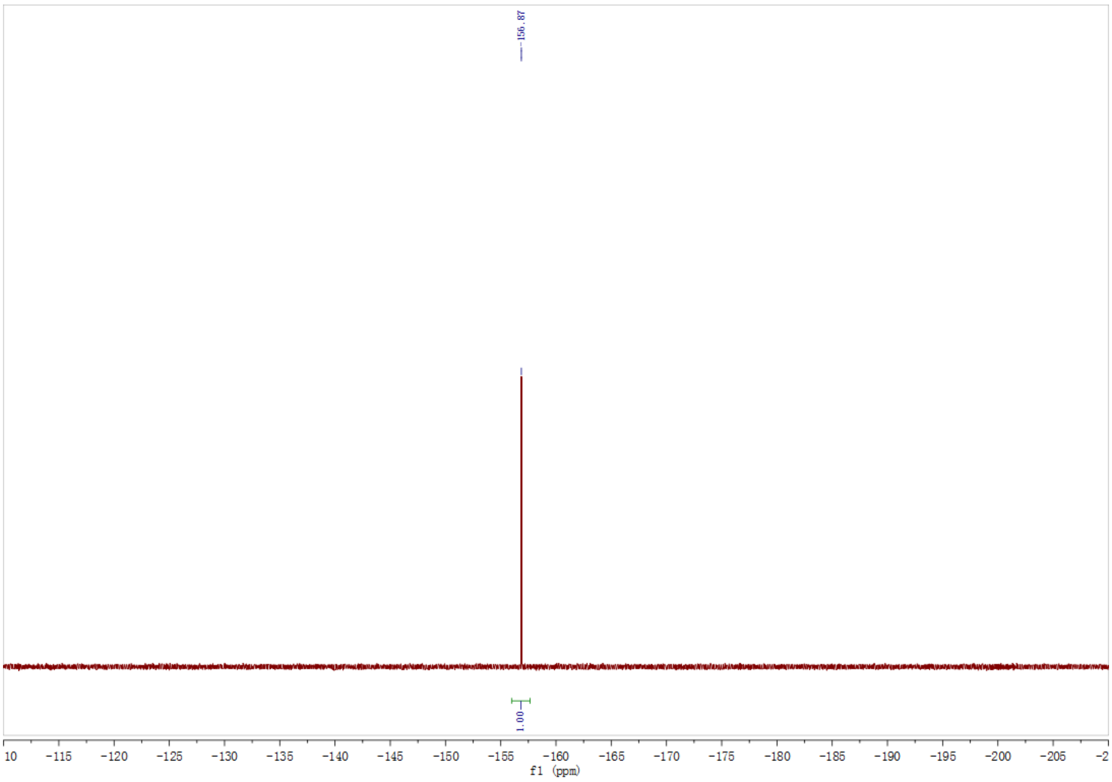

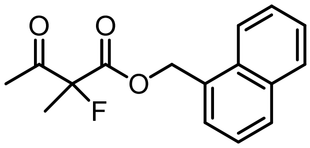

3g

^{1}H NMR (CDCl$_3$, 400 MHz)

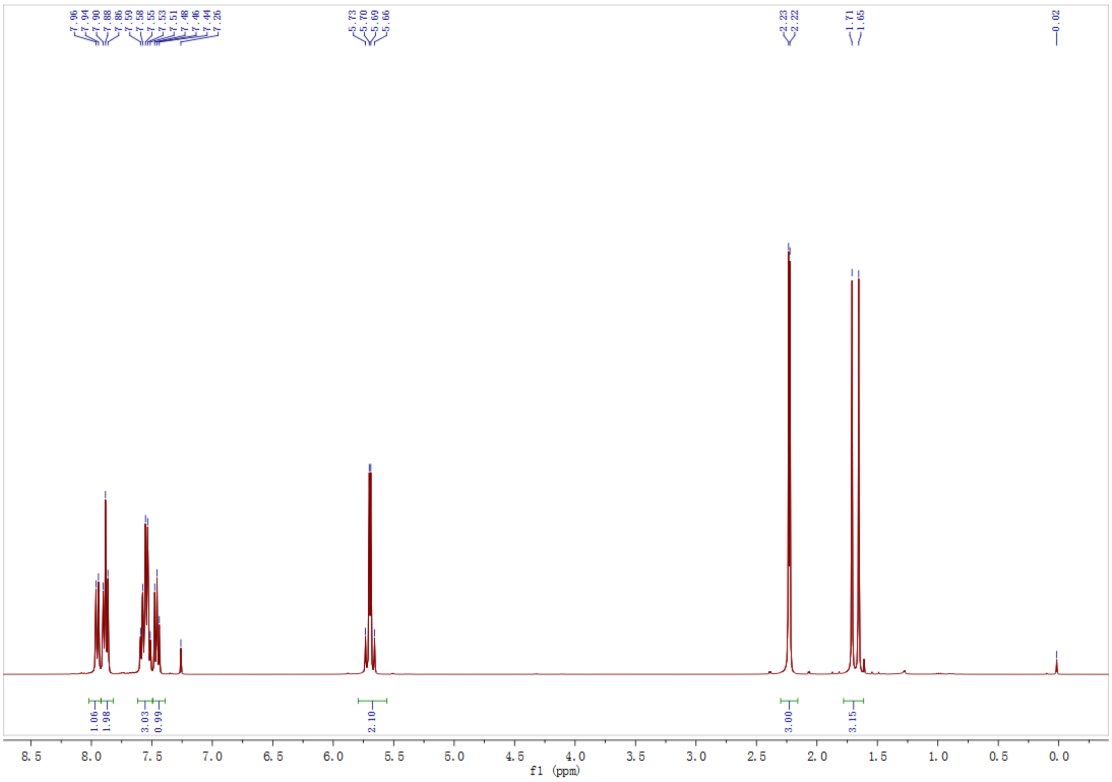

^{13}C NMR ($CDCl_3$, 101 MHz)

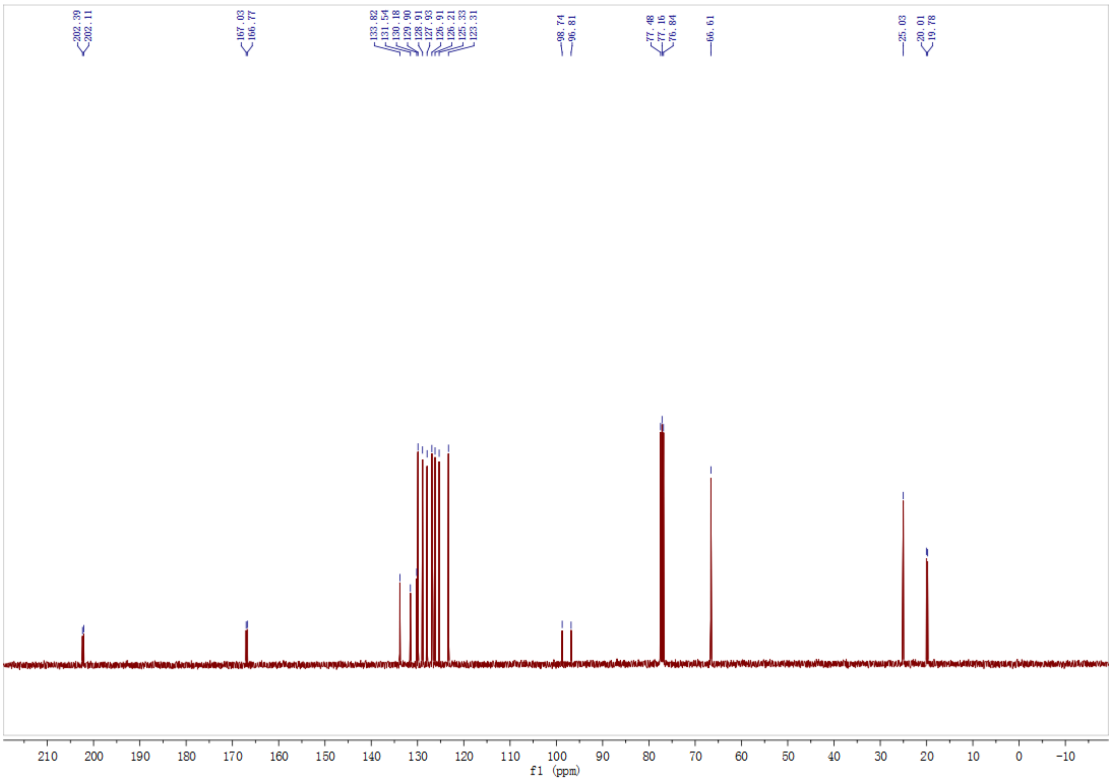

^{19}F NMR ($CDCl_3$, 565 MHz)

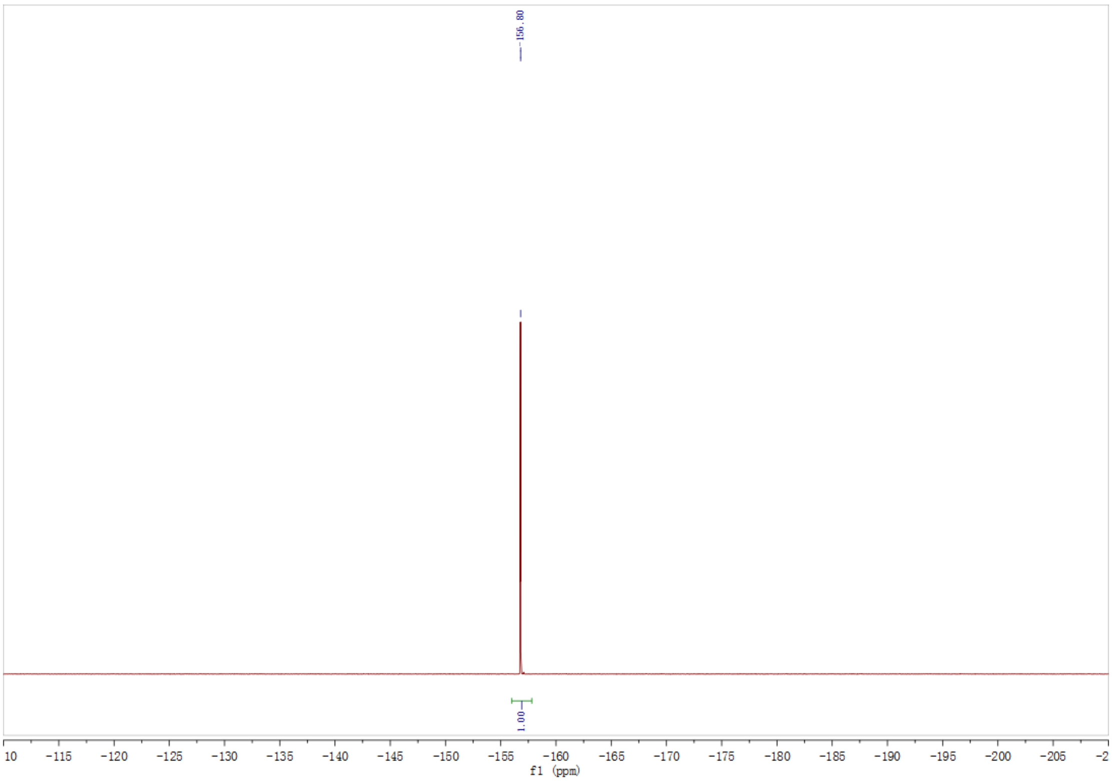

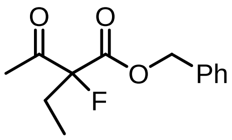

3h

^{1}H NMR ($CDCl_3$, 400 MHz)

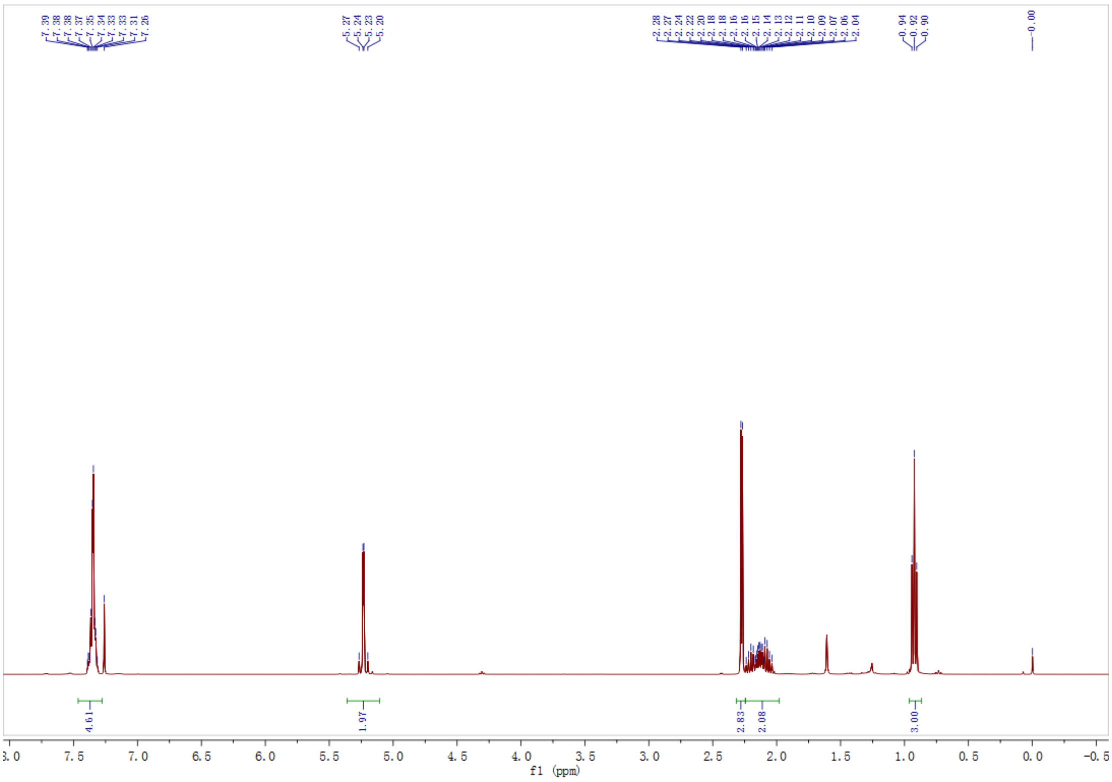

^{13}C NMR ($CDCl_3$, 101 MHz)

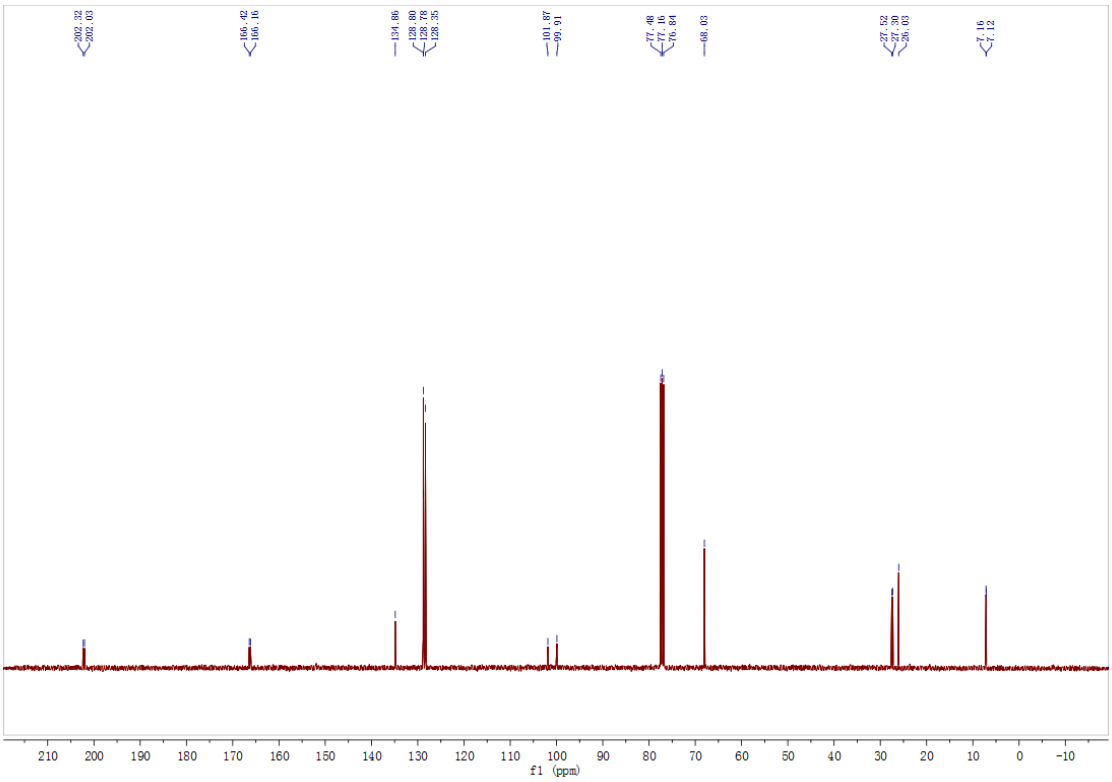

^{19}F NMR ($CDCl_3$, 471 MHz)

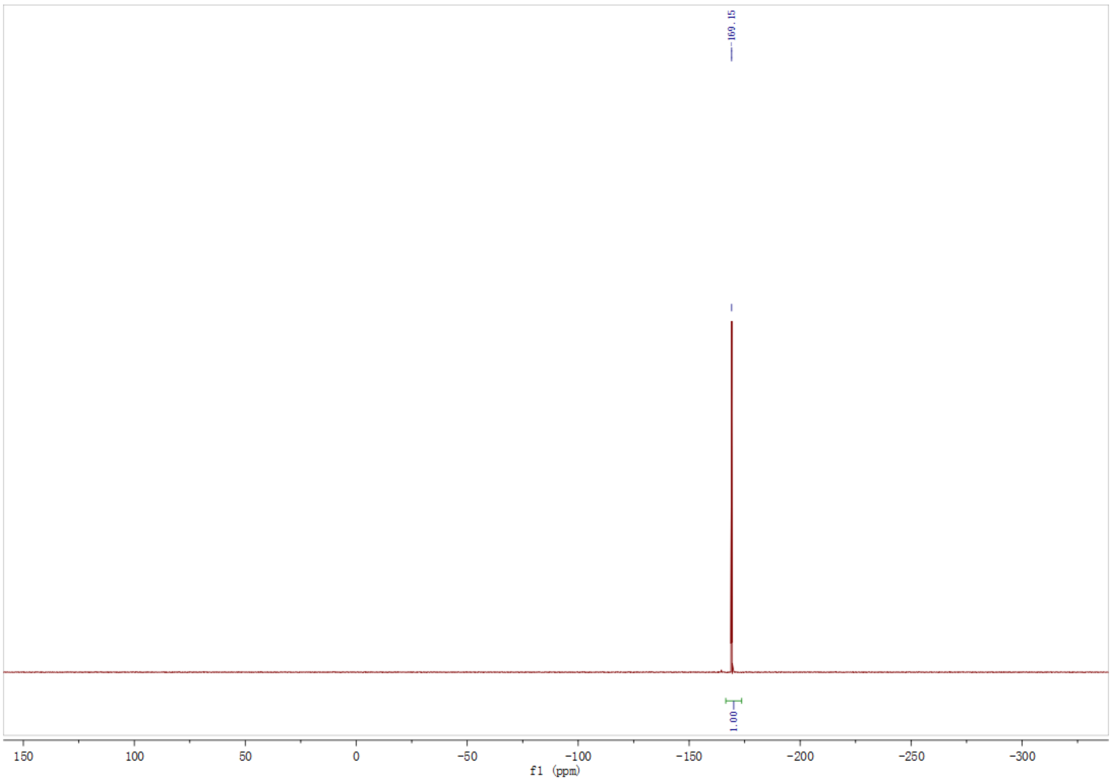

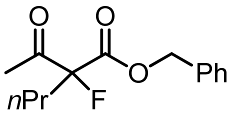

3i

^{1}H NMR ($CDCl_3$, 400 MHz)

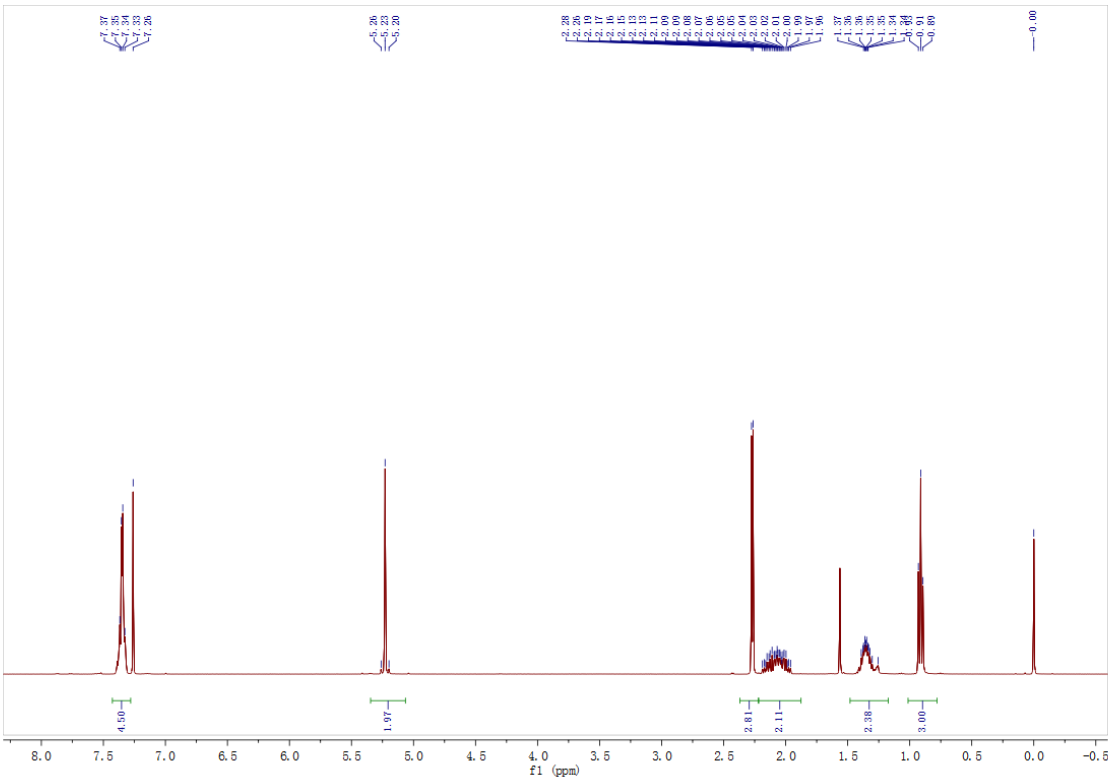

^{13}C NMR (CDCl$_3$, 101 MHz)

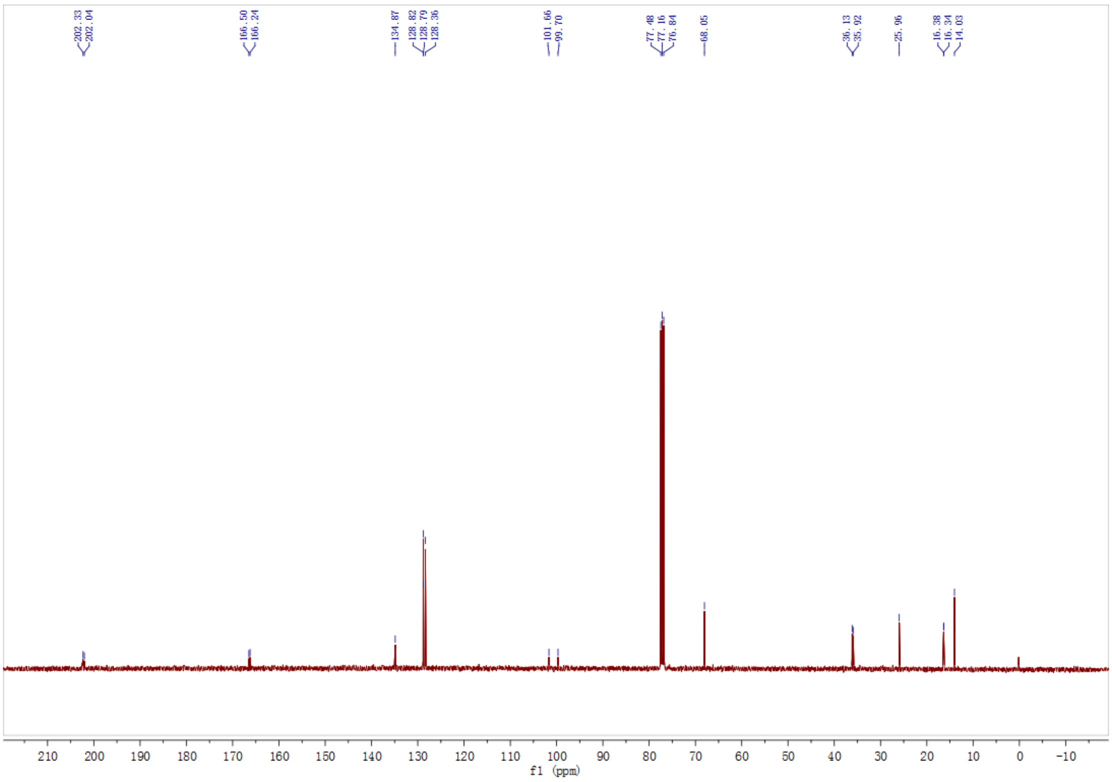

^{19}F NMR (CDCl$_3$, 471 MHz)

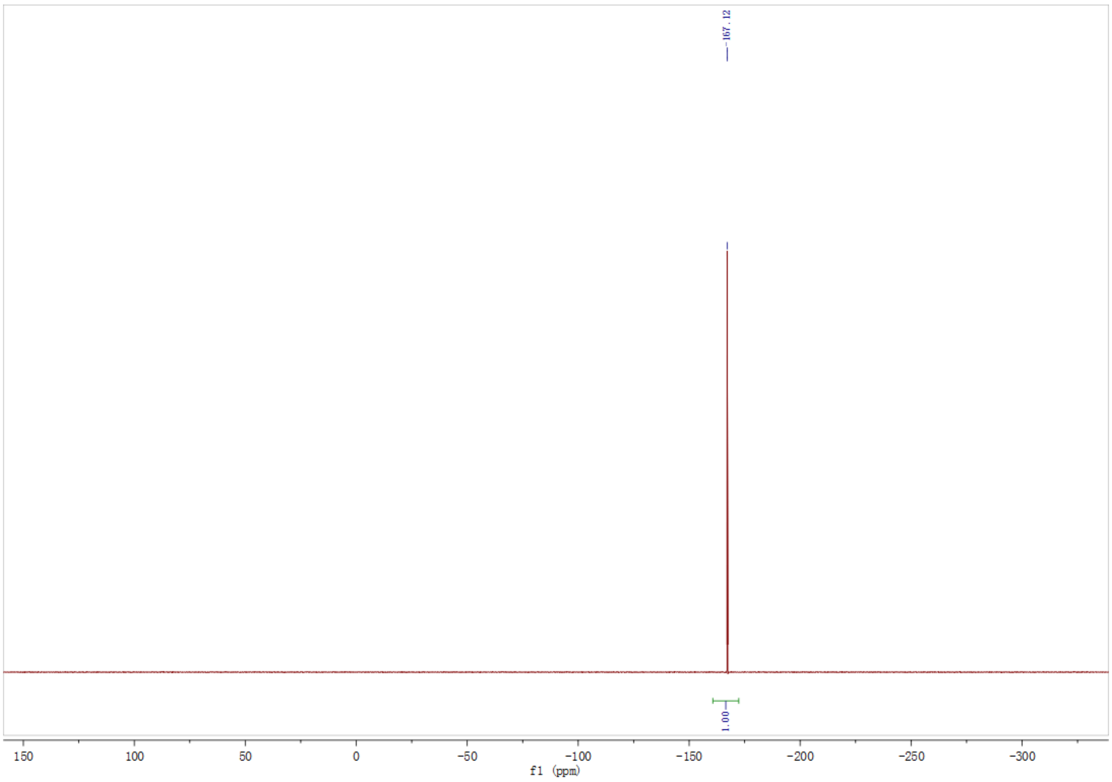

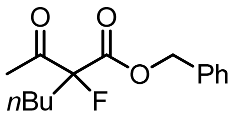

3j

^{1}H NMR ($CDCl_3$, 400 MHz)

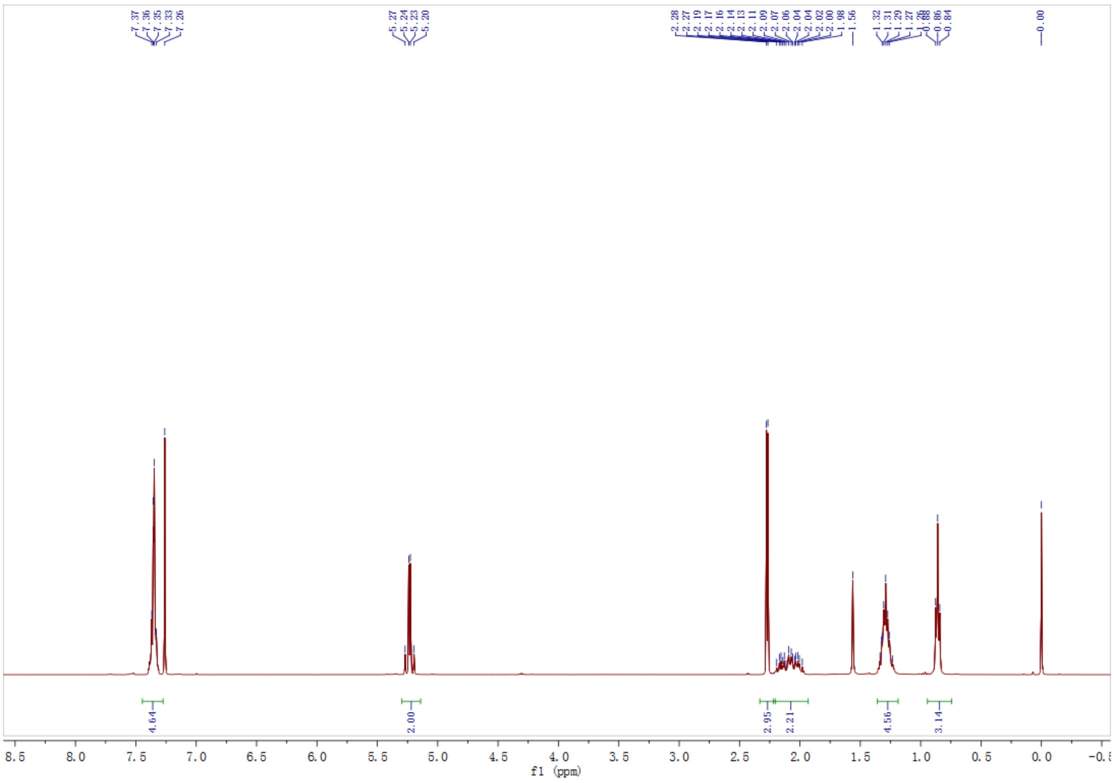

^{13}C NMR ($CDCl_3$, 101 MHz)

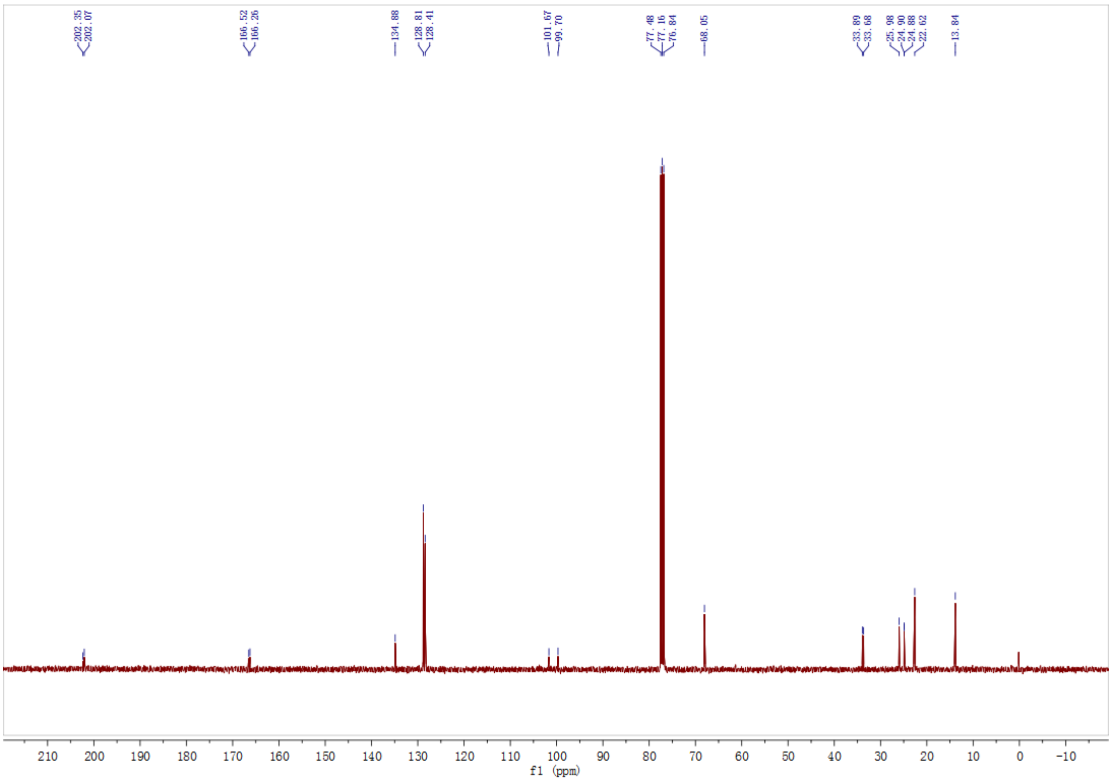

^{19}F NMR ($CDCl_3$, 471 MHz)

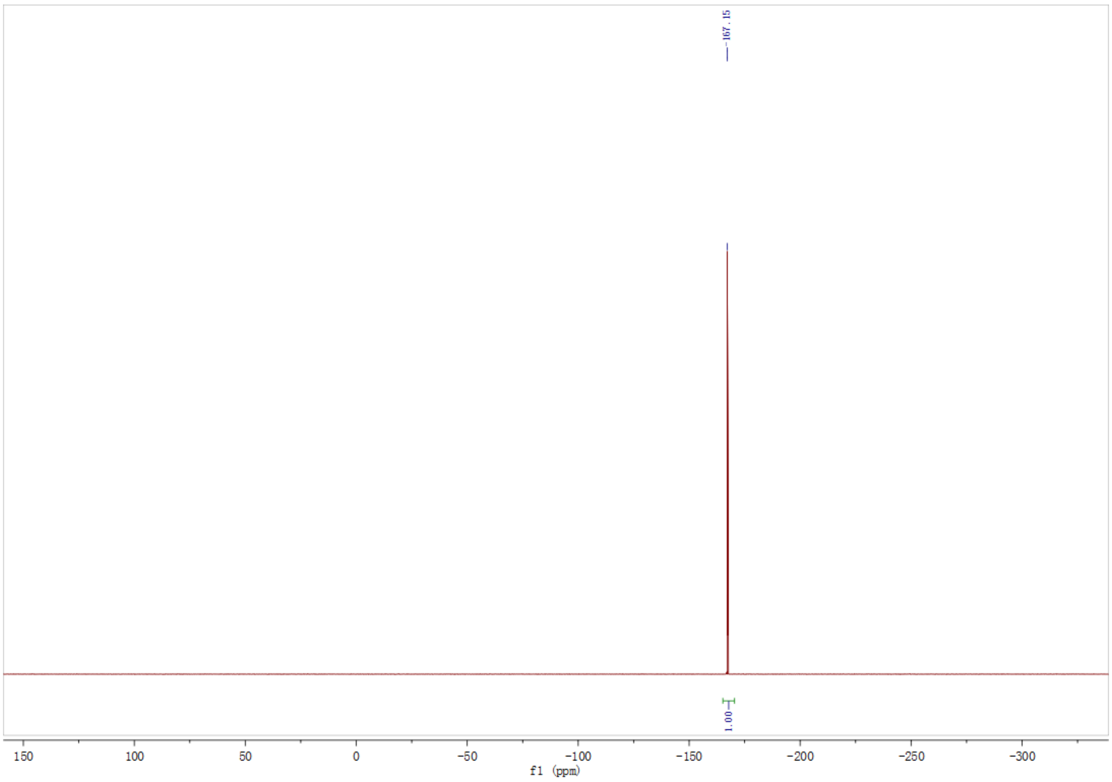

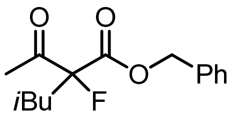

3k

^{1}H NMR (CDCl$_3$, 500 MHz)

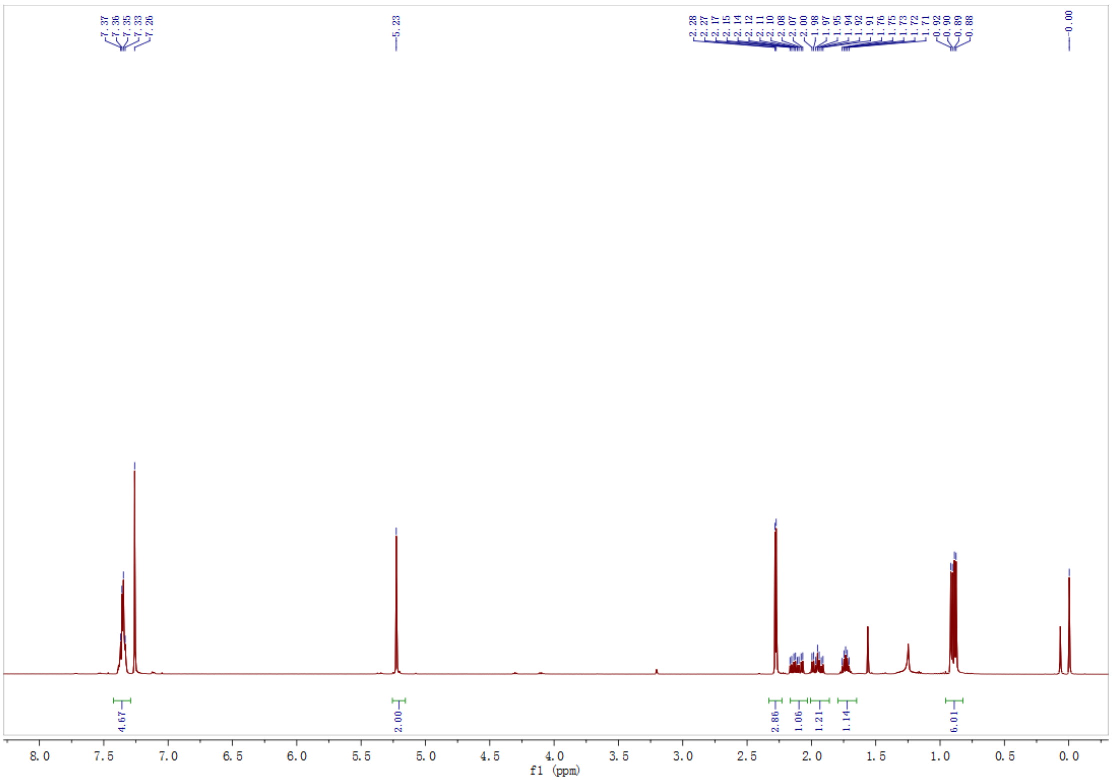

^{13}C NMR (CDCl$_3$, 126 MHz)

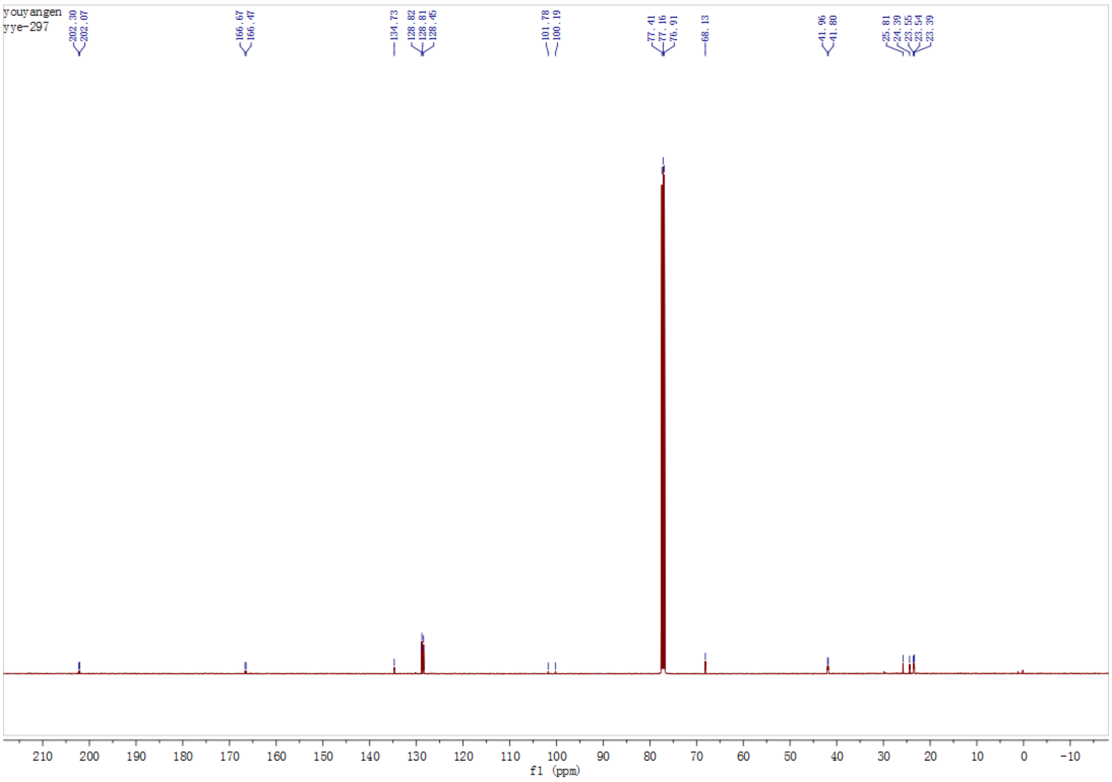

^{19}F NMR (CDCl$_3$, 565 MHz)

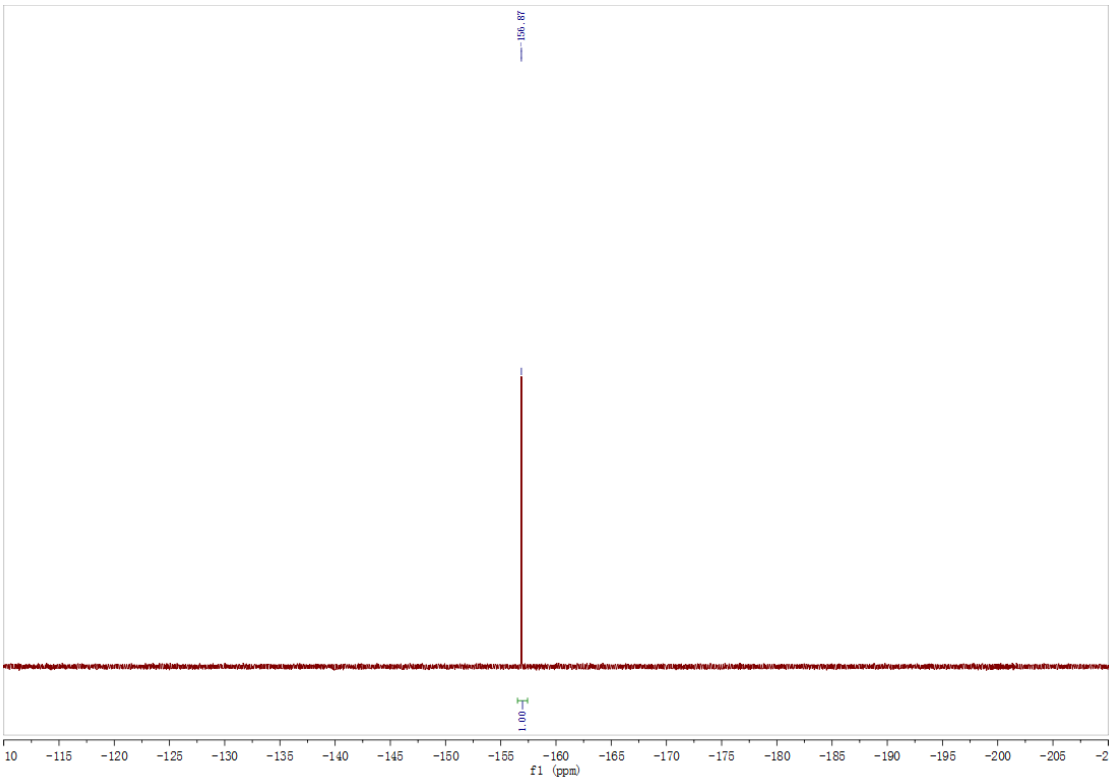

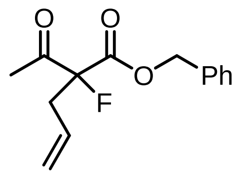

3l

^{1}H NMR (CDCl$_3$, 400 MHz)

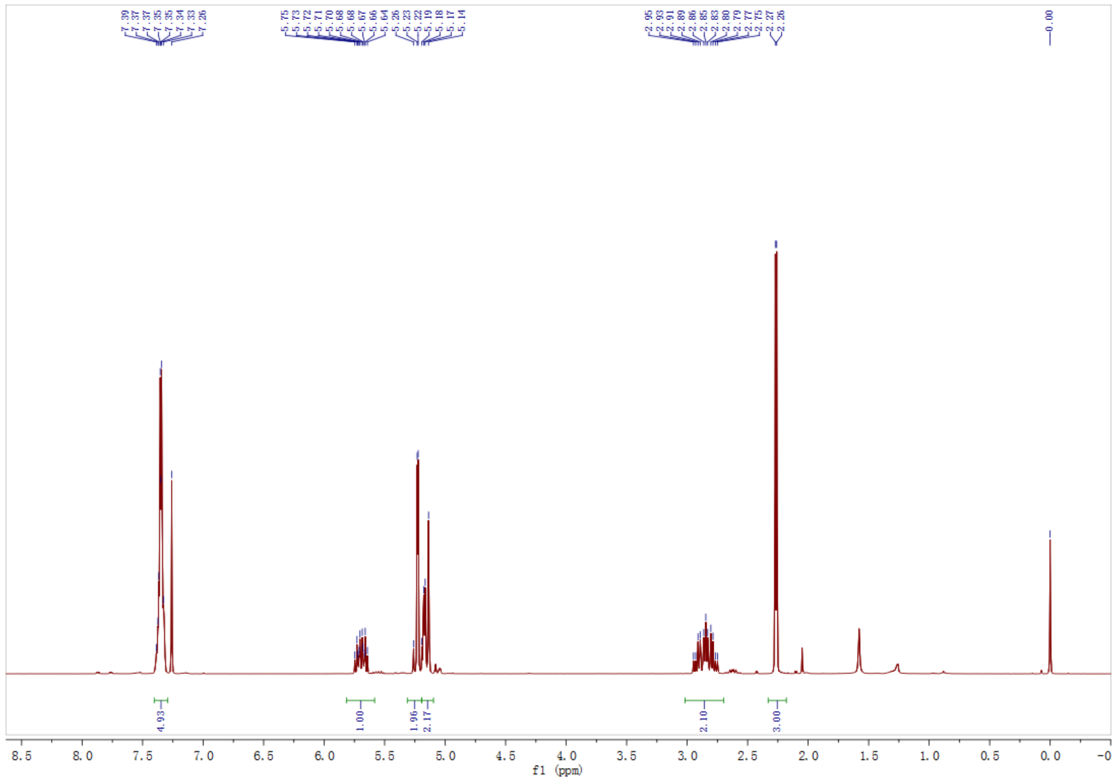

^{13}C NMR ($CDCl_3$, 101 MHz)

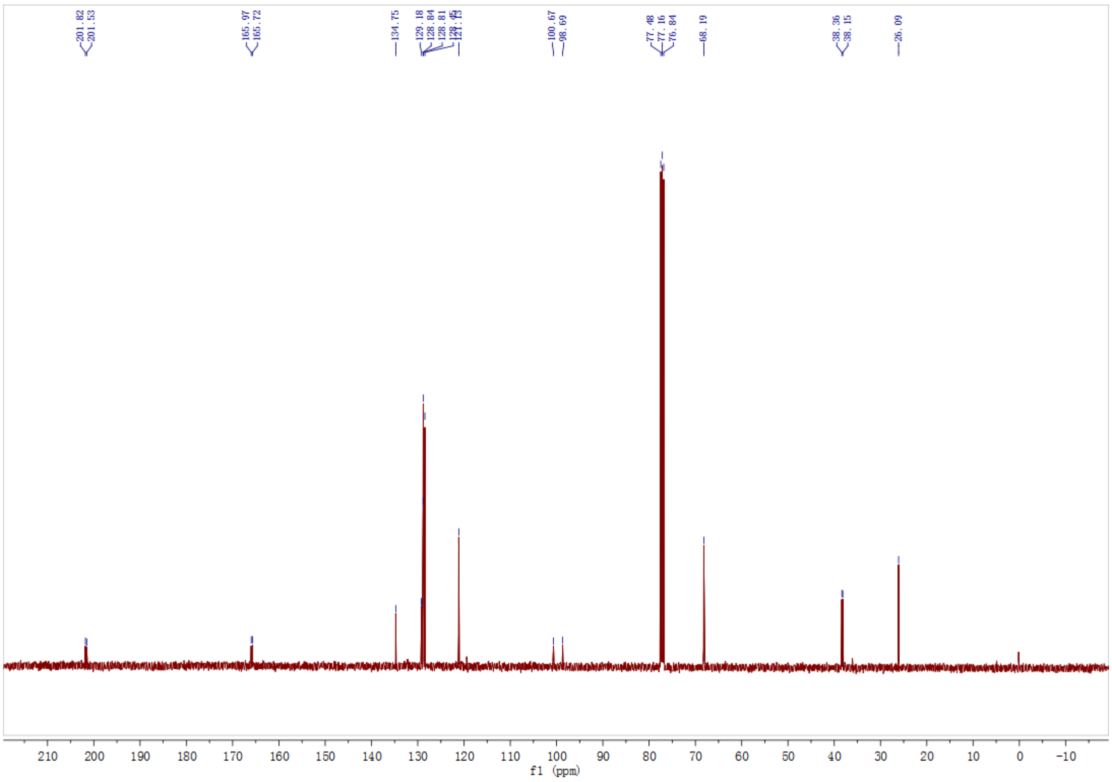

^{19}F NMR ($CDCl_3$, 565 MHz)

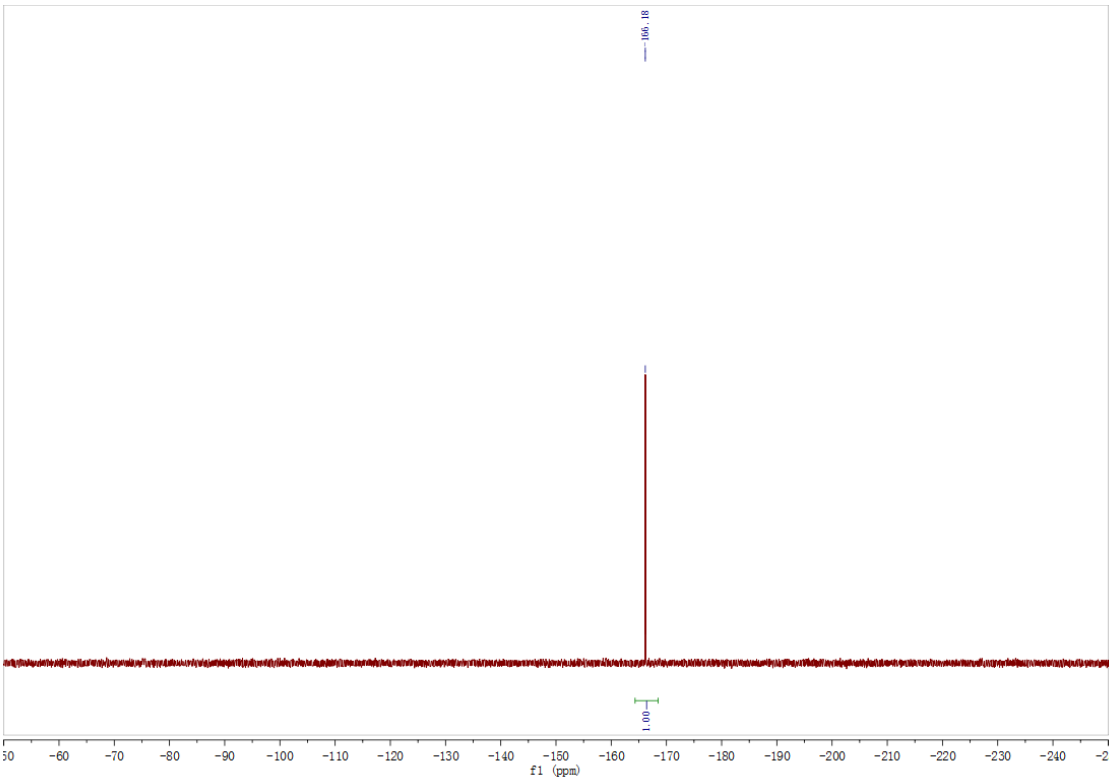

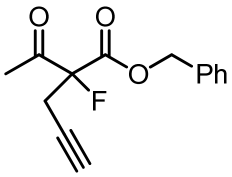

3m

^{1}H NMR ($CDCl_3$, 400 MHz)

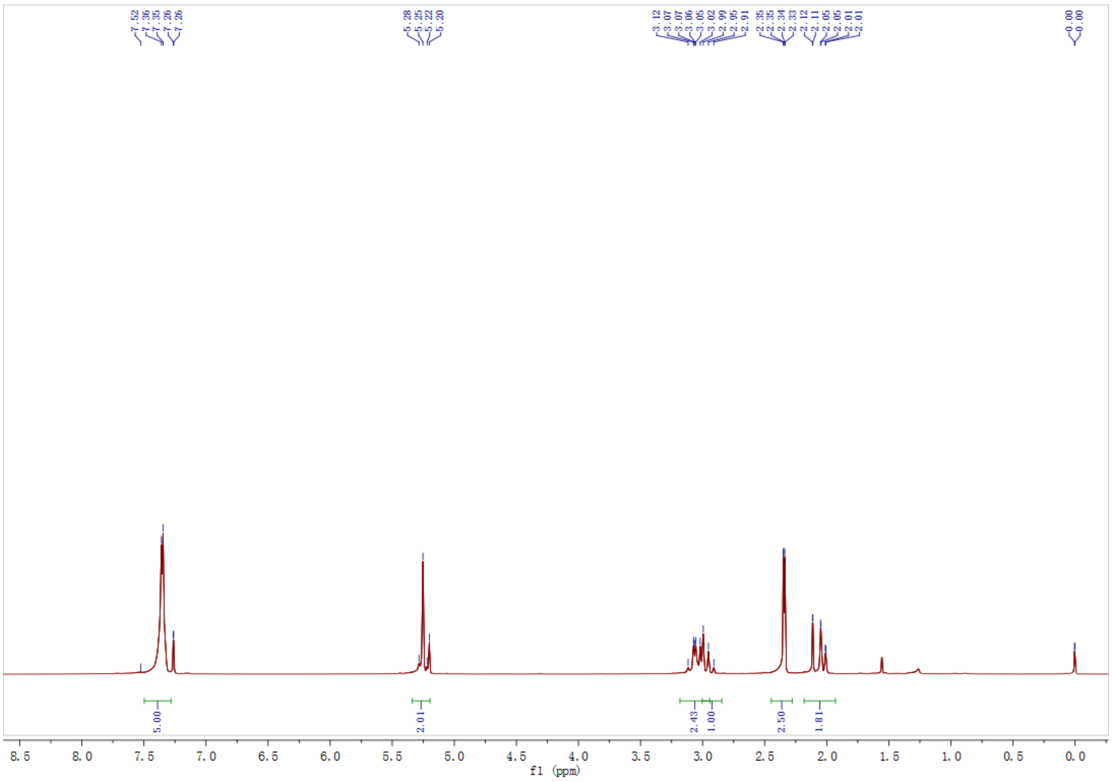

^{13}C NMR ($CDCl_3$, 101 MHz)

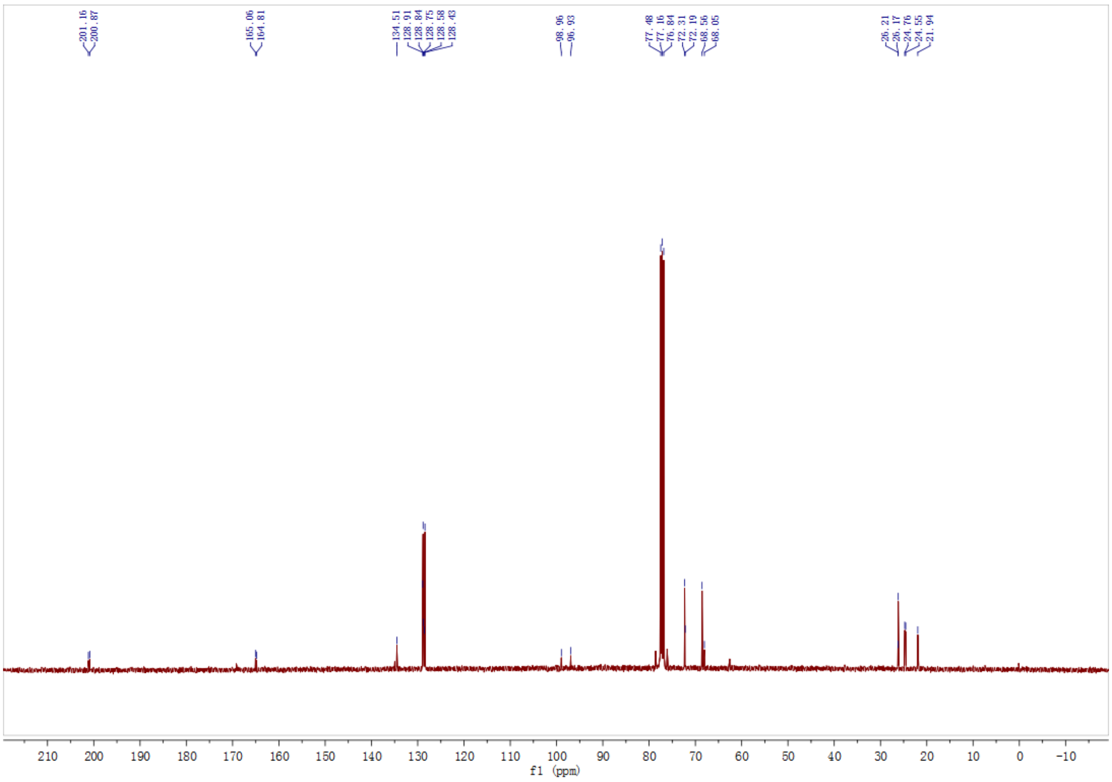

^{19}F NMR ($CDCl_3$, 565 MHz)

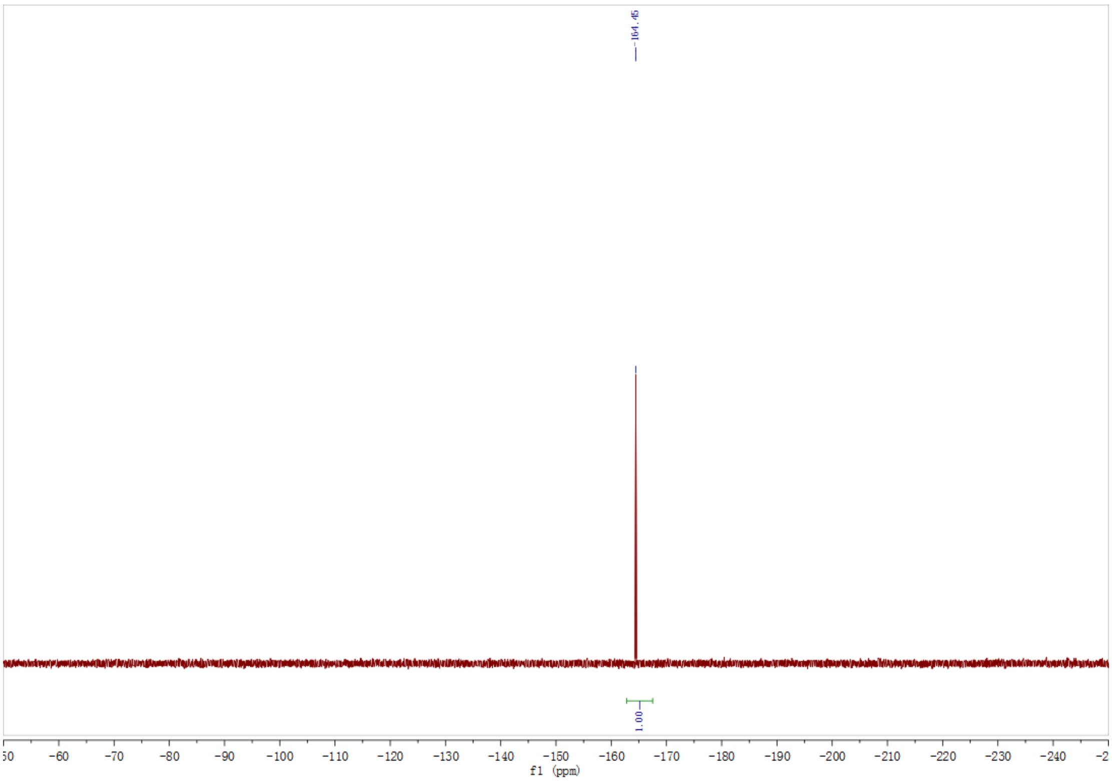

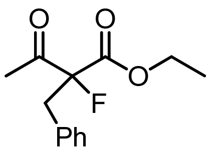

3n

^{1}H NMR (CDCl$_3$, 400 MHz)

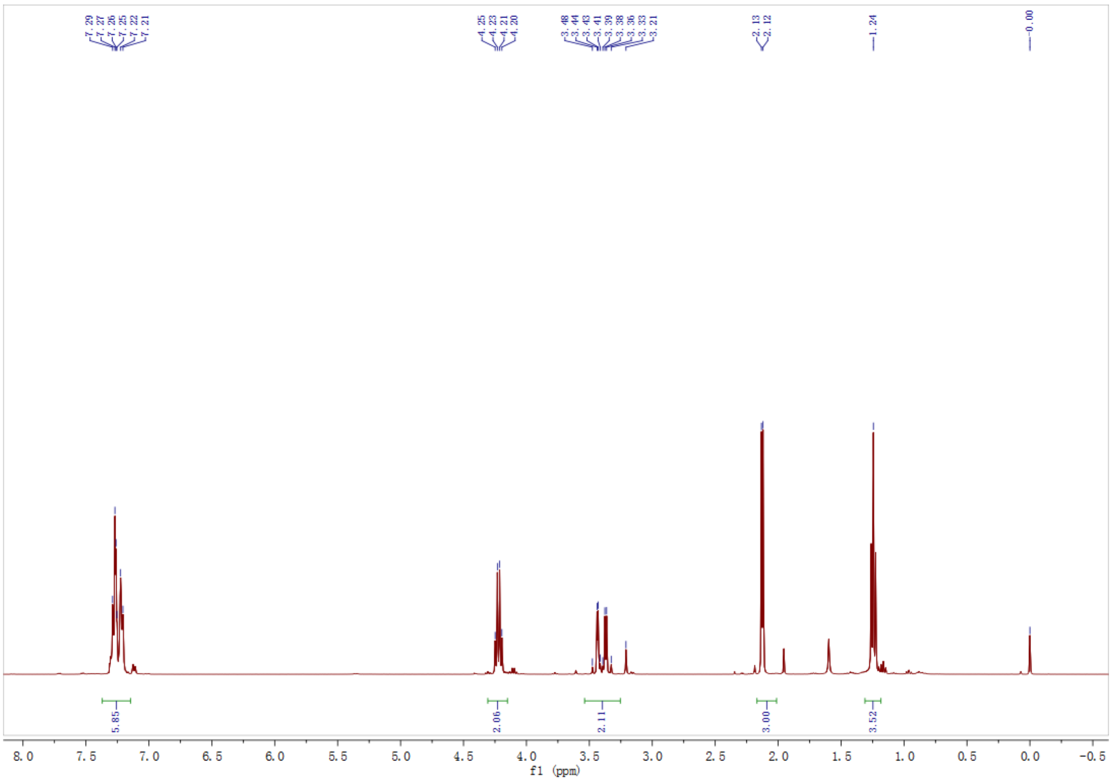

^{13}C NMR ($CDCl_3$, 101 MHz)

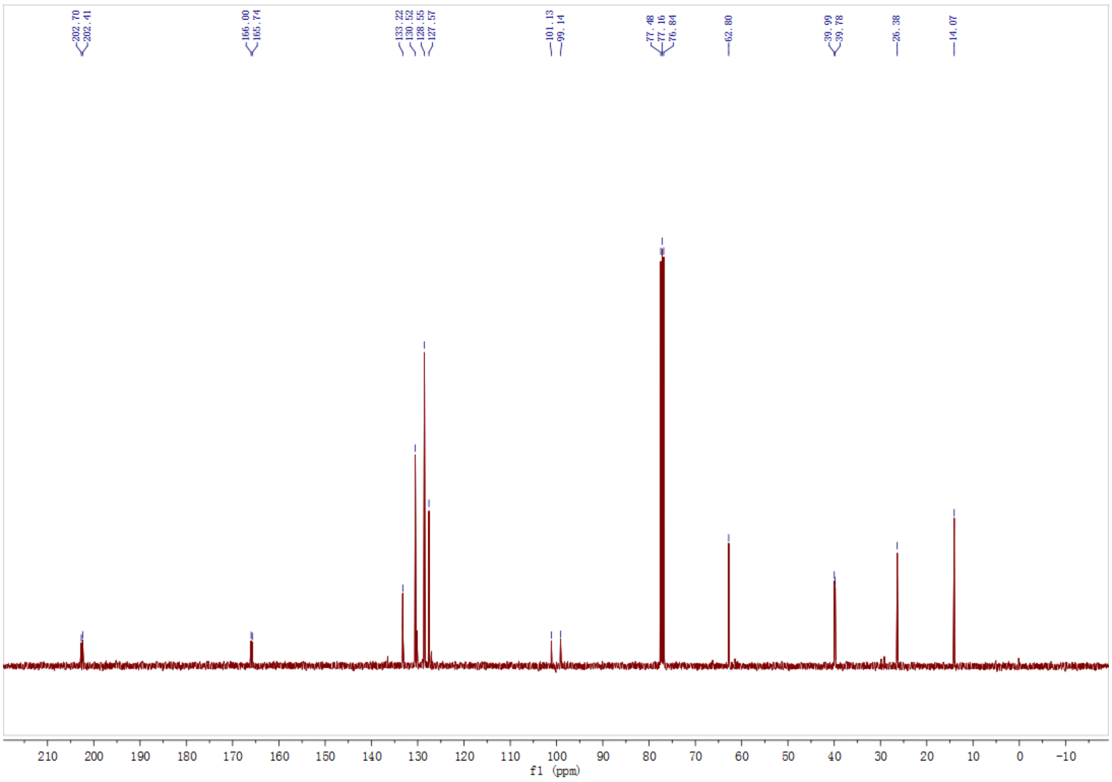

^{19}F NMR ($CDCl_3$, 471 MHz)

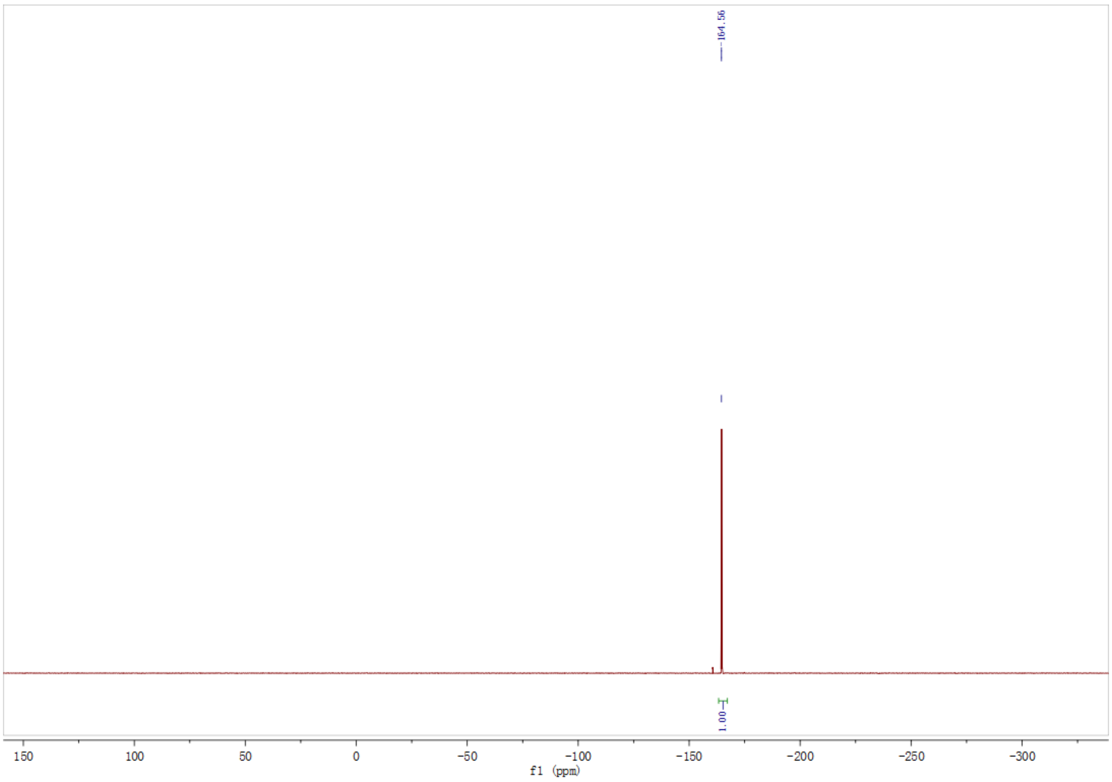

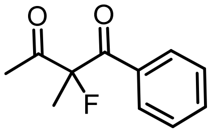

3o

^{1}H NMR ($CDCl_3$, 400 MHz)

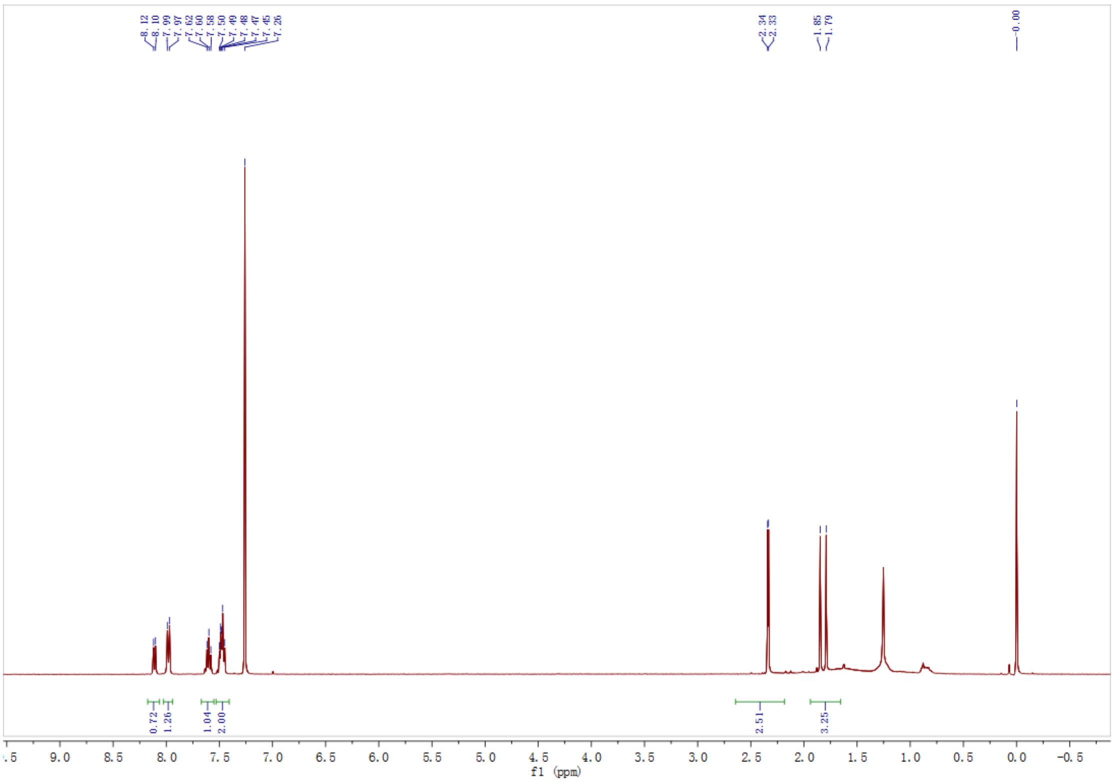

^{13}C NMR ($CDCl_3$, 126 MHz)

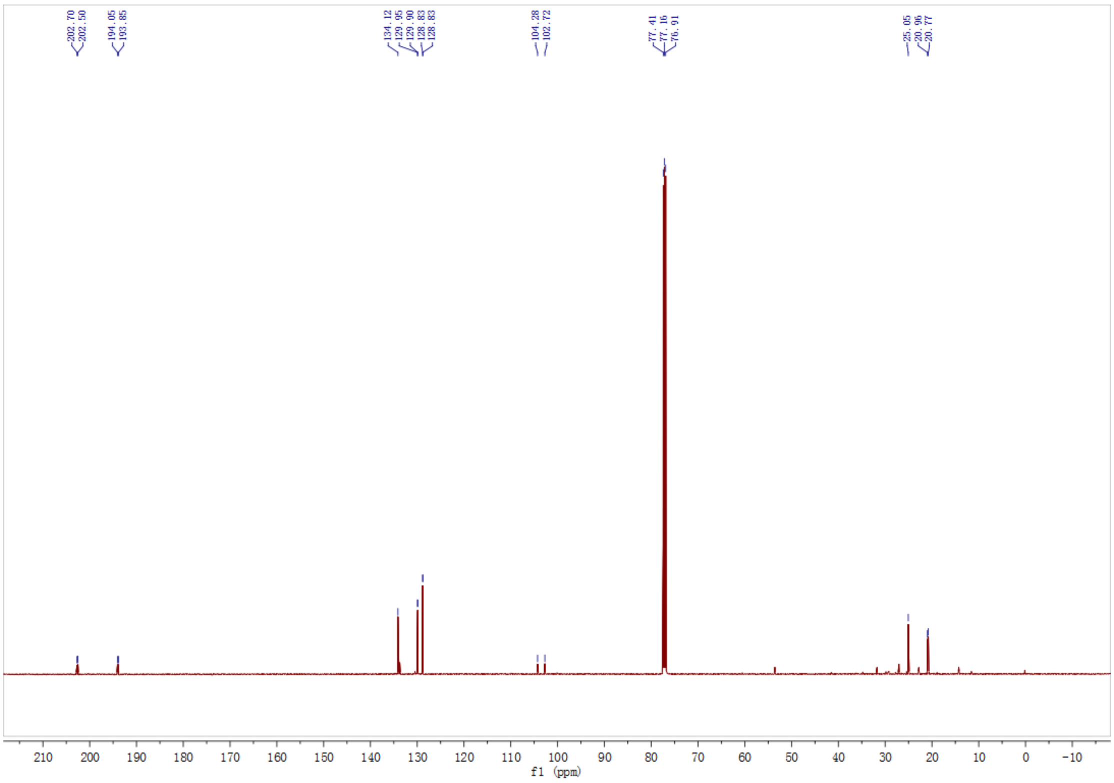

^{19}F NMR ($CDCl_3$, 565 MHz)

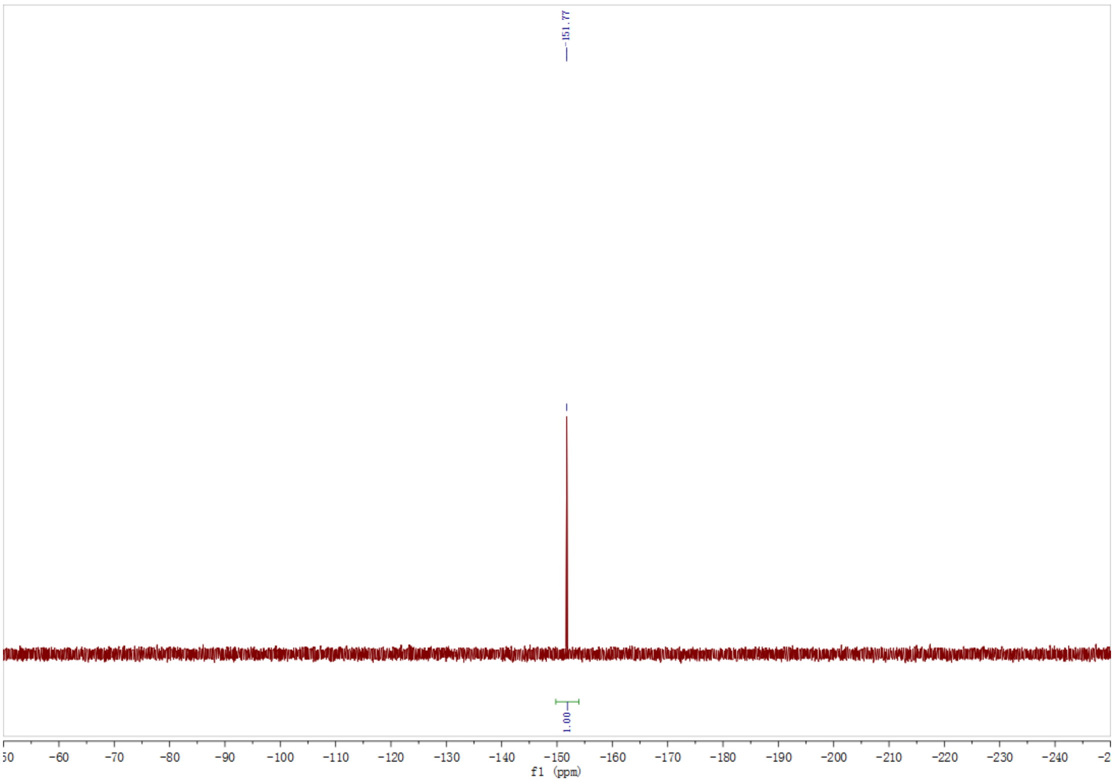

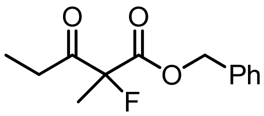

^{1}H NMR ($CDCl_3$, 400 MHz)

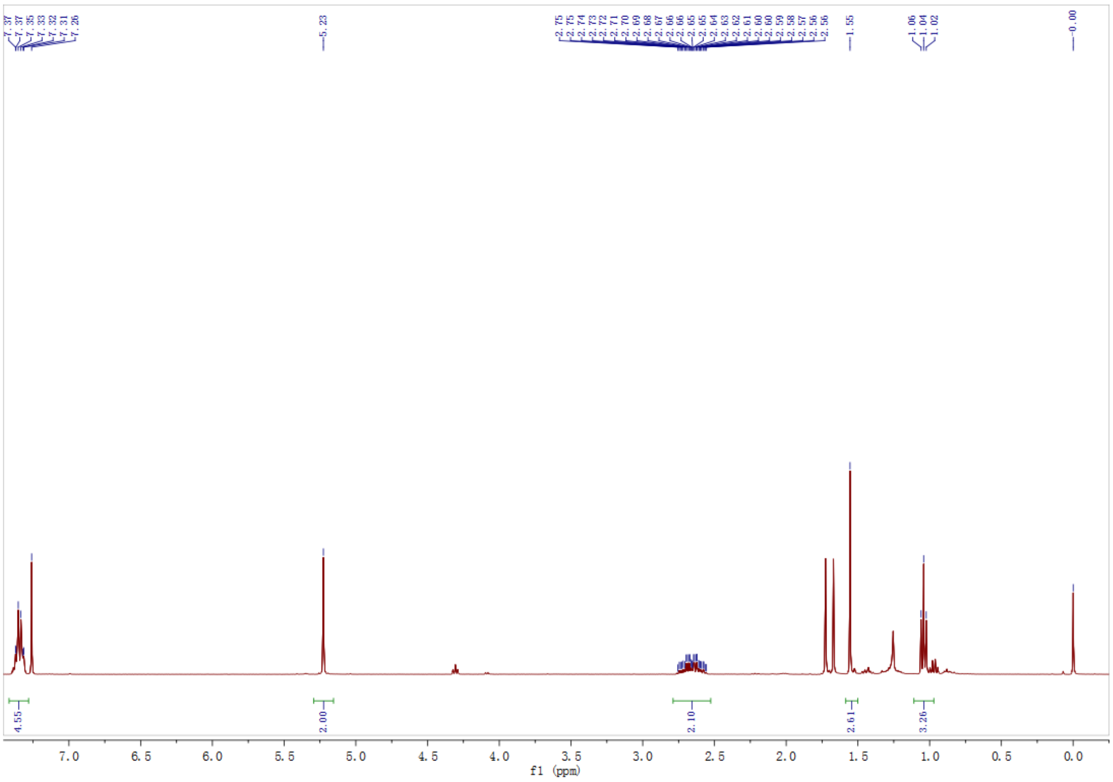

^{13}C NMR ($CDCl_3$, 101 MHz)

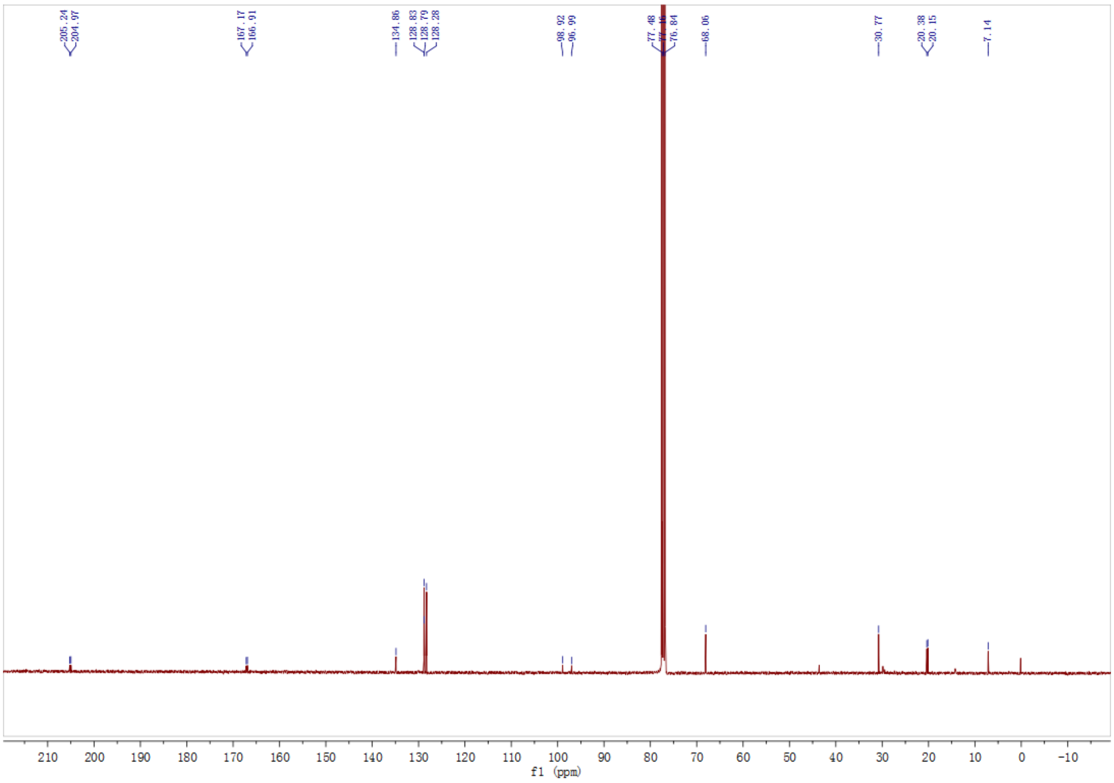

^{19}F NMR ($CDCl_3$, 565 MHz)

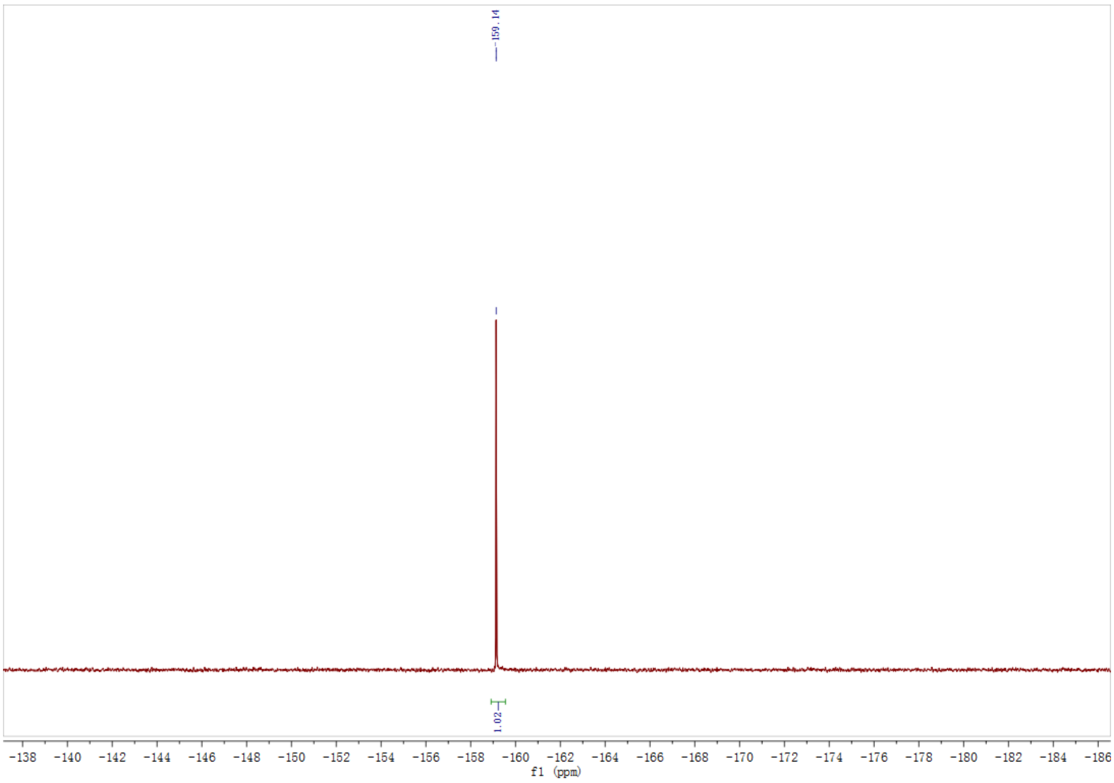

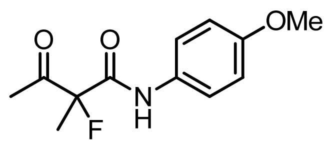

3q

^{1}H NMR ($CDCl_3$, 400 MHz)

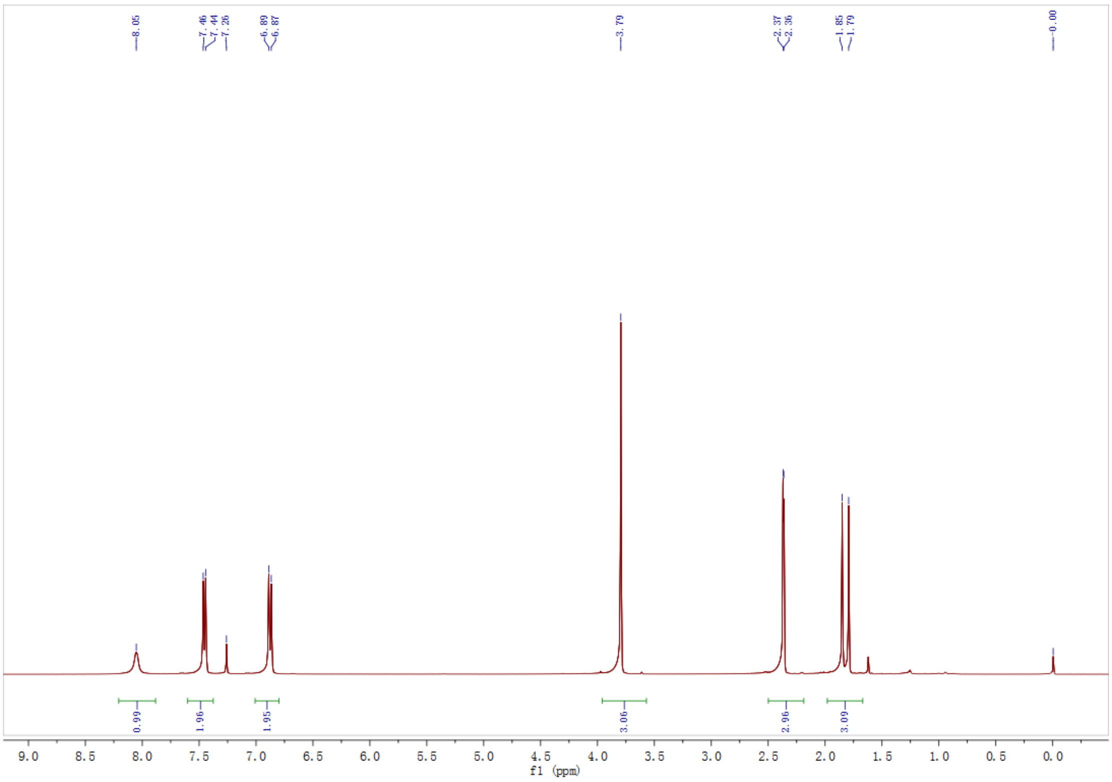

^{13}C NMR ($CDCl_3$, 101 MHz)

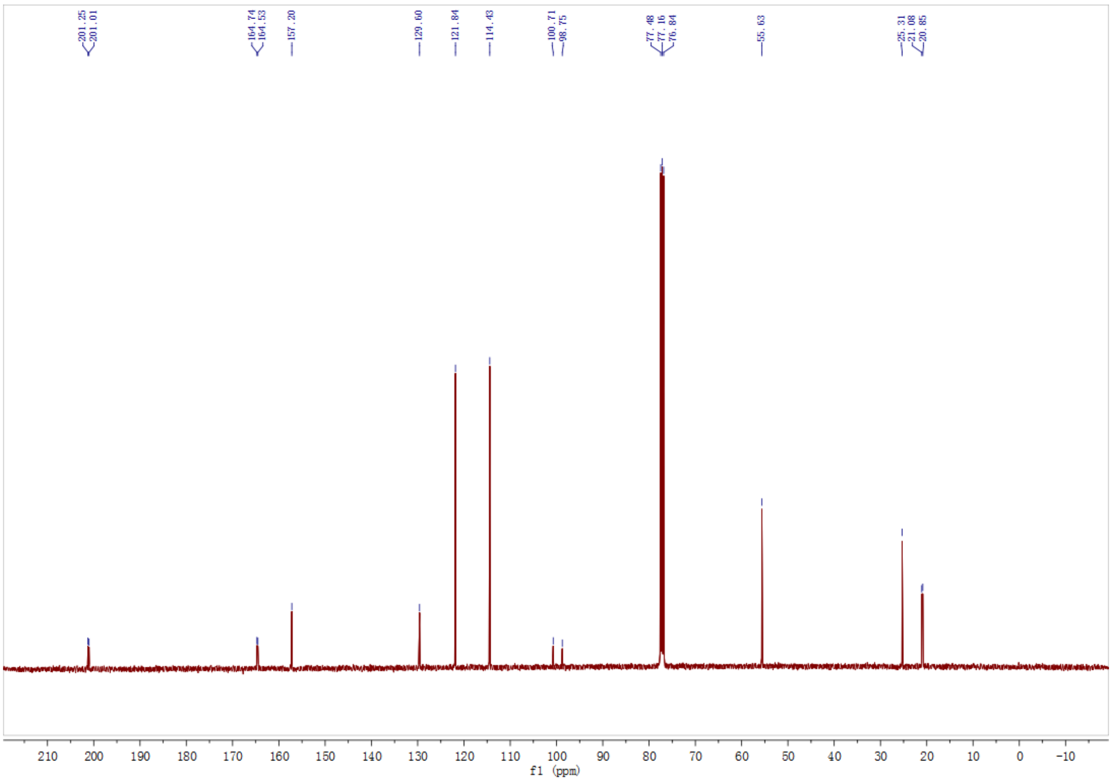

^{19}F NMR ($CDCl_3$, 565 MHz)

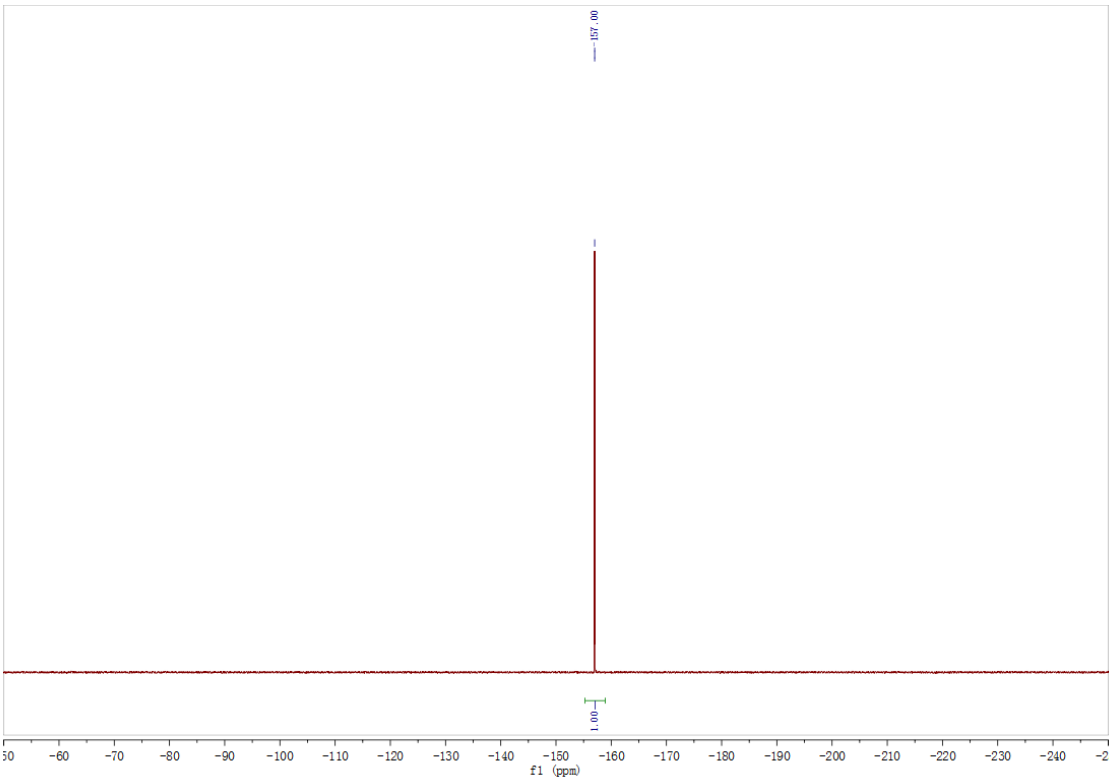

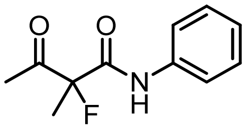

3r

^{1}H NMR ($CDCl_3$, 400 MHz)

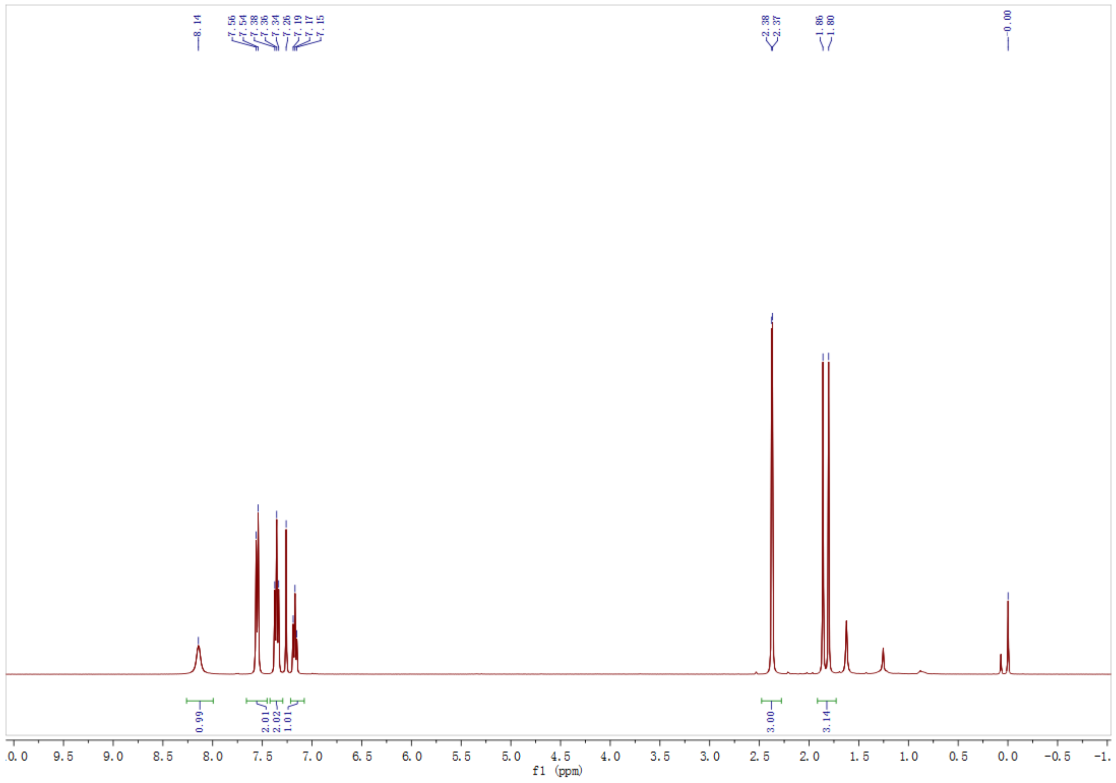

^{13}C NMR ($CDCl_3$, 101 MHz)

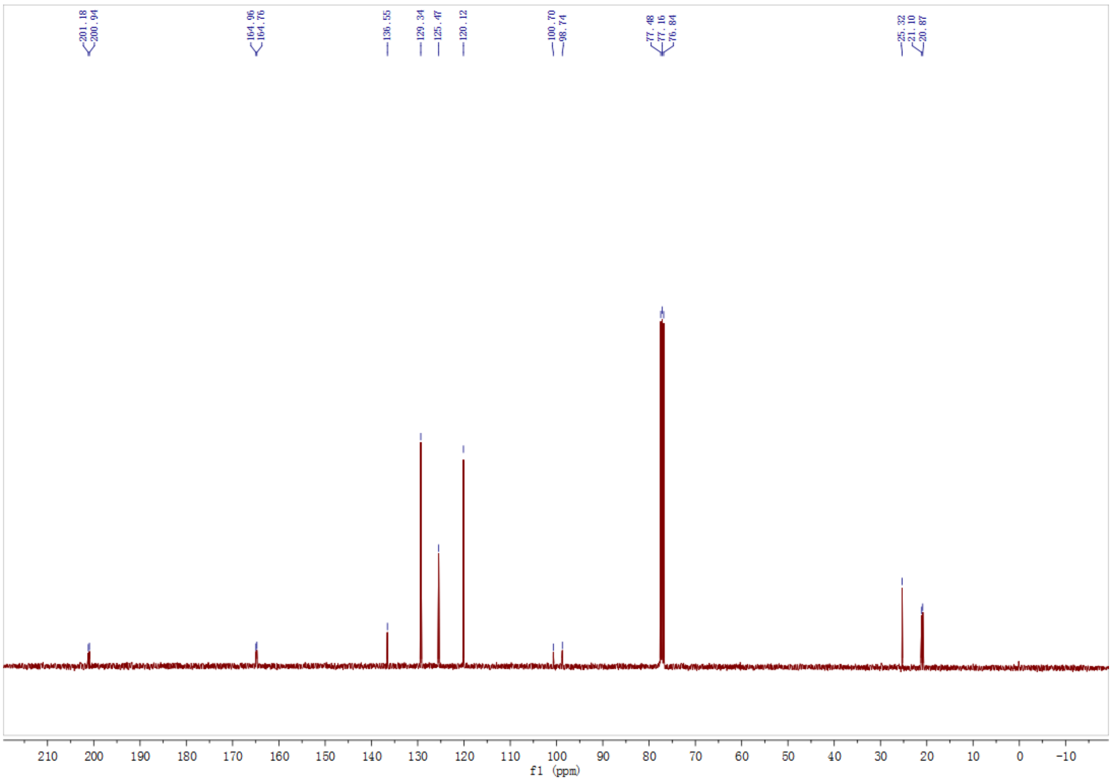

^{19}F NMR ($CDCl_3$, 565 MHz)

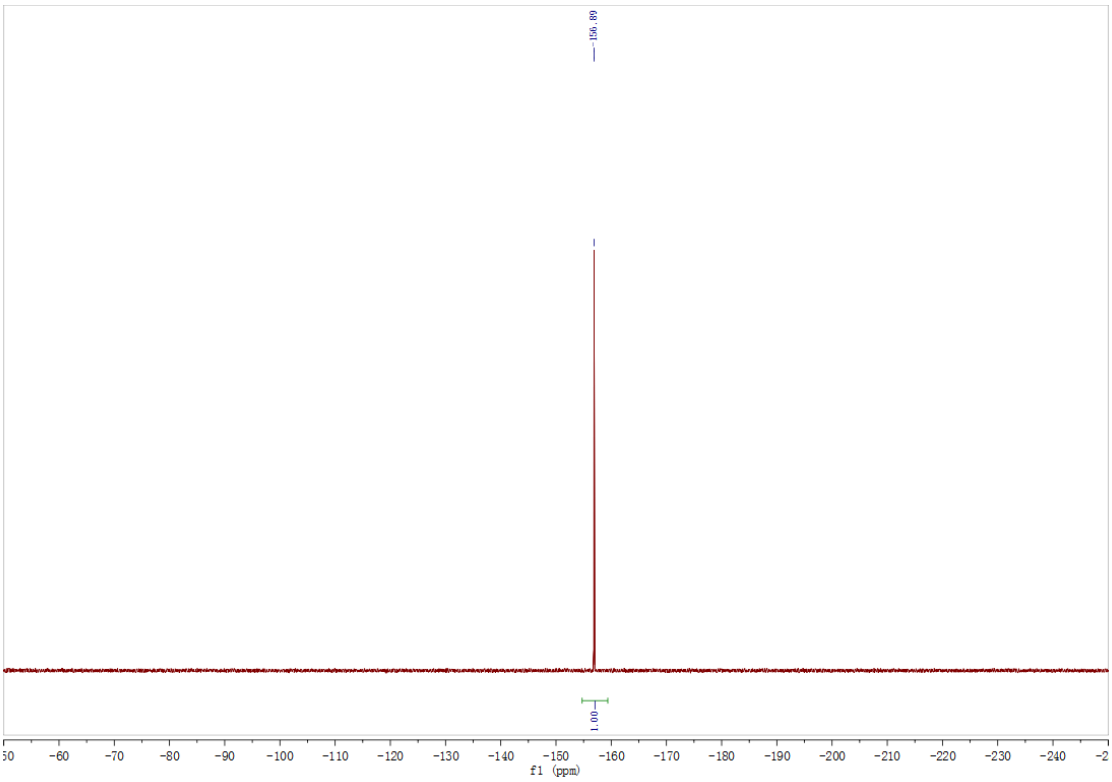

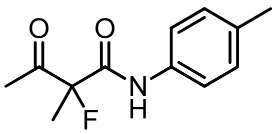

3s

^{1}H NMR ($CDCl_3$, 400 MHz)

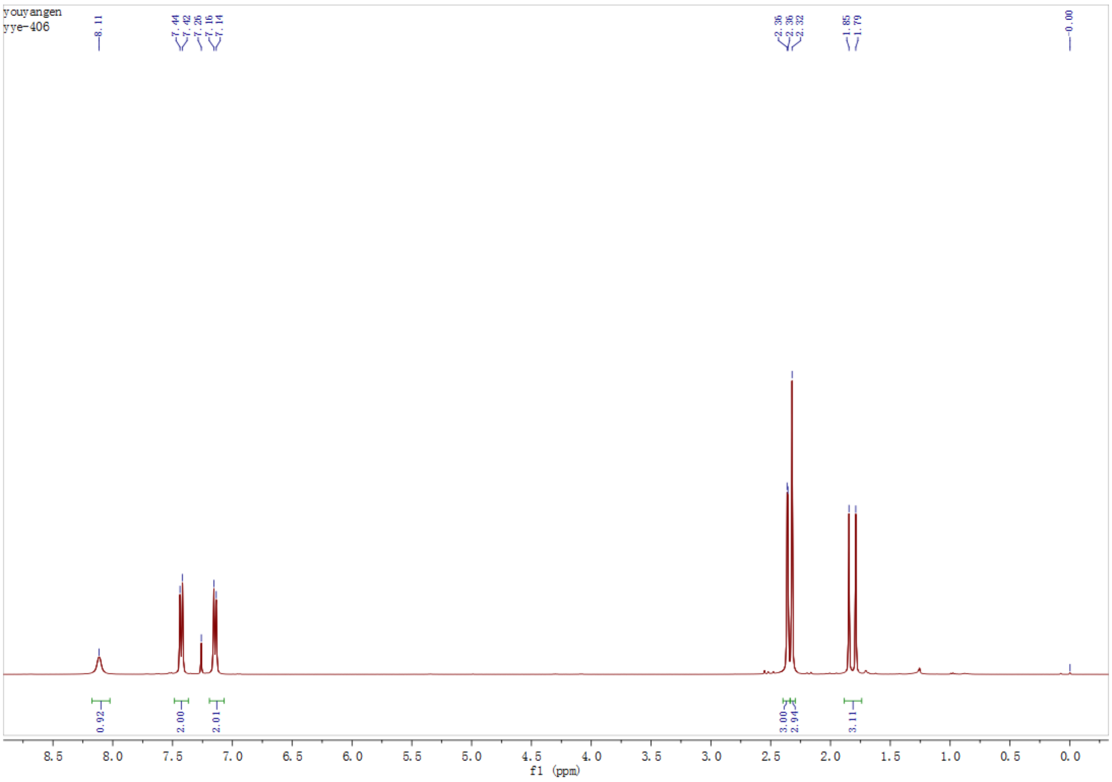

^{13}C NMR ($CDCl_3$, 101 MHz)

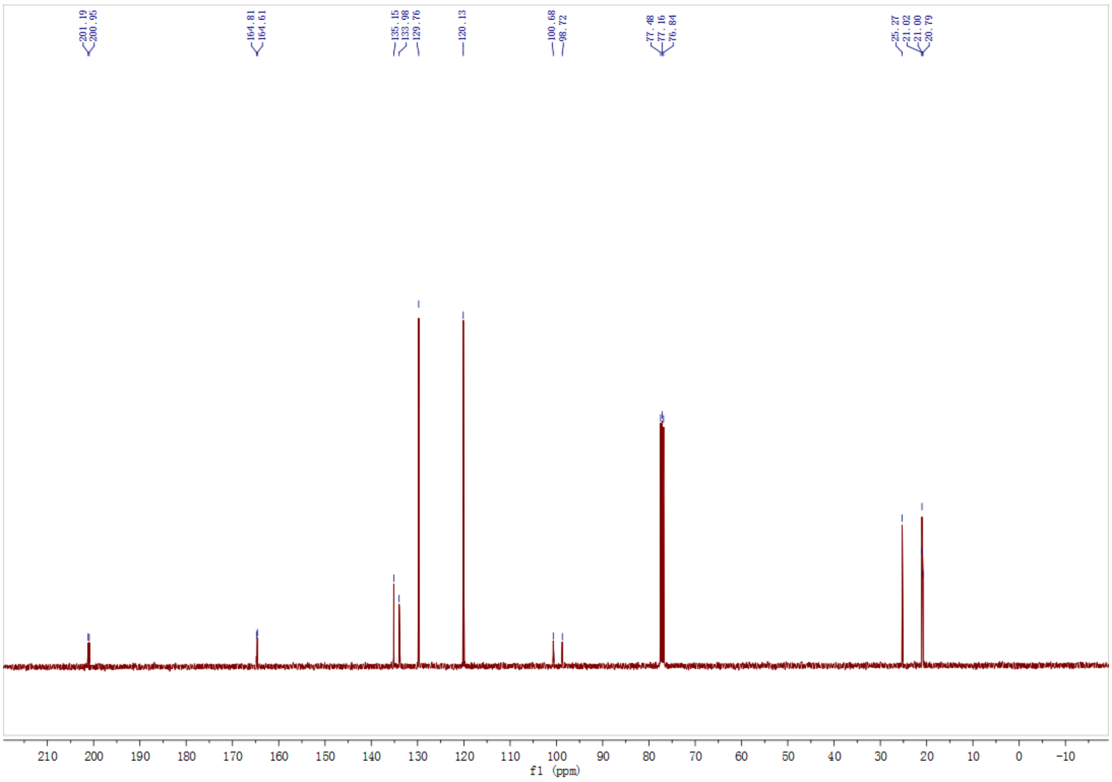

^{19}F NMR ($CDCl_3$, 565 MHz)

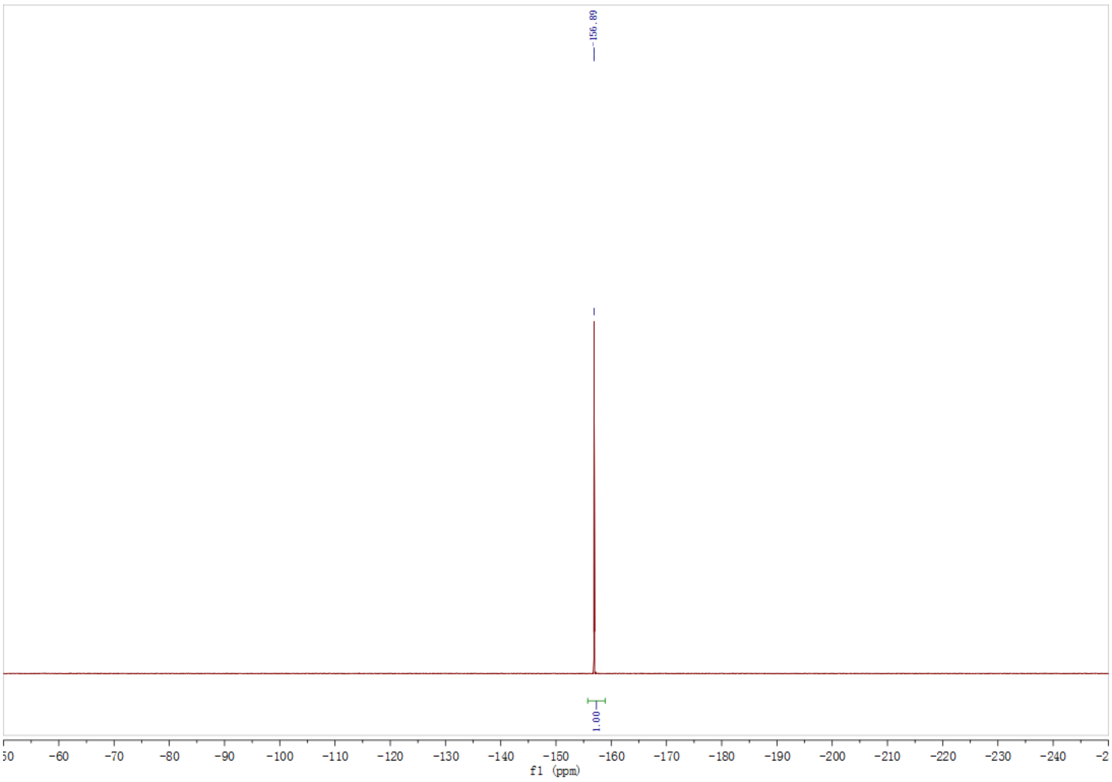

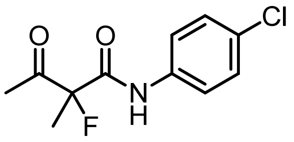

3t

^{1}H NMR ($CDCl_3$, 400 MHz)

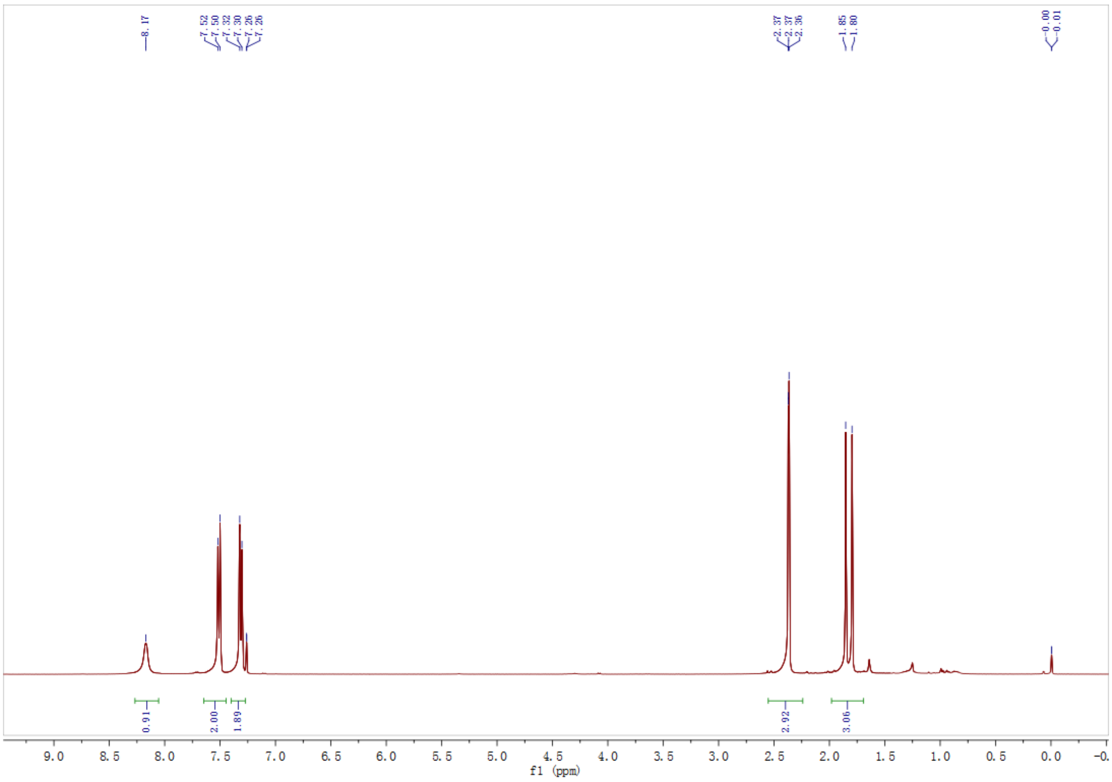

^{13}C NMR ($CDCl_3$, 101 MHz)

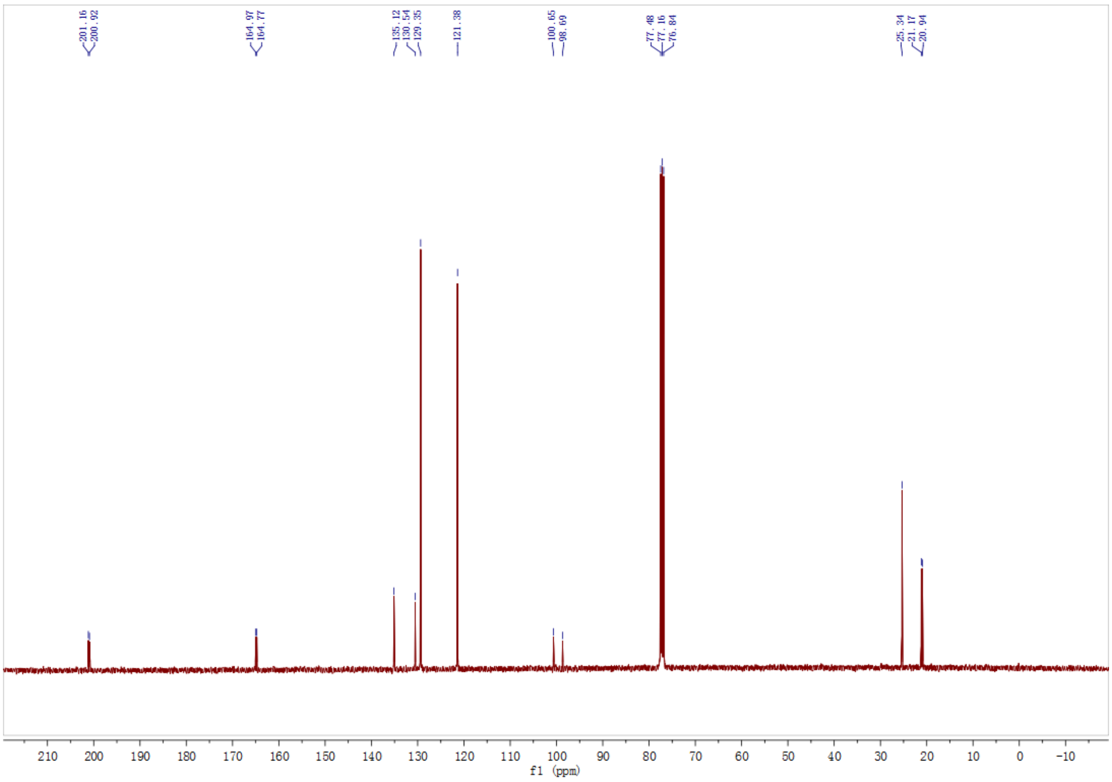

^{19}F NMR ($CDCl_3$, 565 MHz)

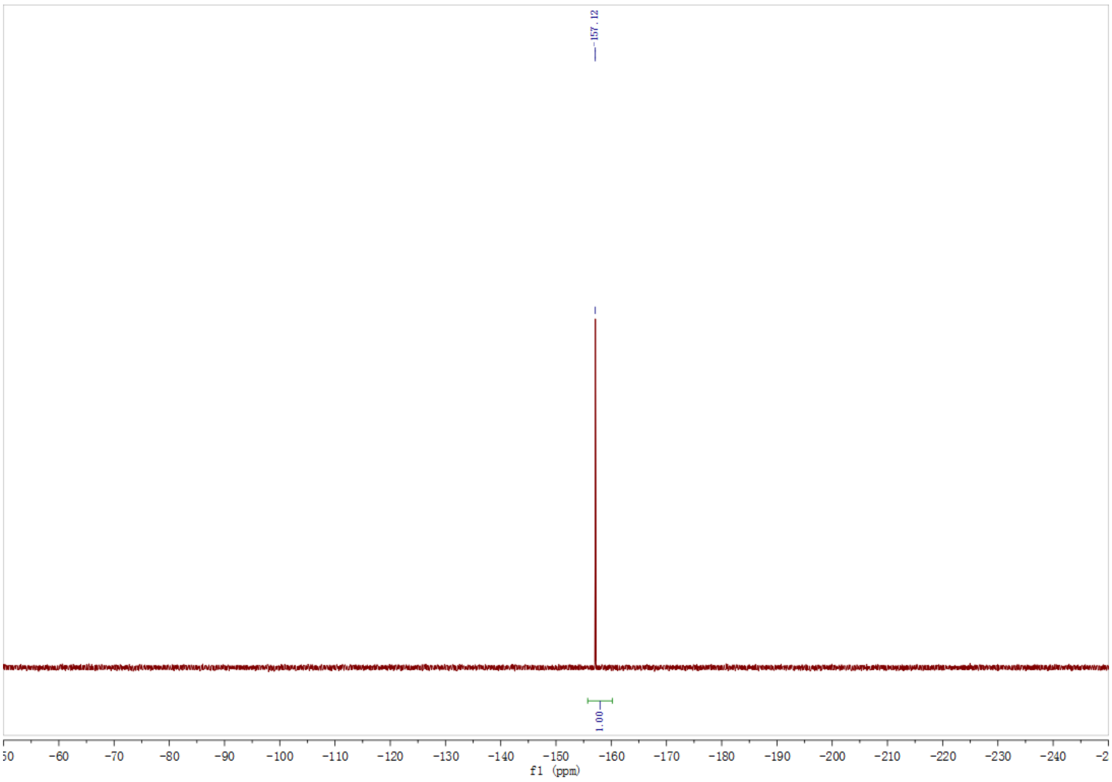

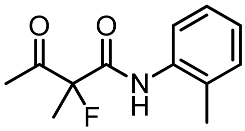

3u

^{1}H NMR ($CDCl_3$, 400 MHz)

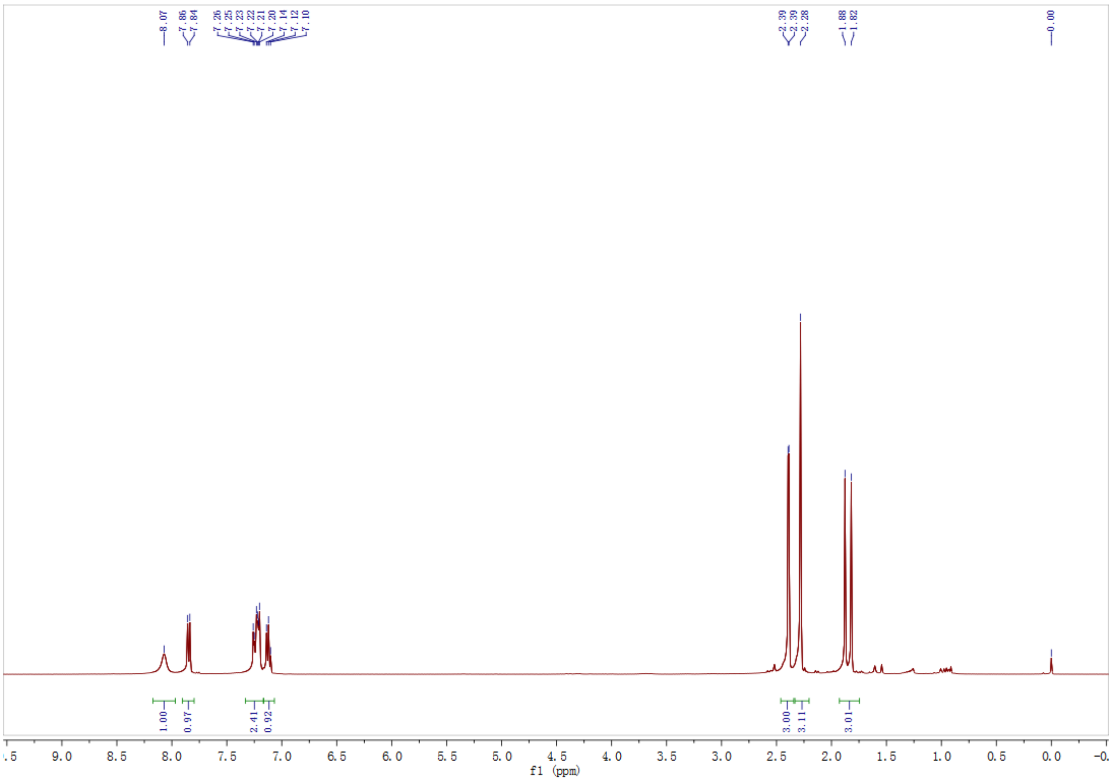

^{13}C NMR (CDCl$_3$, 101 MHz)

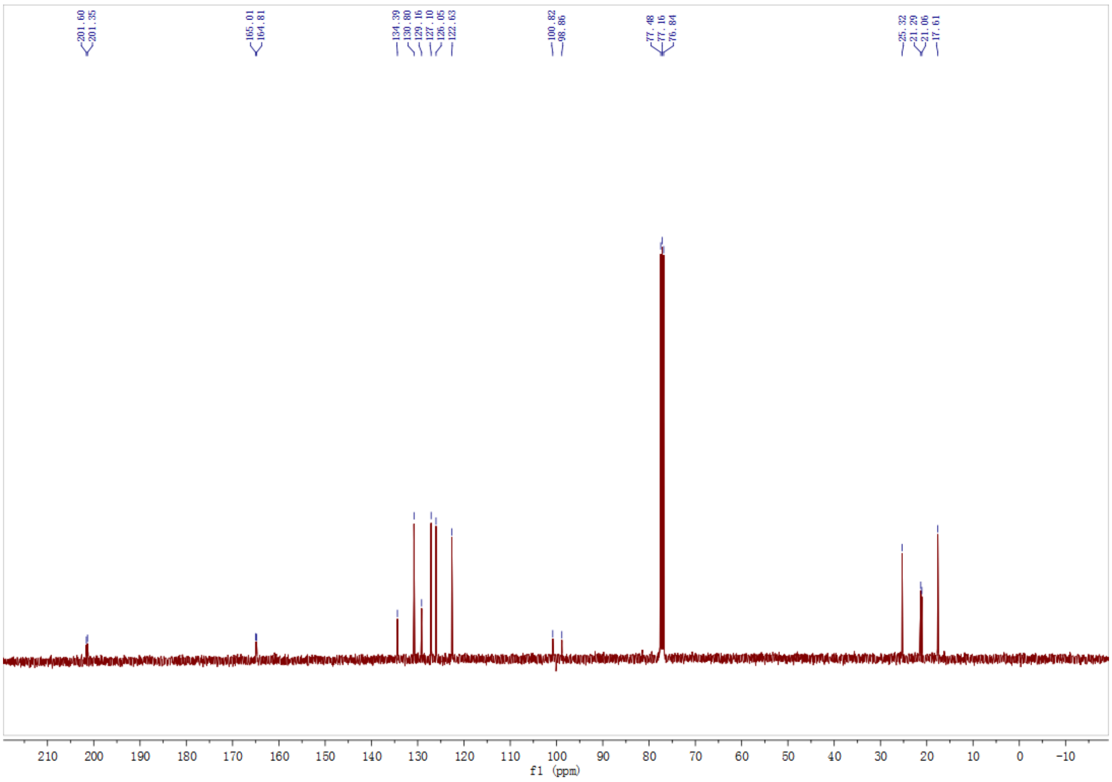

^{19}F NMR (CDCl$_3$, 565 MHz)

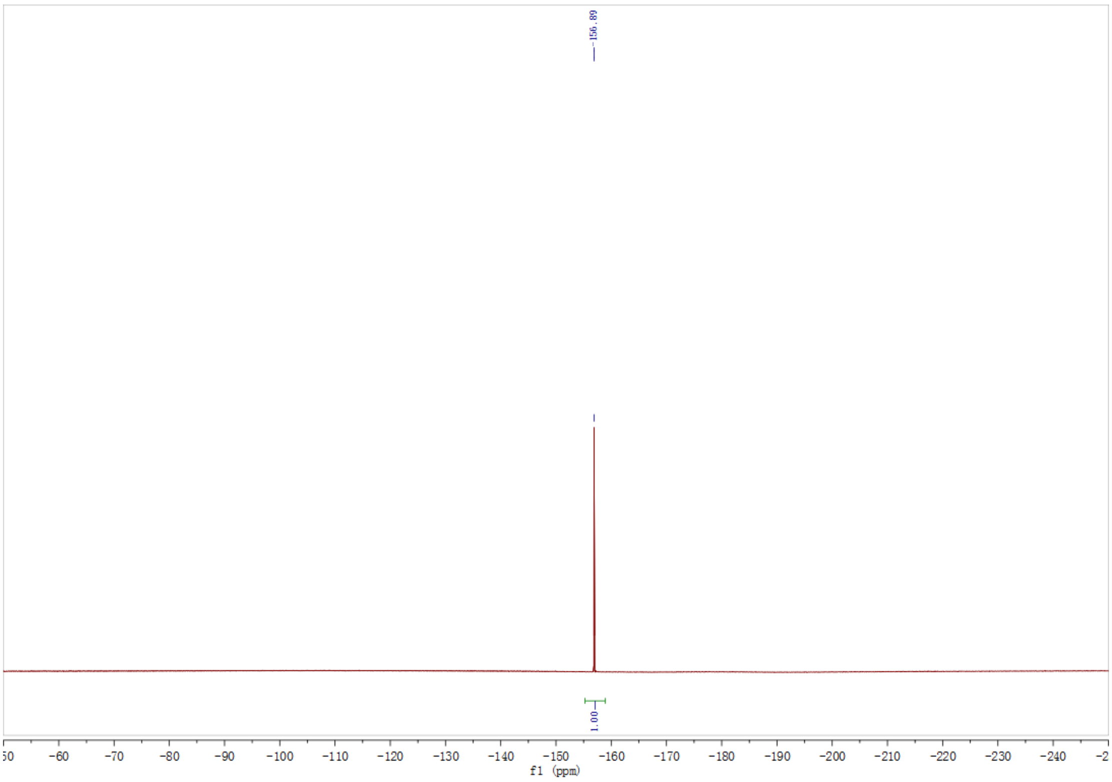

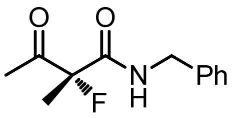

3v

^{1}H NMR ($CDCl_3$, 400 MHz)

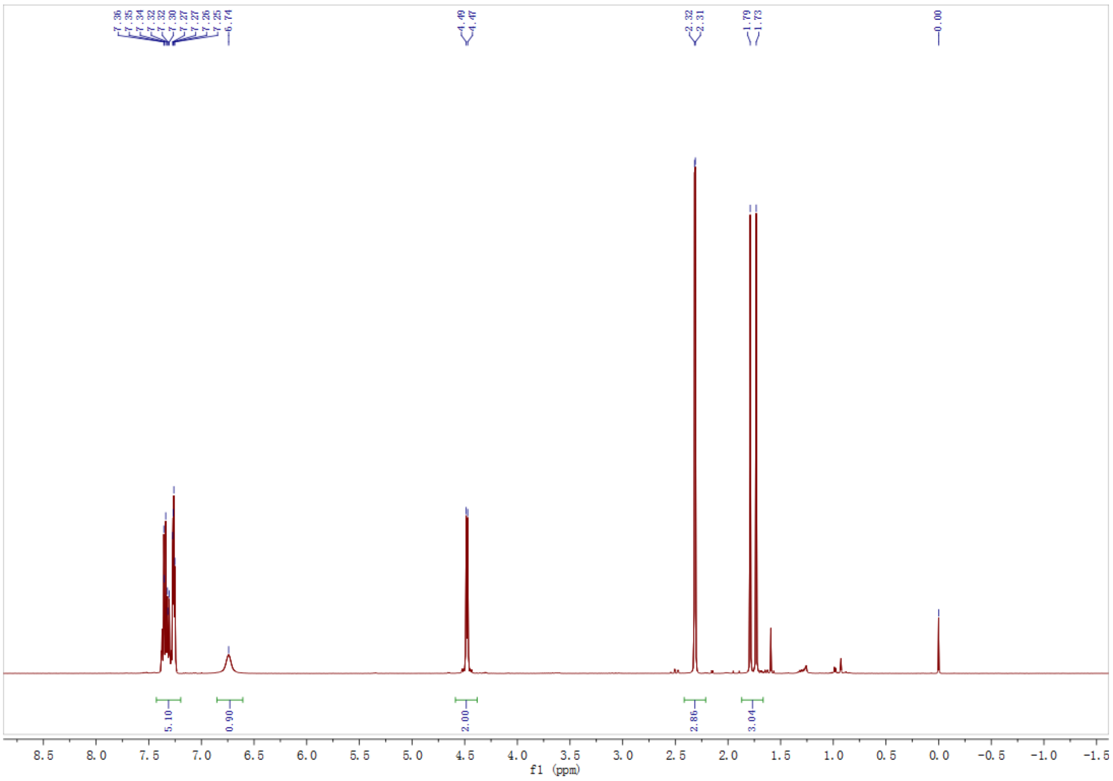

^{13}C NMR ($CDCl_3$, 101 MHz)

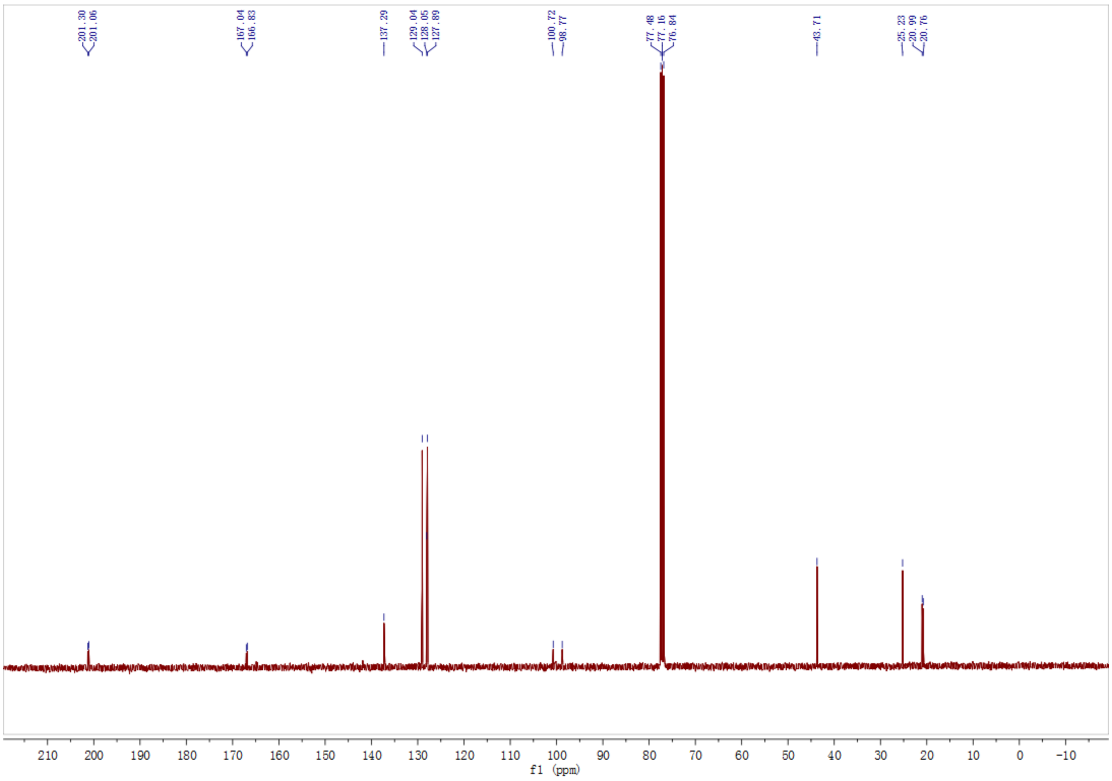

^{19}F NMR ($CDCl_3$, 565 MHz)

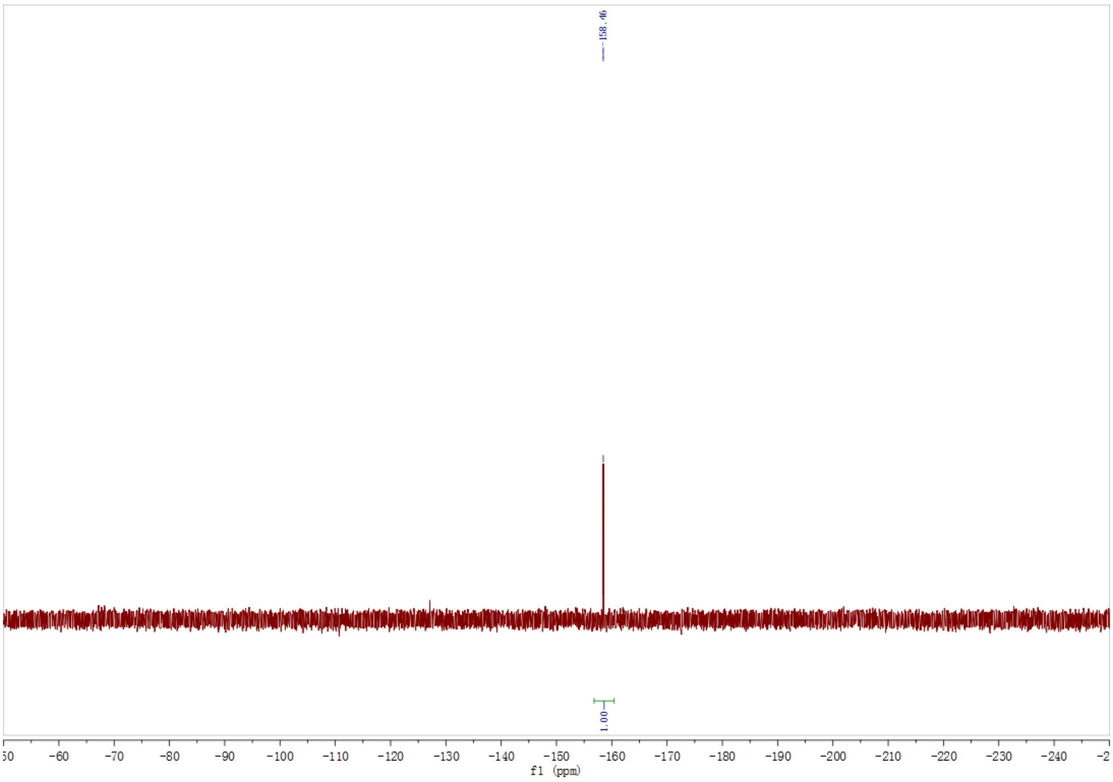

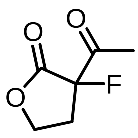

3w

^{1}H NMR (CDCl$_3$, 400 MHz)

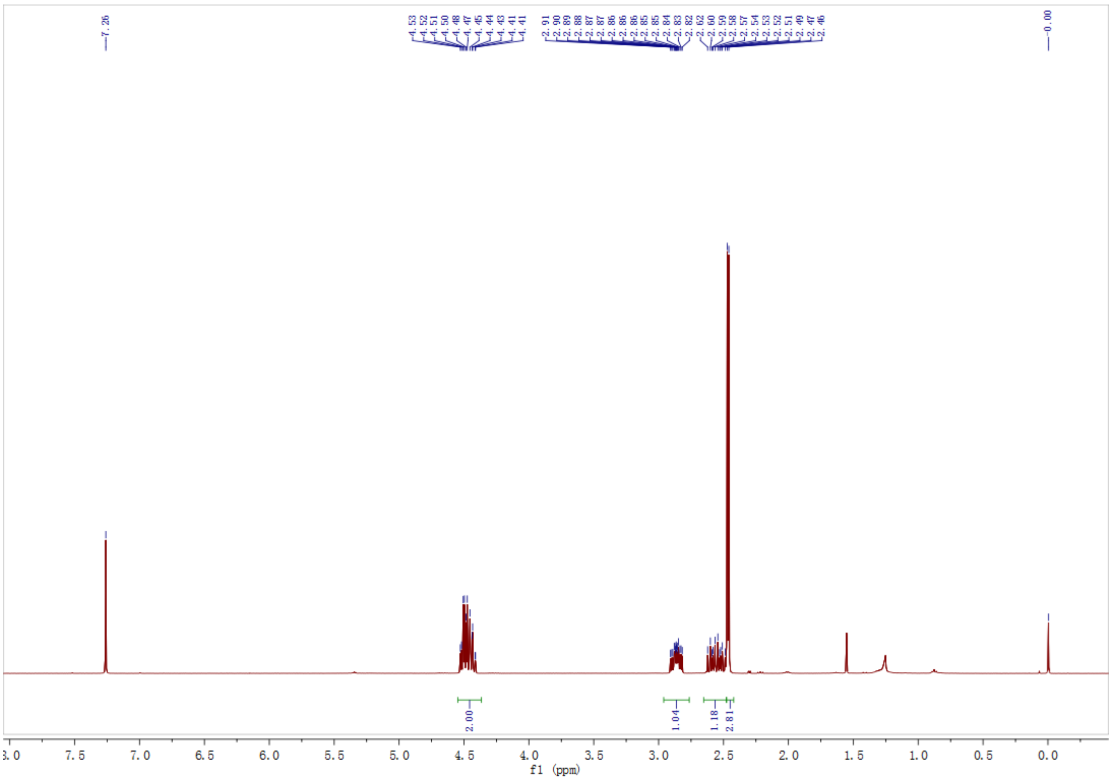

^{13}C NMR ($CDCl_3$, 101 MHz)

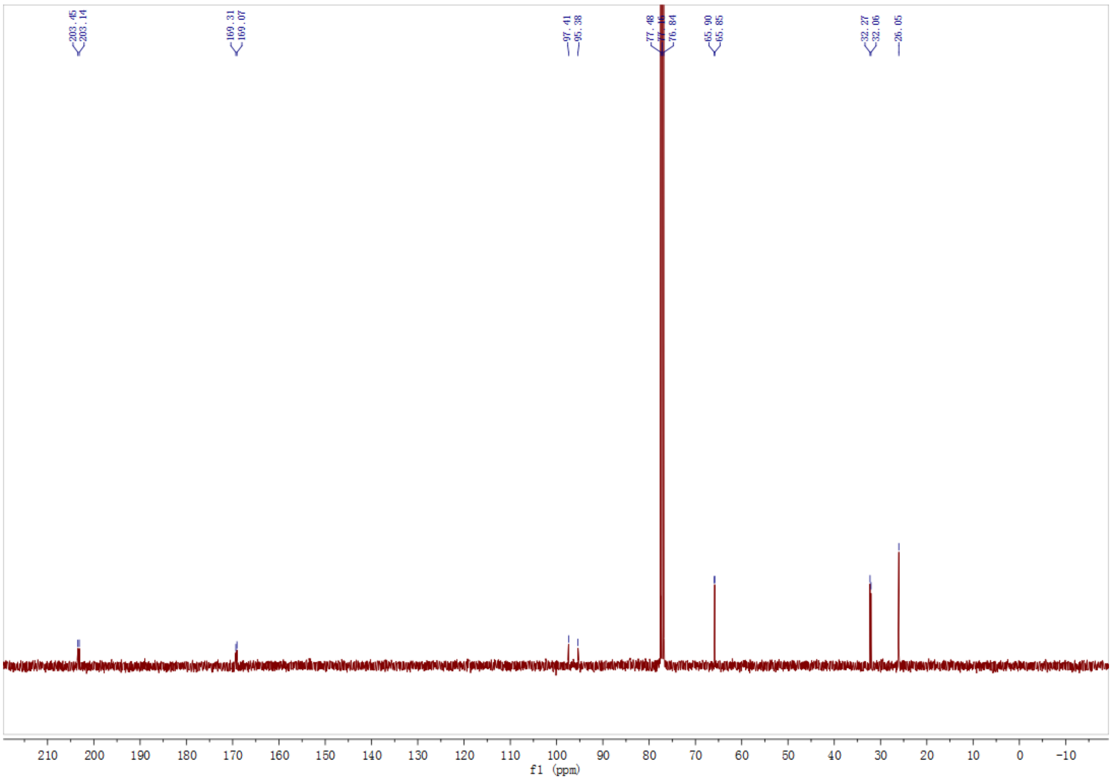

^{19}F NMR ($CDCl_3$, 565 MHz)

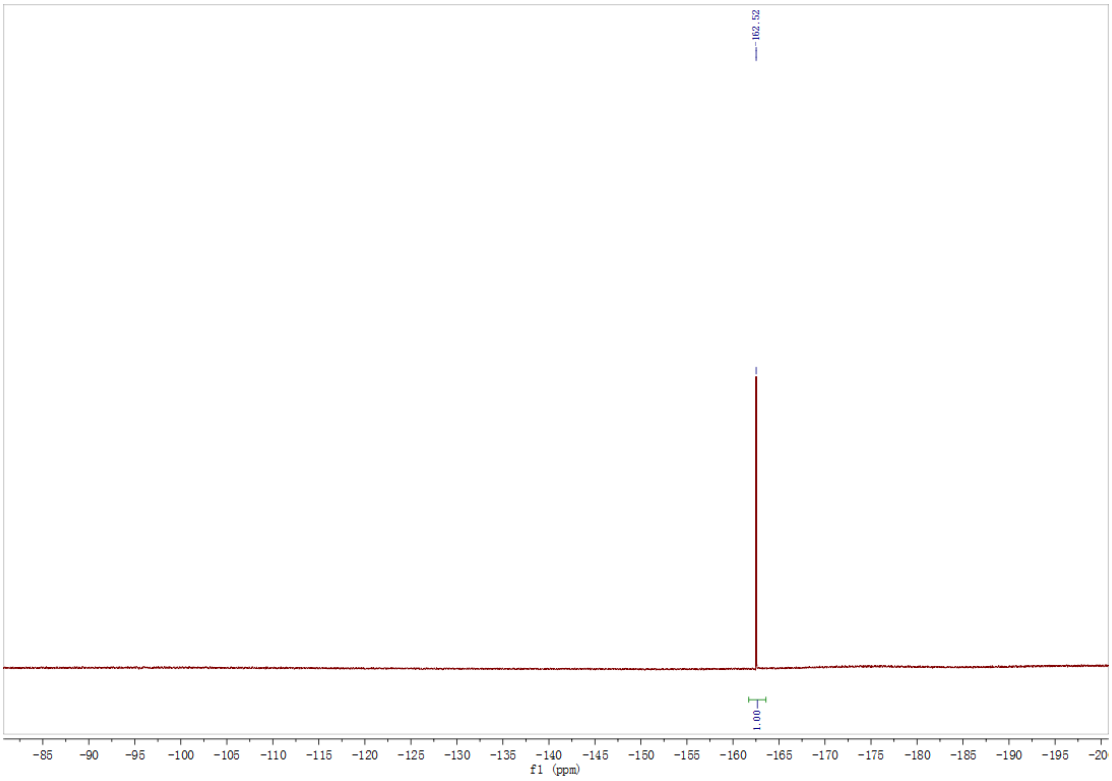

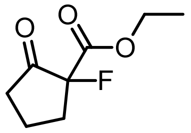

3x

^{1}H NMR (CDCl$_3$, 400 MHz)

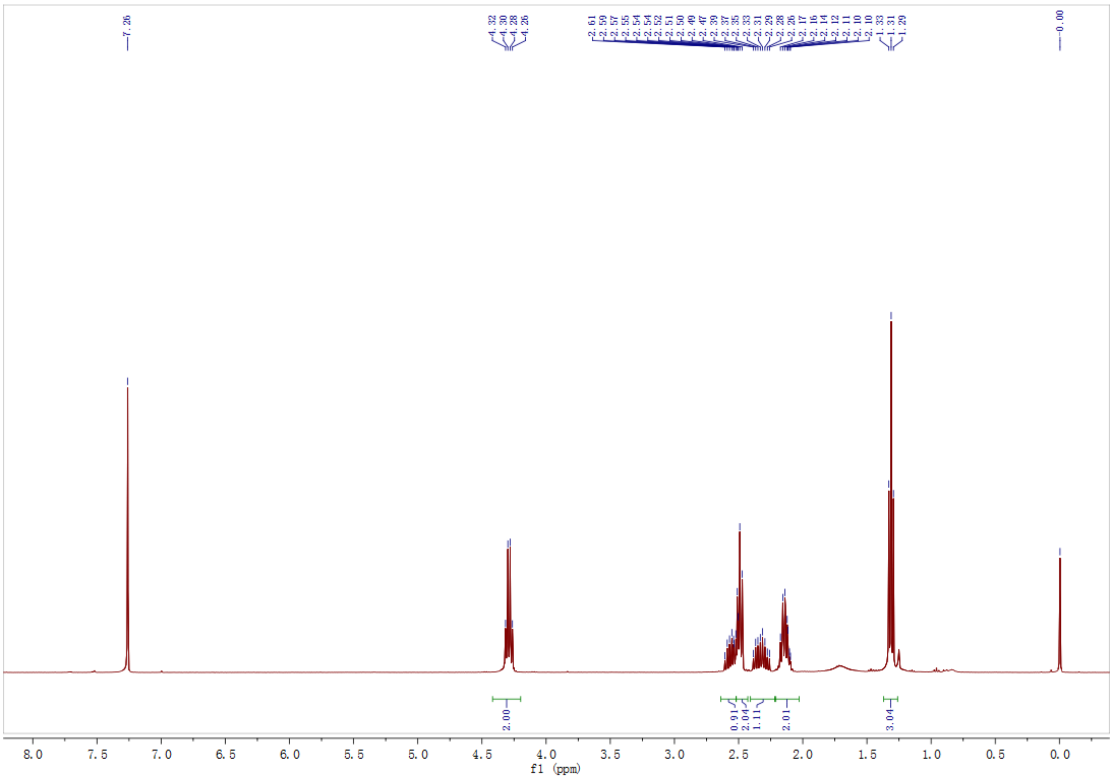

^{13}C NMR ($CDCl_3$, 101 MHz)

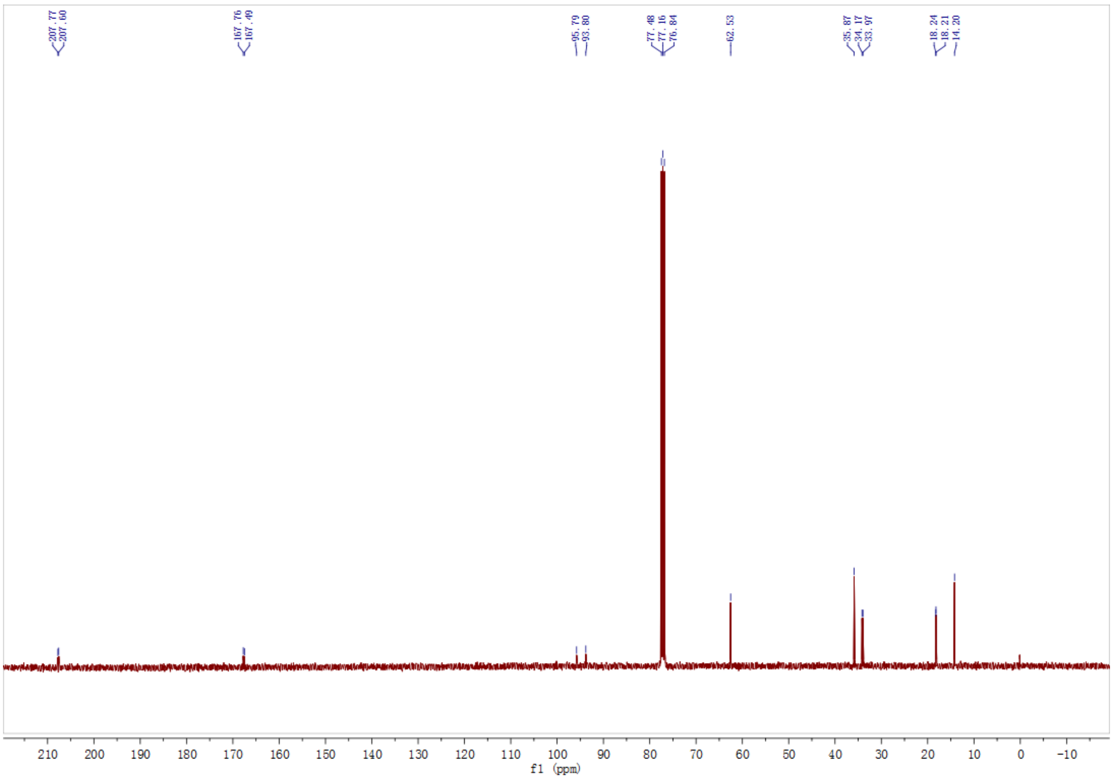

^{19}F NMR ($CDCl_3$, 565 MHz)

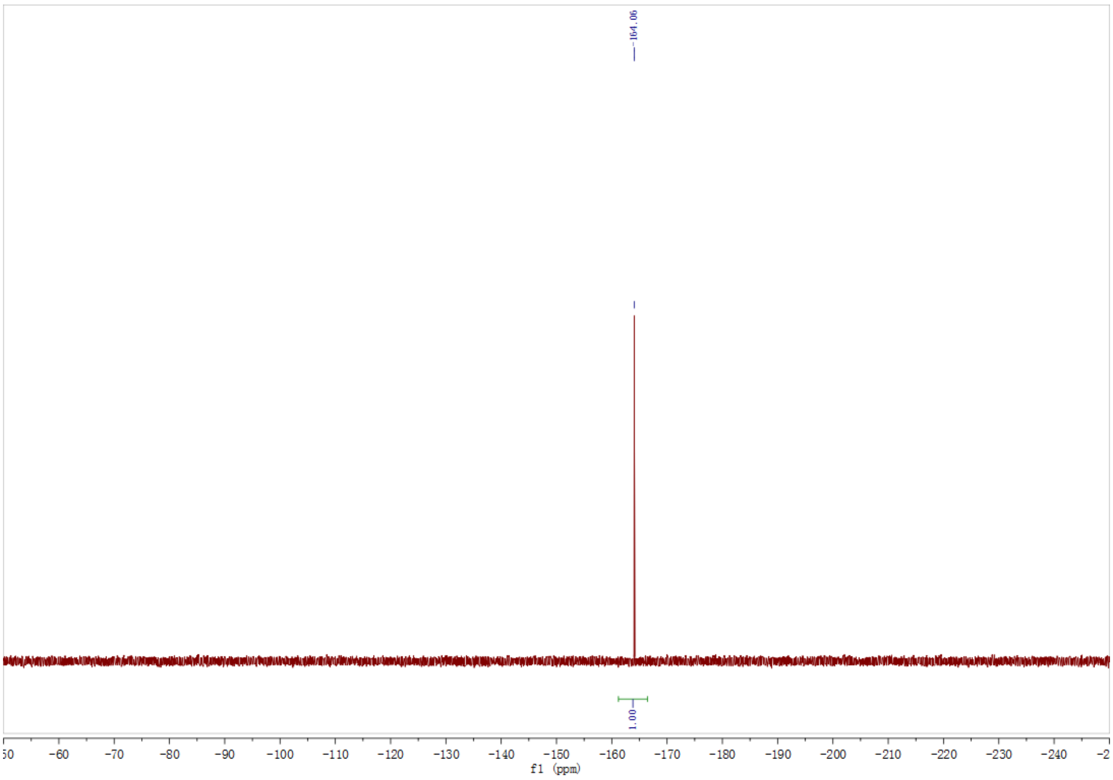

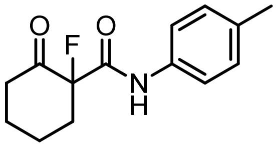

3y

^{1}H NMR ($CDCl_3$, 400 MHz)

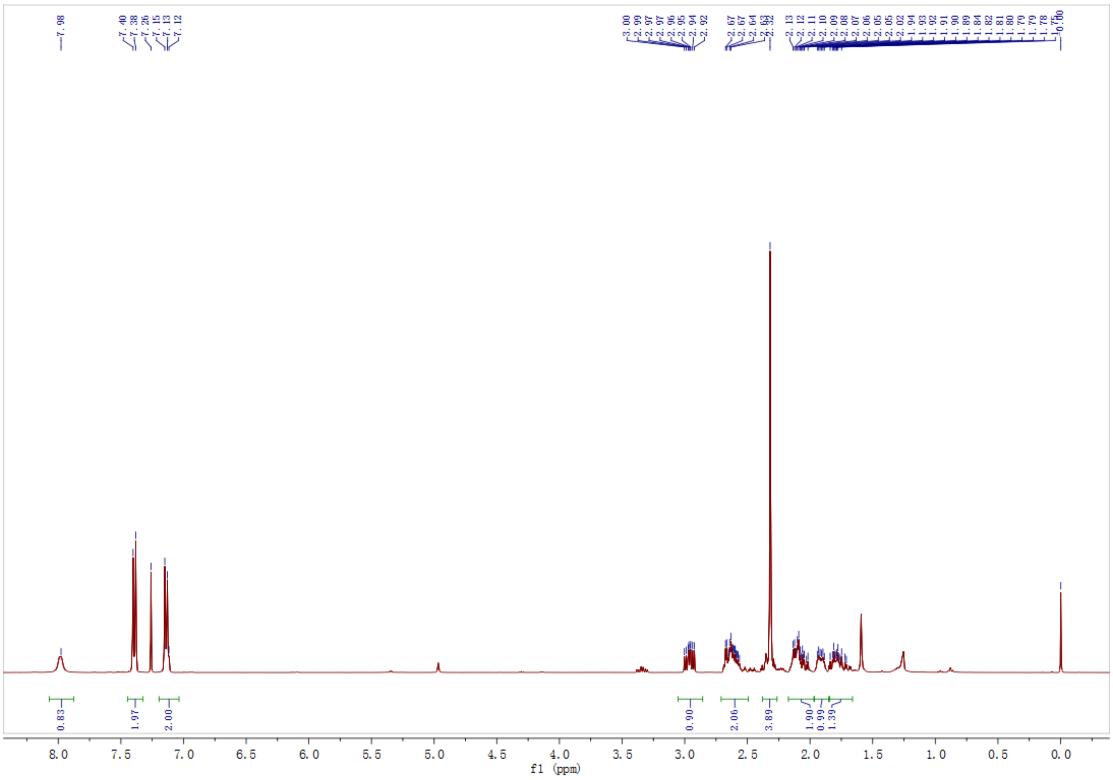

^{13}C NMR ($CDCl_3$, 101 MHz)

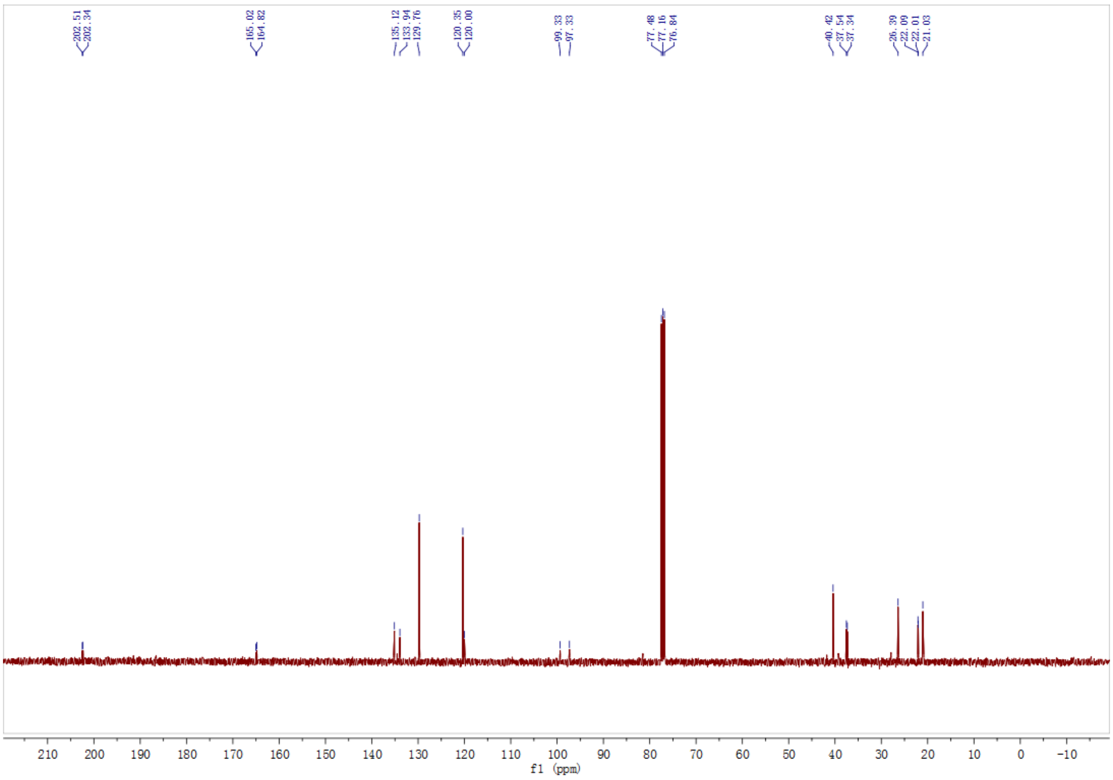

^{19}F NMR ($CDCl_3$, 565 MHz)

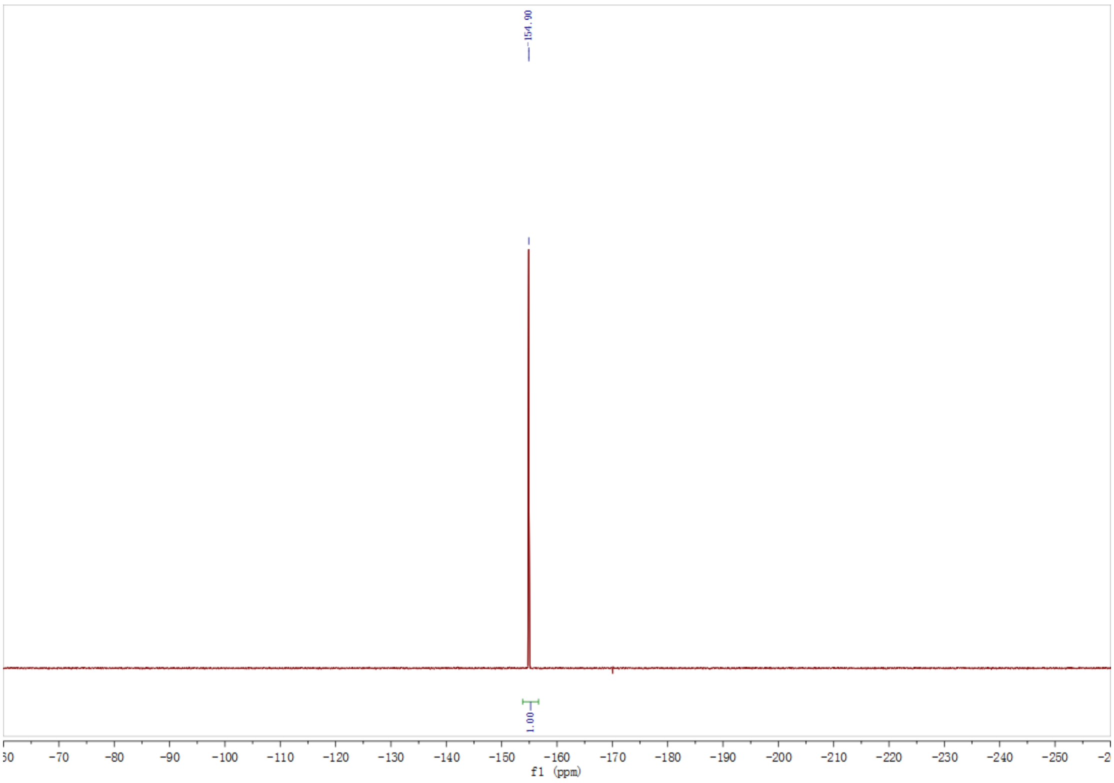

HPLC charts:

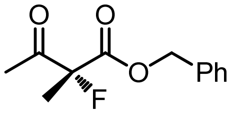

3a

〈Chromatogram〉

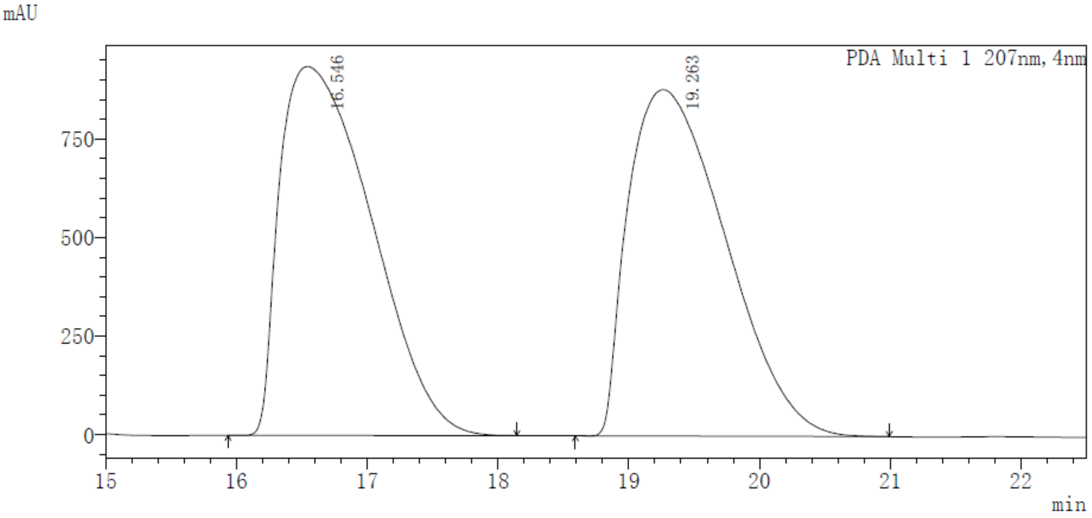

〈Peak Results〉

PDA Ch1 207nm

Index	Time/min	Height/mAU	Quantity/Area	Area %/%
1	16.546	935813	45694254	50.047
2	19.263	879203	45607808	49.953

〈Chromatogram〉

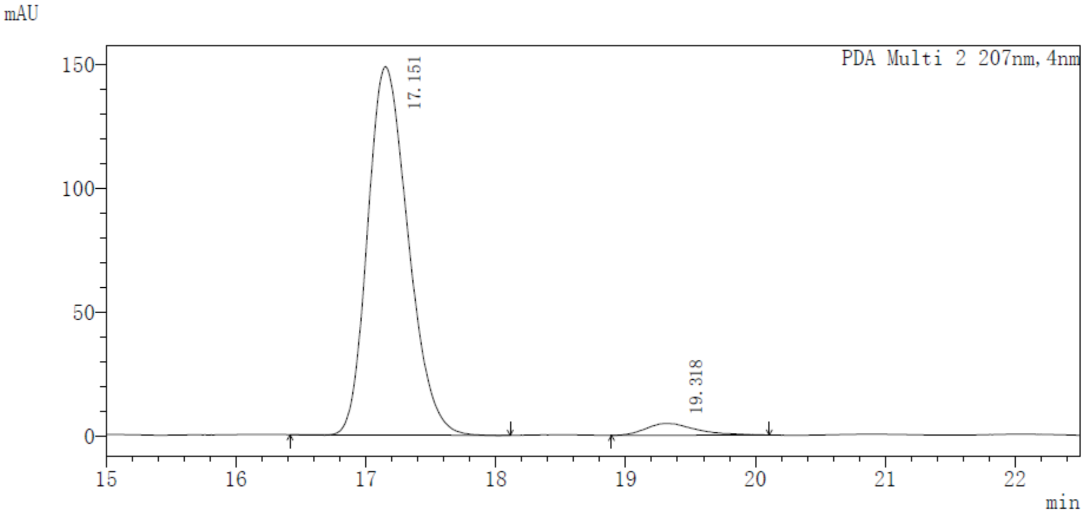

〈Peak Results〉

PDA Ch2 207nm

Index	Time/min	Height/mAU	Quantity/Area	Area %/%
1	17.151	148832	3223443	96.205
2	19.318	4777	127144	3.795

Gram Scale results:

<Chromatogram>

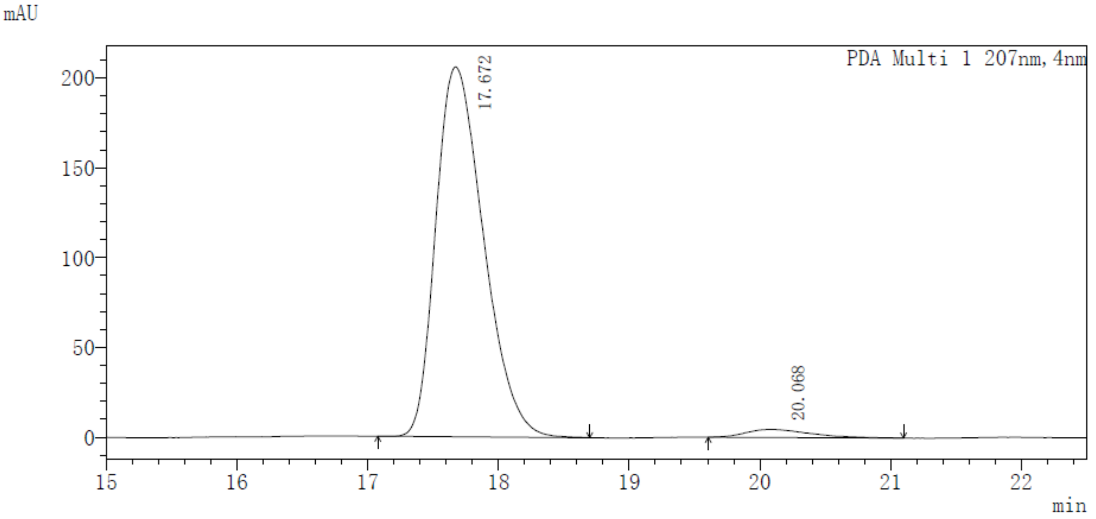

<Peak Results>

PDA Ch1 207nm

Index	Time/min	Height/mAU	Quantity/Area	Area %/%
1	17.672	205932	5228557	97.162
2	20.068	4502	152693	2.838

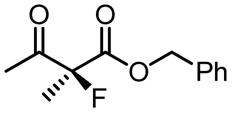

3a

Catalyst II/DNBA II results:

<Chromatogram>

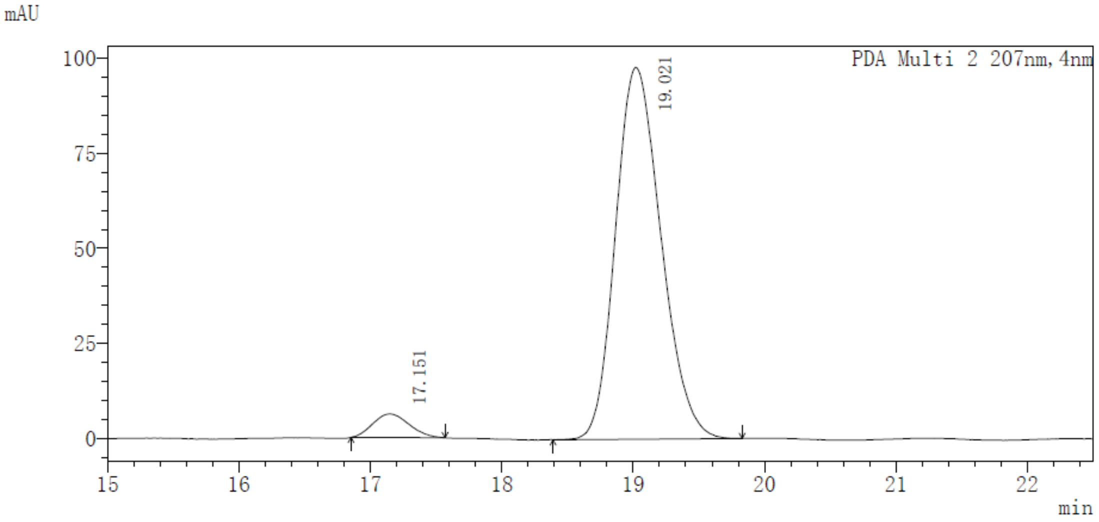

<Peak Results>

PDA Ch2 207nm

Index	Time/min	Height/mAU	Quantity/Area	Area %/%
1	17.151	6211	118382	4.785
2	19.021	97757	2355541	95.215

Catalyst III/TfOH results:

<Chromatogram>

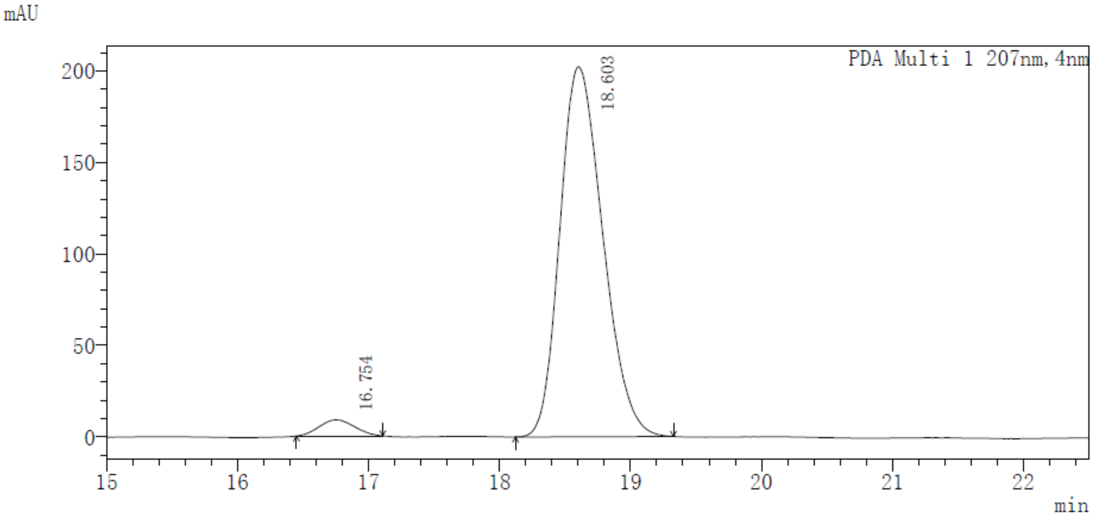

<Peak Results>

PDA Ch1 207nm

Index	Time/min	Height/mAU	Quantity/Area	Area %/%
1	16.754	8802	165086	3.456
2	18.603	202174	4612248	96.544

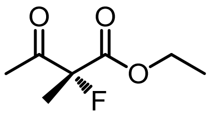

3b

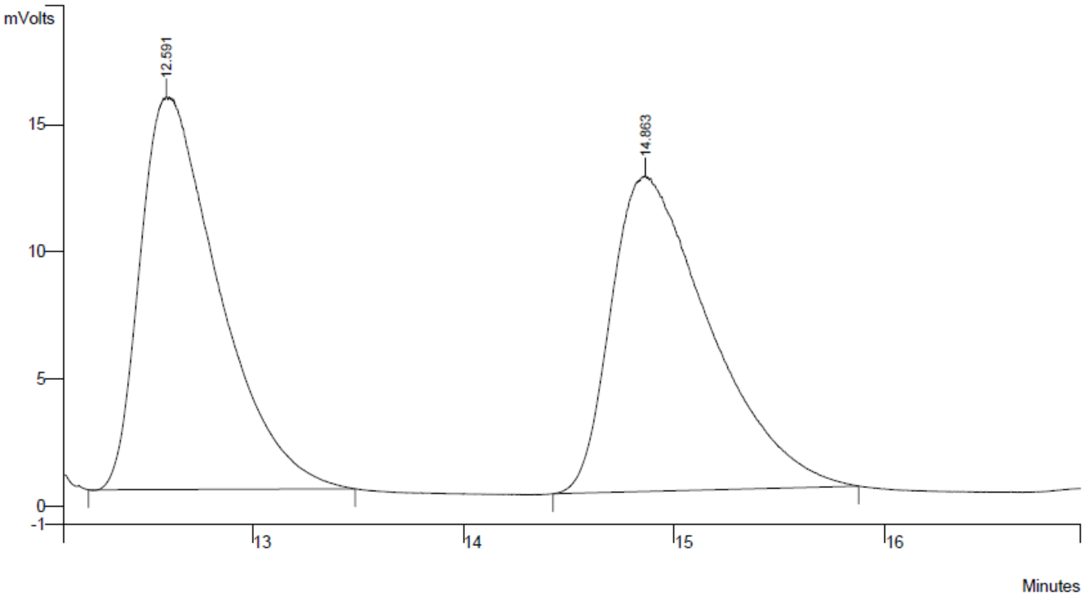

Peak No	Peak Name	Result ()	Ret. Time (min)	Time Offset (min)	Area (counts)	Rel Ret Time	Sep. Code	Width 1/2 (sec)
1		49.7897	12.591	0.000	401106	0.00	BB	23.7
2		50.2103	14.863	0.000	404494	0.00	BB	30.2
	**Totals**	**100.0000**		**0.000**	**805600**			

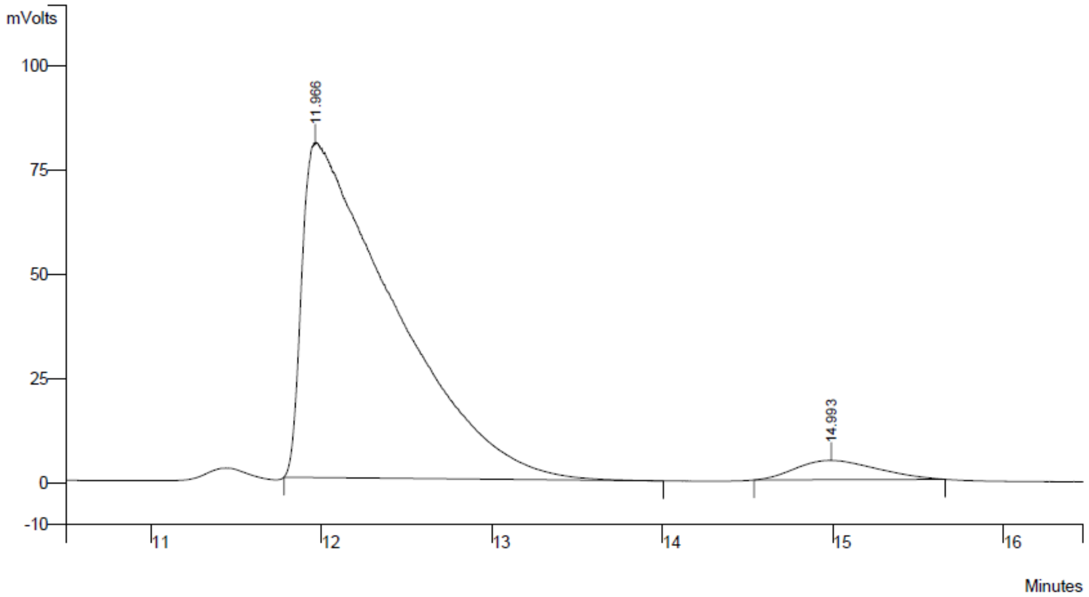

Peak No	Peak Name	Result ()	Ret. Time (min)	Time Offset (min)	Area (counts)	Rel Ret Time	Sep. Code	Width 1/2 (sec)
1		95.2011	11.966	0.000	3022896	0.00	BB	34.0
2		4.7989	14.993	0.000	152380	0.00	BB	32.3
	**Totals**	**100.0000**		**0.000**	**3175276**			

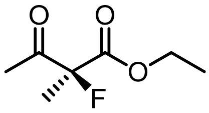

3b

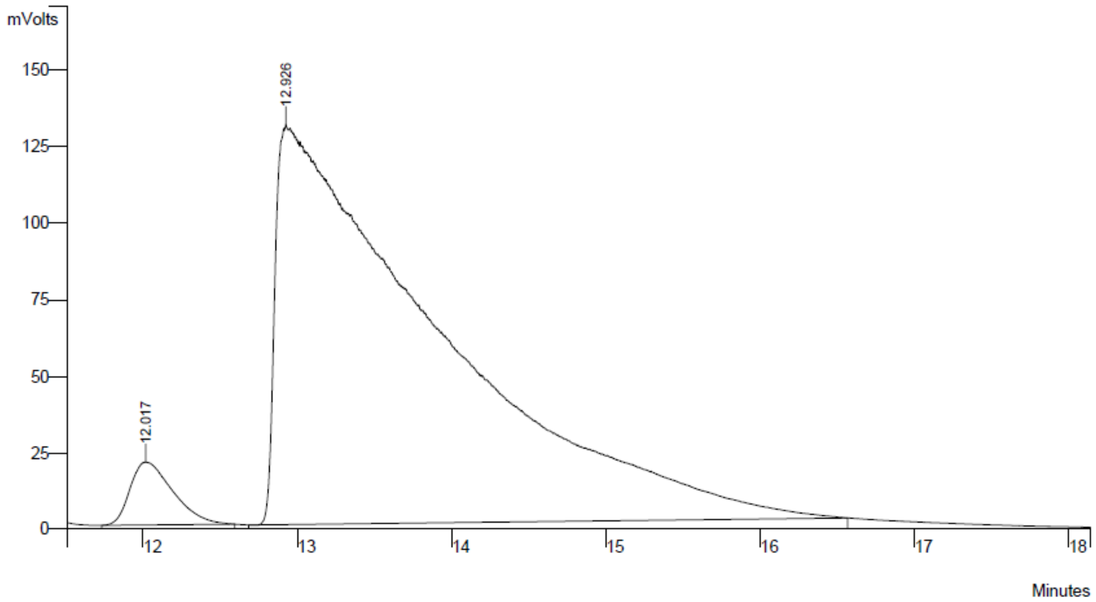

Peak No	Peak Name	Result ()	Ret. Time (min)	Time Offset (min)	Area (counts)	Rel Ret Time	Sep. Code	Width 1/2 (sec)
1		3.9767	12.017	0.000	395009	0.00	BB	18.0
2		96.0233	12.926	0.000	9538172	0.00	BB	62.1
	**Totals**	**100.0000**		**0.000**	**9933181**			

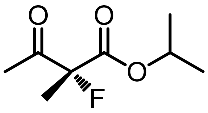

3c

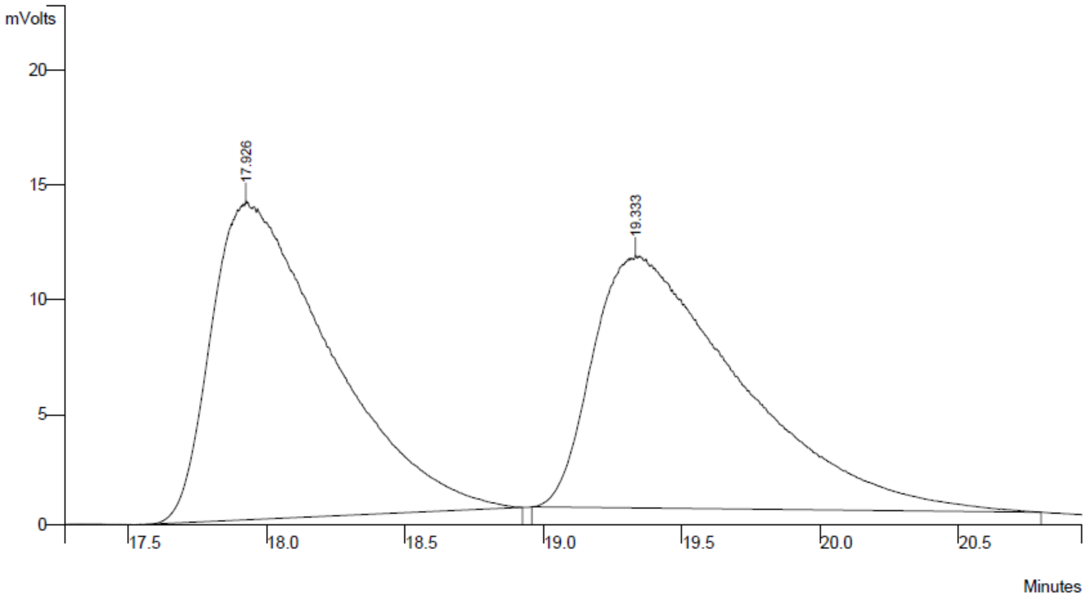

Peak No	Peak Name	Result ()	Ret. Time (min)	Time Offset (min)	Area (counts)	Rel Ret Time	Sep. Code	Width 1/2 (sec)
1		50.4155	17.926	0.000	414358	0.00	BB	28.1
2		49.5845	19.333	0.000	407529	0.00	BB	33.0
	**Totals**	**100.0000**		**0.000**	**821887**			

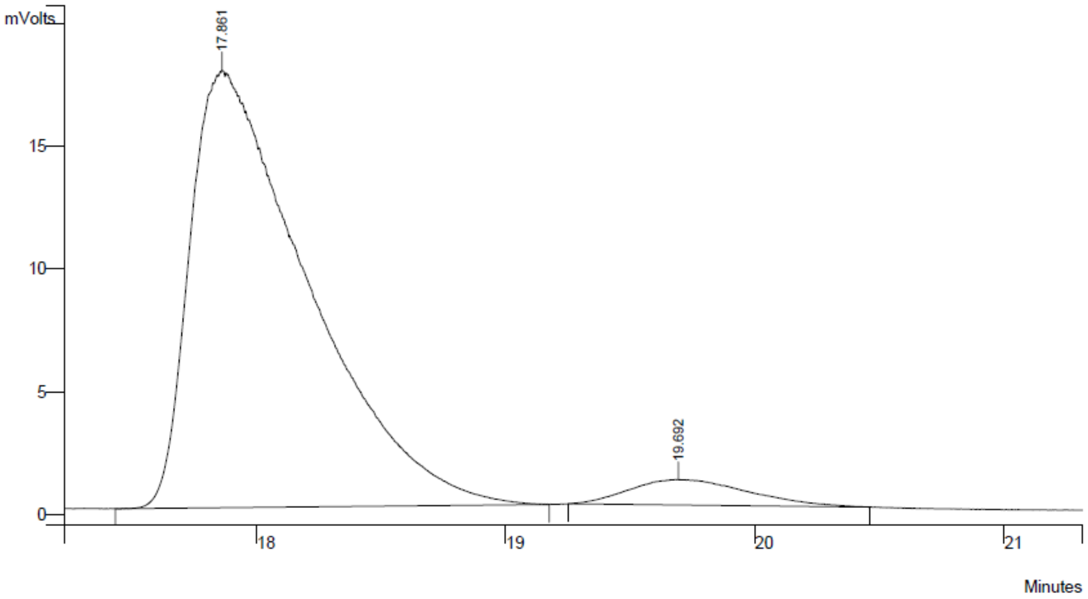

Peak No	Peak Name	Result ()	Ret. Time (min)	Time Offset (min)	Area (counts)	Rel Ret Time	Sep. Code	Width 1/2 (sec)
1		94.4826	17.861	0.000	584734	0.00	BB	30.2
2		5.5174	19.692	0.000	34146	0.00	BB	30.2
	**Totals**	**100.0000**		**0.000**	**618880**			

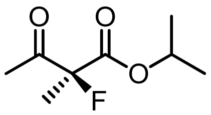

3c

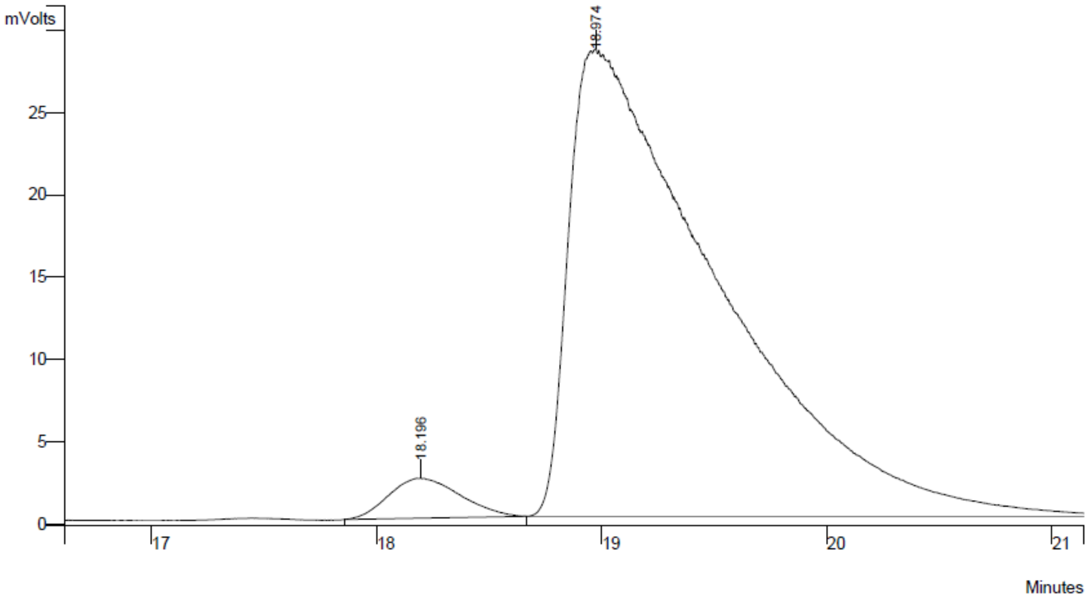

Peak No	Peak Name	Result ()	Ret. Time (min)	Time Offset (min)	Area (counts)	Rel Ret Time	Sep. Code	Width 1/2 (sec)
1		3.9009	18.196	0.000	53280	0.00	BB	21.8
2		96.0991	18.974	0.000	1312568	0.00	BB	40.7
	**Totals**	**100.0000**		**0.000**	**1365848**			

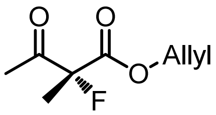

3d

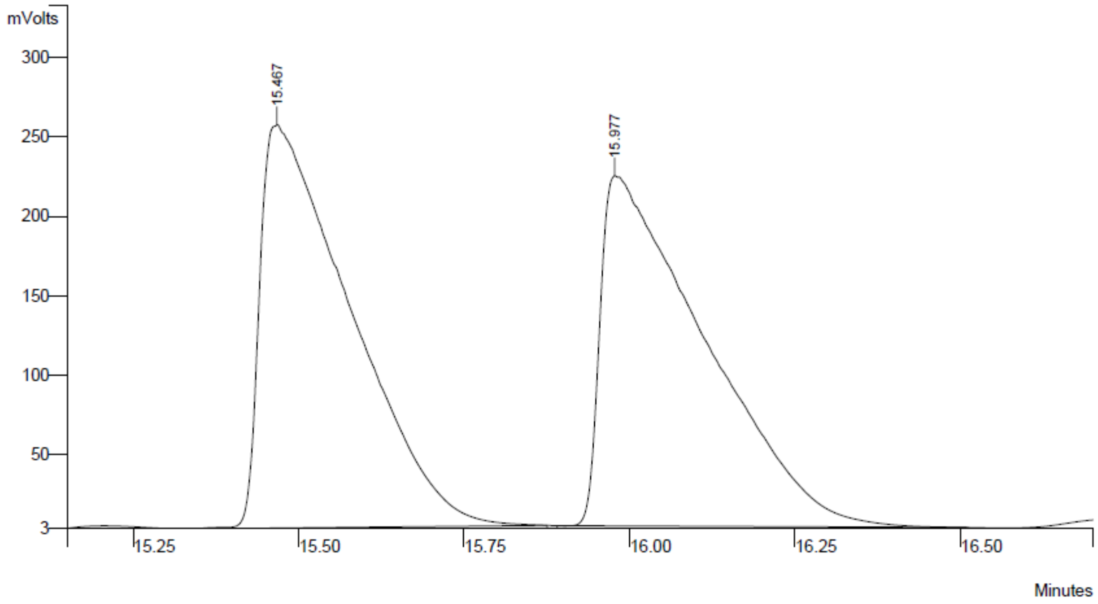

Peak No	Peak Name	Result ()	Ret. Time (min)	Time Offset (min)	Area (counts)	Rel Ret Time	Sep. Code	Width 1/2 (sec)
1		49.9893	15.467	0.000	2414180	0.00	BB	9.0
2		50.0107	15.977	0.000	2415211	0.00	BB	10.2
	**Totals**	**100.0000**		**0.000**	**4829391**			

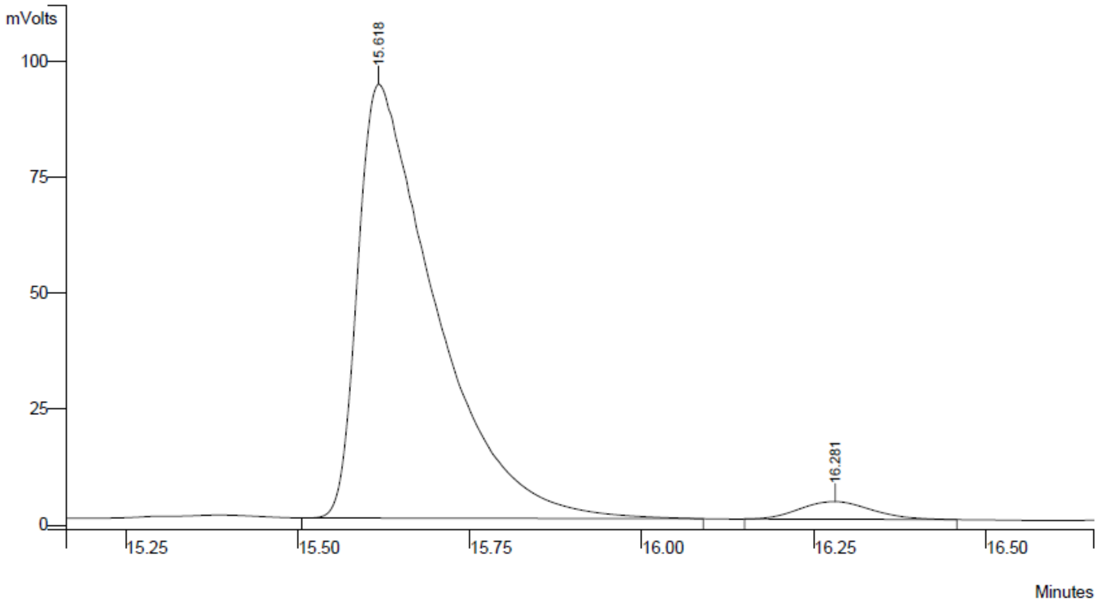

Peak No	Peak Name	Result ()	Ret. Time (min)	Time Offset (min)	Area (counts)	Rel Ret Time	Sep. Code	Width 1/2 (sec)
1		96.3898	15.618	0.000	735348	0.00	BB	6.9
2		3.6102	16.281	0.000	27542	0.00	BB	6.7
	**Totals**	**100.0000**		**0.000**	**762890**			

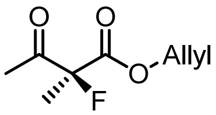

3d

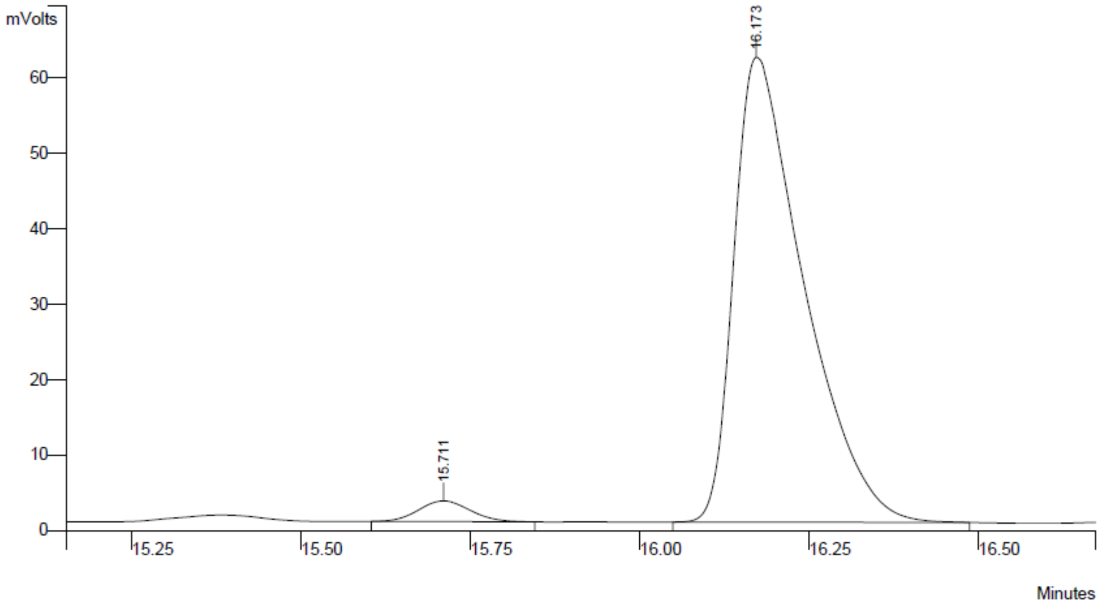

Peak No	Peak Name	Result ()	Ret. Time (min)	Time Offset (min)	Area (counts)	Rel Ret Time	Sep. Code	Width 1/2 (sec)
1		3.1785	15.711	0.000	14532	0.00	BB	4.9
2		96.8215	16.173	0.000	442658	0.00	BB	6.5
	**Totals**	**100.0000**		**0.000**	**457190**			

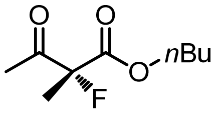

3e

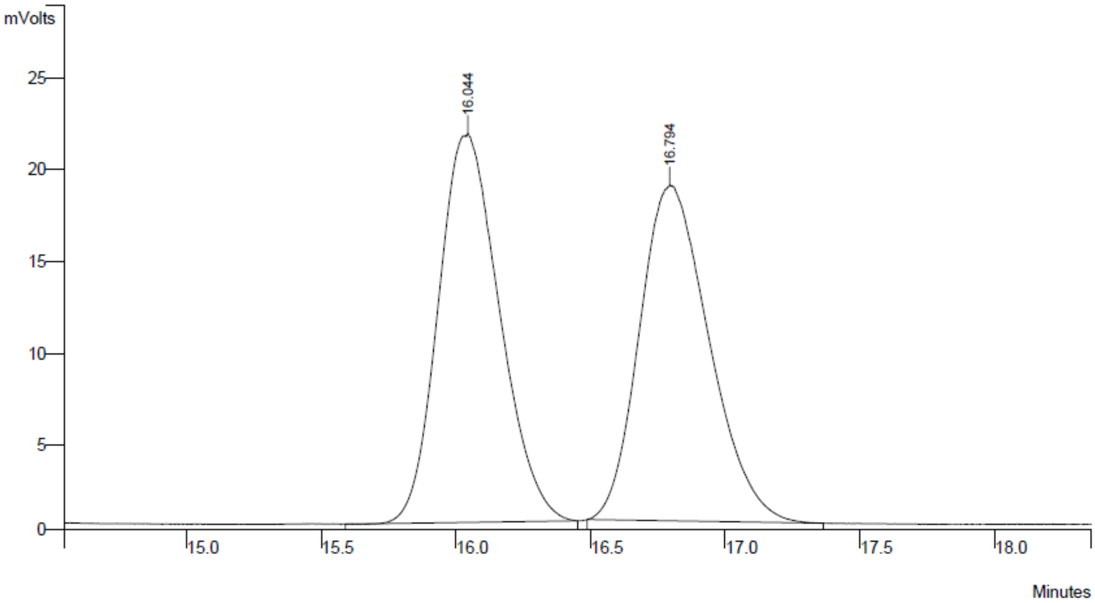

Peak No	Peak Name	Result ()	Ret. Time (min)	Time Offset (min)	Area (counts)	Rel Ret Time	Sep. Code	Width 1/2 (sec)
1		50.4548	16.044	0.000	334106	0.00	BB	14.9
2		49.5452	16.794	0.000	328084	0.00	BB	16.8
	**Totals**	**100.0000**		**0.000**	**662190**			

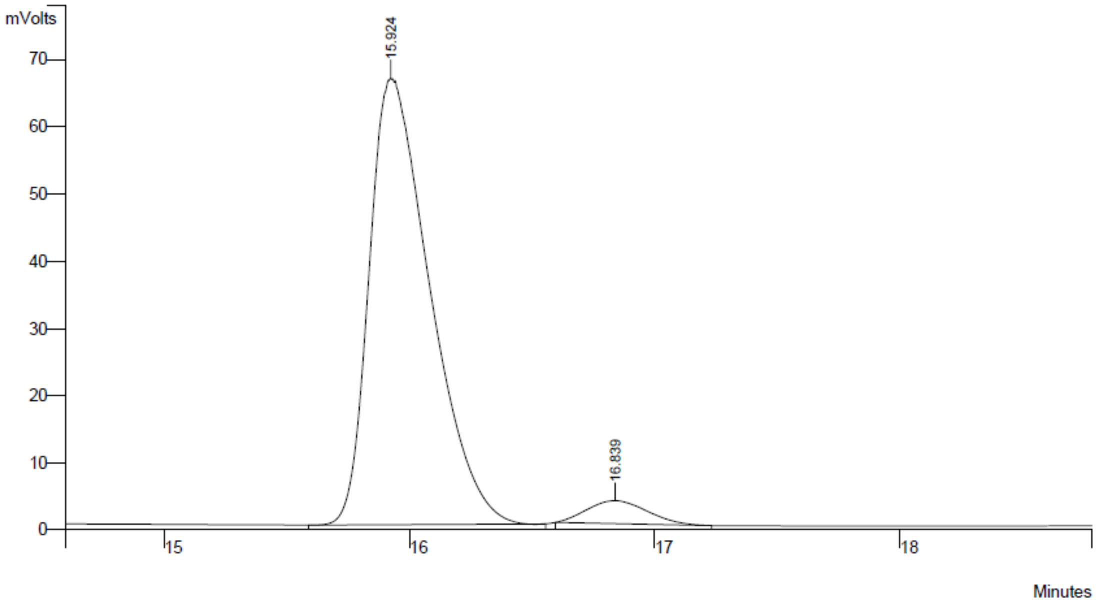

Peak No	Peak Name	Result ()	Ret. Time (min)	Time Offset (min)	Area (counts)	Rel Ret Time	Sep. Code	Width 1/2 (sec)
1		95.1248	15.924	0.000	1138332	0.00	BB	15.9
2		4.8752	16.839	0.000	58341	0.00	BB	15.8
	**Totals**	**100.0000**		**0.000**	**1196673**			

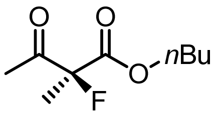

3e

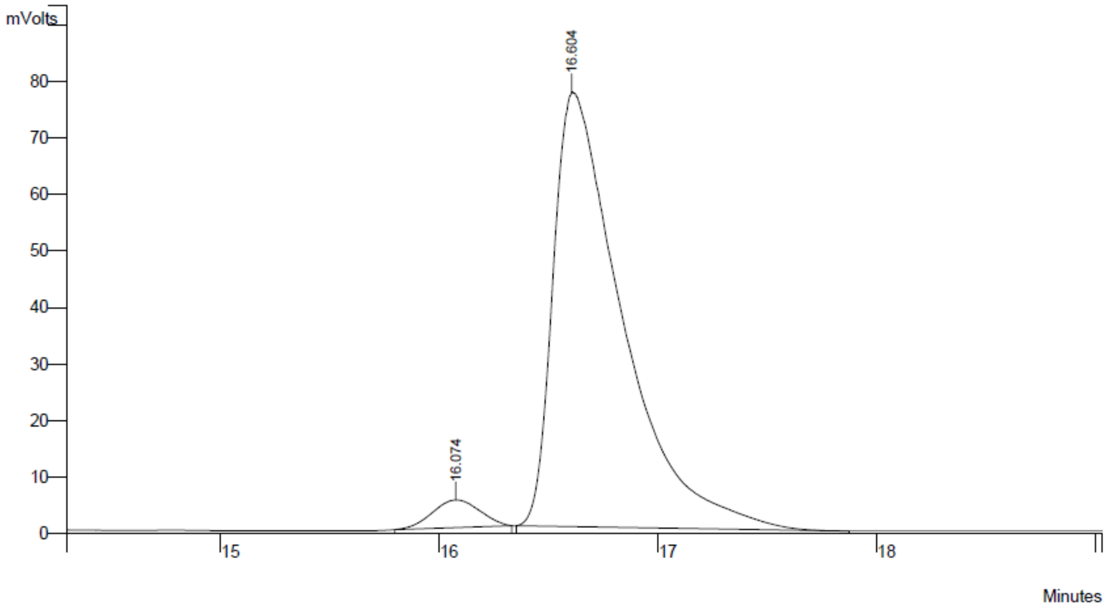

Peak No	Peak Name	Result ()	Ret. Time (min)	Time Offset (min)	Area (counts)	Rel Ret Time	Sep. Code	Width 1/2 (sec)
1		4.1455	16.074	0.000	73211	0.00	BB	15.3
2		95.8545	16.604	0.000	1692838	0.00	BB	19.1
	**Totals**	**100.0000**		**0.000**	**1766049**			

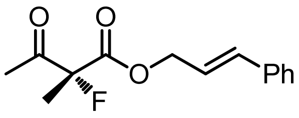

3f

〈Chromatogram〉

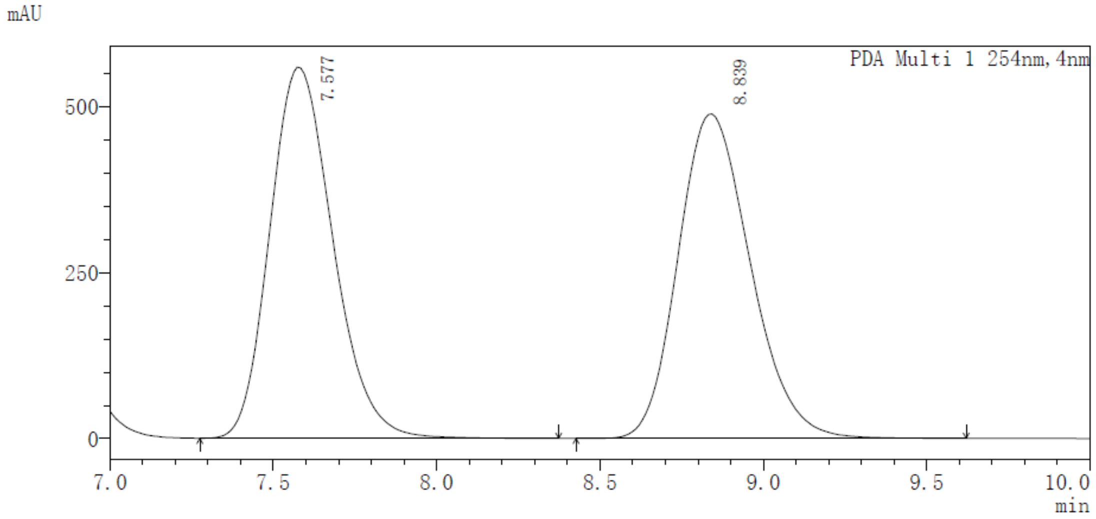

〈Peak Results〉

PDA Ch1 254nm

Index	Time/min	Height/mAU	Quantity/Area	Area %/%
1	7.577	559213	7515319	49.924
2	8.839	488963	7538153	50.076

〈Chromatogram〉

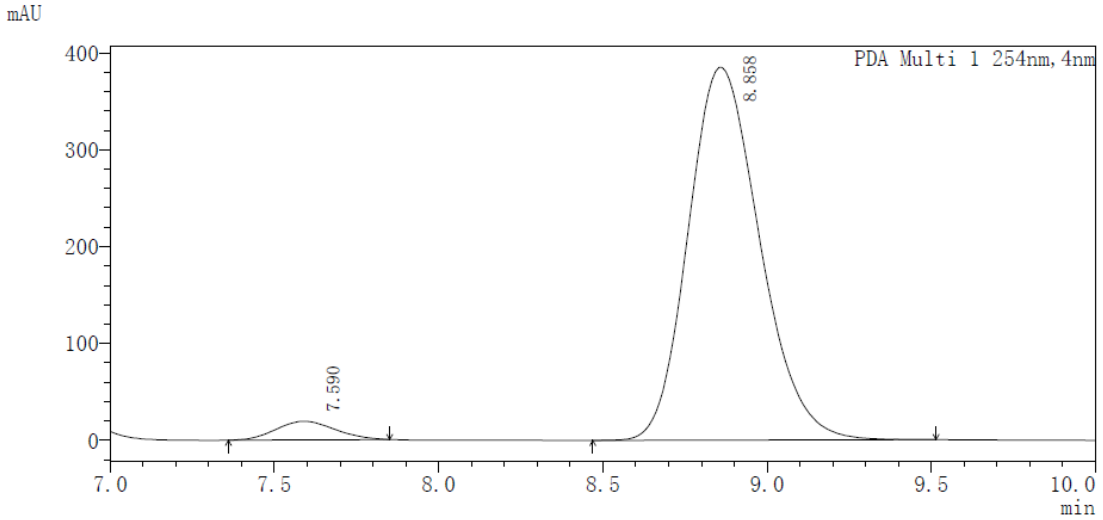

〈Peak Results〉

PDA Ch1 254nm

Index	Time/min	Height/mAU	Quantity/Area	Area %/%
1	7.590	19137	240105	3.957
2	8.858	384943	5828344	96.043

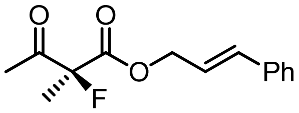

3f

〈Chromatogram〉

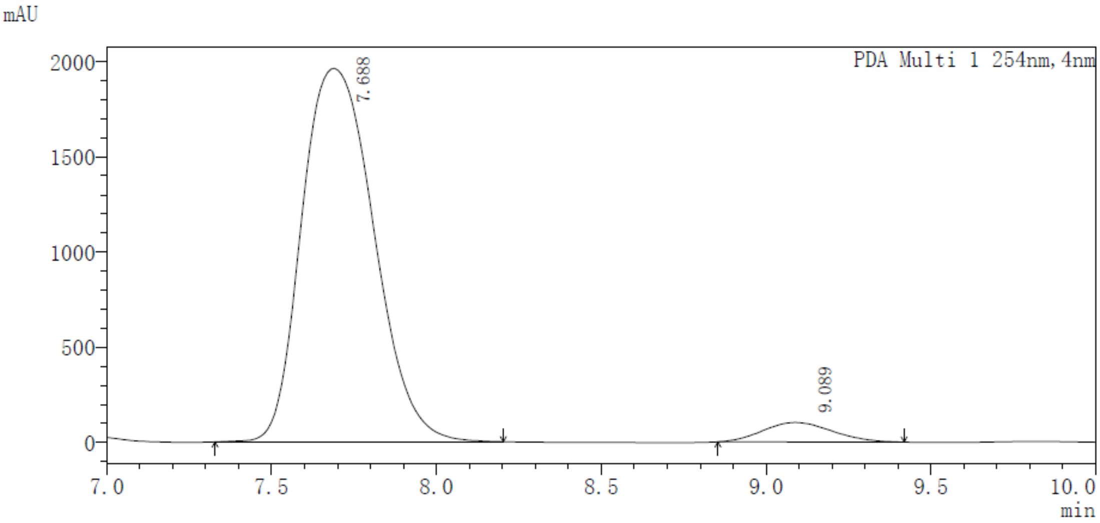

〈Peak Results〉

PDA Ch1 254nm

Index	Time/min	Height/mAU	Quantity/Area	Area %/%
1	7.688	1960147	29876088	95.206
2	9.089	102412	1504271	4.794

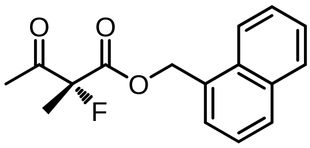

3g

〈Chromatogram〉

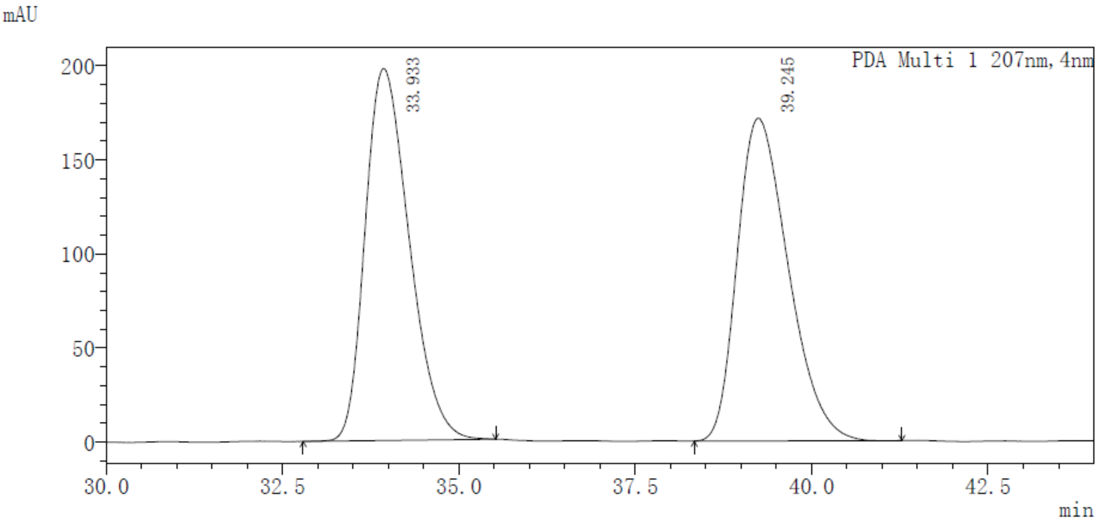

〈Peak Results〉

PDA Ch1 207nm

Index	Time/min	Height/mAU	Quantity/Area	Area %/%
1	33.933	197649	8594684	49.844
2	39.245	171553	8648405	50.156

〈Chromatogram〉

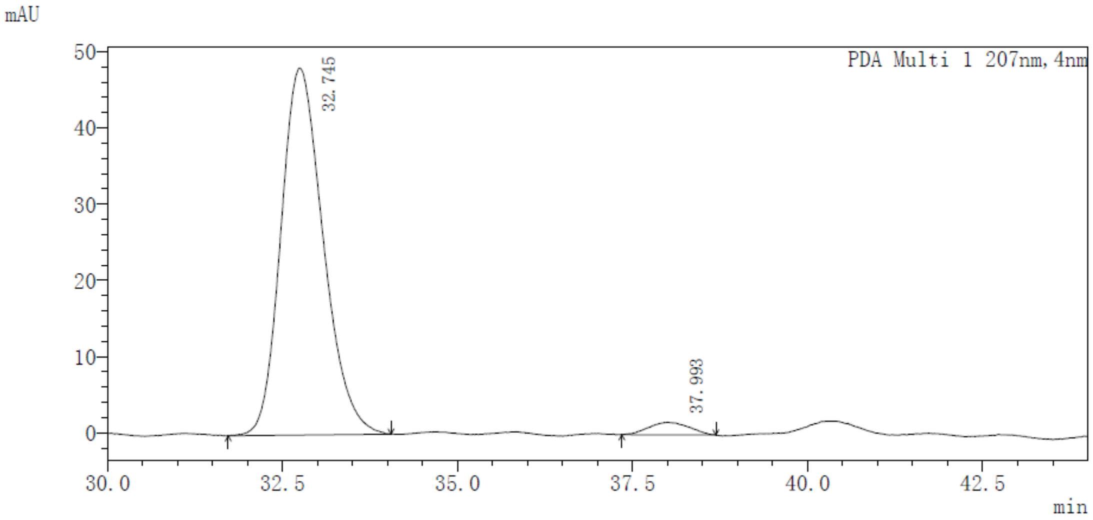

〈Peak Results〉

PDA Ch1 207nm

Index	Time/min	Height/mAU	Quantity/Area	Area %/%
1	32.745	48123	2027376	96.857
2	37.993	1641	65794	3.143

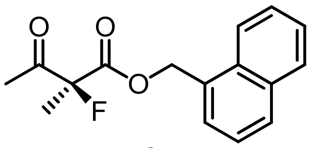

3g

Catalyst III/TfOH results:

<Chromatogram>

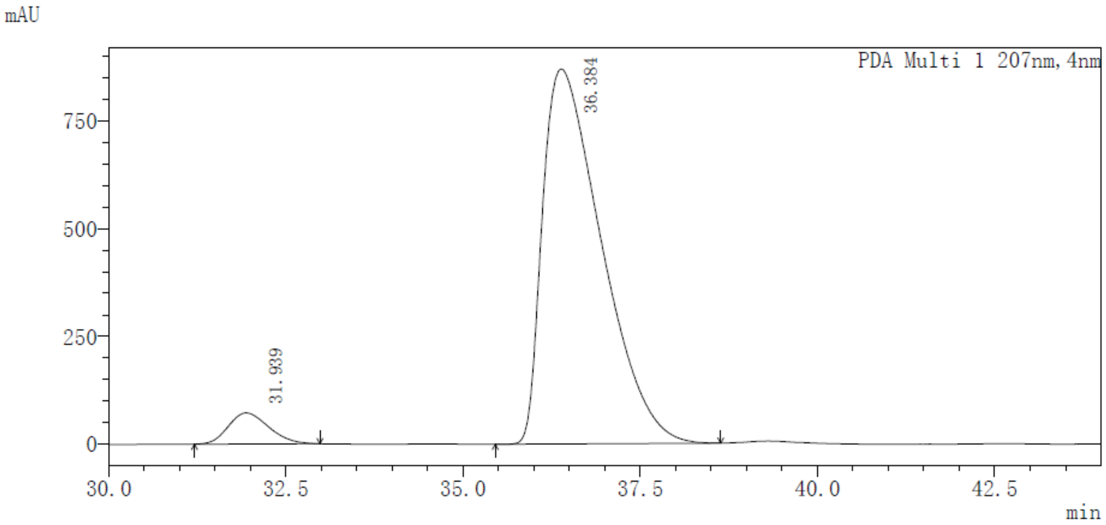

<Peak Results>

PDA Ch1 207nm

Index	Time/min	Height/mAU	Quantity/Area	Area %/%
1	31.939	71970	2862110	5.384
2	36.384	869758	50296468	94.616

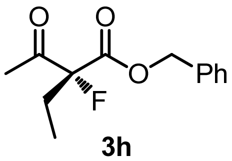

3h

〈Chromatogram〉

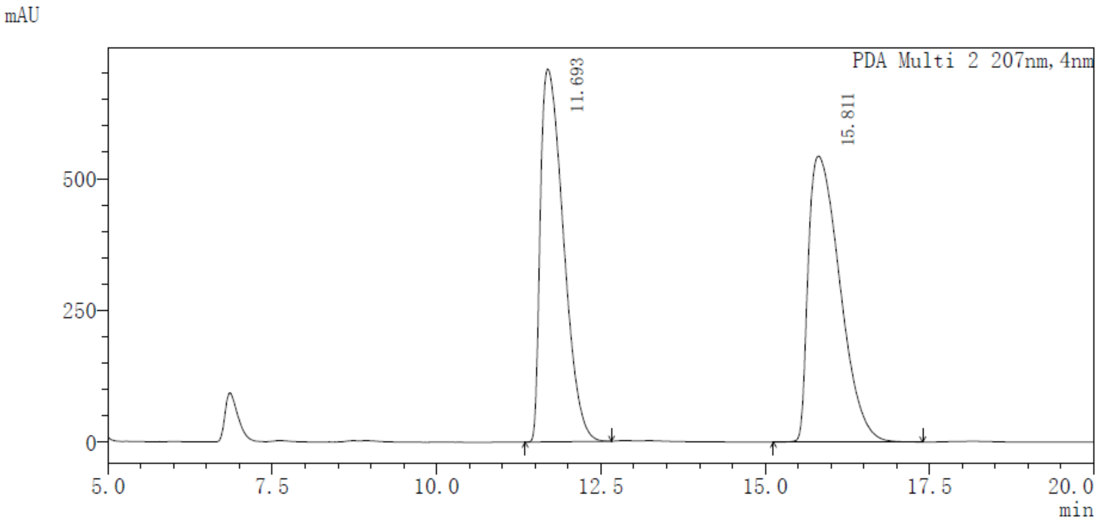

〈Peak Results〉

PDA Ch2 207nm

Index	Time/min	Height/mAU	Quantity/Area	Area %/%
1	11.693	707017	17214217	48.679
2	15.811	542277	18148478	51.321

〈Chromatogram〉

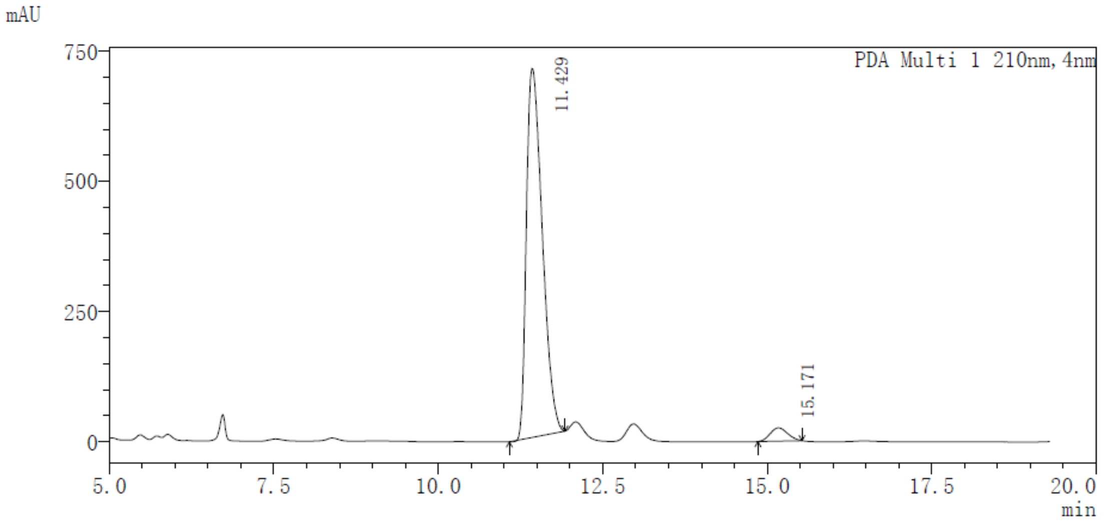

〈Peak Results〉

PDA Ch1 210nm

Index	Time/min	Height/mAU	Quantity/Area	Area %/%
1	11.429	707828	11890298	96.182
2	15.171	25487	471980	3.818

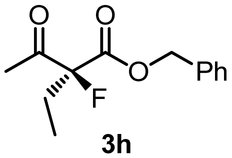

3h

Catalyst II/DNBA II results:

〈Chromatogram〉

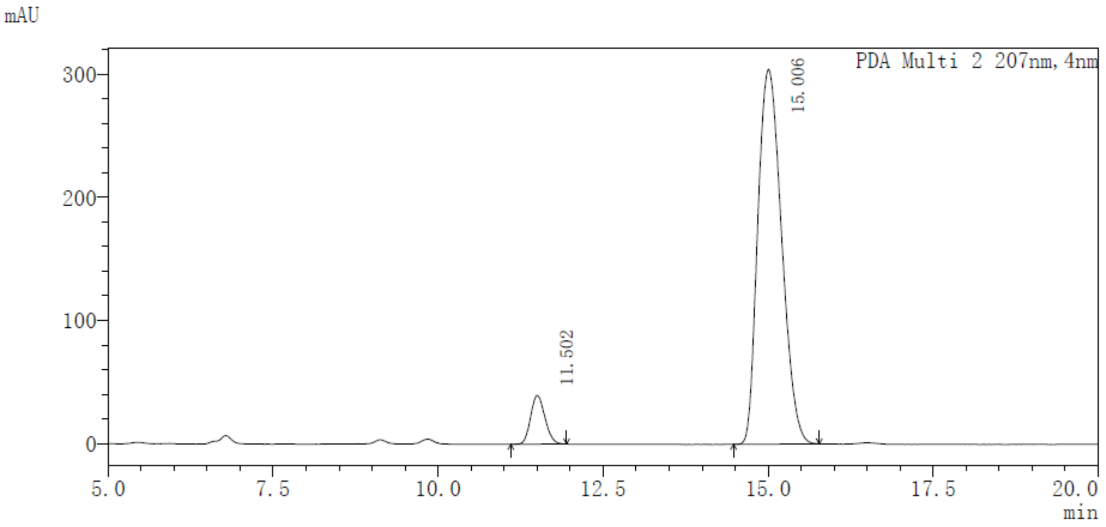

〈Peak Results〉

PDA Ch2 207nm

Index	Time/min	Height/mAU	Quantity/Area	Area %/%
1	11.502	39577	598646	7.266
2	15.006	304758	7640664	92.734

Catalyst III/TfOH results:

〈Chromatogram〉

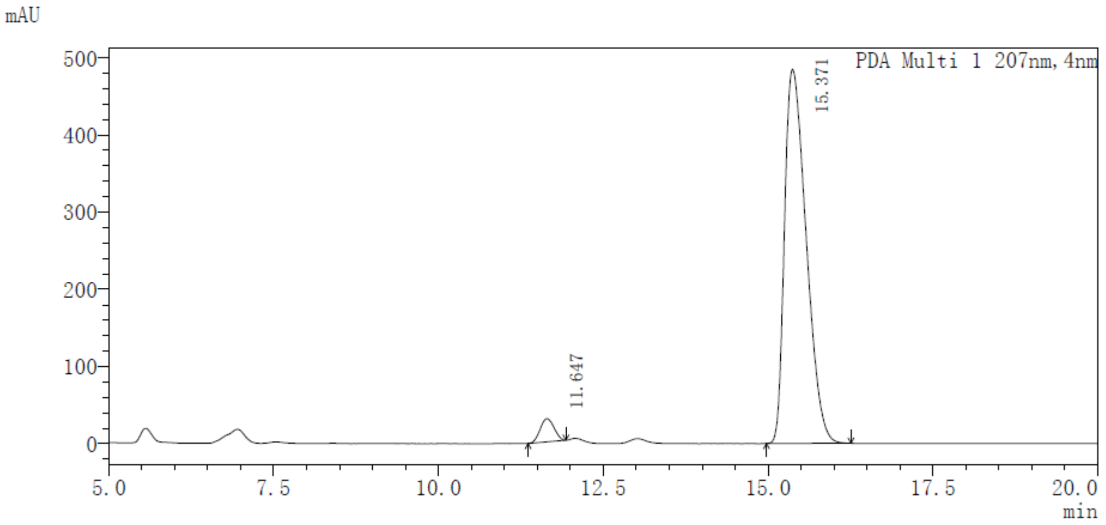

〈Peak Results〉

PDA Ch1 207nm

Index	Time/min	Height/mAU	Quantity/Area	Area %/%
1	11.647	30101	442554	3.847
2	15.371	484870	11061840	96.153

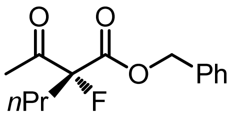

3i

〈Chromatogram〉

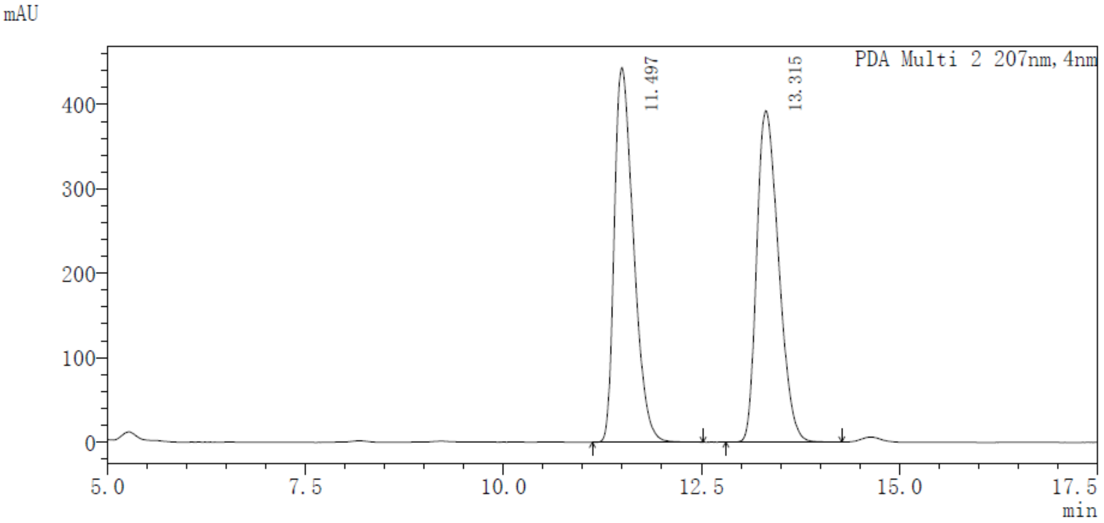

〈Peak Results〉

PDA Ch2 207nm

Index	Time/min	Height/mAU	Quantity/Area	Area %/%
1	11.497	443482	7489392	49.802
2	13.315	392265	7548808	50.198

〈Chromatogram〉

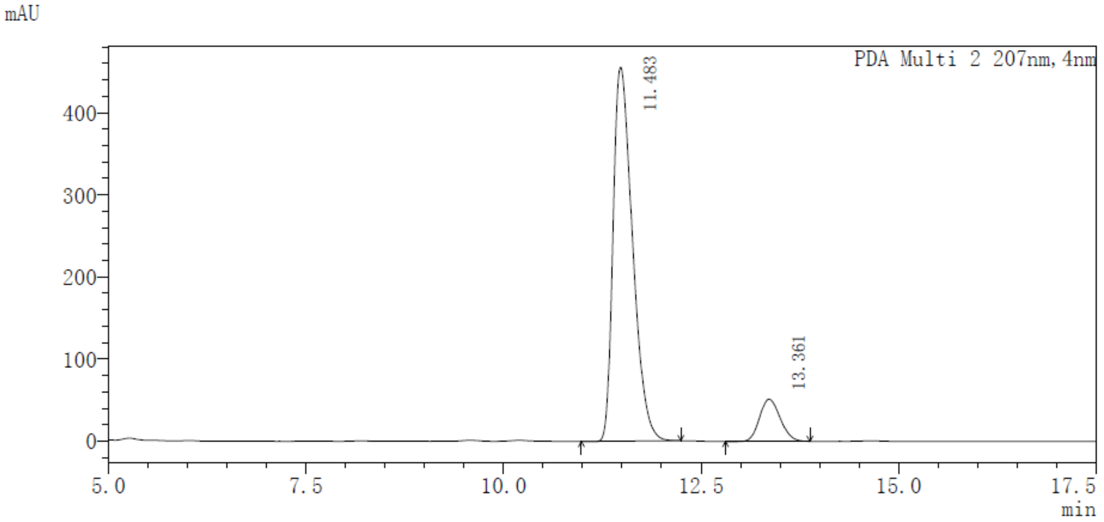

〈Peak Results〉

PDA Ch2 207nm

Index	Time/min	Height/mAU	Quantity/Area	Area %/%
1	11.483	455154	7790217	89.250
2	13.361	51557	938343	10.750

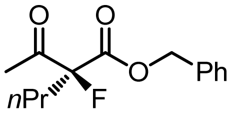

3i

Catalyst II/DNBA II results:

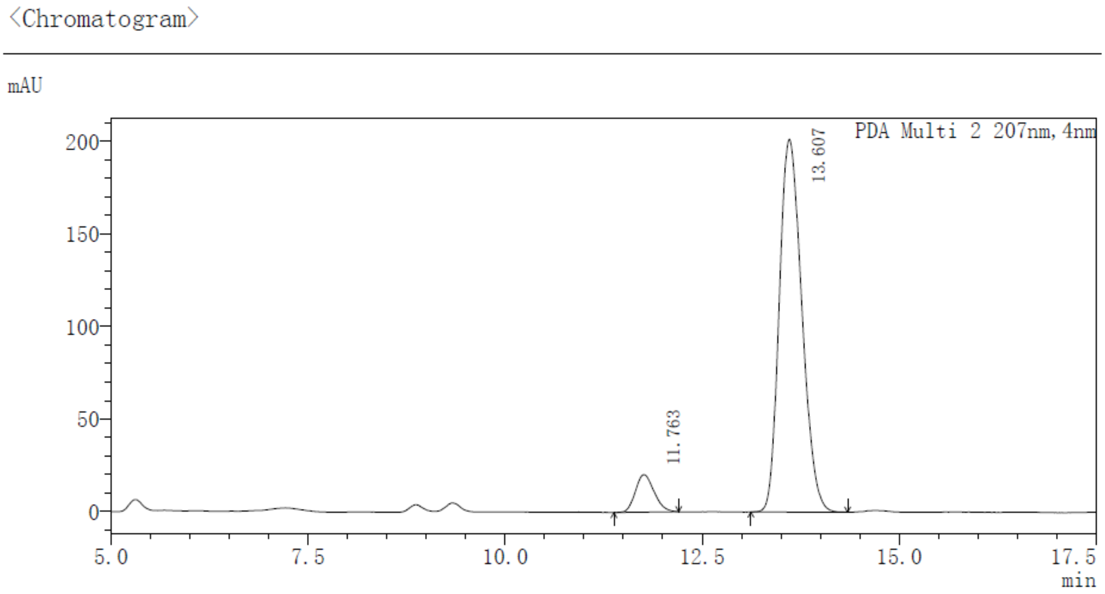

<Peak Results>
PDA Ch2 207nm

Index	Time/min	Height/mAU	Quantity/Area	Area %/%
1	11.763	20237	334500	7.684
2	13.607	201285	4018449	92.316

Catalyst III/TfOH results:

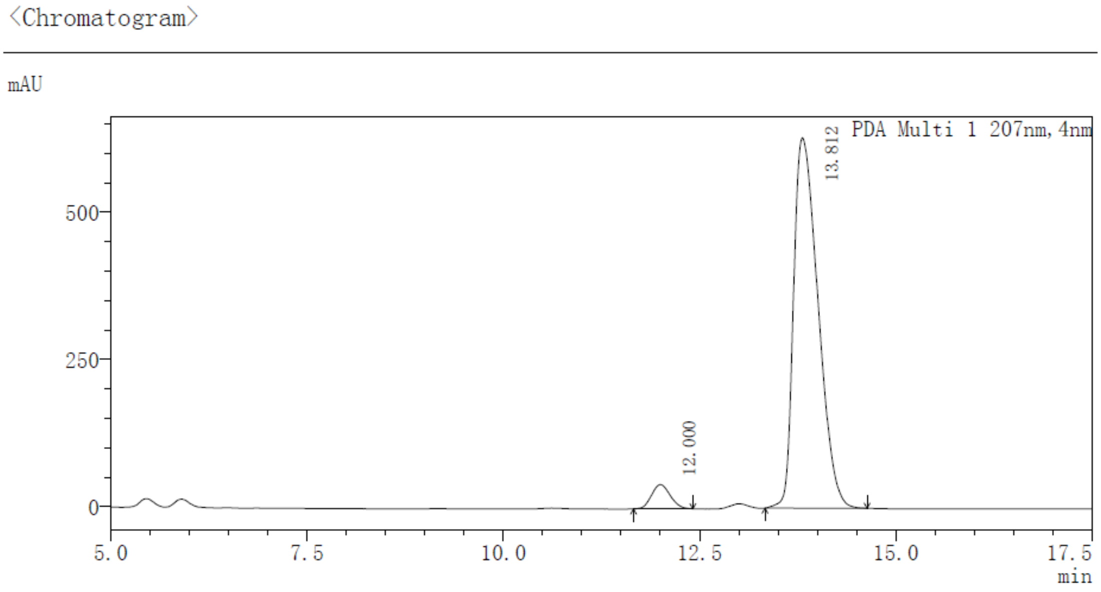

<Peak Results>
PDA Ch1 207nm

Index	Time/min	Height/mAU	Quantity/Area	Area %/%
1	12.000	41185	687886	4.804
2	13.812	629435	13632086	95.196

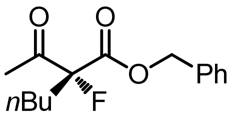

3j

〈Chromatogram〉

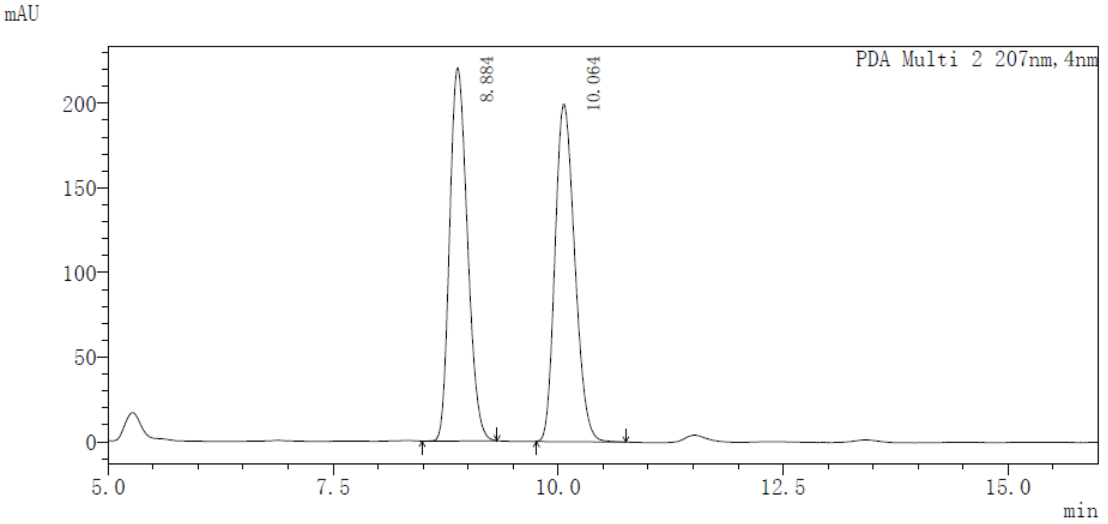

〈Peak Results〉

PDA Ch2 207nm

Index	Time/min	Height/mAU	Quantity/Area	Area %/%
1	8.884	220481	3028418	49.918
2	10.064	199346	3038319	50.082

〈Chromatogram〉

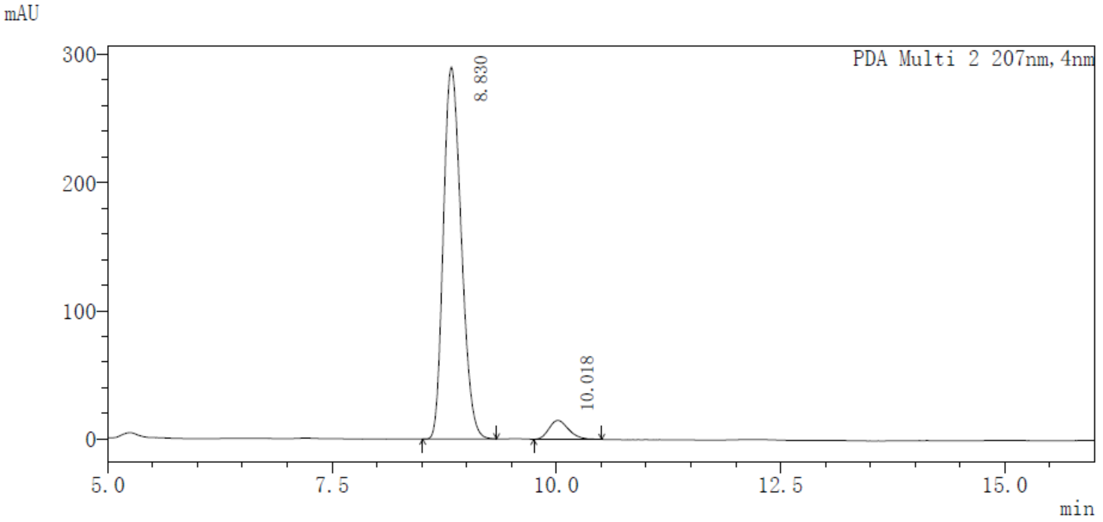

〈Peak Results〉

PDA Ch2 207nm

Index	Time/min	Height/mAU	Quantity/Area	Area %/%
1	8.830	290080	4007658	94.669
2	10.018	14764	225673	5.331

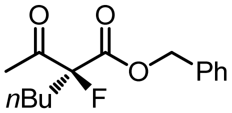

3j

Catalyst II/DNBA II results:

<Chromatogram>

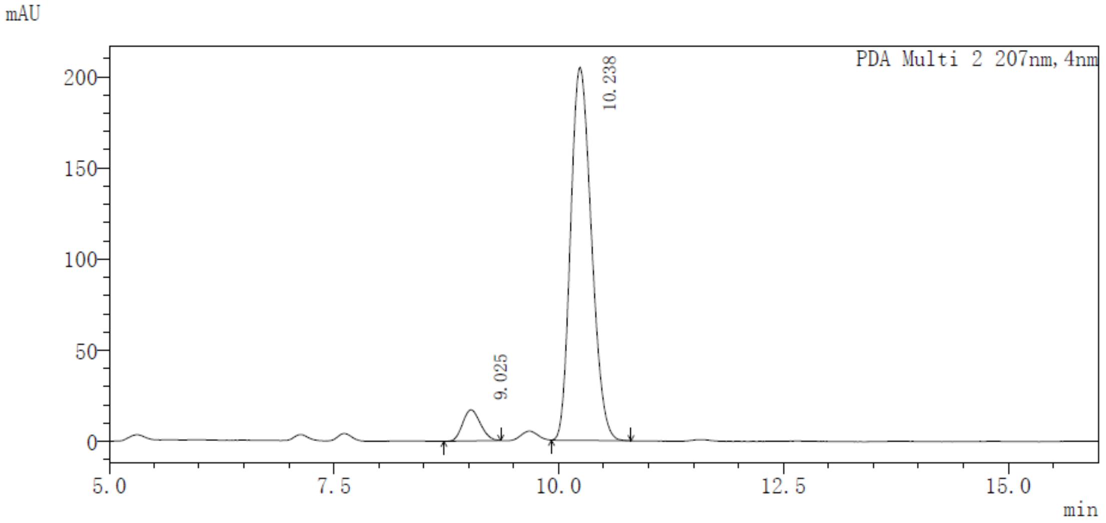

<Peak Results>

PDA Ch2 207nm

Index	Time/min	Height/mAU	Quantity/Area	Area %/%
1	9.025	17085	235130	6.667
2	10.238	204538	3291606	93.333

Catalyst III/TfOH results:

<Chromatogram>

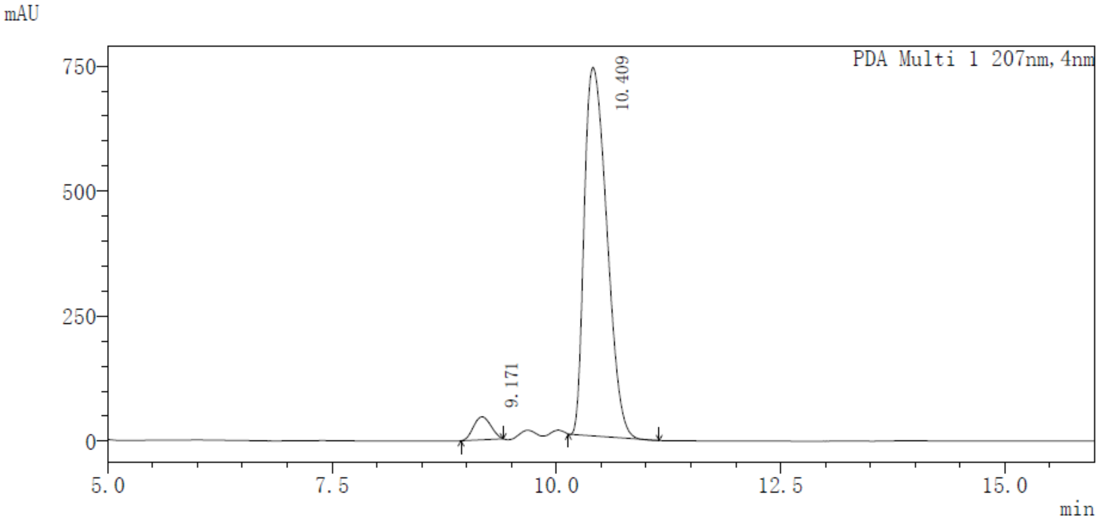

<Peak Results>

PDA Ch1 207nm

Index	Time/min	Height/mAU	Quantity/Area	Area %/%
1	9.171	45881	621215	4.609
2	10.409	736619	12857190	95.391

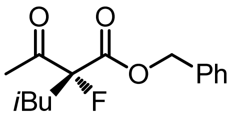

3k

〈Chromatogram〉

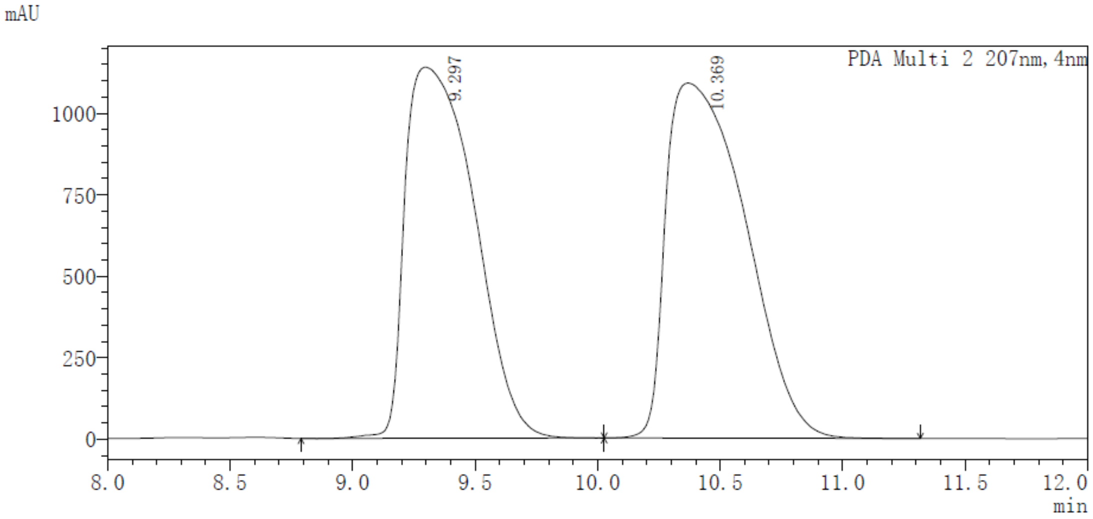

〈Peak Results〉

PDA Ch2 207nm

Index	Time/min	Height/mAU	Quantity/Area	Area %/%
1	9.297	1138941	22484723	47.652
2	10.369	1090014	24700151	52.348

〈Chromatogram〉

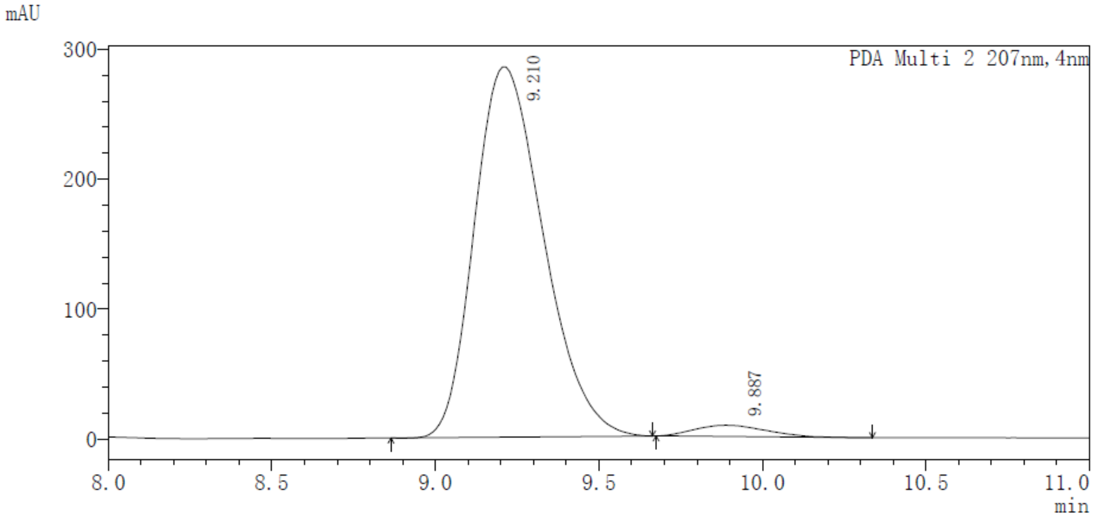

〈Peak Results〉

PDA Ch2 207nm

Index	Time/min	Height/mAU	Quantity/Area	Area %/%
1	9.210	284742	4190332	96.980
2	9.887	8598	130487	3.020

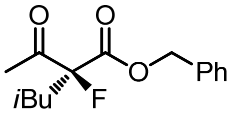

3k

Catalyst II/DNBA II results:

<Chromatogram>

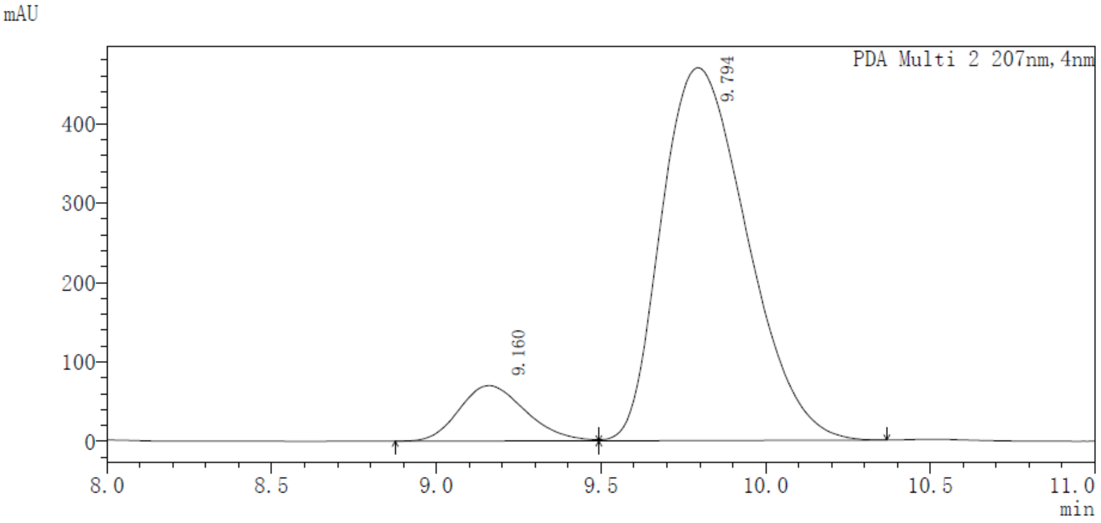

<Peak Results>

PDA Ch2 207nm

Index	Time/min	Height/mAU	Quantity/Area	Area %/%
1	9.160	69695	977038	10.314
2	9.794	469605	8496078	89.686

Catalyst III/TfOH results:

<Chromatogram>

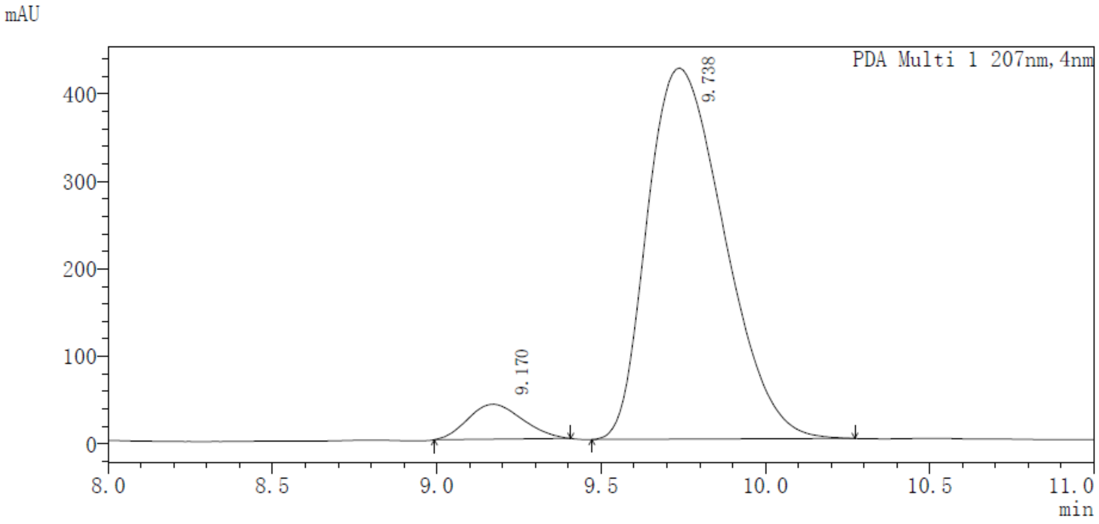

<Peak Results>

PDA Ch1 207nm

Index	Time/min	Height/mAU	Quantity/Area	Area %/%
1	9.170	39989	463474	6.278
2	9.738	424350	6919468	93.722

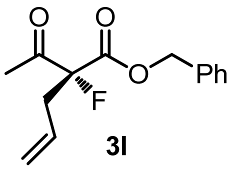

3l

〈Chromatogram〉

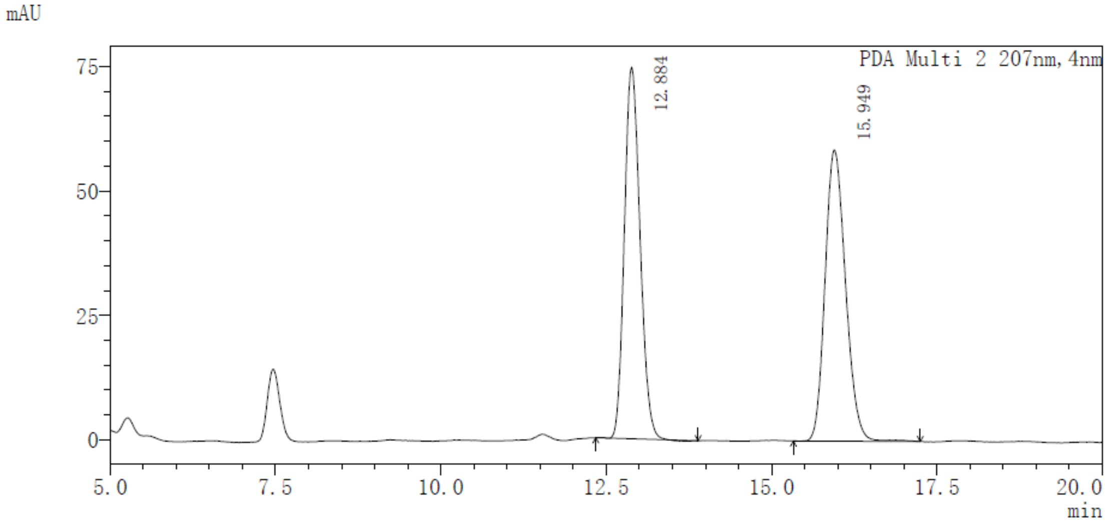

〈Peak Results〉

PDA Ch2 207nm

Index	Time/min	Height/mAU	Quantity/Area	Area %/%
1	12.884	74598	1229851	49.335
2	15.949	58520	1263029	50.665

〈Chromatogram〉

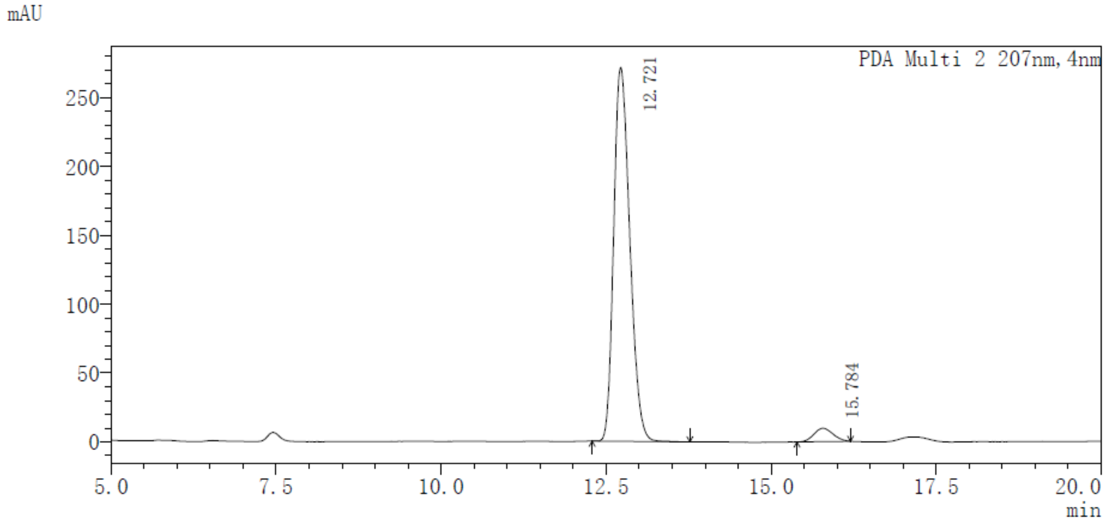

〈Peak Results〉

PDA Ch2 207nm

Index	Time/min	Height/mAU	Quantity/Area	Area %/%
1	12.721	271466	4613221	95.927
2	15.784	9760	195855	4.073

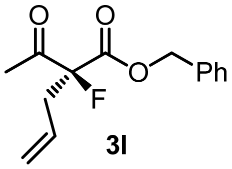

3l

Catalyst II/DNBA II results:

〈Chromatogram〉

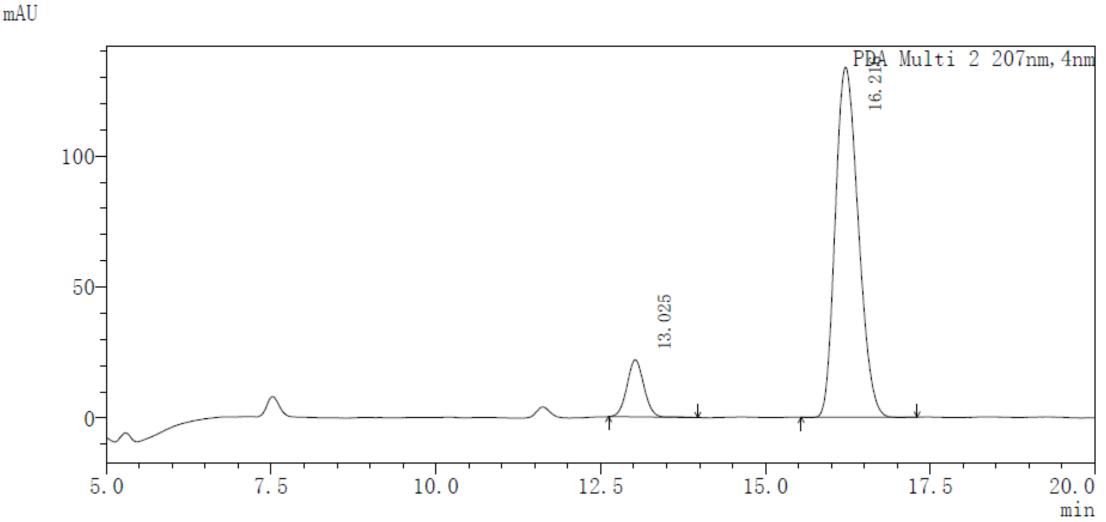

〈Peak Results〉

PDA Ch2 207nm

Index	Time/min	Height/mAU	Quantity/Area	Area %/%
1	13.025	21833	390690	10.752
2	16.216	133570	3242901	89.248

Catalyst III/TfOH results:

〈Chromatogram〉

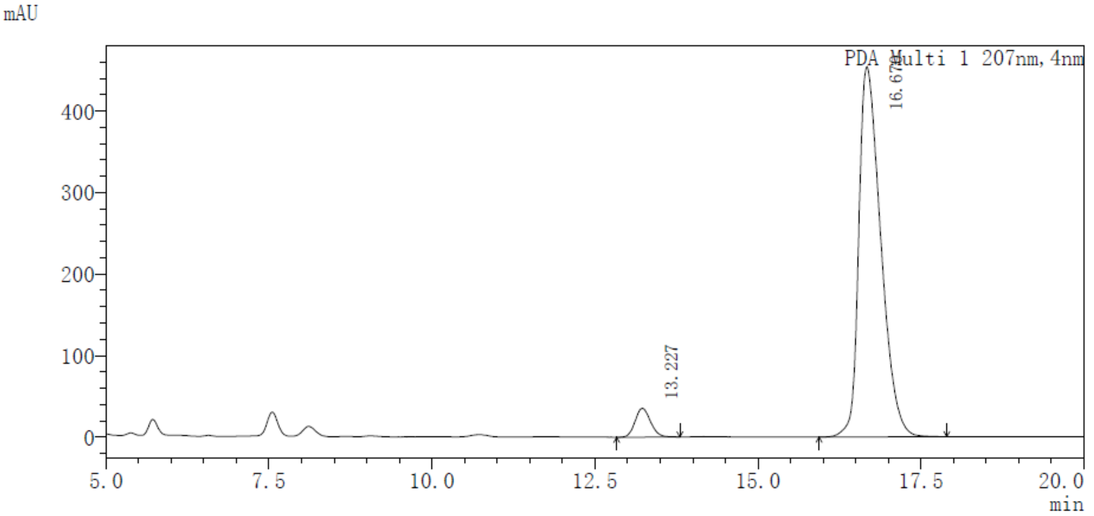

〈Peak Results〉

PDA Ch1 207nm

Index	Time/min	Height/mAU	Quantity/Area	Area %/%
1	13.227	35310	579236	5.158
2	16.670	453833	10651286	94.842

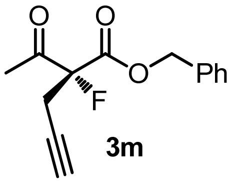

3m

⟨Chromatogram⟩

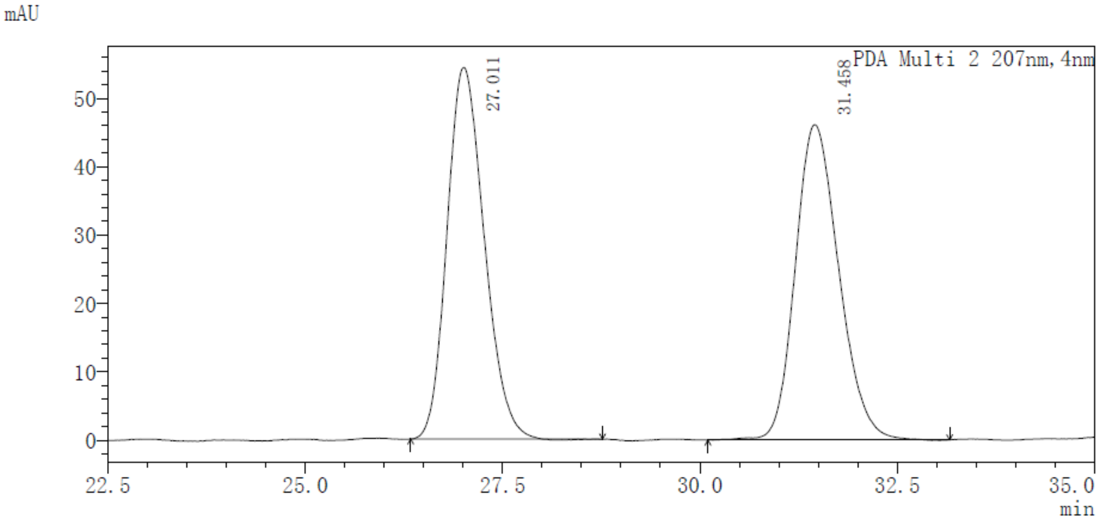

⟨Peak Results⟩

PDA Ch2 207nm

Index	Time/min	Height/mAU	Quantity/Area	Area %/%
1	27.011	54347	1782413	50.113
2	31.458	46150	1774357	49.887

⟨Chromatogram⟩

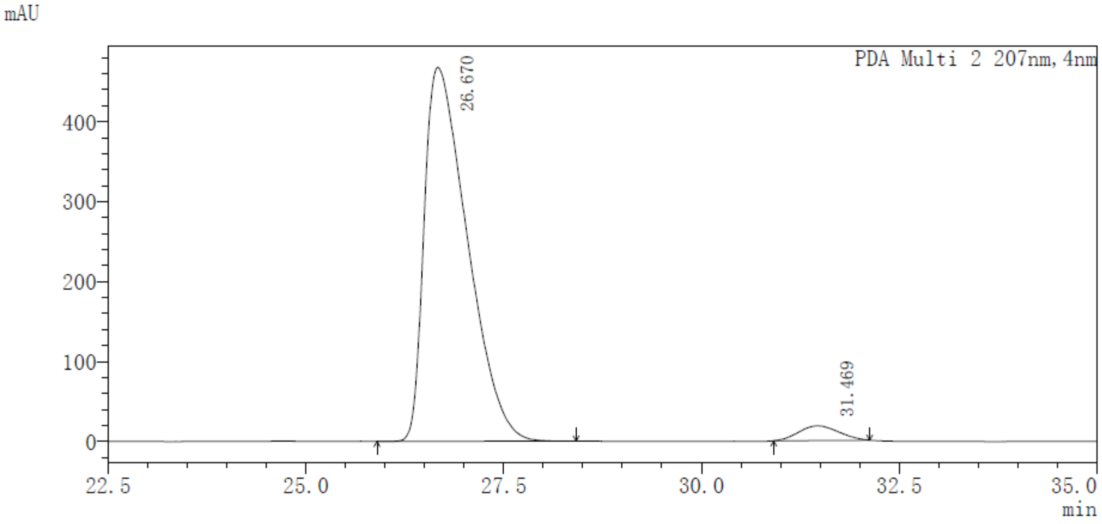

⟨Peak Results⟩

PDA Ch2 207nm

Index	Time/min	Height/mAU	Quantity/Area	Area %/%
1	26.670	467748	17704419	96.485
2	31.469	18422	645071	3.515

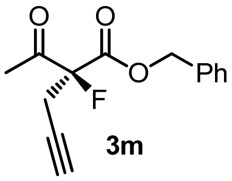

3m

Catalyst II/DNBA II results:

<Chromatogram>

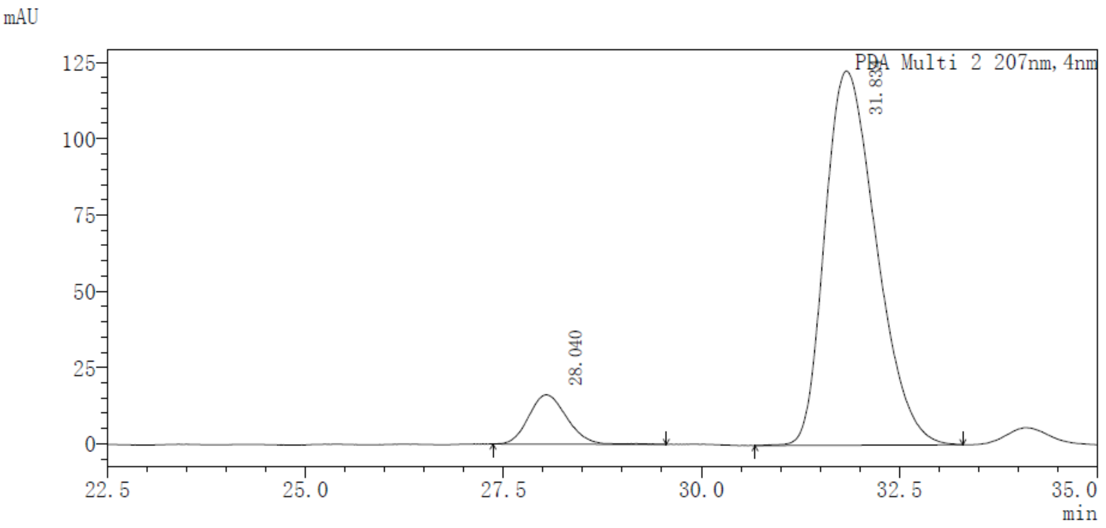

<Peak Results>

PDA Ch2 207nm

Index	Time/min	Height/mAU	Quantity/Area	Area %/%
1	28.040	16180	540258	8.897
2	31.834	122615	5532346	91.103

Catalyst III/TfOH results:

<Chromatogram>

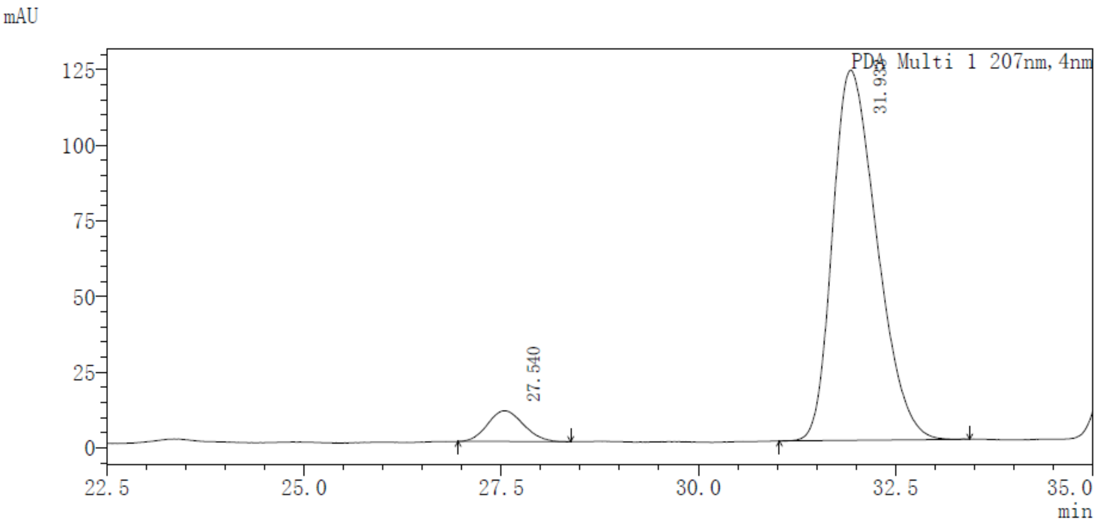

<Peak Results>

PDA Ch1 207nm

Index	Time/min	Height/mAU	Quantity/Area	Area %/%
1	27.540	10168	320421	6.269
2	31.933	122394	4790903	93.731

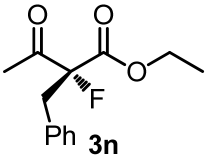

3n

〈Chromatogram〉

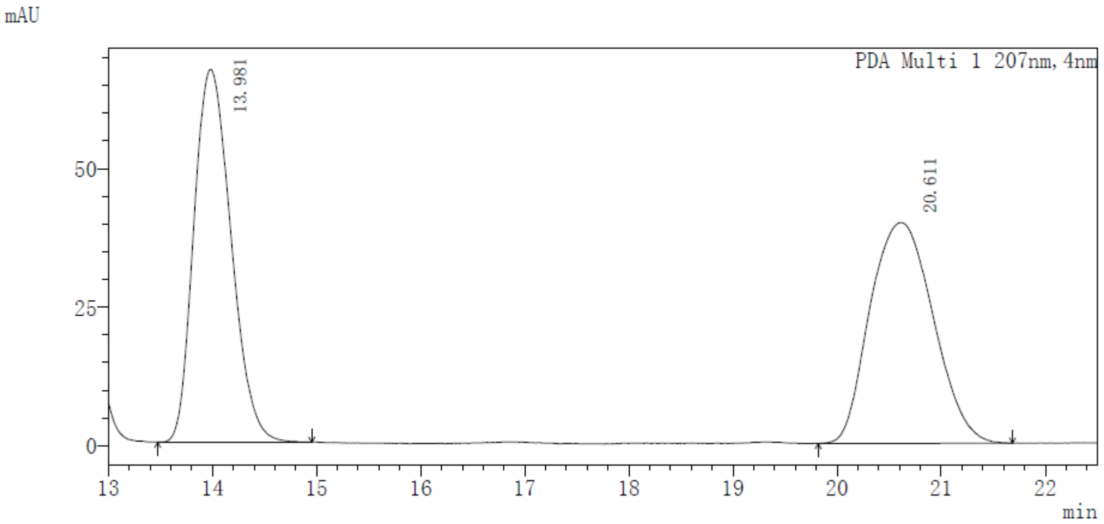

〈Peak Results〉

PDA Ch1 207nm

Index	Time/min	Height/mAU	Quantity/Area	Area %/%
1	13.981	67286	1674988	49.839
2	20.611	39843	1685823	50.161

〈Chromatogram〉

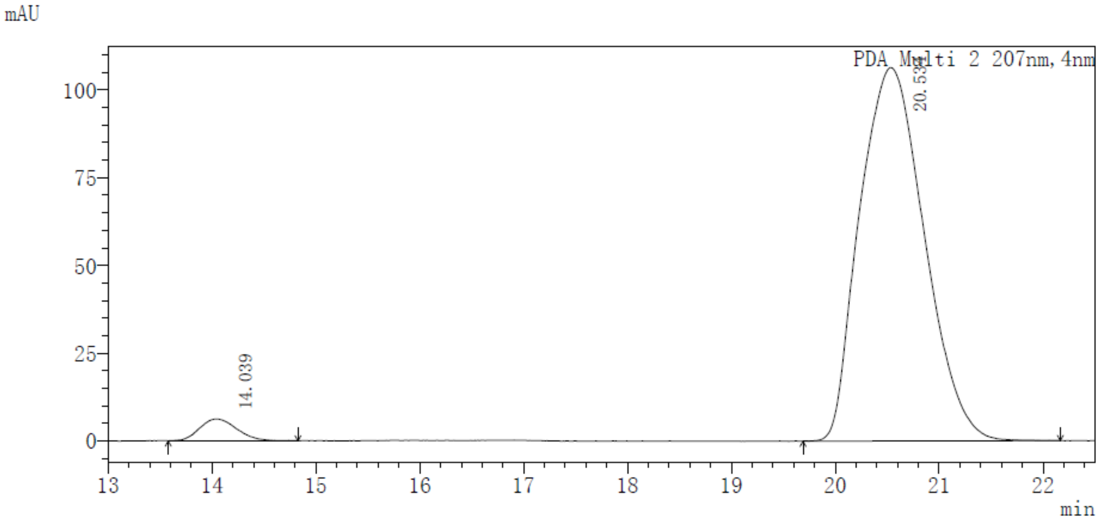

〈Peak Results〉

PDA Ch2 207nm

Index	Time/min	Height/mAU	Quantity/Area	Area %/%
1	14.039	6241	154566	3.248
2	20.534	106419	4604925	96.752

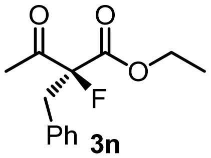

3n

Catalyst II/DNBA II results:

〈Chromatogram〉

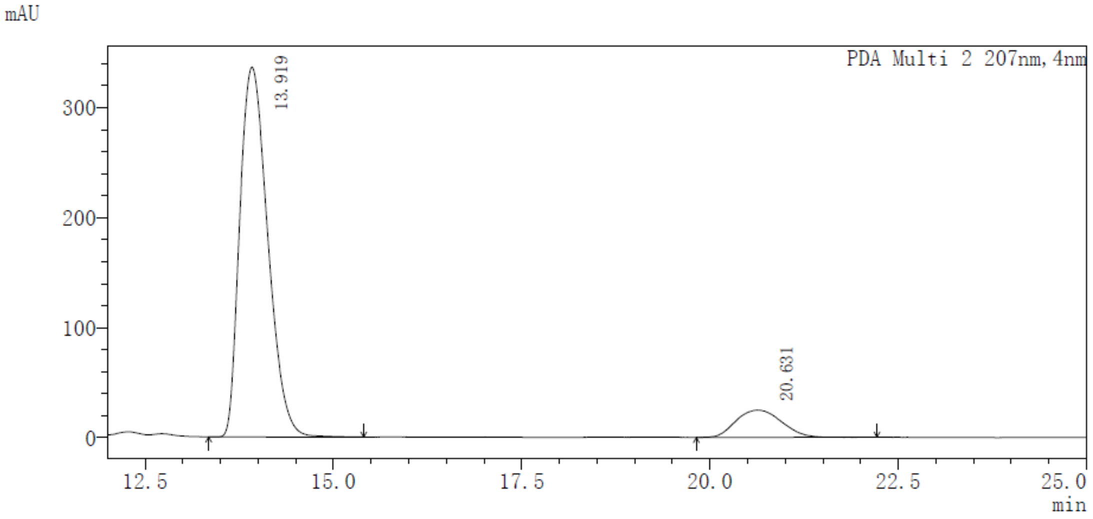

〈Peak Results〉

PDA Ch2 207nm

Index	Time/min	Height/mAU	Quantity/Area	Area %/%
1	13.919	336131	8687949	89.336
2	20.631	24720	1037116	10.664

Catalyst III/TfOH results:

〈Chromatogram〉

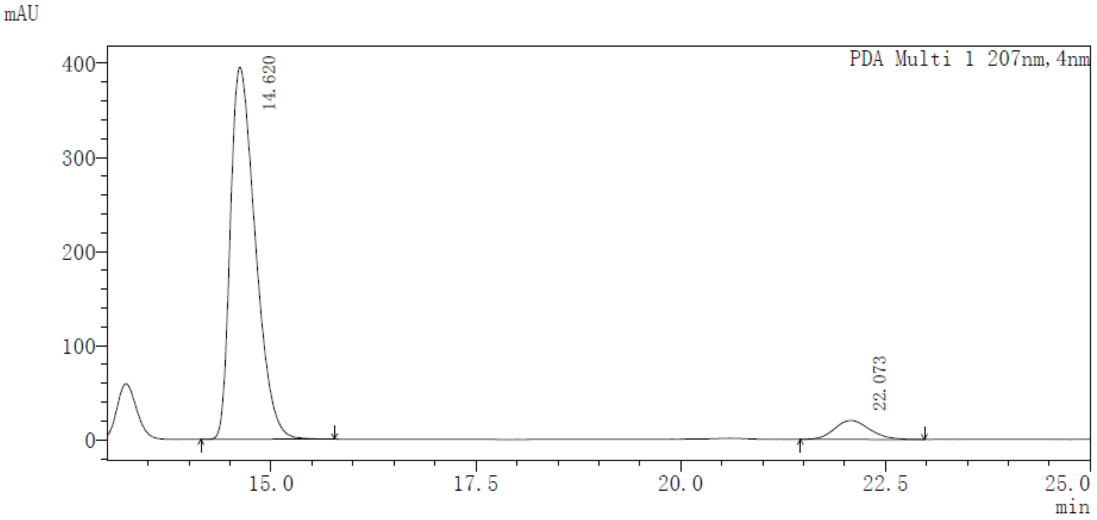

〈Peak Results〉

PDA Ch1 207nm

Index	Time/min	Height/mAU	Quantity/Area	Area %/%
1	14.620	395305	8383647	93.349
2	22.073	20141	597364	6.651

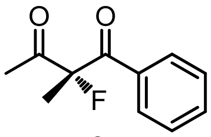

3o

〈Chromatogram〉

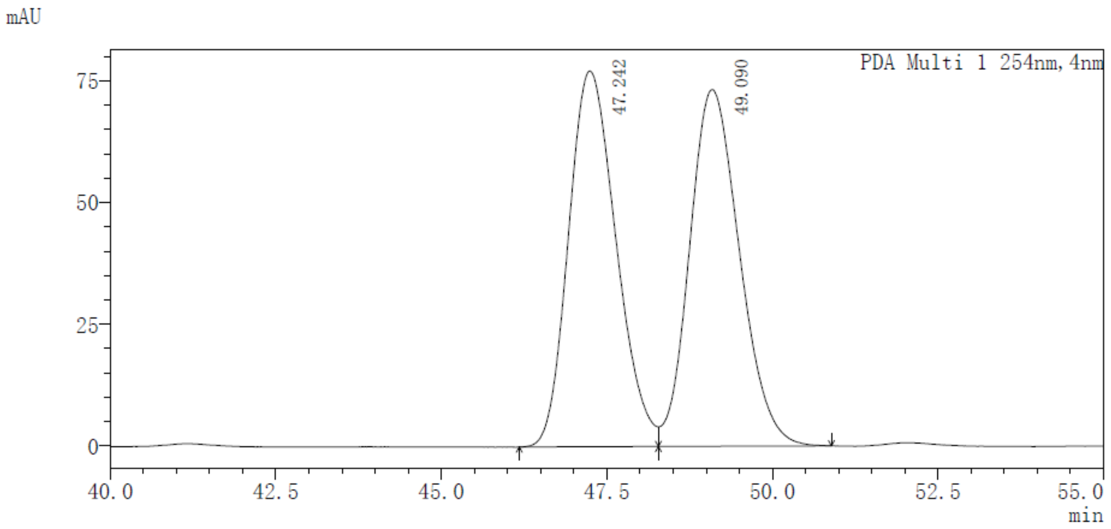

〈Peak Results〉

PDA Ch1 254nm

Index	Time/min	Height/mAU	Quantity/Area	Area %/%
1	47.242	77202	3854077	50.049
2	49.090	73306	3846604	49.951

〈Chromatogram〉

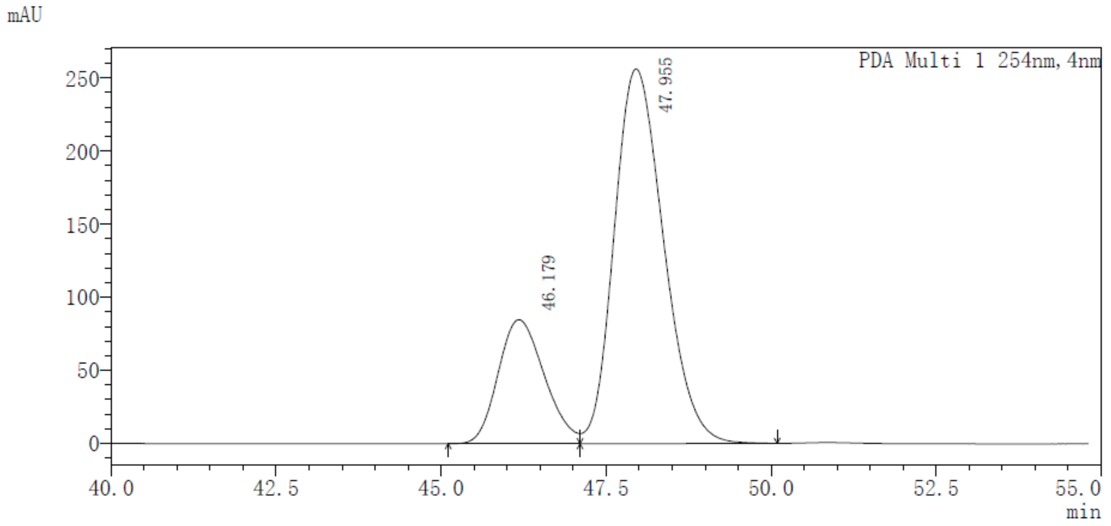

〈Peak Results〉

PDA Ch1 254nm

Index	Time/min	Height/mAU	Quantity/Area	Area %/%
1	46.179	84831	4130107	23.650
2	47.955	256273	13333700	76.350

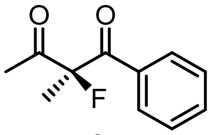

3o

〈Chromatogram〉

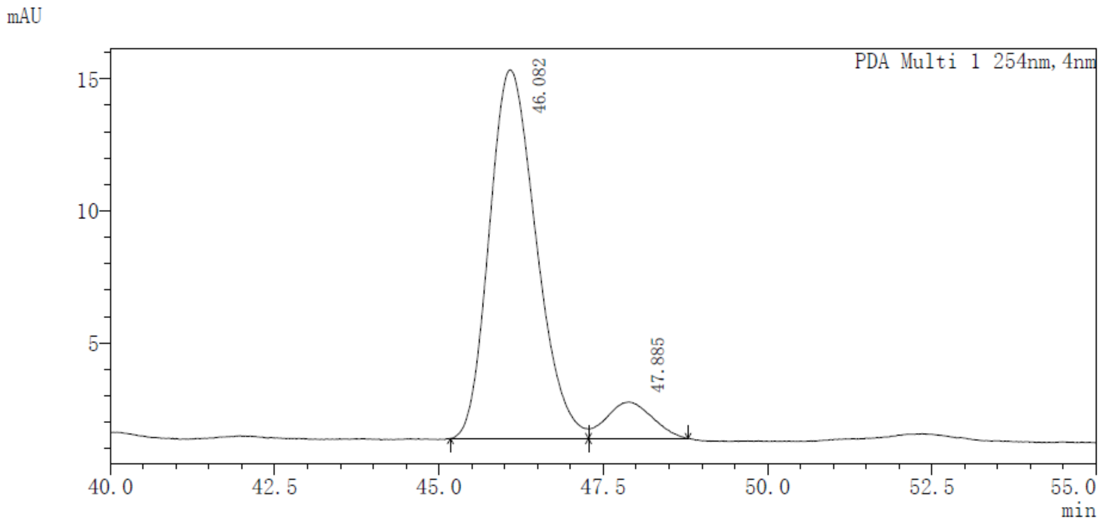

〈Peak Results〉

PDA Ch1 254nm

Index	Time/min	Height/mAU	Quantity/Area	Area %/%
1	46.082	13960	690859	91.175
2	47.885	1376	66866	8.825

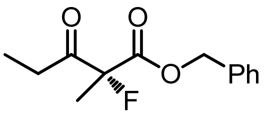

3p

<Chromatogram>

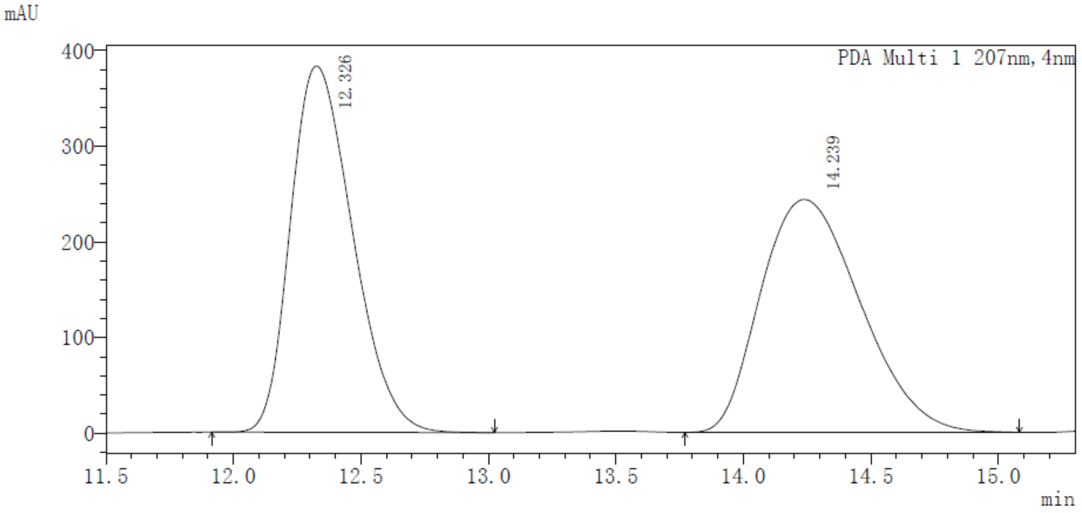

<Peak Results>

PDA Ch1 207nm

Index	Time/min	Height/mAU	Quantity/Area	Area %/%
1	12.326	382513	6490897	49.472
2	14.239	243351	6629563	50.528

<Chromatogram>

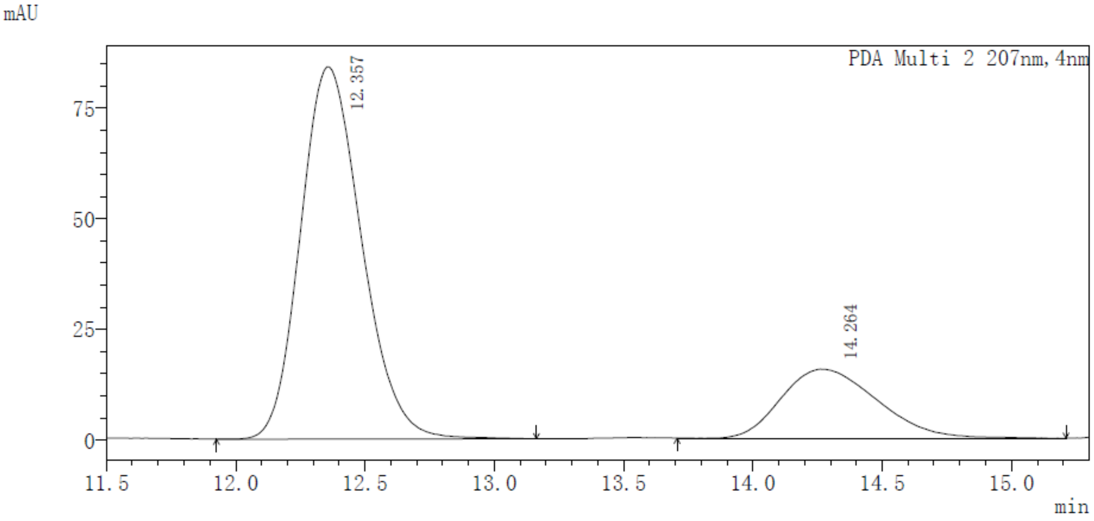

<Peak Results>

PDA Ch2 207nm

Index	Time/min	Height/mAU	Quantity/Area	Area %/%
1	12.357	84132	1384462	77.514
2	14.264	15584	401608	22.486

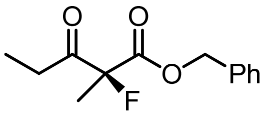

3p

〈Chromatogram〉

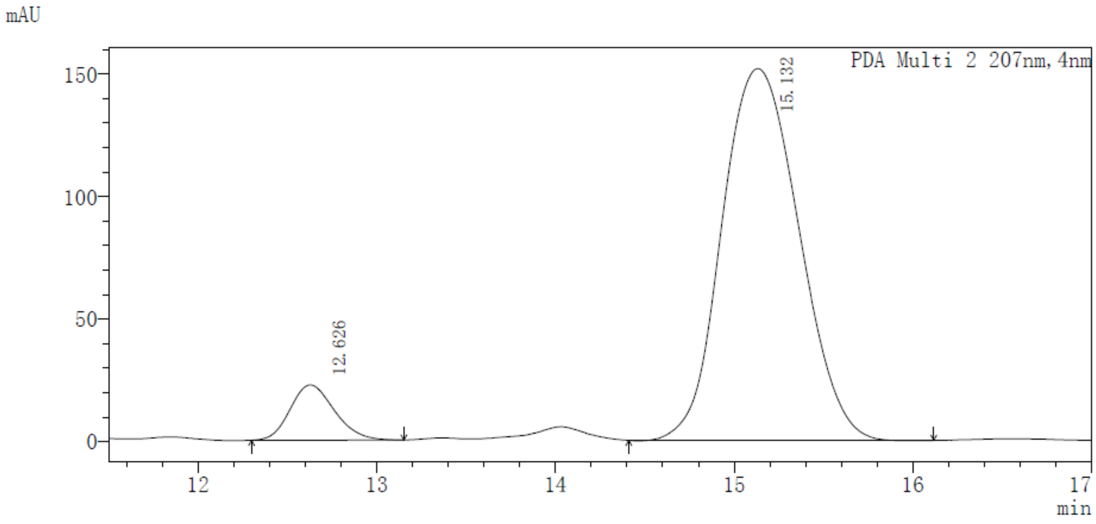

〈Peak Results〉

PDA Ch2 207nm

Index	Time/min	Height/mAU	Quantity/Area	Area %/%
1	12.626	22553	387298	7.846
2	15.132	151791	4549039	92.154

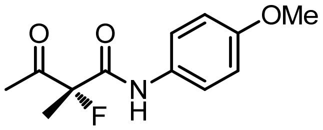

3q

<Chromatogram>

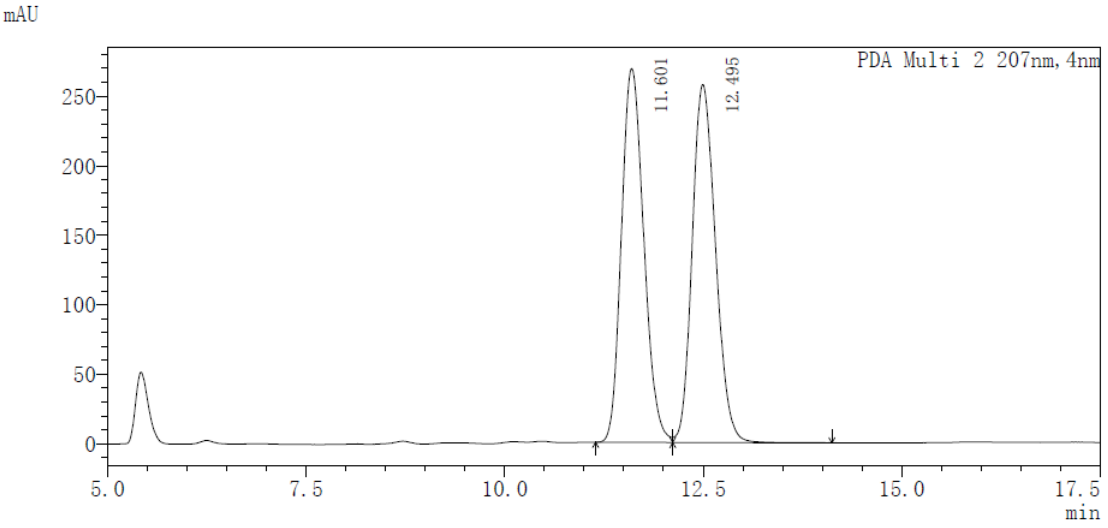

<Peak Results>

PDA Ch2 207nm

Index	Time/min	Height/mAU	Quantity/Area	Area %/%
1	11.601	268570	5255607	49.979
2	12.495	257377	5260082	50.021

<Chromatogram>

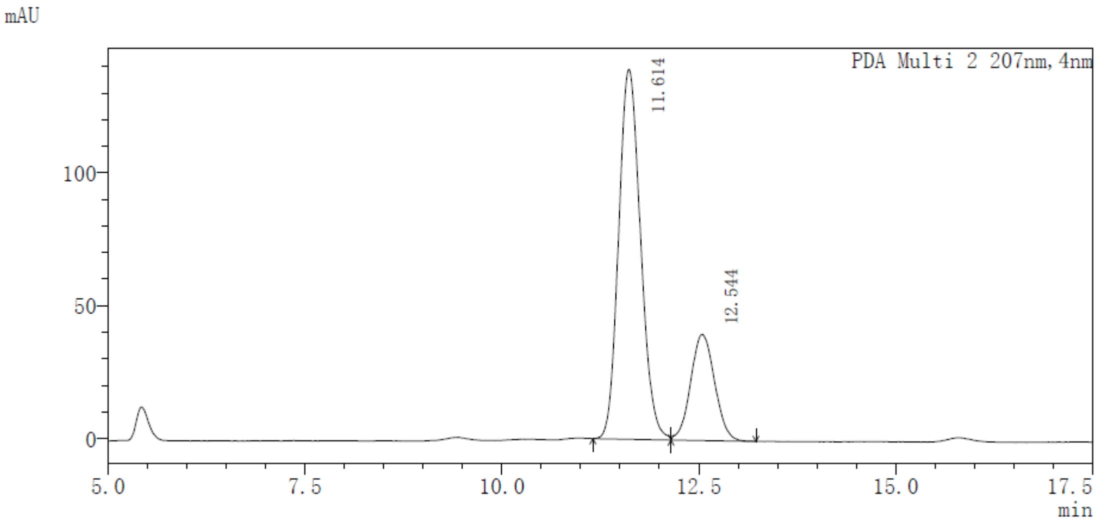

<Peak Results>

PDA Ch2 207nm

Index	Time/min	Height/mAU	Quantity/Area	Area %/%
1	11.614	139115	2693387	76.070
2	12.544	39936	847271	23.930

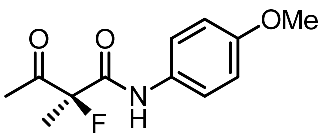

3q

〈Chromatogram〉

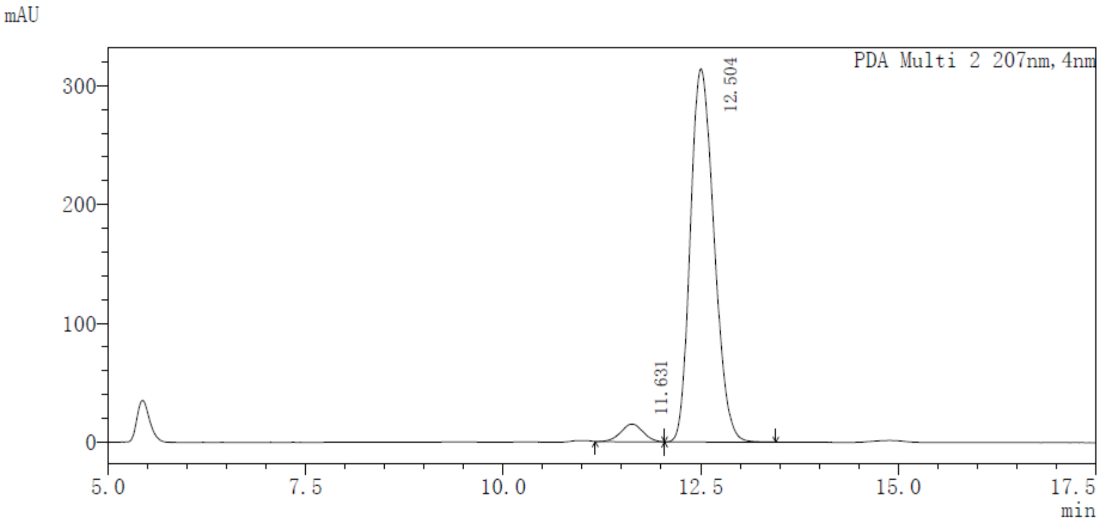

〈Peak Results〉

PDA Ch2 207nm

Index	Time/min	Height/mAU	Quantity/Area	Area %/%
1	11.631	14870	290277	4.241
2	12.504	313776	6554530	95.759

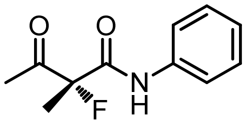

3r

〈Chromatogram〉

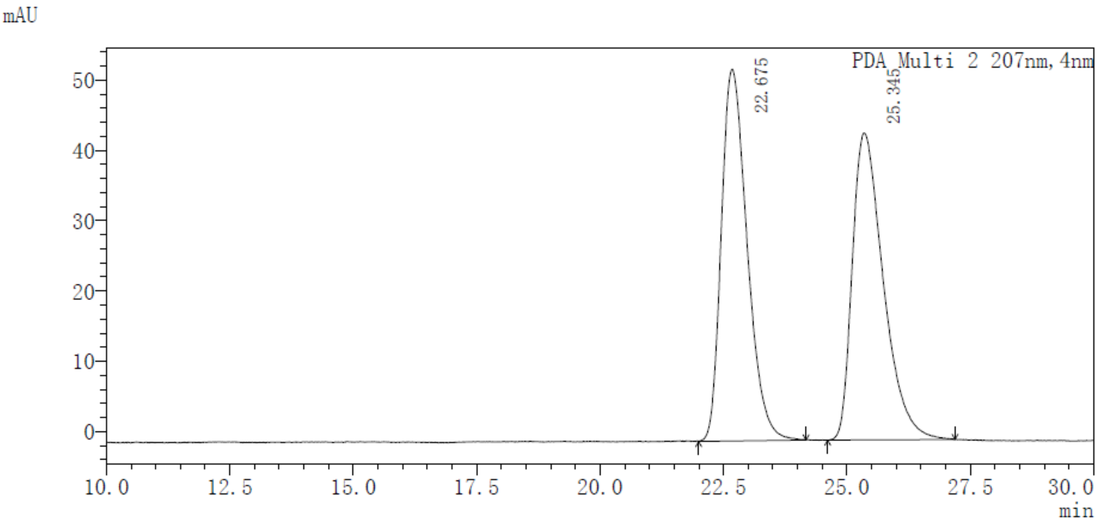

〈Peak Results〉

PDA Ch2 207nm

Index	Time/min	Height/mAU	Quantity/Area	Area %/%
1	22.675	52891	1925136	50.164
2	25.345	43668	1912542	49.836

〈Chromatogram〉

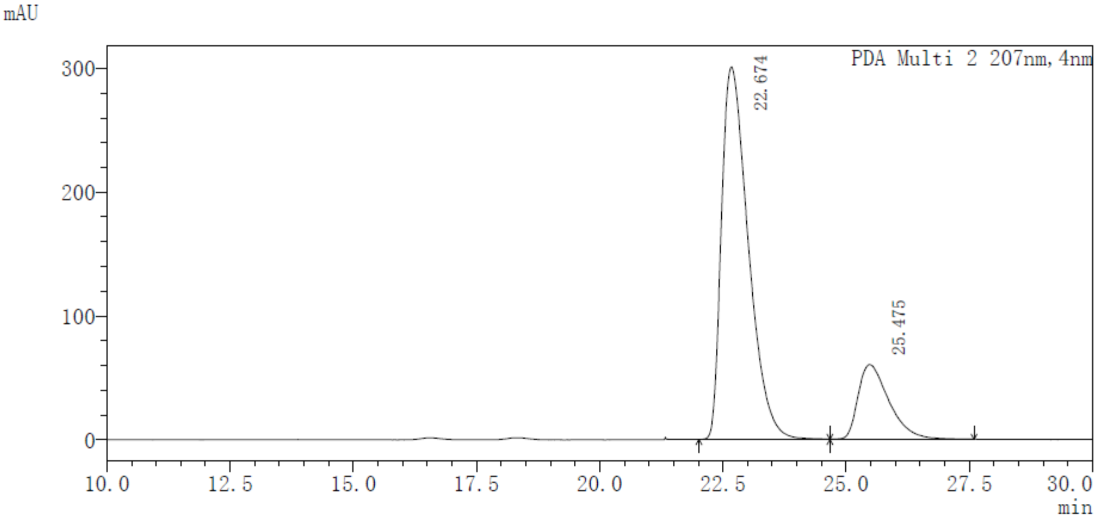

〈Peak Results〉

PDA Ch2 207nm

Index	Time/min	Height/mAU	Quantity/Area	Area %/%
1	22.674	301009	11395125	81.183
2	25.475	60443	2641155	18.817

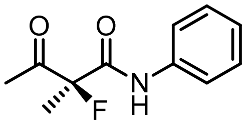

3r

<Chromatogram>

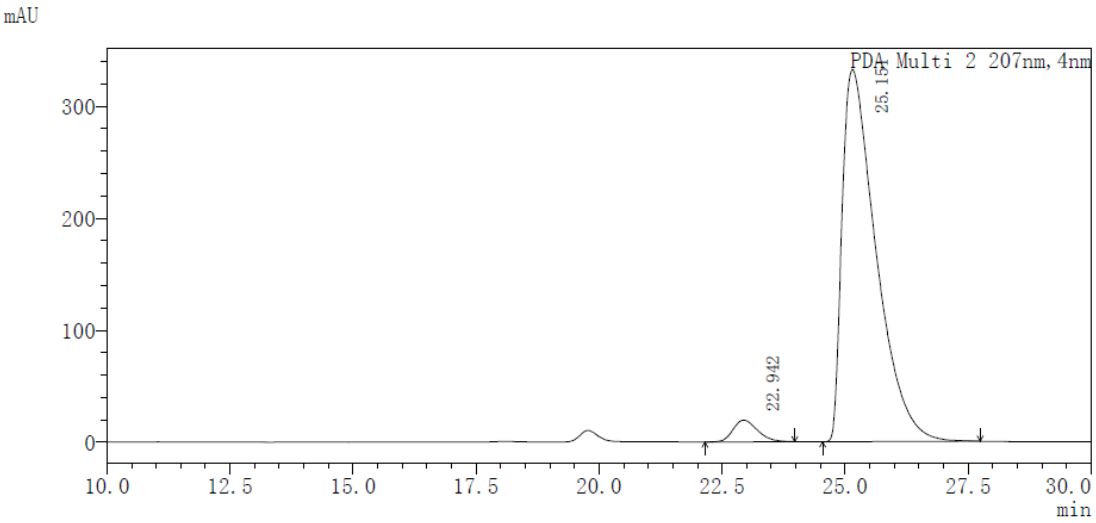

<Peak Results>

PDA Ch2 207nm

Index	Time/min	Height/mAU	Quantity/Area	Area %/%
1	22.942	19422	668565	4.024
2	25.151	332496	15944912	95.976

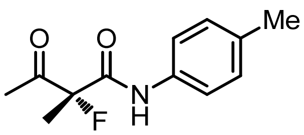

3s

〈Chromatogram〉

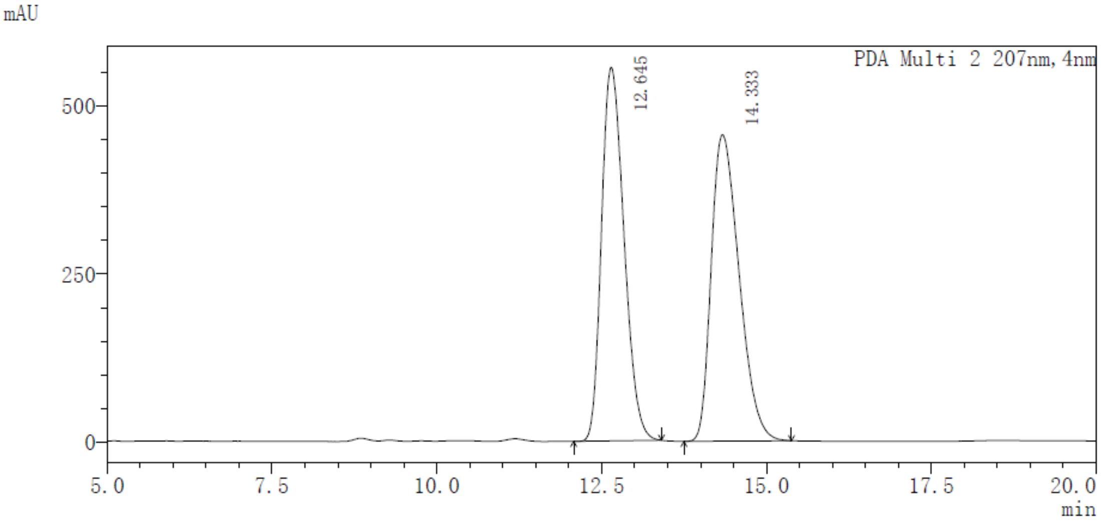

〈Peak Results〉

PDA Ch2 207nm

Index	Time/min	Height/mAU	Quantity/Area	Area %/%
1	12.645	555456	13191157	49.581
2	14.333	455718	13414105	50.419

〈Chromatogram〉

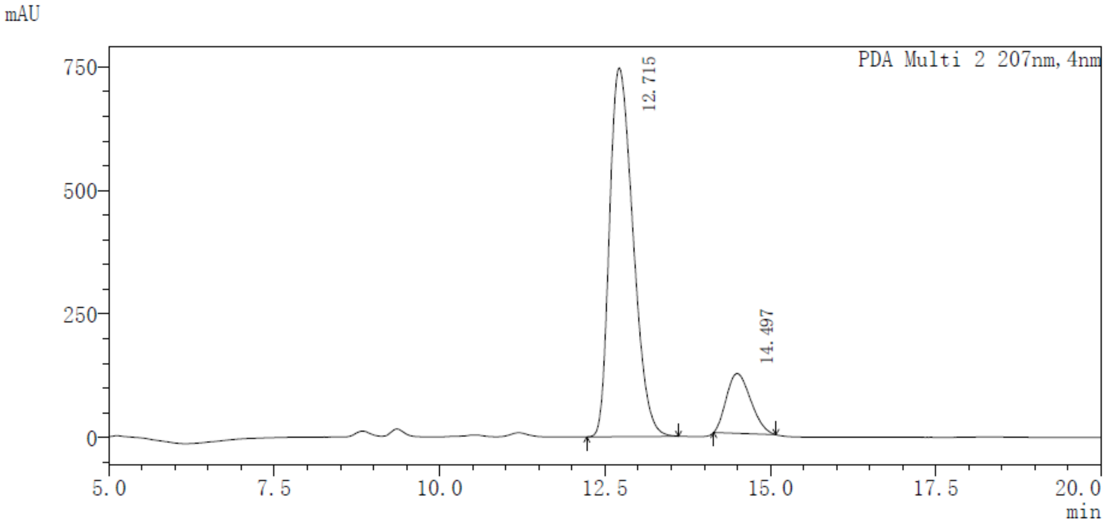

〈Peak Results〉

PDA Ch2 207nm

Index	Time/min	Height/mAU	Quantity/Area	Area %/%
1	12.715	746348	18507427	85.660
2	14.497	121009	3098220	14.340

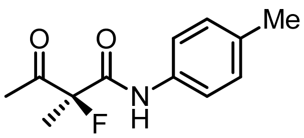

3s

⟨Chromatogram⟩

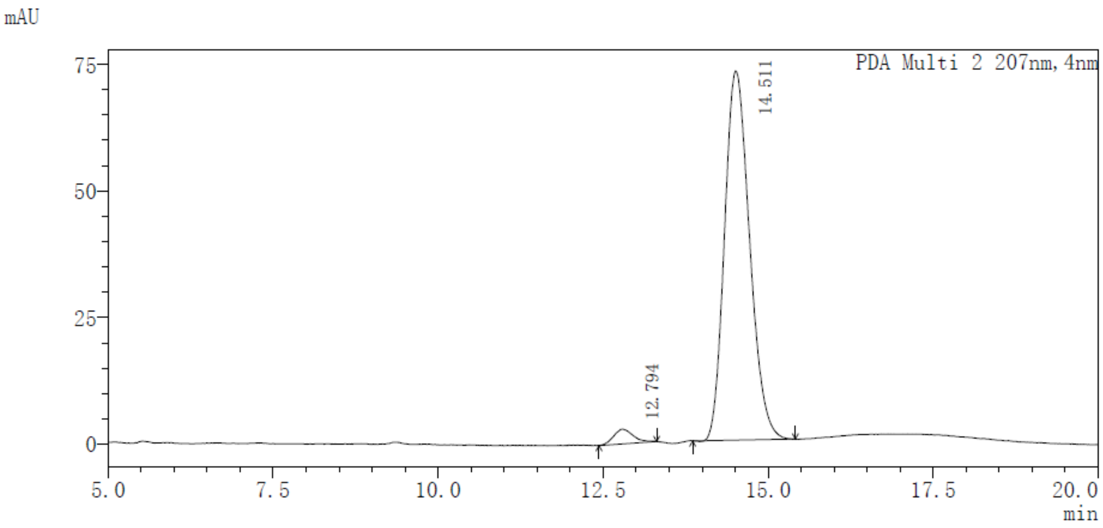

⟨Peak Results⟩

PDA Ch2 207nm

Index	Time/min	Height/mAU	Quantity/Area	Area %/%
1	12.794	2909	60183	3.031
2	14.511	72859	1925386	96.969

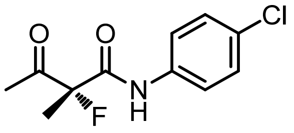

3t

〈Chromatogram〉

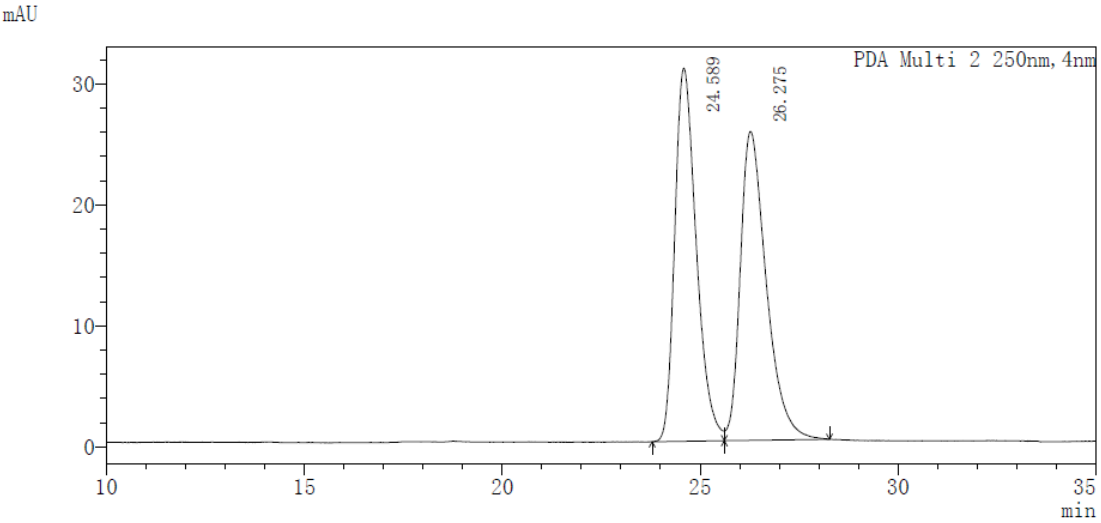

〈Peak Results〉

PDA Ch2 250nm

Index	Time/min	Height/mAU	Quantity/Area	Area %/%
1	24.589	30829	1165618	49.959
2	26.275	25524	1167528	50.041

〈Chromatogram〉

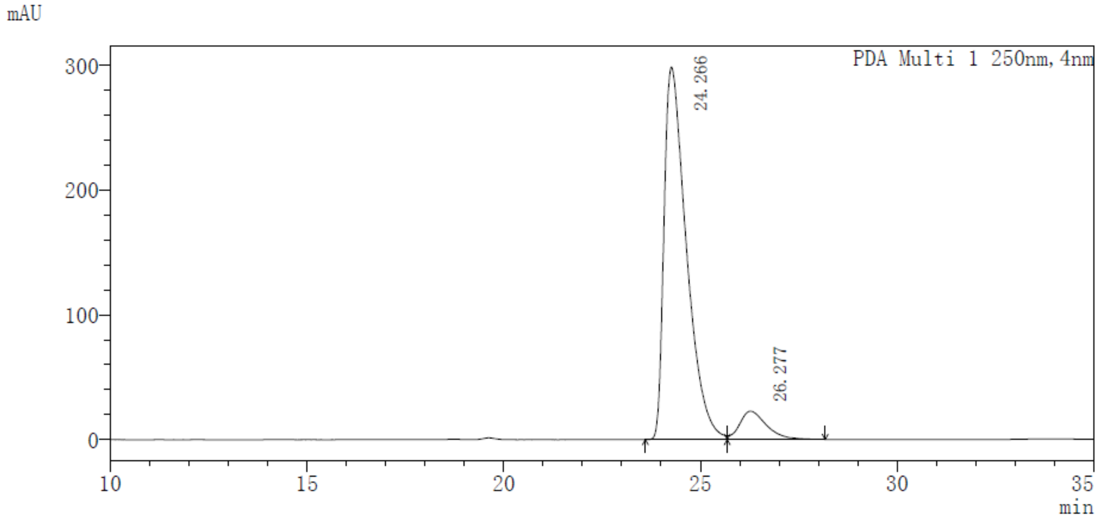

〈Peak Results〉

PDA Ch1 250nm

Index	Time/min	Height/mAU	Quantity/Area	Area %/%
1	24.266	298781	11810291	91.645
2	26.277	22747	1076776	8.355

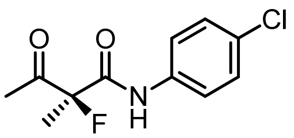

3t

〈Chromatogram〉

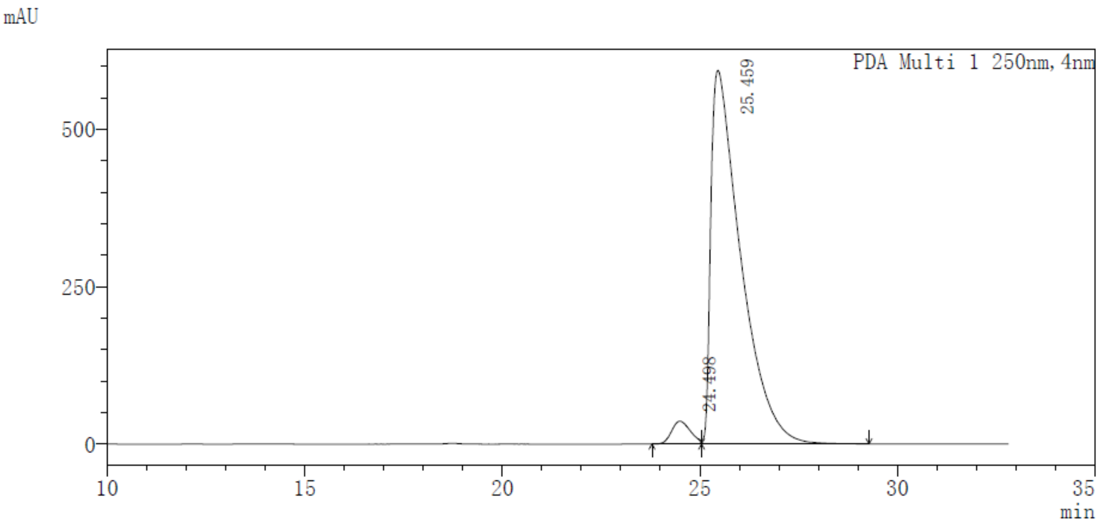

〈Peak Results〉

PDA Ch1 250nm

Index	Time/min	Height/mAU	Quantity/Area	Area %/%
1	24.498	36345	1204518	3.714
2	25.459	593687	31230915	96.286

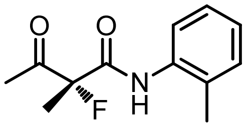

3u

⟨Chromatogram⟩

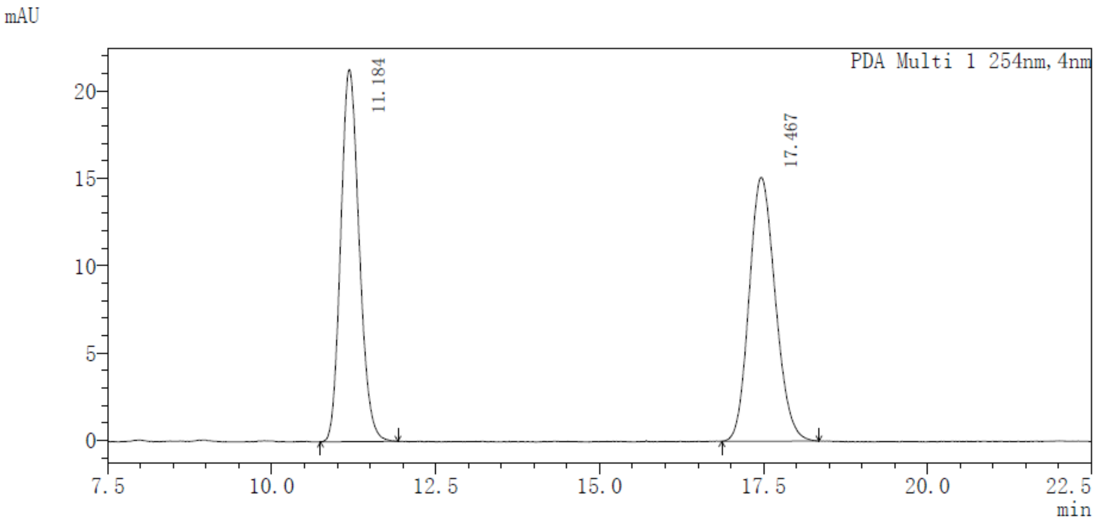

⟨Peak Results⟩

PDA Ch1 254nm

Index	Time/min	Height/mAU	Quantity/Area	Area %/%
1	11.184	21291	417121	49.813
2	17.467	15096	420250	50.187

⟨Chromatogram⟩

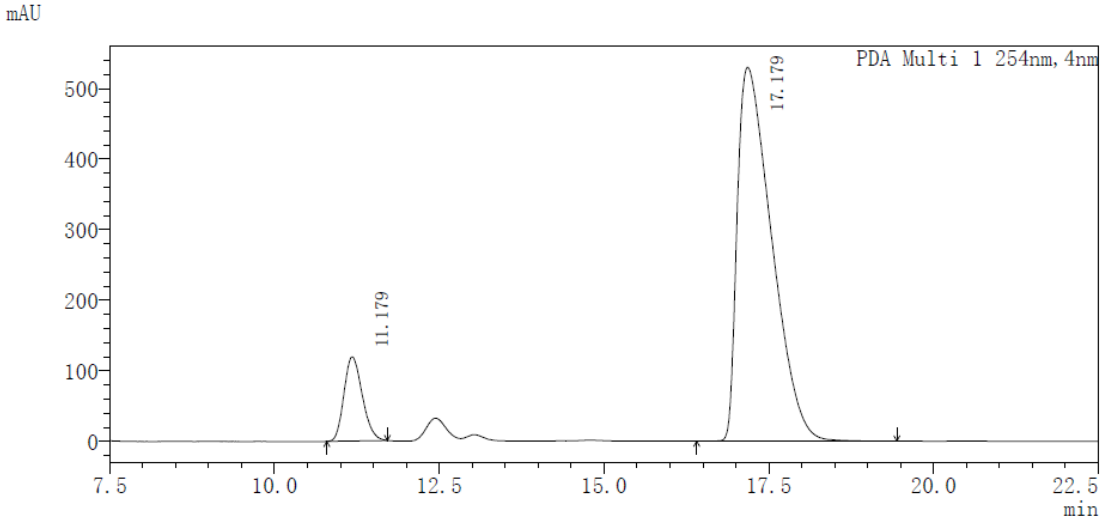

⟨Peak Results⟩

PDA Ch1 254nm

Index	Time/min	Height/mAU	Quantity/Area	Area %/%
1	11.179	119251	2309123	10.734
2	17.179	529418	19202959	89.266

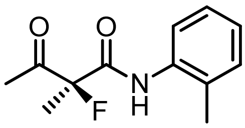

3u

〈Chromatogram〉

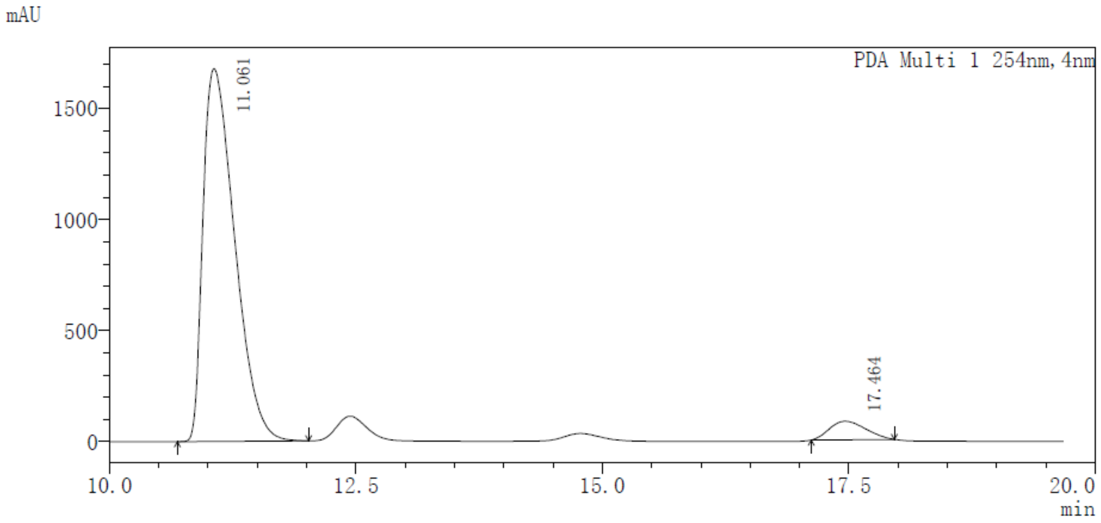

〈Peak Results〉

PDA Ch1 254nm

Index	Time/min	Height/mAU	Quantity/Area	Area %/%
1	11.061	1678830	37543174	94.620
2	17.464	83371	2134663	5.380

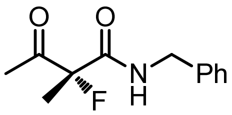

3v

〈Chromatogram〉

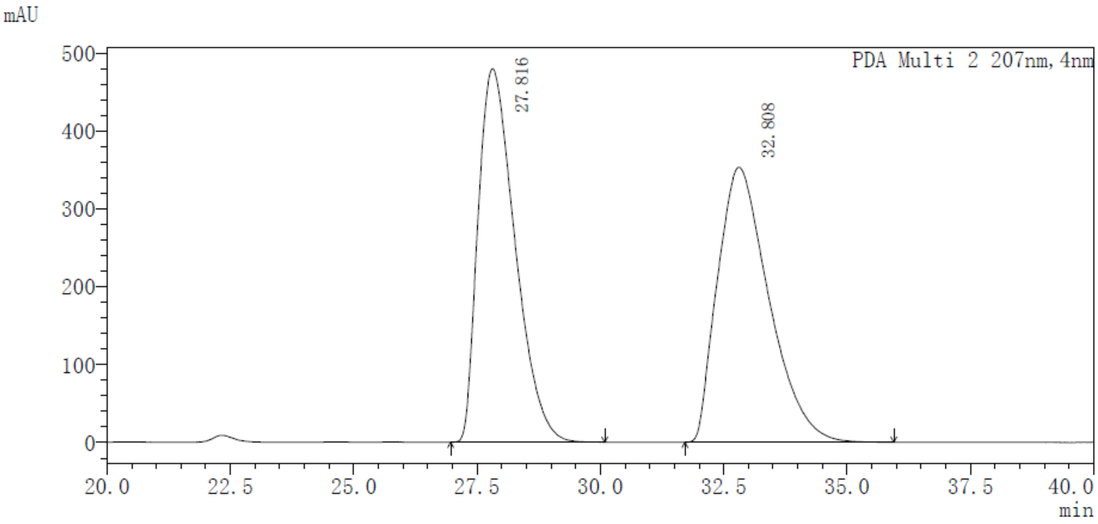

〈Peak Results〉

PDA Ch2 207nm

Index	Time/min	Height/mAU	Quantity/Area	Area %/%
1	27.816	479868	25014143	49.463
2	32.808	353485	25556806	50.537

〈Chromatogram〉

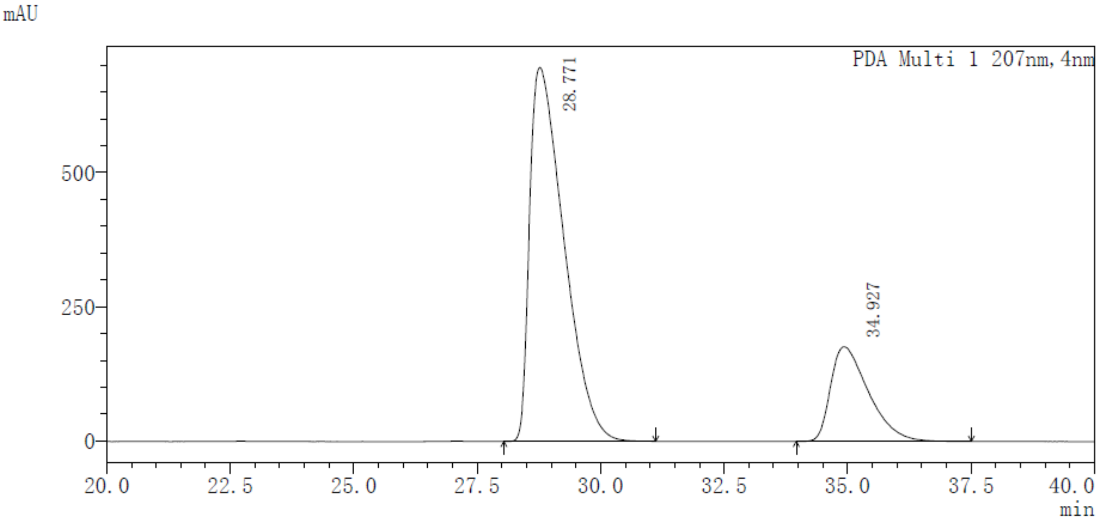

〈Peak Results〉

PDA Ch1 207nm

Index	Time/min	Height/mAU	Quantity/Area	Area %/%
1	28.771	696349	33615304	78.071
2	34.927	176379	9441870	21.929

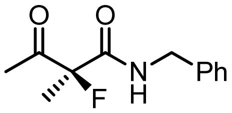

3v

〈Chromatogram〉

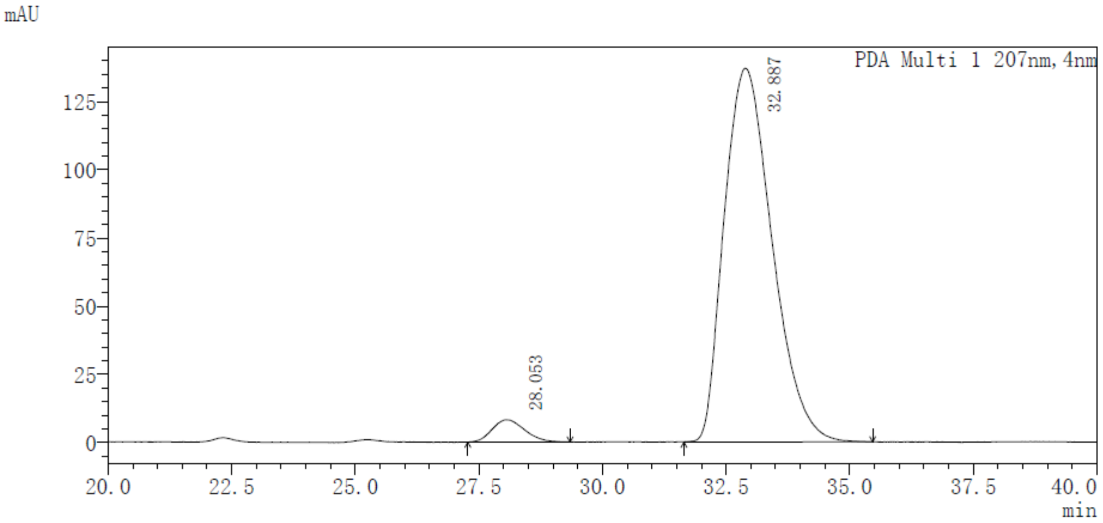

〈Peak Results〉

PDA Ch1 207nm

Index	Time/min	Height/mAU	Quantity/Area	Area %/%
1	28.053	8197	374474	3.890
2	32.887	136991	9251971	96.110

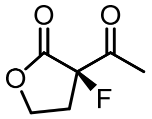

3w

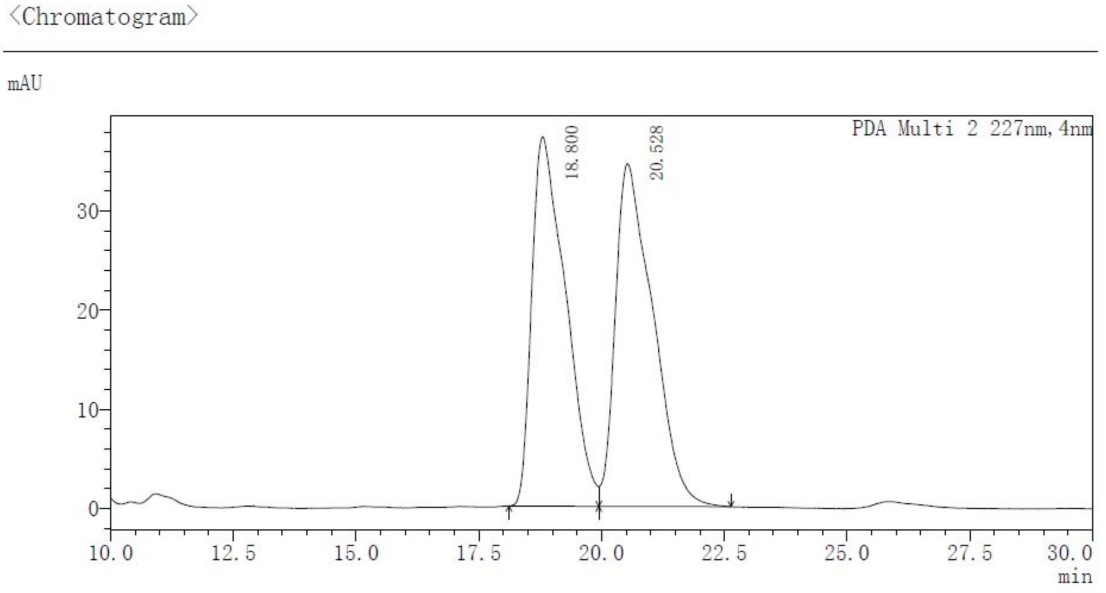

〈Peak Results〉

PDA Ch2 227nm

Index	Time/min	Height/mAU	Quantity/Area	Area %/%
1	18.800	37256	1787004	49.315
2	20.528	34561	1836661	50.685

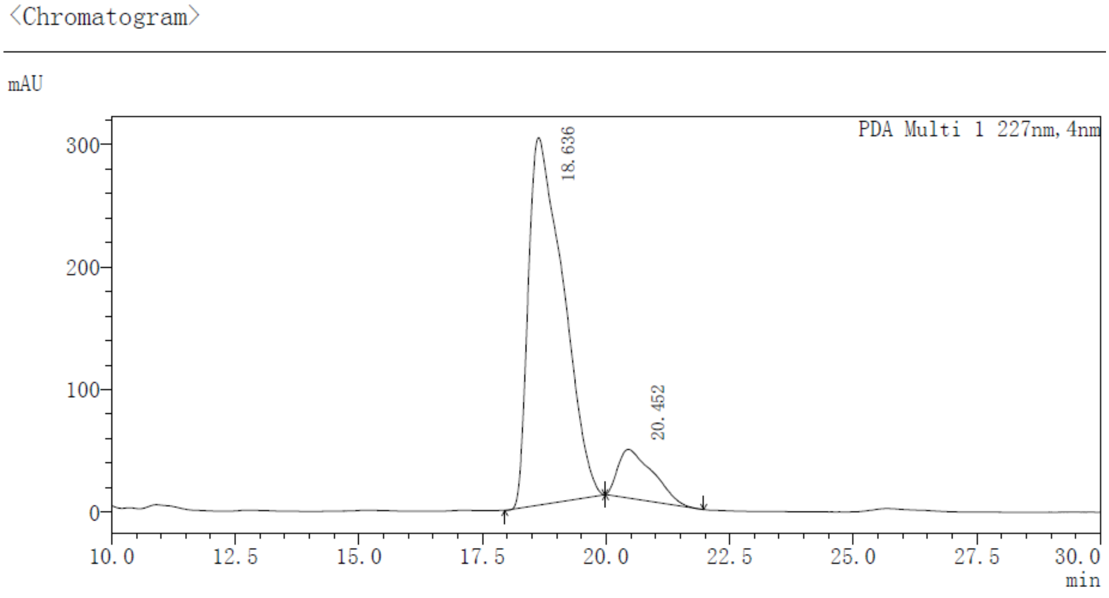

〈Peak Results〉

PDA Ch1 227nm

Index	Time/min	Height/mAU	Quantity/Area	Area %/%
1	18.636	299381	14678765	88.144
2	20.452	39659	1974365	11.856

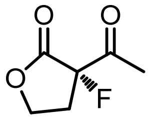

3w

Catalyst II/DNBA II results:

<Chromatogram>

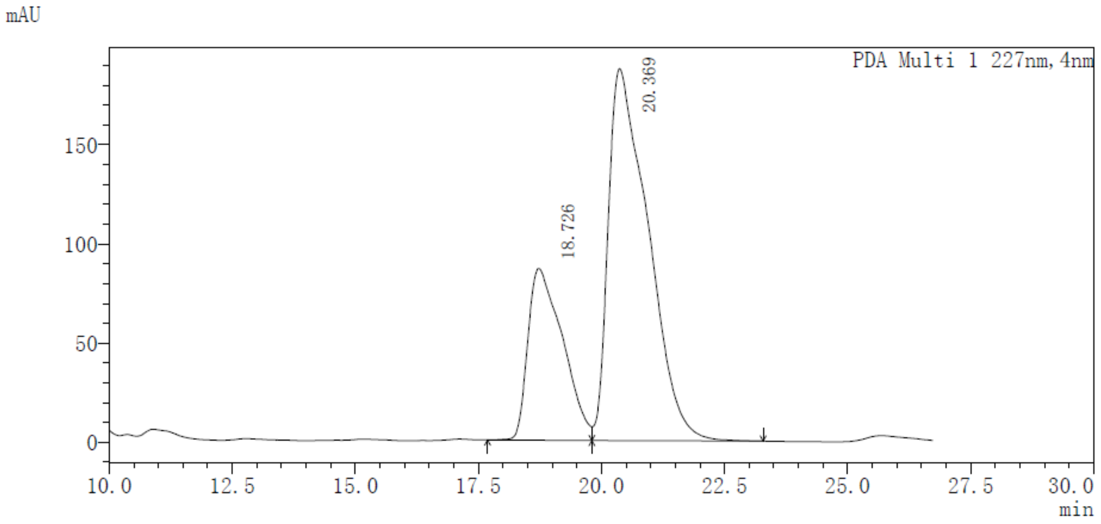

<Peak Results>

PDA Ch1 227nm

Index	Time/min	Height/mAU	Quantity/Area	Area %/%
1	18.726	86542	4261156	28.726
2	20.369	187360	10572397	71.274

Catalyst III/TfOH results:

<Chromatogram>

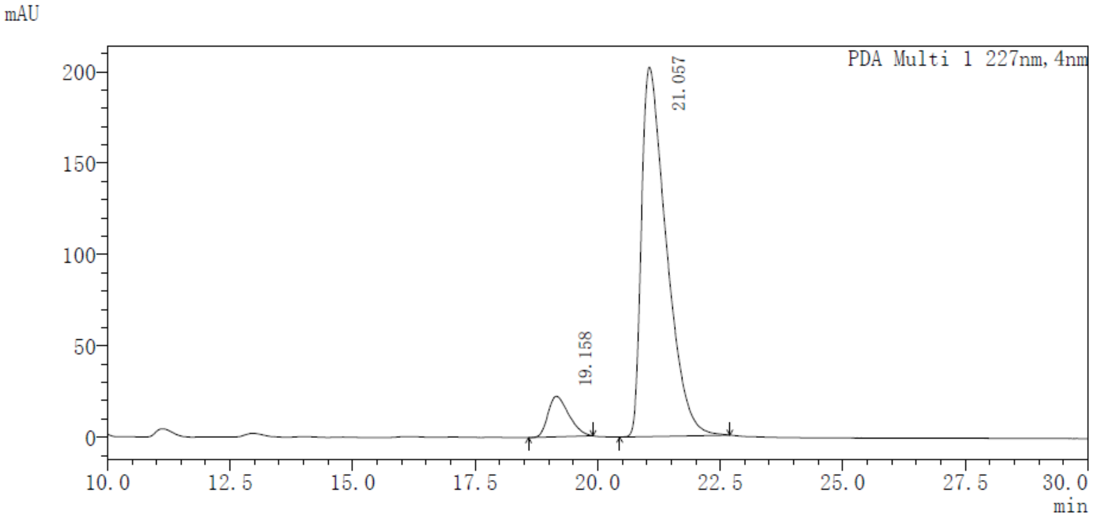

<Peak Results>

PDA Ch1 227nm

Index	Time/min	Height/mAU	Quantity/Area	Area %/%
1	19.158	22157	653081	8.374
2	21.057	202090	7145536	91.626

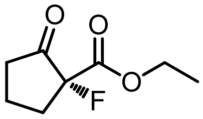

3x

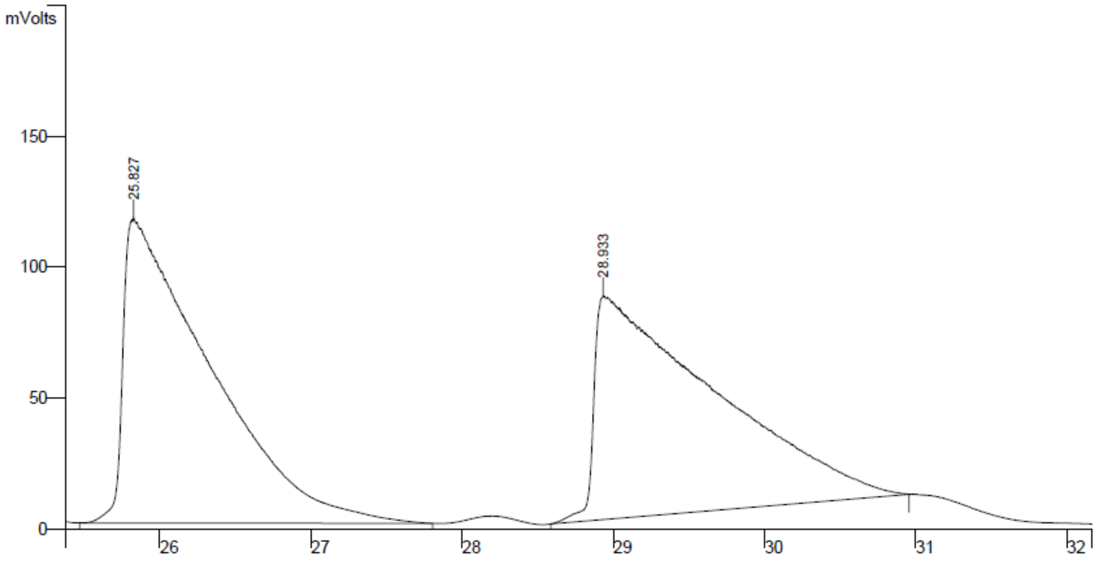

Peak No	Peak Name	Result ()	Ret. Time (min)	Time Offset (min)	Area (counts)	Rel Ret Time	Sep. Code	Width 1/2 (sec)
1		49.6769	25.827	0.000	4645817	0.00	BB	35.7
2		50.3231	28.933	0.000	4706251	0.00	BB	57.8
	**Totals**	**100.0000**		**0.000**	**9352068**			

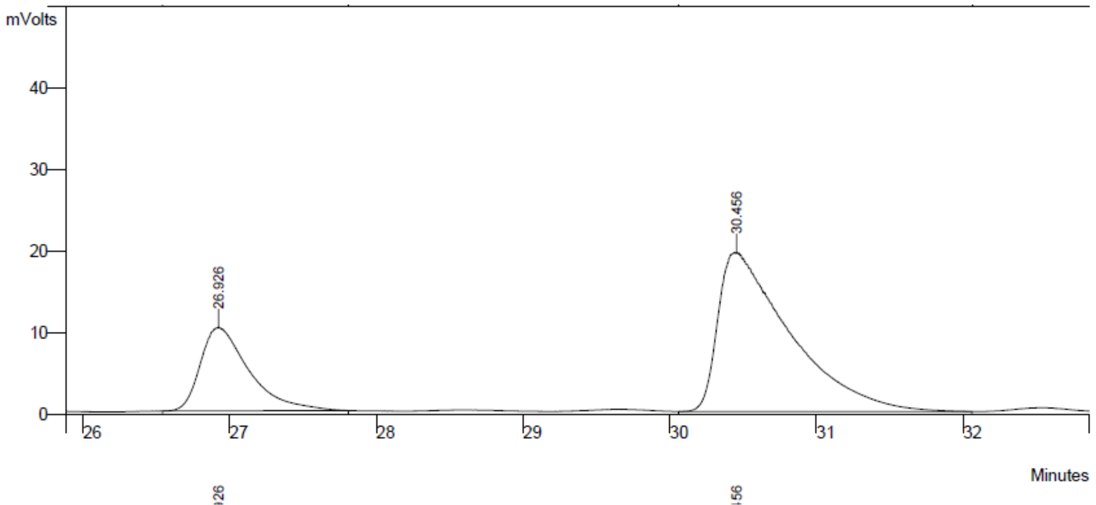

Peak No	Peak Name	Result ()	Ret. Time (min)	Time Offset (min)	Area (counts)	Rel Ret Time	Sep. Code	Width 1/2 (sec)
1		26.1639	26.926	0.000	235439	0.00	BB	20.2
2		73.8361	30.456	0.000	664421	0.00	BB	29.8
	**Totals**	**100.0000**		**0.000**	**899860**			

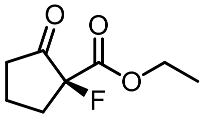

3x

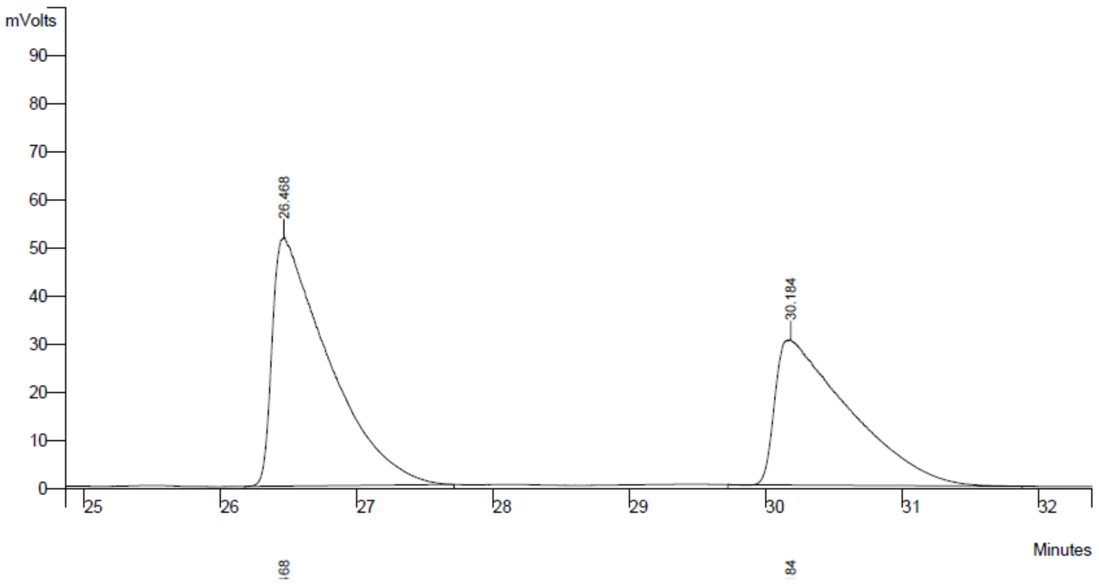

Peak No	Peak Name	Result ()	Ret. Time (min)	Time Offset (min)	Area (counts)	Rel Ret Time	Sep. Code	Width 1/2 (sec)
1		56.5710	26.468	0.000	1484207	0.00	BB	25.9
2		43.4290	30.184	0.000	1139409	0.00	BB	34.6
	**Totals**	**100.0000**		**0.000**	**2623616**			

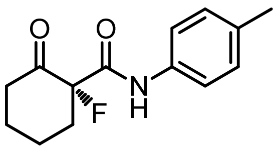

3y

〈Chromatogram〉

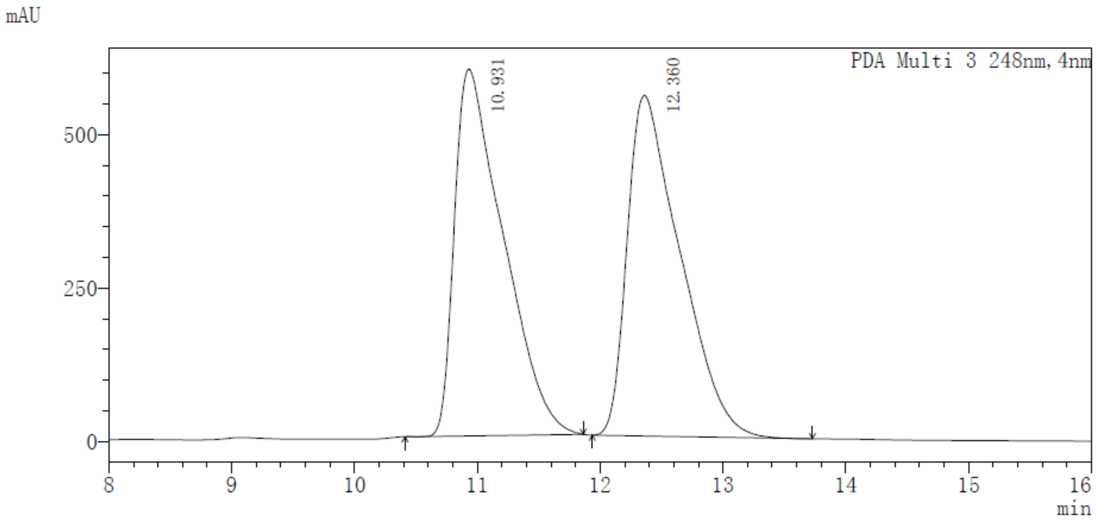

〈Peak Results〉

PDA Ch3 248nm

Index	Time/min	Height/mAU	Quantity/Area	Area %/%
1	10.931	597668	16500000	49.747
2	12.360	555243	16668061	50.253

〈Chromatogram〉

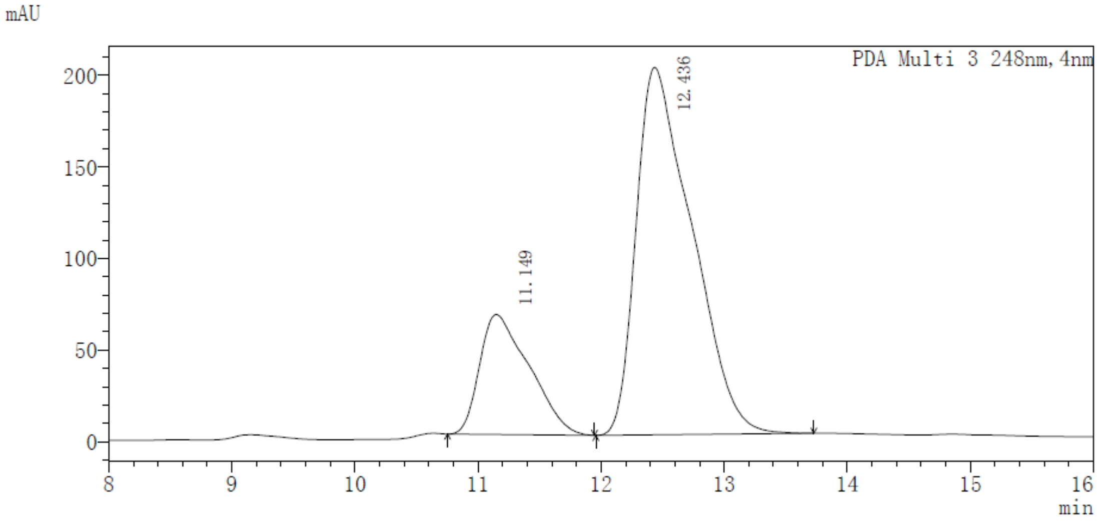

〈Peak Results〉

PDA Ch3 248nm

Index	Time/min	Height/mAU	Quantity/Area	Area %/%
1	11.149	65537	1837757	22.495
2	12.436	200520	6331756	77.505

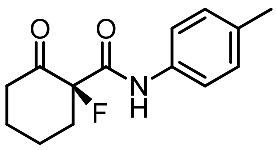

3y

〈Chromatogram〉

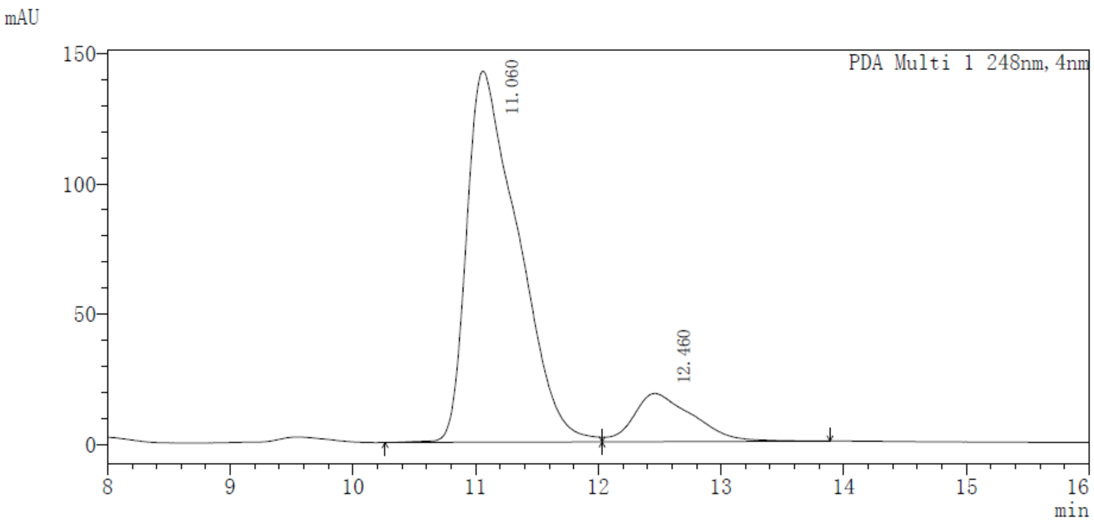

〈Peak Results〉

PDA Ch1 248nm

Index	Time/min	Height/mAU	Quantity/Area	Area %/%
1	11.060	142264	4149104	87.101
2	12.460	18470	614469	12.899
